# Evaluating the Genetic Capacity of *Mycoplasmas* for Coenzyme A Biosynthesis in a Search for New Anti-mycoplasma Targets

**DOI:** 10.3389/fmicb.2021.791756

**Published:** 2021-12-20

**Authors:** Tertius Alwyn Ras, Erick Strauss, Annelise Botes

**Affiliations:** Department of Biochemistry, Stellenbosch University, Stellenbosch, South Africa

**Keywords:** *Mycoplasma*, drug development, coenzyme A biosynthesis, biosynthetic variability, host dependence, dephospho-coenzyme A kinase, pantothenate kinase

## Abstract

*Mycoplasmas* are responsible for a wide range of disease states in both humans and animals, in which their parasitic lifestyle has allowed them to reduce their genome sizes and curtail their biosynthetic capabilities. The subsequent dependence on their host offers a unique opportunity to explore pathways for obtaining and producing cofactors – such as coenzyme A (CoA) – as possible targets for the development of new anti-mycoplasma agents. CoA plays an essential role in energy and fatty acid metabolism and is required for membrane synthesis. However, our current lack of knowledge of the relevance and importance of the CoA biosynthesis pathway in mycoplasmas, and whether it could be bypassed within their pathogenic context, prevents further exploration of the potential of this pathway. In the universal, canonical CoA biosynthesis pathway, five enzymes are responsible for the production of CoA. Given the inconsistent presence of the genes that code for these enzymes across *Mycoplasma* genomes, this study set out to establish the genetic capacity of mycoplasmas to synthesize their own CoA *de novo*. Existing functional annotations and sequence, family, motif, and domain analysis of protein products were used to determine the existence of relevant genes in *Mycoplasma* genomes. We found that most *Mycoplasma* species do have the genetic capacity to synthesize CoA, but there was a differentiated prevalence of these genes across species. Phylogenetic analysis indicated that the phylogenetic position of a species could not be used to predict its enzyme-encoding gene combinations. Despite this, the final enzyme in the biosynthesis pathway – dephospho-coenzyme A kinase (DPCK) – was found to be the most common among the studied species, suggesting that it has the most potential as a target in the search for new broad-spectrum anti-mycoplasma agents.

## Introduction

Mycoplasmas are responsible for a wide range of diseases in humans and animals, impacting health and economic activity – especially in the agricultural sector. They are cell wall-less bacteria of the class Mollicutes that are among the smallest known organisms capable of self-replication (Razin et al., 1998), with reduced genome sizes that have resulted in severely curtailed biosynthetic capabilities (Rivera and Cedillo, 2015). Consequently, many mycoplasmas lead a parasitic lifestyle, in which they are dependent on their host for essential nutrients. This symbiotic relationship presents both a challenge and an opportunity for the development of new treatments for *Mycoplasma* infections. The challenge arises from the close relationship between pathogen and host, which makes it difficult to specifically target the *Mycoplasma*. In addition to this, antimicrobial resistance against commonly used cell wall synthesis inhibitors is a serious concern (Meyer and Van Rossum, 2014; Chernova et al., 2016) However, the mycoplasma’s dependence on certain host-derived growth factors offers a unique opportunity for the discovery of inhibitors of a process or pathway that is central to this interaction, and that would be detrimental to the survival of the organism.

In this context, the mechanism by which mycoplasmas obtain the central metabolic cofactor coenzyme A (CoA) presents itself as an especially relevant case study. First, the enzymes involved in CoA biosynthesis have long been regarded as possible targets for antimicrobial development given their essential role in central energy and fatty acid metabolism, and the prediction that the bacterial enzymes could selectively be inhibited based on their divergence from those of their eukaryotic hosts (Spry et al., 2008; Moolman et al., 2014; Balish and Distelhorst, 2016). Second, CoA is also required for membrane biosynthesis and therefore growth of *Mycoplasma* (Pollack et al., 1997). Third, *in vitro* studies have shown that some of the genes encoding the enzymes responsible for CoA biosynthesis in mycoplasmas are dispensable. The extreme example is the organism with the “minimal bacterial genome” discovered following the genome reduction of *Mycoplasma mycoides*, which did not have any of the enzymes required for producing its own CoA (Yus et al., 2009; Hutchison et al., 2016). While *de novo* CoA biosynthesis may therefore also be a worthwhile target for the development of new anti-mycoplasma agents, our current lack of knowledge of the relevance and importance of this process in mycoplasmas, and whether it could be bypassed within their pathogenic context, prevents further exploration of the potential of this pathway.

**Figure 1:**
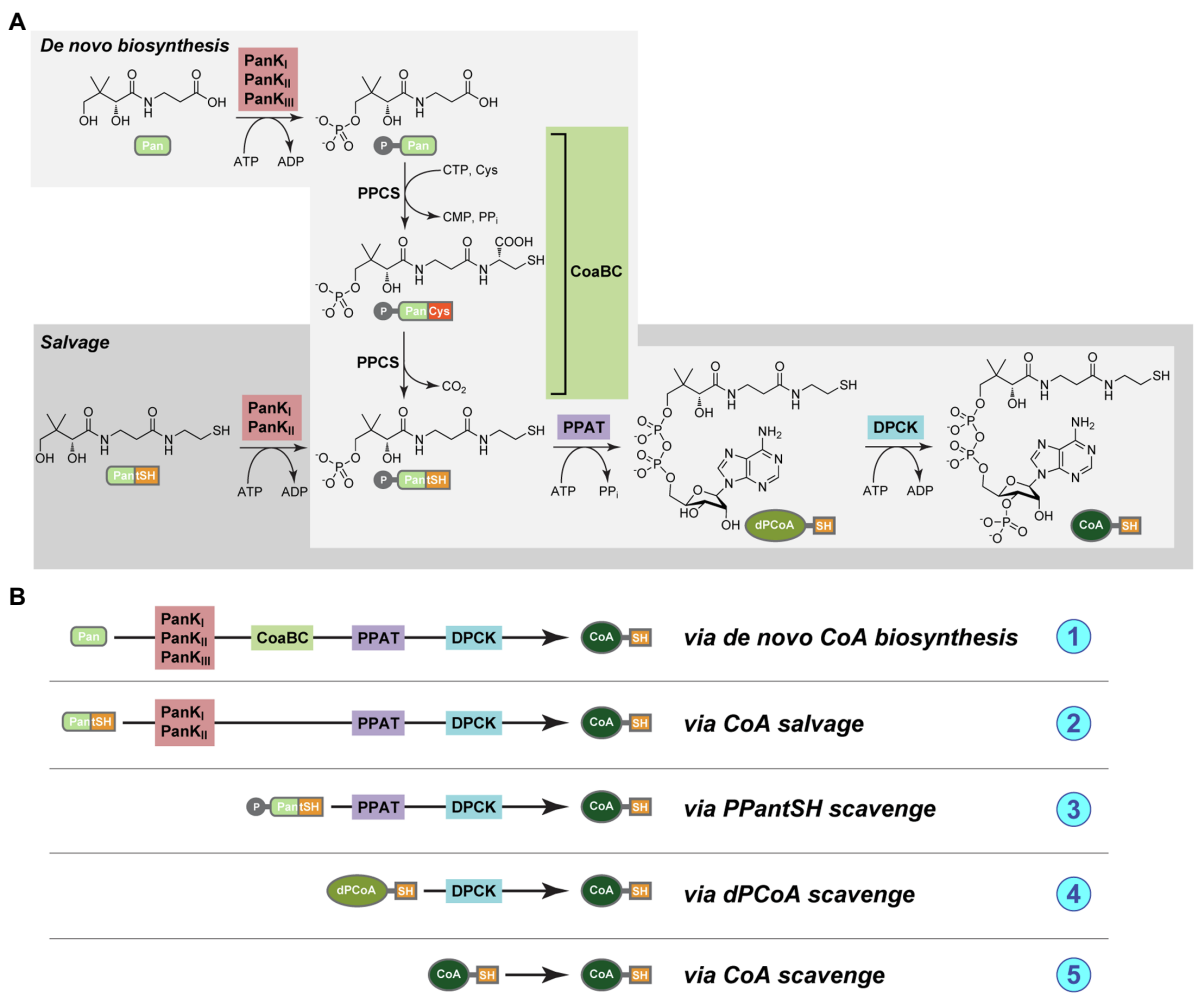
**(A)** The pathways for *de novo* coenzyme A biosynthesis (light gray box) and coenzyme A (CoA) salvage (dark gray box). The compounds are pantothenic acid (Pan, or vitamin B_5_), 4′-phosphopantothenic acid (P-Pan), pantetheine (PantSH), 4′-phosphopantetheine (PPantSH), dephospho-coenzyme A (dPCoA), CoA, and the enzymes pantothenate kinase (PanK, in red box), CoaBC [a bifunctional protein with both phosphopantothenoylcysteine synthetase (PPCS) and phosphopantothenoylcysteine decarboxylase (PPCDC) activities, in green box], phosphopantetheine adenylyltransferase (PPAT, in purple box), and finally dephospho-coenzyme A kinase (DPCK, blue box; Strauss, 2010). **(B)** A schematic representation of the various mechanisms whereby CoA could potentially be obtained, and of the enzyme combinations that would be required in each case. (1) *Via de novo* biosynthesis from Pan; (2) *via* salvage from PantSH; (3) *via* scavenge of PPantSH from the host or environment; (4) *via* scavenge of dPCoA from the host or environment; and (5) *via* scavenge of CoA from the host or environment.

In the universal, canonical CoA biosynthesis pathway five enzymes are responsible for the conversion of the precursor substrate pantothenic acid (Pan, or vitamin B_5_) into CoA (Figure 1A). The first enzyme is pantothenate kinase (PanK), followed by CoaBC – a bifunctional protein in bacteria that has both phosphopantothenoylcysteine synthetase (PPCS) and phosphopantothenoylcysteine decarboxylase (PPCDC) activities. This is followed by phosphopantetheine adenylyltransferase (PPAT), with the last enzyme in the pathway being dephospho-coenzyme A kinase (DPCK; Strauss, 2010). An important possible deviation from the canonical pathway entails the use of the CoA degradation product pantetheine (PantSH) as an alternative substrate by PanK in a CoA salvage mechanism that allows for the bypassing of the PPCS and PPCDC activities of the CoaBC protein (Figure 1A). However, this is only possible for a certain subset of PanK enzymes, which occur as three distinct types: type I (PanK_I_) and type III PanK (PanK_III_) enzymes (encoded for by the *coaA* and *coaX* genes, respectively) that are found in prokaryotes, and type II PanKs (PanK_II_) found in eukaryotes and selected *Staphylococcus* and *Bacillus* species. In particular, PanK_III_ enzymes are not able to use PantSH as substrate and organisms with this type of PanK can therefore not make use of the CoA salvage pathway and bypass CoaBC. In addition, there has also been some evidence that certain cells are able to take up more advanced CoA biosynthetic intermediates, suggesting the possible existence of mechanisms whereby CoA could be obtained through the action of only PPAT and DPCK, or even DPCK alone (Sibon and Strauss, 2016). A schematic summary of all these mechanisms is presented in Figure 1B.

With this as background, we set out to establish the genetic capacity of *Mycoplasma* to synthesize their own CoA *de novo* using Pan as substrate, or through some truncated variations of the canonical pathway. Such an analysis is particularly warranted due to the inconsistent presence of the genes that code for the CoA biosynthesis enzymes across *Mycoplasma* genomes (Daugherty et al., 2002; Spry et al., 2008; Yus et al., 2009; De Jonge et al., 2013). While this could be a reflection of the parasitic lifestyle of these organisms, it could also be a result of insufficient annotation, since *Mycoplasma* genomes typically contain a large number of hypothetical protein-coding genes. Distinguishing between these possibilities is crucial if CoA biosynthesis is to be considered to hold any realistic potential for anti-mycoplasma drug development.

In this study, we determine the existence of genes encoding the CoA biosynthesis pathway enzymes among *Mycoplasma* genomes using existing functional annotations as well as sequence, family, motif, and domain analysis of protein products. We discovered an unexpectedly differentiated prevalence of these genes across *Mycoplasma* species, with a few specific combinations emerging. We therefore used phylogenetic analysis to determine the evolutionary relationship of the *Mycoplasma* species and the functional relationship of their CoA biosynthetic enzymes using 16S rRNA and amino acid data, respectively. This allowed us to uncover the enzyme targets that would hold the most potential for the development of new broad-spectrum or species-specific anti-mycoplasma agents.

## Materials and Methods

### Selection of *Mycoplasma* Genomes Used in This Study

The annotated genomes of 62 *Mycoplasma* species were investigated in this study with some genomes being complete and others only available at contiguous sequence (contig) level (Supplementary Table 1). The 16S rRNA phylogeny of Wium et al. (2015) was used as starting point to select 28 *Mycoplasma* species that also had CoA biosynthesis pathway information available on the Kyoto Encyclopedia of Genes and Genomes (KEGG) pathway and/or SEED viewer subsystems databases. The species were selected to represent the typical 16S rRNA phylogenetic groupings (Weisburg et al., 1989; Johansson et al., 1998; Johansson and Petterson, 2002) as well as a range of hosts. To further increase the variety, additional species with pathway information were selected from the KEGG, SEED, and National Centre for Biotechnology Information (NCBI) databases. Due to our interest in ostrich-infecting *Mycoplasma*, the genome of *Mycoplasma* sp. Ms02 was also included in this study.

Given the exploratory nature of this study only one strain of a selected species was used. All the amino acid sequences were downloaded from the NCBI database and accession numbers are given in Supplementary Table 2.

### Determining the Prevalence of Enzyme-Encoding Genes Using Existing Functional Annotations

Thirty-seven of the selected *Mycoplasma* species contained annotated gene information for one or more enzymes of the CoA biosynthesis pathway in the KEGG and/or SEED databases (Overbeek et al., 2005). The results of both databases were combined and compared to that on the NCBI database from which the amino acid sequences of the annotated genes were obtained. For species selected from NCBI that had no corresponding KEGG/SEED information available, the enzyme encoding genes were identified within their genomes using blastp searches with relevant amino acid sequences of annotated enzymes, identified *via* the KEGG or SEED databases, as query.

All the associated amino acid sequences were downloaded from the NCBI protein database for further protein a phylogenetic analysis (Pruitt et al., 2012). The arrangement of enzyme-encoding genes relative to one another within a genome was also determined in species with a complete genome or where genes were situated on the same contig within a draft genome.

### Identification of Gene Homologs

For all the genomes in which one or more of the four CoA biosynthesis pathway enzyme-encoding genes were not annotated, gene homologs were identified using a combination of blastp and psi-blast searches (Altschul and Koonin, 1998). In such cases, the amino acid sequences of annotated gene products were used as query. Identified sequences were deemed homologs if they exhibited an amino acid sequence identity of at least 30% over at least 70% of the query sequence. Identity was further confirmed using predicted protein family, domain, and motif information as well as the genomic location relative to other CoA enzyme-encoding genes.

### Family, Motif, and Domain Analysis of Protein Products

**Figure 2:**
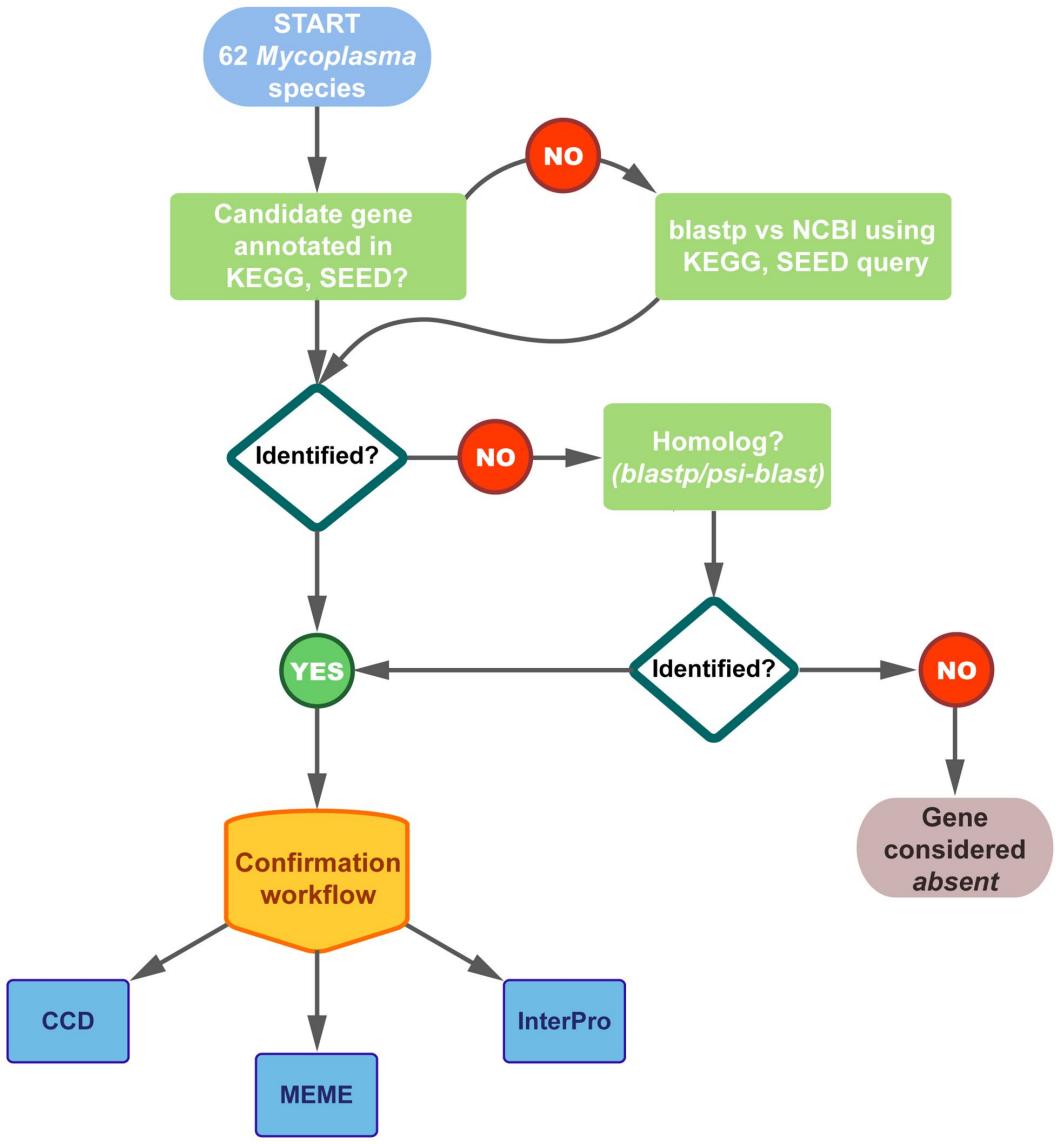
Schematic representation of the workflow followed to identify the genes encoding CoA biosynthesis enzymes in the 62 studied *Mycoplasma* genomes. Genomes were selected from Kyoto Encyclopedia of Genes and Genomes (KEGG), SEED, and National Centre for Biotechnology Information (NCBI) databases. Thirty-seven of the species had annotations for one/more of the pathway genes in KEGG and/or SEED. Results were combined and compared to that on NCBI. For species on NCBI with no corresponding KEGG/SEED information, enzymes were identified through blastp searches. Fifty-four of the species had at least one of the CoA biosynthetic enzyme-encoding genes annotated and the identity of all these genes were confirmed using the indicated databases. Where no genes were annotated, homologs were searched using blastp or psi-blast applying a minimum threshold of 30% amino acid identity over minimum 70% of the query sequence. The confirmation workflow considered family, domain, and motif analyses using the indicated databases; see Materials and Methods for details.

Analysis of annotated and newly identified amino acid sequences of all the pathway enzymes for all the species were performed to confirm existing annotations as well as the identity of homologs as CoA biosynthetic enzymes. The workflow followed is schematically represented in Figure 2. NCBI Conserved Domain Database (CDD) v3.16 was used to predict the family relation and domains of protein products encoded by annotated CoA biosynthesis pathway enzyme-encoding genes as well as identified gene homologs. For additional verification, proteins were also analyzed using InterPro v65.0, which allows for several different member databases to be searched simultaneously (Blum et al., 2021).

Conserved motifs within the protein sequences of each enzyme were determined using the Multiple Expectation maximisation for Motif Elicitation (MEME) algorithm from the MEME suite v4.12.0. (Bailey et al., 2015). The search parameters were set to identify motifs of between six and 50 amino acids in length, with a maximum of four motifs per sequence. The predicted motifs were compared to known functional motifs of the corresponding enzymes.

### Phylogenetic Analyses

For analysis of evolutionary relationships of *Mycoplasma* species, the 16S rRNA gene sequences were obtained from NCBI nucleotide database; the relevant accession numbers are listed in Supplementary Table 2. Included in this analysis were representative species of bacterial sister genera from which mycoplasmas have been shown to have evolved (Woese, 1987). These species, for which the 16S rRNA sequences were obtained from Genbank, are: *Clostridium innocuum* (KR364751.1), *Lactobacillus fermentum* (FJ462686.1), and *Streptococcus pneumoniae* (NR_115239.1). *Bacillus coahuilensis* (EF014447.1) was used as outgroup.

For analysis of the functional relationships of enzymes, their amino acid sequences were obtained from the NCBI protein database; the relevant accession numbers are listed in Supplementary Table 2. Amino acid sequences from the same representative species as before were included where available, and *B. coahuilensis* was again chosen as outgroup (Supplementary Table 3). No PanK_III_ protein was included for *L. fermentum*, as it only possesses a PanK_I_ enzyme. Similarly, no bifunctional CoaBC protein was included for *S. pneumoniae*, as it possesses separate PPCS and PPCDC proteins (Gerdes et al., 2002).

Using Clustal Omega (Sievers and Higgins, 2018) available at https://www.ebi.ac.uk/services, multiple sequence alignments (MSAs) were generated for 16S rRNA genes as well as the protein sequences of individual CoA biosynthesis enzymes. MSAs were manually adjusted in BioEdit v7.0.5.2 (Hall, 1999) to ensure optimal alignment, and where required, the Geneious v11.0.2 (Kearse et al., 2012) program due to its enhanced alignment functionalities. Amino acid sequences were used for the alignments since the nucleotide sequences of the respective enzyme-encoding genes are very heterogeneous and could not be aligned with confidence.

Maximum likelihood phylogenies were constructed using RAxML-HPC2 on XSEDE v8.2.10 (Stamatakis, 2014) *via* the CIPRES Science Gateway v3.3 web portal (Miller et al., 2010). Evaluation of clade support was performed with bootstrap analysis, which was set to automatically stop when the majority rule criterion is reached according to program recommendations. Bootstrap values ≥75% were considered resolved and well supported, values of ≤75% but ≥50% were considered resolved but moderately supported, and values ≤50% were considered poorly supported. Only values of ≥50% are indicated on the phylogenetic trees.

## Results

### Determining the Prevalence of CoA Biosynthetic Enzyme-Encoding Genes Using Existing Functional Annotations

As a result of inconsistent annotation, a combination of database information from KEGG and SEED as well as blastp searches were used to find genes that had functional annotations corresponding to any of the enzymes involved in the CoA biosynthesis pathway (Figure 2). Of the 62 *Mycoplasma* species evaluated, 54 had at least one of the CoA biosynthetic enzyme-encoding genes annotated within their genome (Table 1). For species with pathway information on KEGG and SEED databases, it was found that the information between these databases did not always agree and in some instances also did not match genome information available on NCBI.

**Table 1:** Summary of genes encoding for enzymes of the coenzyme A biosynthesis pathway found within the genomes of evaluated *Mycoplasma* species.

Phylogenetic group^a^	Mycoplasma species	Representative host	CoA biosynthetic pathway enzyme			
			PanK	CoaBC	PPAT	DPCK
Hominis group	*M. anatis* ^*^	Ducks	1	2	3	4
	*M. arginini* ^*^	Mammals	1	2	3	4
	*M. columborale* ^*^	Pigeons	1	2	3	4
	*M. cricetuli* ^*^	Hamsters	1	2	3	4^h^
	*M. mobile* ^	Tench	1	2	3^p^	4
	*M. sturni* ^*^	Songbirds	1	2	3	4^h^
	*M. synoviae* ^	Galliforms	1	2	3^p^	4^h^
	*M. gallinaceum* ^	Galliforms	1^h^	2^h^	3	4^d^
	*M. alligatoris* ^*^	Alligators	1		3	4^h^
	*M. buteonis* ^*^	Raptors	1		3	4^h^
	*M. crocodyli* ^	Crocodiles	1		3	4
	*M. molare* ^*^	Dogs	1		3	4
	*M. pulmonis* ^	Mice	1		3	4
	*M. agalactiae* ^	Small ruminants			3	4
	*M. bovigenitalium* ^	Cattle			3	4
	*M. bovis* ^	Cattle			3	4
	*M. californicum* ^	Cattle			3	4
	*M. canis* ^*^	Dogs			3	4^h^
	*M. collis* ^*^	Rodents			3	4
	*M. columbinum* ^*^	Pigeons			3	4
	*M. felifaucium* ^*^	Pumas			3	4
	*M. felis* ^*^	Cats			3	4
	*M. fermentans* ^	Humans			3	4
	*M. gallinarum* ^*^	Galliforms			3	4
	*M. iners* ^*^	Galliforms			3	4
	*M. leonicaptivi* ^*^	Lions			3	4
Hominis group	*M. lipofaciens* ^*^	Galliforms			3	4
	*M. opalescens* ^*^	Dogs			3	4
	*M. primatum* ^*^	Monkeys			3	4
	*M. simbae* ^*^	Lions			3	4
	*M. conjunctivae* ^	Small ruminants			3	5
	*M. bovoculi* ^	Cattle				5
	*M. dispar* ^	Cattle				5
	*M. flocculare* ^	Pigs				5^h^
	*M. hyopneumoniae* ^	Pigs				5
	*M. ovipneumoniae* ^*^	Sheep				5
	*M. hyorhinis* ^	Pigs				4
	*M.* sp. Ms02^	Ostrich				4
	*M. arthritidis* ^	Rats				
	*M. canadense* ^	Cattle				
	*M. hominis* ^	Humans				
Spiroplasma group	*M. capricolum* subsp. *capricolum*^	Small ruminants			3	4
	*M. capricolum* subsp. *capripneumoniae*^	Goats			3	4
	*M. leachii* ^	Cattle			3	4
	*M. mycoides* subsp. *capri* LC^	Small ruminants			3	4
	*M. mycoides* subsp. *mycoides* SC^	Cattle			3	4
	*M. putrefaciens* ^	Small ruminants			3	4
	*M. yeatsii* ^	Goats			3	4
Pneumoniae group	*M. iowae* ^	Turkeys	1	2s^b^ ^h^	3	4
	*M. testudinis* ^*^	Tortoises	1	2^h^	3	4
	*M. alvi* ^*^	Cattle	1		3	4
	*M. penetrans* ^	Humans	1		3	4
	*M. pirum* ^*^	Humans	1		3	4
	*M. gallisepticum* ^	Galliforms				4
Pneumoniae group	*M. genitalium* ^	Humans				4
	*M. imitans* ^*^	Ducks, geese				4
	*M. pneumoniae* ^	Humans				4
	*M. haemocanis*^ ^c^	Dogs				
	*M. ovis*^ ^c^	Sheep				
	*M. parvum*^ ^c^	Pigs				
	*M. suis*^ ^c^	Pigs				
	*M. wenyonii*^ ^c^	Cattle				

^ Complete genome.

* Genome available as contigs.

a Based on 16S rRNA sequence data.

b Separate PPCS and PPCDC enzymes (no bifunctional protein) with PPCS annotated as “hypothetical protein.”

c Belongs to the hemotropic cluster.

h Annotated as a “hypothetical protein.”

p Annotated as a “putative protein.”

d Disrupted open reading frame.

1 = PanK type II; 2 = CoaBC; 3 = PPAT; 4 = DPCK; and 5 = HAD-DPCK. *M.* species = Mycoplasma species with no pathway enzymes identified.

For those species with PanK-encoding genes, the protein products were indicated as “type III pantothenate kinase.” The common gene annotation used in prokaryotes for PanK_III_-encoding genes is *coaX*, but this annotation was not used in any of the included *Mycoplasma* genomes. Instead, genes were only referred to by their locus tag. There are, however, *Mycoplasma* genomes (not included in this study) in which the *coaX* annotation is used. Although, the PanK EC number (2.7.1.33) is correctly indicated in such instances, the protein product is incorrectly given as a transcriptional regulator due to initial incorrect annotation of this protein (Brand and Strauss, 2005).

Phosphopantothenoylcysteine synthetase and PPCDC activities were associated with a protein encoded by a single gene which in most genomes were annotated as *coaBC*, with the protein product indicated as a “bifunctional phosphopantothenoylcysteine decarboxylase/phosphopantothenate-cysteine ligase (CoaBC).” Within genomes used in this study, *Mycoplasma iowae* was the only exception and contained two different genes encoding for PPCS and PPCDC separately. However, these genes were not annotated as *coaB* and *coaC*, but only contained a locus tag with the protein product of PPCS indicated as a hypothetical protein. Separate genes are also found in all other strains of *M. iowae*, indicating that this separation is not due to a sequencing error in the genome of the strain chosen for this study. Although separate PPCS- and PPCDC-encoding genes were not found in any of the other species investigated in this study, this phenomenon is not unique to *M. iowae*. In the *Mycoplasma fermentans*, NCTC10117 genome (not used in this study) separate PPCS- and PPCDC-encoding genes can also be found and as is the case with all *M. iowae* strains, these genes were consistently separated by four nucleotides. Besides a locus tag as annotation, the separate PPCDC-encoding gene is often incorrectly annotated as *coaBC* with the protein product indicated as “DNA/pantothenate metabolism flavoprotein” (in reference to the gene first being discovered as affecting DNA synthesis; Spitzer and Weiss, 1985). The adjacent PPCS would then simply have a locus tag with the protein product indicated as “phosphopantothenate-cysteine ligase” (a synonym for phosphopantothenoylcysteine synthetase), or as “hypothetical protein.”

The PPAT-encoding genes were annotated in some species as *coaD*, but for most species the gene only contained a locus tag with the protein product indicated as “phosphopantetheine adenylyltransferase.”

The DPCK-encoding gene was the most commonly found in all the species, but with a few exceptions these genes were not annotated as *coaE* and only contained a locus tag. The protein products were, however, indicated as “dephospho-CoA kinase.” Ten species only contained a DPCK-encoding gene in their genomes. Four of these coded for a bifunctional haloacid dehalogenase (HAD)-like/dephospho-CoA kinase (HAD-DPCK) protein. The DPCK-encoding part of this gene was found at the 3′ side of the open reading frame translating into a protein with a DPCK domain at the C-terminal end. *Mycoplasma conjunctivae* was the only species that had a HAD-DPCK-encoding gene in combination with another CoA biosynthesis enzyme-encoding gene, this being for the PPAT enzyme.

In species with both PanK_III_- and CoaBC-encoding genes, the open reading frames always overlapped with 12–24 nucleotides, with the CoaBC-encoding gene found upstream of the PanK_III_-encoding one. This is not a common feature in prokaryotes, as PanK and CoaBC-encoding genes are most commonly either adjacent (e.g., *Clostridium innocuum*) or >100,000 bp apart (e.g., *Lactobacillus* and *Bacillus* sp.). In *Mycoplasma* species with a PanK_III_, but separate PPCS- and PPCDC-encoding genes, there was no consistent distance between the PanK_III_- and the PPCS/PPCDC-encoding genes.

In species containing all four CoA biosynthesis enzyme-encoding genes the distances and orientation of the PanK_III_- and CoaBC- or PPCS/PPCDC-encoding genes, relative to that for PPAT and DPCK, were not consistent. This was also true for species containing the combination of PanK_III_-, PPAT-, and DPCK-encoding genes, with the exception being species in the Spiroplasma group. These displayed a distance in the range of 122–224 kb between the PPAT- and DPCK-encoding gene locations.

In species that contained only PPAT- and DPCK-encoding genes there was no consistent distance or arrangement between these genes. Again, species in the Spiroplasma group were the exception with distances in the range of 122–224 kb between the genes encoding for PPAT and DPCK, respectively. Among the species evaluated only members of the Spiroplasma and Hominis groups had PPAT- and DPCK-encoding gene combinations.

### Identification of CoA Biosynthesis Enzyme-Encoding Gene Homologs

At the time this investigation was initiated (2018), no CoA biosynthesis pathway enzyme-encoding genes or gene homologs could be identified in eight of the 62 *Mycoplasma* genomes evaluated (Table 1; Figure 2). In genomes with no annotated PanK_III_-encoding gene, a homolog was identified in *Mycoplasma gallinaceum* with a protein product of 248 aa in length. This falls within the typical range of annotated PanK_III_ amino acid sequences of 220–275 aa.

In genomes with no annotated CoaBC-encoding gene, homologs were identified in *M. gallinaceum* and *Mycoplasma testudines*. The relative orientation and overlap between the PanK- and CoaBC-encoding gene homologs in *M. gallinaceum* was consistent with that found for annotated genes. However, *M. testudines* had a smaller overlap of only six nucleotides. The average length of the annotated CoaBC sequences was 360–410 aa. The two hypothetical CoaBC proteins are within this range with 385 aa and 380 aa for *M. gallinaceum* and *Mycoplasma testudinis*, respectively.

Furthermore, among genomes with no PPAT-encoding gene, a homolog could be identified in both *Mycoplasma synoviae* and *Mycoplasma mobile*. The typical length of annotated PPAT protein sequences ranges between 135 aa and 175 aa. Both putative PPAT proteins fall within this range with respective protein lengths of 145 aa and 148 aa for *M. mobile* and *M. synoviae*.

Seven species were found to contain a DPCK-encoding gene homolog, of which one was identified to encode for a HAD-DPCK (*Mycoplasma flocculare*). The average length of annotated DPCK sequences is 165–205 aa and the hypothetical DPCK proteins of *Mycoplasma canis* (189 aa), *Mycoplasma cricetuli* (191 aa), *Mycoplasma sturni* (187 aa), *Mycoplasma alligatoris* (185 aa), *Mycoplasma buteonis* (189 aa), and *M. synoviae* (168 aa) all fall within this range. The average length of annotated HAD-DPCK protein sequences is 445 aa, of which the HAD-domain is ~260 aa. The hypothetical HAD-DPCK protein of *M. flocculare* (447 aa) was found to be within this range.

*Mycoplasma gallinaceum* B2096 8B was the only species evaluated in this study that contained no DPCK-encoding gene despite having all three other genes. The KEGG database similarly indicated *M. gallinaceum* (strain not specified) does not contain this gene. A genetically close genome (*M. gallinaceum* NCTC10183) was, however, found to contain an annotated DPCK-encoding gene. The nucleotide sequence of this gene (coded by “LR214950.1:904112.904693”) was therefore used in a blastn search to identify a possible homolog in B2096 8B. A hit with 96% identity and 100% query coverage was found in B2096 8B at the complement position of 322424.323005, which codes for a putative DPCK protein. This region currently contains a hypothetical disrupted protein indicating that the open reading frame was misidentified.

### Family, Motif, and Domain Analysis of CoA Biosynthesis Protein Products

To confirm that genes annotated as hypothetical- or putative proteins were in fact homologs of CoA biosynthesis enzyme-encoding genes, their protein products were evaluated and compared to annotated genes in terms of protein family relationship, as well as domain and motifs contained within the proteins (Figure 2).

#### PanK_III_-Encoding Genes

Except for *Mycoplasma anatis*, the CDD search results (Supplementary Table 4) confirmed the protein product of all the annotated PanK_III_-encoding genes and identified homologs to be of the PanK_III_ type. CDD predicted all of these proteins to belong to the ASKHA superfamily (cl17037), which is the expected assignment for PanK_III_. The InterPro searches (Supplementary Table 5) supported the CDD results as it could predict that all of the proteins, including that of *M. anatis*, belong to Interpro family IPR004619 (“Type III pantothenate kinase”), along with the biological process gene ontology (GO) term, GO:0004594 (pantothenate kinase activity).

Similarities among proteins were further evaluated by identifying conserved motifs using the MEME algorithm. The advantage of using this algorithm is that it does not rely on existing database information for motif discovery, but this does mean that identified motifs may not correspond to a specific function. The four motifs identified in the PanK proteins (Supplementary Table 6; Supplementary Figure 1) all overlapped with PanK_III_-specific domains predicted by CDD and InterPro. Based on active site information from UniProtKB for the PanK_III_ of *Mycoplasma crocodyli* MP145, MEME Motif 1 contains a nucleotide-binding region within which the PHOSPHATE1-motif, attributed to the ASKHA superfamily, can be found (Yang et al., 2006). The MEME Motif 2 contains a conserved amino acid (D) that acts as a proton acceptor and is part of the PAN-motif (*h*G*h*DR, *h* = hydrophobic residue) that is unique to PanK_III_. Amino acids within this motif are involved in binding its substrate, Pan. MEME Motif 2 also contains a conserved amino acid (T) that is involved in ATP binding which forms part of the ASKHA-associated PHOSPHATE2-motif. The PanK_III_-specific INTERFACE-motif is situated in the MEME Motif 3, but the sequence does not exactly match the indicated motif pattern of GGxIxPG (x = any residue). The PAN- and INTERFACE-motifs are said to distinguish PanK_III_s from other ASKHA family proteins.

No MEME Motif 2 or 4 could be identified for the *M. anatis* and *M. buteonis* PanK_III_ enzymes, while the PanK_III_s of several species did not contain a Motif 4. Since MEME identifies ungapped motifs any insertions or deletions in the amino acid sequence will influence the identification of a motif within a specified region. Whether or not a motif was present had no relation to how many of the other pathway enzyme-encoding genes were present in the genome of the species concerned. This may indicate that although CoA biosynthesis-associated genes code for enzymes that function in the same pathway, they are not collectively under the same selection pressure.

#### CoaBC-Encoding Genes

Conserved Domain Database search results (Supplementary Table 7) confirmed the protein product of all annotated CoaBC-encoding genes and identified homologs to be “bi-functional phosphopantothenoylcysteine decarboxylase/phosphopantothenate-cysteine ligase” proteins. The same results were obtained for the separate PPCS and PPCDC proteins of *M. iowae*. CDD predicted all bifunctional proteins to belong to the DNA/pantothenate metabolism flavoprotein (DFP) superfamily (cl27193), as well as the flavoprotein superfamily (cl19190).

The InterPro searches (Supplementary Table 8) identified the InterPro family IPR005252 signature (“Coenzyme A biosynthesis bifunctional protein, CoaBC”) in most of the CoaBC proteins, including the hypothetical protein of *M. gallinaceum*. InterPro also predicted the biological process GO term GO:0015937 (“CoA biosynthetic process”) and molecular function GO terms, GO:0004632 (“PPCS activity”) and GO:0004633 (“PPCDC activity”). Although no InterPro family IPR005252 signature was identified in the CoaBC of *M. mobile* or the hypothetical CoaBC of *M. testudinis*, the InterPro domain signatures, IPR003382 (“Flavoprotein”) and IPR007085 (DFP, C-terminal), were identified and is also present in all of the other protein sequences. These respective InterPro domain signatures were also identified in PPCDC (IPR003382) and PPCS (IPR007085) of *M. iowae*. Except for *M. gallinaceum*, *M. testudinis*, and *M. mobile*, a N-terminal signaling region was predicted for CoaBC proteins, which implies that they are either membrane-bound or situated inside the *Mycoplasma* membrane. Similarly, a “Prokaryotic membrane lipoprotein lipid attachment site” was identified at the N-terminus in the PPCDC of *M. iowae*.

The regions of the MEME identified motifs (Supplementary Table 9) corresponded with the identified signature sequences predicted by CDD and InterPro. Two of the motifs (Motif 1 and Motif 2) were located in the PPCDC domain of the bifunctional protein as well as the separately encoded PPCDC protein of *M. iowae*. Motif 3 and Motif 4, on the other hand, were found in the PPCS domain of the bifunctional protein, but Motif 4 was absent from the separately encoded PPCS protein of *M. iowae*. Moreover, the protein sequences of *M. mobile* and *M. testudinis* were the only two CoaBC proteins which also had no Motif 4 in their PPCS domain (Supplementary Figure 2). Based on information from *C. innocuum*, a possible active site (proton donor) was contained within Motif 2 while all other possible nucleotide binding sites (CTP) were outside of the identified motifs. Changing the MEME parameters to a maximum of six motifs resulted in an additional nucleotide binding site falling within the identified motifs.

#### PPAT-Encoding Genes

In agreement with previous studies (Izard, 2002), the CDD results confirmed all the annotated and putative protein products of the PPAT-encoding genes as “phosphopantetheine adenylyltransferase” (Supplementary Table 10). Additionally, CDD predicted all the proteins to belong to the nucleotidyltransferase superfamily (cl00015). Similar results were obtained from the InterPro searches (Supplementary Table 11), where the family signature, IPR001980 (Phosphopantetheine adenylyltransferase), was identified in all the protein sequences. Furthermore, the InterPro predicted the GO term for the biological process and molecular function as GO:0015937 (CoA biosynthetic process) and GO:0004595 (PPAT activity), respectively. The regions used by CDD and Interpro for predictions in *M. mobile* were noticeably shorter (66% of the total sequence length) which is probably why it is annotated as a putative protein sequence.

Four motifs were identified using MEME (Supplementary Table 12) which overlapped with signature sequences identified by CDD and InterPro. There were segments of these predicted motifs that agreed with the literature-reported motifs (Izard and Geerlof, 1999; Izard, 2002) including prominent residues involved in substrate binding (Supplementary Figure 3). Yet again, the protein sequence of *M. mobile* was the outlier, in which only Motif 1 and 2 could be identified. The absence of Motif 3 and 4 correlates with the shorter recognition region of CDD and InterPro. Also, no Motif 3 could be identified within the protein sequences of *Mycoplasma alvi*, *Mycoplasma penetrans*, *Mycoplasma pirum*, *M. iowae*, and *M. testudinis*. Motif 3 specifically contains a portion of the active site where substrate binding takes place *via* an amide bond.

#### DPCK-Encoding Genes

The identity of all the DPCK protein sequences could be confirmed using CDD searches (Supplementary Table 13). In agreement with previous studies (O’Toole et al., 2003), all of the sequences, including the DPCK domain of the HAD-DPCK, were predicted to belong to both the nucleoside/nucleotide kinase (NK)-(cl17190) and CoaE (cl28605) superfamily. The HAD domain of the HAD-DPCK proteins, including the hypothetical protein of *M. flocculare*, was predicted to belong to the HAD-like (cl21460) and Hydrolase 3 (cl26787) superfamily. The HAD proteins also contain a HAD-like domain predicted to belong to the Cof subfamily, which falls within the Class-IIB subfamily of the HAD-like superfamily. Proteins assigned as Cof proteins are generally cofactor phosphatases (in reference to the activity of the Cof protein of *Escherichia coli*, which acts on intermediates in thiamine biosynthesis) and other HAD family phosphatases (Kuznetsova et al., 2006).

The InterPro family signature, IPR001977 (“Dephospho-CoA kinase”), as well as the homologous superfamily signature, IPR027417 (“P-loop containing nucleoside triphosphate hydrolase”) was identified in all of the annotated protein sequences (Supplementary Table 14). The regions matching these two signatures overlapped in all sequences. The corresponding GO terms predicted were: GO:0015937 (“CoA biosynthetic process”), referring to the biological process; along with GO:0004140 (“DPCK activity”) and GO:0005524 (“ATP binding”), referring to the molecular function. For the eight hypothetical DPCK proteins, only the homologous superfamily signature (IPR027417) could be identified, but given that the region matching to this signature overlaps with that of the family signature (IPR001977) found in the annotated proteins, these sequences were viewed to be related. With regards to the HAD-like domain of the homologous HAD-DPCK protein of *M. flocculare*, InterPro could only identify the family signature, IPR006379 (“HAD-superfamily hydrolase, subfamily IIB”).

**Figure 3:**
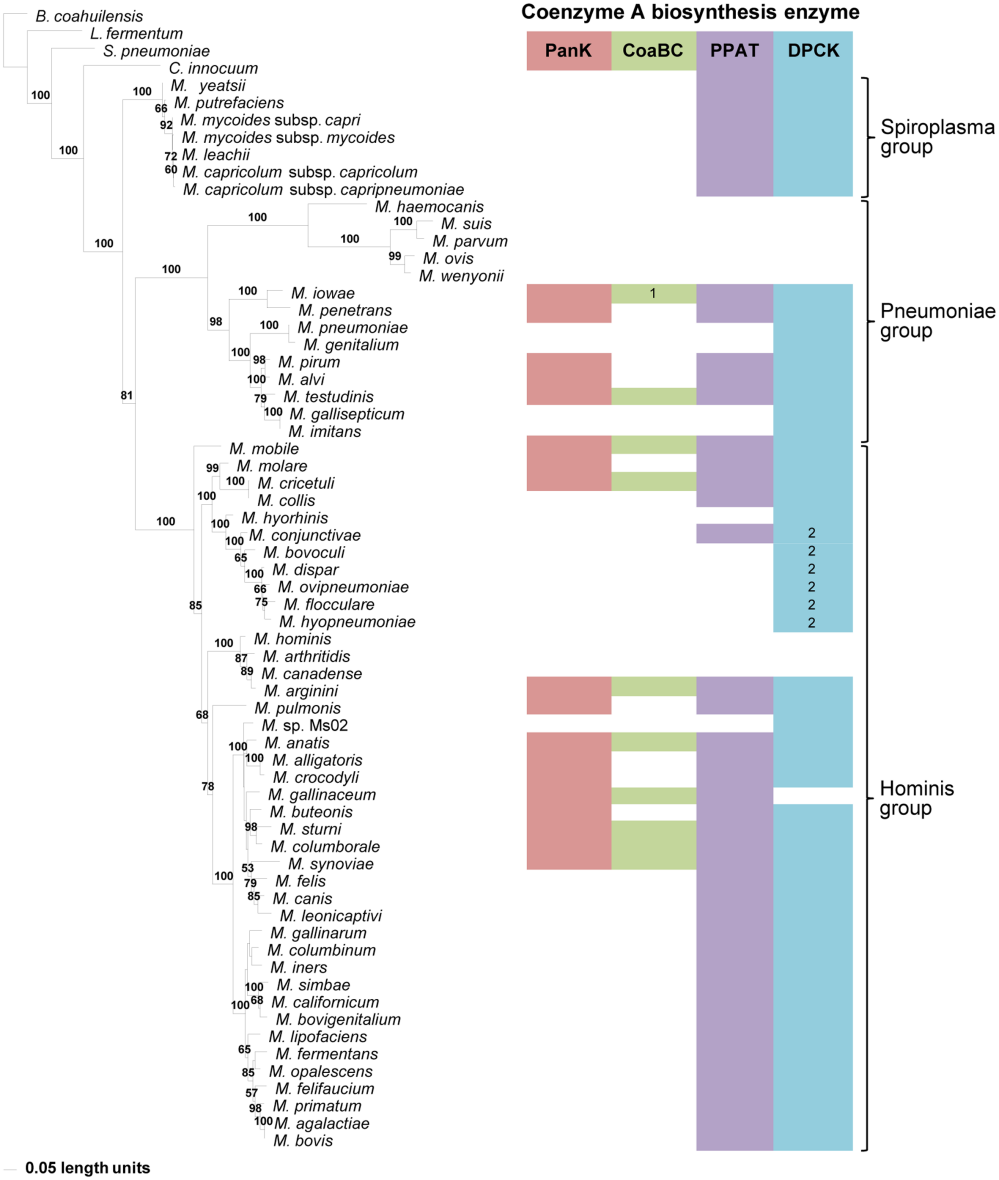
The maximum likelihood phylogeny based on 16S rRNA gene sequences. Bootstrap values (≥50%) are indicated above lines. The identified coenzyme A biosynthetic pathway enzymes of each *Mycoplasma* species are indicated with a color chart: PanK (red), CoaBC (green), PPAT (purple), and DPCK (blue). Notations: 1 (green) – separate PPCS and PPCDC enzymes; 2 (blue) – HAD-DPCK protein.

The four MEME predicted motifs (Supplementary Table 15) in the DPCK protein overlapped with the regions identified by the CDD and InterPro searches. Except for *M. mobile*, all species from the Hominis group (Figure 3; Table 1) contained all four motifs in their DPCK protein. This included the DPCK domain of all HAD-DPCKs. The only exception was *M. mobile*, which, similar to species from the Pneumoniae and Spiroplasma groups, contained no Motif 4. In the case of *M. mobile*, this is due to a single amino acid deletion in the region of Motif 4. Based on information from *M. pneumoniae*, only Motif 1 is associated with a known active site in the form of a nucleotide (ATP) binding site that overlapped with a P-loop/Walker A motif (Walker et al., 1982; Supplementary Figure 4).

### Correlating Evolutionary Relationship With CoA Biosynthesis Through Phylogenetic Analyses

A 16S rRNA gene phylogeny was generated to determine if the evolutionary relationships of *Mycoplasma* species reflect which of the CoA biosynthesis pathway enzyme-encoding genes are found in their genomes. Three well-resolved and supported groups were retrieved in this phylogeny, i.e., the Spiroplasma, Pneumoniae, and Hominis groups (Figure 3). Each group had identifiable and well-supported subclades; however, there was not always consistency between the combination of CoA synthesis pathway genes present in a species and its grouping within a specific clade. An exception to this was the Spiroplasma group which produced a single clade, in which all members only had PPAT and DPCK-encoding genes. Also, species with no CoA biosynthesis enzyme-encoding genes, and those with only a HAD-DPCK-encoding gene, grouped in a single clade in the Pneumoniae- and Hominis group respectively. In addition to this, among the species evaluated, the CoaBC enzyme appeared more frequently among Hominis group members.

Although the lack of one or more enzyme-encoding genes within species with incomplete genomes might influence deductions that can be made, this inconsistent distribution also holds for those with apparent complete genome information. However, when comparing taxa within the Pneumoniae or Hominis clades, evolutionary changes over time (branch lengths) were not reflected by a reduction in the number of CoA synthesis genes.

To determine if the grouping of species relative to the number of CoA synthesis genes in a genome can better be explained at a functional level, a MSA of the respective proteins was used for phylogenetic analysis. Since *M. iowae* contained separate genes encoding for PPCS and PPCDC, two separate alignments were created for the CoaBC enzyme: one without the *M. iowae* sequences, and one including the *M. iowae* PPCS and PPCDC proteins represented as a concatenated sequence.

**Figure 4:**
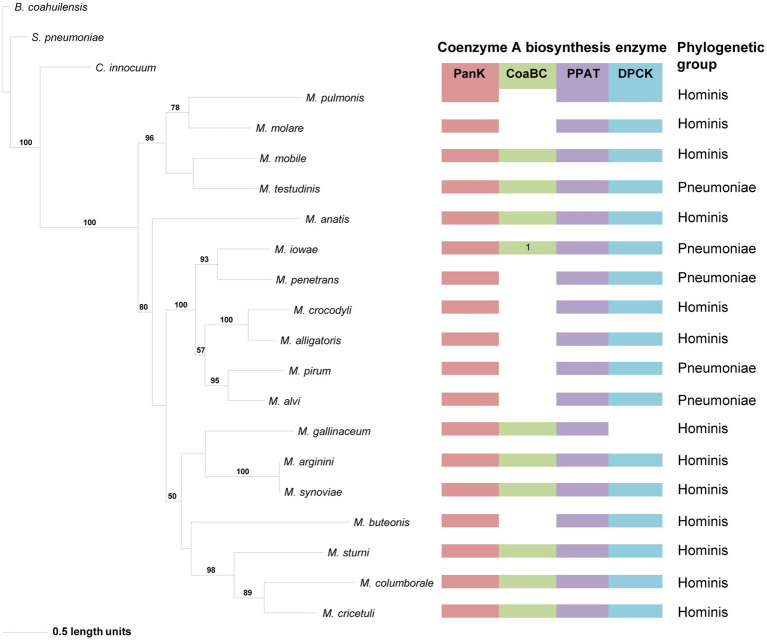
The maximum likelihood phylogeny based on the PanK type III amino acid sequences. Bootstrap values (≥50%) are indicated above lines. Grouping of the species according to the 16S rRNA sequence-based phylogeny, indicated on the right. The identified coenzyme A biosynthetic pathway enzymes of each *Mycoplasma* species are indicated with a color chart: PanK (red), CoaBC (green), PPAT (purple), and DPCK (blue). Notation: 1 (green) –separate PPCS and PPCDC enzymes.

The phylogeny based on the PanK_III_ (Figure 4) revealed well-supported clades. The distribution of species within clades did not always agree with that of the 16S phylogeny, but similar to the 16S phylogeny there was no correlation between the species in a clade and the number of CoA synthesis enzymes present in their genomes. Sequences with a missing MEME Motif 4 (*M. mobile*, *Mycoplasma molare*, *Mycoplasma pulmonis*, and *M. testudinis*) were all located within the same clade, but the corresponding species did not have the same combination of CoA synthesis genes. *Mycoplasma buteonis* and *M. anatis* both have Motif 2 and 4 absent but are found in different clades due to sequence differences outside of the identified motifs.

**Figure 5:**
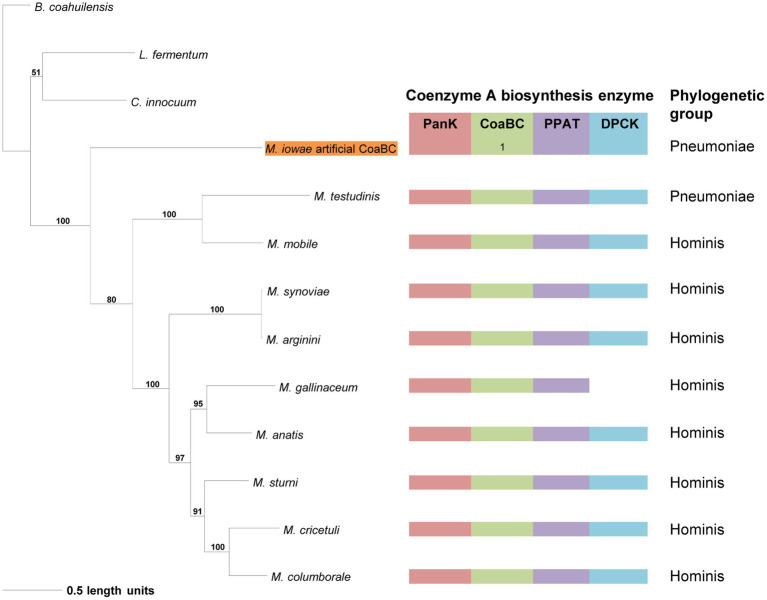
The maximum likelihood phylogeny based on the CoaBC amino acid sequences, including *Mycoplasma iowae*. Bootstrap values (≥50%) are indicated above lines. Grouping of the species according to the 16S rRNA sequence-based phylogeny, indicated on the right. The identified coenzyme A biosynthetic pathway enzymes of each *Mycoplasma* species are indicated with a color chart: PanK (red), CoaBC (green), PPAT (purple), and DPCK (blue). Notation: 1 (green) –separate PPCS and PPCDC enzymes. The orange highlighted species is the concatenated PPCDC and PPCS protein sequence.

The distribution of species in the CoaBC-based phylogeny also did not follow that of the 16S phylogeny (Figure 5). The concatenated sequence of the separate *M. iowae* PPCS and PPCDC protein sequences was retrieved in a basal position and therefore did not influence the tree topology. Except for *M. mobile* and *M. testudinis* that had a missing MEME Motif 4 and therefore grouped in a single clade, there was no apparent reason, beyond sequence similarities, for the distribution of species within this phylogeny.

**Figure 6:**
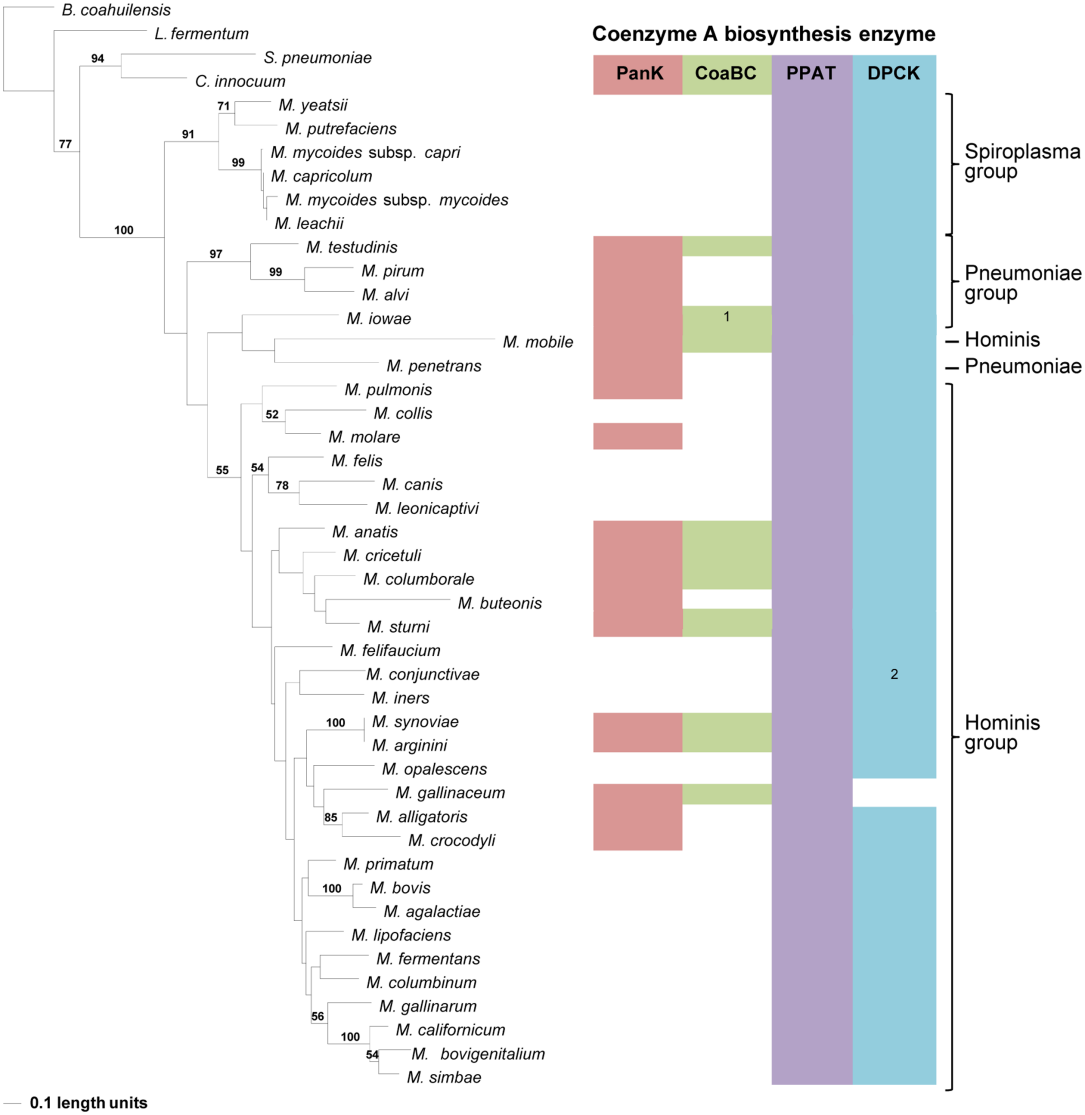
The maximum likelihood phylogeny based on the PPAT amino acid sequences. Bootstrap values (≥50%) are indicated above lines. Suggestive groupings, which resemble that of the 16S rRNA sequence-based phylogeny, are indicated by the brackets (right), with *Mycoplasma mobile* and *Mycoplasma penetans* as exceptions. The identified coenzyme A biosynthetic pathway enzymes of each *Mycoplasma* species are indicated with a color chart: PanK (red), CoaBC (green), PPAT (purple), and DPCK (blue). Notations: 1 (green) – separate PPCS and PPCDC enzymes; 2 (blue) – HAD-DPCK protein.

In the PPAT phylogeny, a distinctive and well-supported clade for the Spiroplasma group could be retrieved similar to that in the 16S phylogeny (Figure 6). For the rest of the phylogeny, Pneumoniae- and Hominis groupings could be observed for most species, but the relationships did not always agree with that of the 16S phylogeny. The distribution of PPAT proteins was also not related to the presence of a PanK and/or CoaBC-encoding genes in the relevant species genome. However, with the exception of *Mycoplasma colis*, the PPAT proteins of species with only PPAT- and DPCK-encoding genes in their genome are always grouped in separate clades. This was also only for members of the Hominis and Spiroplasma groups since no species from the Pneumoniae group had a strictly PPAT-DPCK enzyme combination in their genome. Furthermore, no MEME Motif 3 could be identified in the PPAT sequences of *M. alvi*, *M. iowae*, *M. mobile*, *M. pirum*, *M. penetrans*, and *M. testudinis*, yet these species were grouped in two different clades indicating that phylogenetic distributions were not mainly influenced by motif sequences.

**Figure 7:**
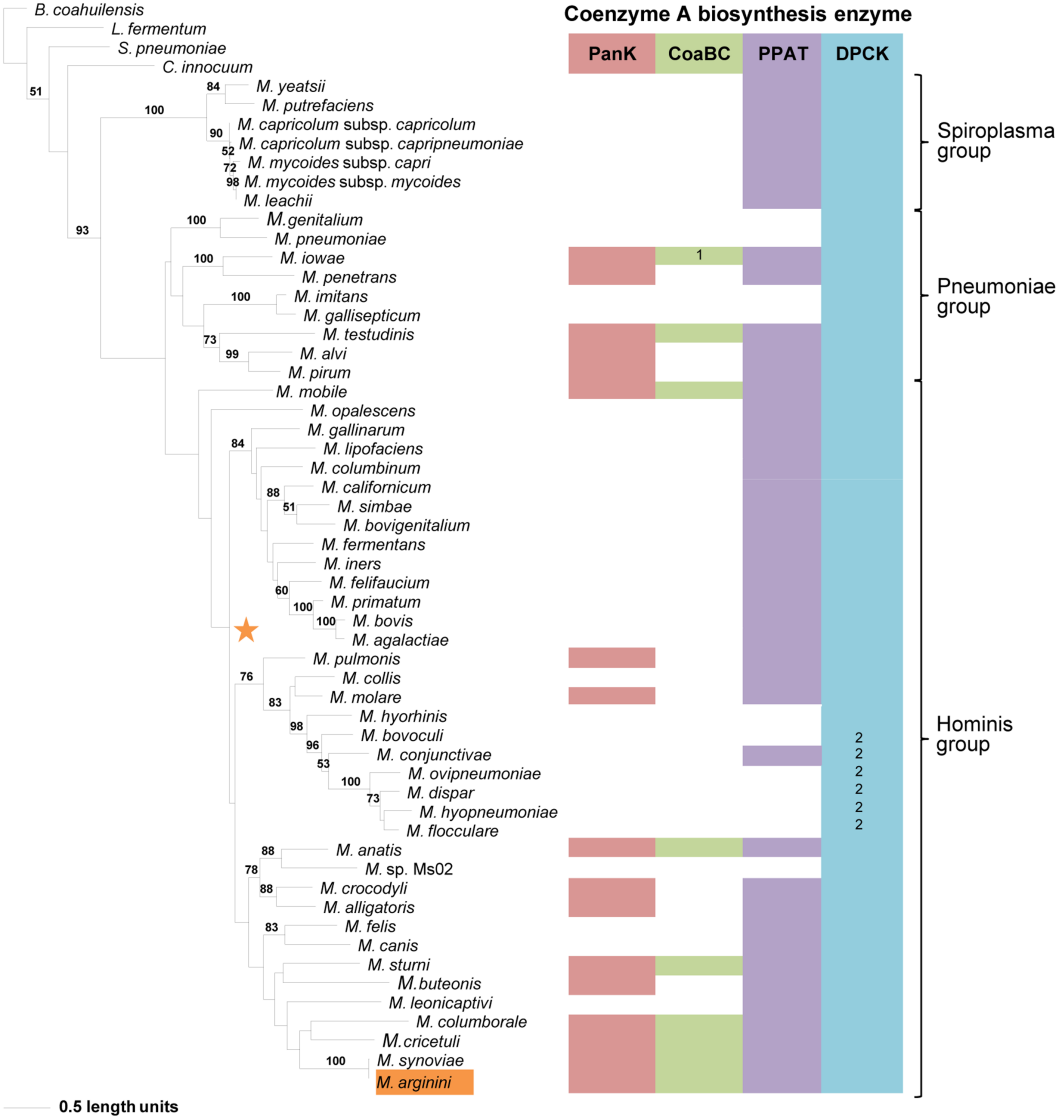
The maximum likelihood phylogeny based on the DPCK amino acid sequences. Bootstrap values (≥50%) are indicated above lines. Suggestive groupings of the three prominent clades, which resemble that of the 16S rRNA sequence-based phylogeny, are indicated by the brackets (right). The identified coenzyme A biosynthetic pathway enzymes of each *Mycoplasma* species are indicated with a color chart: PanK (red), CoaBC (green), PPAT (purple), and DPCK (blue). Notations: 1 (green) –separate PPCS and PPCDC enzymes; 2 (blue) – HAD-DPCK protein. The orange highlighted species is the sub-clade species variation from the 16S rRNA sequence-based phylogeny. The orange star represents the location of *Mycoplasma arginini* in the 16S rRNA sequence-based phylogeny.

Compared to the other enzymes, the DPCK phylogeny was able to retrieve the same Spiroplasma, Pneumoniae, and Hominis groupings as in the 16S phylogeny (Figure 7). Also, except for *Mycoplasma arginini* and *M. cricetuli*, the DPCK proteins of all other species were grouped in the same well-supported clades as in the 16S phylogeny. This included species with a HAD domain as part of the DPCK protein. *Mycoplasma arginini* grouped with species that have no CoA synthesis genes in the 16S phylogeny, which were therefore not present in the DPCK phylogeny. The reason for the different grouping of *M. cricetuli* is not clear. All the species in which the MEME-predicted Motif 4 of DPCK enzymes was absent were from either the Spiroplasma or Pneumoniae group; these are then also found in a single clade in the respective groups.

## Discussion

Our study of CoA biosynthesis in *Mycoplasma* found that their genomes contain genes encoding the CoA pathway enzymes (Figure 1A), but all four genes were not present in every species and different combinations were found across genomes. Among the evaluated genomes available on NCBI, the typical gene annotations of *coaA*/*coaX*, *coaBC* (or *coaB*/*coaC*), *coaD*, and *coaE* were seldom found and therefore were of no use in confirming the presence of these genes within *Mycoplasma* genomes. Information from the KEGG and SEED databases was also limited since not all species – or strains of a species – are represented within these databases.

A description of protein products was, however, more consistent and this allowed the identification of associated genes which were then mostly annotated with a locus tag number. Where no protein products were indicated, enzyme homologs could be identified within several genomes using a combination of NCBI blastp or psi-blast searches using known protein sequences from a genetically close species as query. To further confirm, the identity of the gene its protein product was evaluated in terms of protein family, domain, and motif information using the web-based CDD, InterPro, and MEME Suite recourses.

Of the 62 *Mycoplasma* species evaluated in this study, 10 contained all four enzyme-encoding genes of the CoA synthesis pathway in their genomes. These 10 species were only found in the Hominis and Pneumoniae groups in the 16S phylogeny. Except for *M. iowae*, which contained separate PPCS- and PPCDC-encoding genes, the rest all had a bi-functional CoaBC-encoding gene which is more typical of prokaryotes. Although all strains of *M. iowae* contain separate genes, differences among strains were found in *M. fermentans*. The open reading frame of all CoaBC-encoding genes always overlapped with that of PanK, which could imply that the genes are transcribed as a single operon and are therefore under the same selective pressure. However, where the PPCS- and PPCDC encoding genes are separated, the PanK-encoding gene is found thousands of base pairs upstream, but at no fixed distance and transcription as a single operon can therefore be excluded.

Except for *M. sturni*, the cellular localization of the PanK enzymes could not be predicted using InterPro, but prokaryotic PanKs are typically intracellular proteins. However, the PanK of *M. sturni* was predicted to be a transmembrane protein (Supplementary Table 5) with the extracellular domain containing the PAN- and INTERFACE-motifs that are specifically involved in interaction with the PanK_III_ substrate, Pan (Yang et al., 2006). It could therefore bind extracellular Pan and convert it to 4′-phosphopantothenic acid (P-Pan) for further processing by CoaBC, which was also predicted to be an extracellular, membrane-bound protein. However, if the PanK enzymes in other *Mycoplasma* species are in fact situated in the cytoplasm, then the product of PanK, P-Pan, will have to be exported before it can be used as a substrate by CoaBC. Unassisted export of this substrate seems highly unlikely since it is very polar (consisting of a phosphate group and a carboxylic acid moiety) and would thus require a dedicated transporter to cross the membrane. Alternatively, P-Pan might be obtained from the host if readily available in the environment. If this is possible, then the PanK enzyme might not even be required for CoA synthesis in these organisms, but have an entirely different role. How and where the subsequent processing of the CoaBC product, 4′-phosphopantetheine (PPantSH), will take place is not clear since the cellular localization of the *Mycoplasma* PPAT could not be predicted. Prokaryotic PPAT enzymes are typically located in the cytoplasm and if this is also the case for *Mycoplasma* then PPantSH would have to be imported after being released from the CoaBC enzyme.

**Figure 8:**
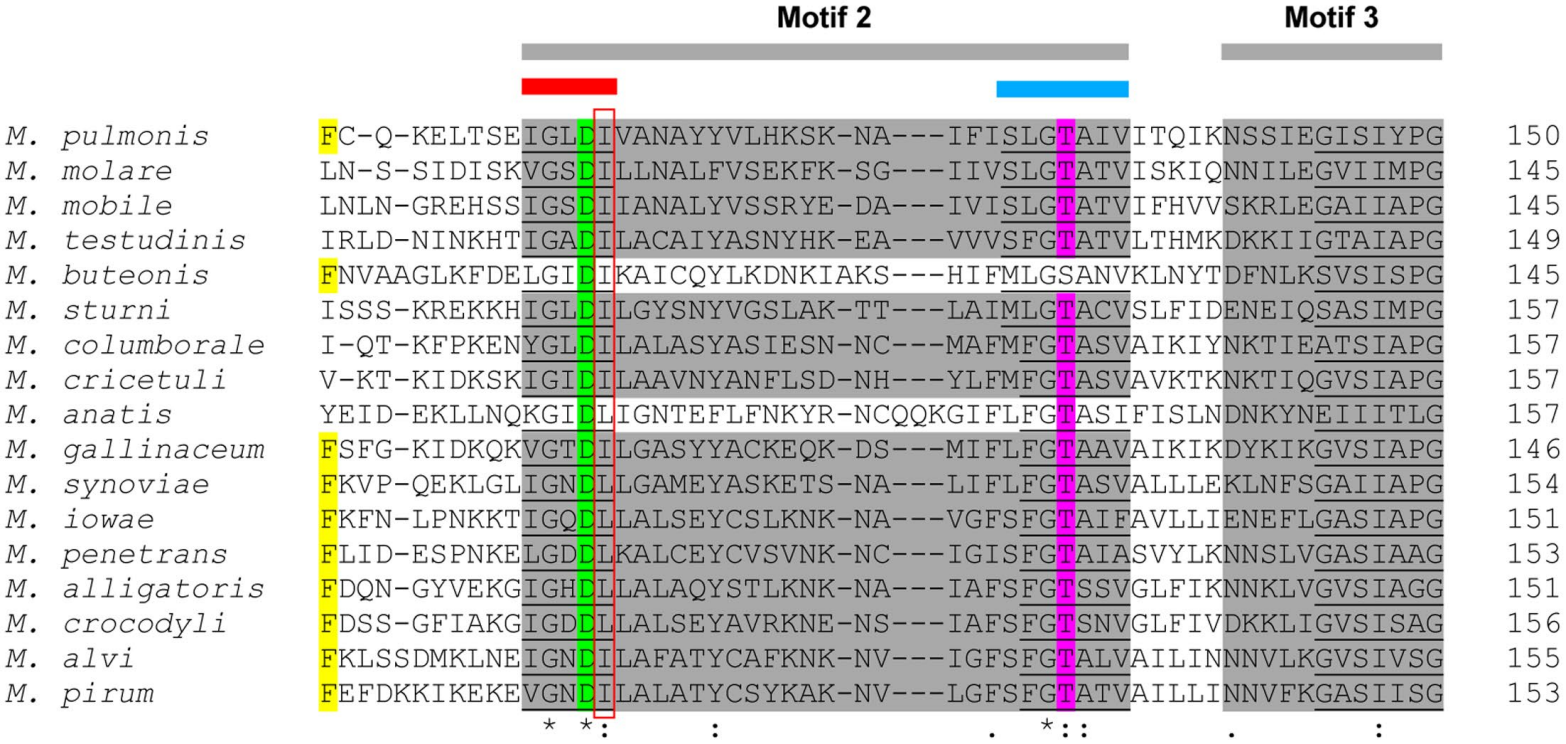
A section of the multiple sequence alignment (MSA) of the PanK_III_ amino acid sequences. The Multiple Expectation maximisation for Motif Elicitation (MEME) motifs are highlighted in gray. The PAN-motif (red bar) and INTERFACE-motif (blue bar) are both situated in MEME-predicted Motif 2. Insertions are indicated by dashes (-). The *Mycoplasma* PAN-motif mutation site (R substituted by L or I) is indicated by the red box. Yellow, green, and magenta highlighted residues represent conserved amino acids that are involved in substrate interactions.

In eight of the evaluated species, a CoaBC-encoding gene was absent with only PanK-, PPAT-, and DPCK-encoding genes present. In the 16S phylogeny these species were only found in the Pneumoniae and Hominis groups, but none in the Spiroplasma group. This combination of enzymes would seem to indicate that CoA biosynthesis occurs *via* the salvage pathway (Figure 1B). However, *Mycoplasma* PanKs are all predicted to be PanK_III_ type enzymes, none of which are known to accept PantSH – the substrate of the salvage pathway – as a substrate (Strauss, 2010). When comparing the PAN-motif (*h*G*h*DR) in *Mycoplasma* PanK_III_ enzymes to that described for PanK_III_s from other bacteria, the arginine residue is replaced by leucine or isoleucine in *Mycoplasma* (Figure 8). This arginine residue plays a prominent role in the active site of the PanK enzyme, since it is directly involved in an interaction with the substrate, Pan. Therefore, the substitution of this charged residue with an uncharged hydrophobic residue will most likely have major consequences with regards to the binding of Pan. The INTERFACE-motif in *Mycoplasma* similarly deviates from the general residue sequence. Since these two motifs together form a substrate-binding site in PanK_III_s, the substitution of residues in both motifs may allow the enzymes to use PantSH as a substrate so that CoA biosynthesis could take place *via* the salvage pathway. It is also possible that the substitutions in these motifs have no impact on PanK substrate specificity, and that like other PanK_III_s these enzymes are also not able to phosphorylate PantSH. They might thus have a role unrelated to CoA synthesis as suggested previously, with these species only relying on the PPAT and DPCK proteins to obtain CoA. Unfortunately, our attempts to date to experimentally evaluate the substrate specificity of the *Mycoplasma* PanKs were unsuccessful due to difficulties in obtaining soluble protein for use in activity assays.

The majority of *Mycoplasma* species evaluated in this study only contained genes encoding for PPAT and DPCK. These species were all situated in either the Hominis or Spiroplasma groups, but none in the Pneumoniae group. If these genes do produce functional proteins, PPAT would have to obtain its PPantSH substrate in the absence of PanK. Recently, it has become evident that certain cells, including some micro-organisms, might be able to obtain PPantSH from their surroundings to fulfil their CoA needs (Sibon and Strauss, 2016). Also, in *Drosophila melanogaster* Schneider 2 cells, PPantSH is able to cross the membrane without the help of a transporter (Srinivasan et al., 2015). How *Mycoplasma* would obtain PPantSH remains an open question. However, all indications are that PPantSH is readily available in the environment. In addition to being an intermediate product of the *de novo* CoA biosynthetic pathway (required by the host), PPantSH can also be produced *via* the degradation of CoA and the acyl carrier proteins involved in lipid biosynthesis (Jackowski and Rock, 1984b). Furthermore, *E. coli* was found to excrete PPantSH, but also never re-imported it (Jackowski and Rock, 1984a). Thus, PPantSH supplied by the host or surrounding microbial communities would allow *Mycoplasma* to indeed salvage PPantSH from the environment for conversion to CoA by the action of PPAT and DPCK (Figure 1B).

The most common enzyme-encoding gene that could be identified among the investigated mycoplasmas was that for DPCK. With some exceptions, this gene was present in all of the mycoplasmas. Eleven of the species contained only a DPCK-enzyme encoding gene and these species were distributed over the Hominis and Pneumoniae groups. Five of these were HAD-DPCK-encoding genes and these were only found among the Hominis group members. *Mycoplasma conjunctivae* was the only species to contain a HAD-DPCK-encoding gene in combination with another CoA-synthesis pathway gene (PPAT).

The fact that only the final enzyme in the CoA synthesis pathway could be identified for some species suggests that the DPCK enzymes scavenge their substrate – dephospho-coenzyme A (dPCoA) – either from the host or the surrounding environment (Figure 1B). The DPCK enzymes of *Mycoplasma gallisepticum* and *Mycoplasma imitans* were both predicted to contain signal peptides and be located extracellularly, which would allow easy access to dPCoA from the host or environment. The cellular localization of the rest could not be predicted, but given that DPCK enzymes are typically found intracellularly, this would require the *Mycoplasma* to have a (as of yet unidentified) dPCoA transport system. The presence of only DPCK and their concomitant requirement for the uptake of dPCoA has also been predicted for various *Rickettsia* and *Chlamydia* spp. (Strauss, 2010; Driscoll et al., 2017). Furthermore, the characterization of a mitochondrial transporter of dPCoA in *D. melanogaster* suggests that a prokaryotic version of such a transporter might exist (Vozza et al., 2017). The obvious dependency of mycoplasmas on their host for many metabolites and intermediates – due to their restricted metabolic biosynthetic potential – highlights the necessity for a vast set of transporters. However, while many *Mycoplasma* transporters are vaguely annotated based on similarities to established transporter classes or membrane segments, little is known regarding their actual substrate preferences or specificities (Großhennig et al., 2013).

The role of the HAD domain in the bifunctional HAD-DPCK enzyme is not clear. As indicated before, the HAD protein is predicted to belong to the Cof subfamily of proteins which consists of phosphatases. These types of phosphatases are promiscuous and seem to be associated with numerous vitamin and cofactor metabolic processes (Lawhorn et al., 2004; Kuznetsova et al., 2006). While the phosphatase activity might suggest a role in regulation (by removing phosphates required for biosynthesis) this has not yet been experimentally tested.

Only eight *Mycoplasma* species with no CoA biosynthesis enzyme-encoding genes could be identified. Five of these form part of the hemotropic *Mycoplasma* cluster (Table 1; Brown, 2010), which is a division within the Pneumoniae group while the rest were from the Hominis group. The mycoplasmas in the hemotropic cluster are also referred to as hemoplasmas and are blood-borne pathogens that infect the erythrocyte (Messick, 2004). The absence of genes in the eight species was confirmed by a comparative genomics study that showed them to not contain any enzymes involved in CoA metabolism, the pentose-phosphate pathway or pyruvate dehydrogenase complex (Guimaraes et al., 2014). In the case of the hemoplasma, the nutrient-rich environment of the blood provides the ideal conditions for additional degenerative evolution, and thereby metabolic reduction. However, it is interesting to note that the blood-borne *Plasmodium* parasite that causes malaria also infects erythrocytes, and yet CoA biosynthesis is the only vitamin-requiring pathway that is essential in this organism (Spry et al., 2008). Clearly, the need for CoA depends on the organism and its metabolic requirements, and for many pathogens, this remains largely unexplored.

The phylogeny produced using 16S rRNA gene sequences reflected the evolutionary relationships of the investigated mycoplasmas as reported in the literature (Weisburg et al., 1989; Johansson et al., 1998; Maniloff, 2002; Brown, 2010; Wium et al., 2015). Except for the Spiroplasma group, where all species only contained genes encoding for PPAT and DPCK, the evolutionary relationships of species were not found to correlate with the combination of CoA synthesis genes contained within their genomes.

Apart from the DPCK phylogeny, the distribution of species in the phylogeny of individual enzymes did not resemble the evolutionary relationships found in the 16S rRNA phylogeny. Moreover, the functional relationship of one enzyme in the CoA synthesis pathway also did not determine which of the other enzymes would be present in a species. However, members of the Spiroplasma group did produce distinctive clades in both the PPAT and DPCK phylogenies. With a few exceptions, this was also the case for members of the Hominis group with only PPAT and DPCK proteins.

From our results, it can be concluded that most *Mycoplasma* species do have the genetic capacity to synthesize CoA, but there is an unpredictability according to the universally accepted 16S rRNA phylogeny among species in terms of which enzyme-encoding gene combinations can be expected, and therefore what the required substrate for CoA synthesis would be in each case. This variation may be entirely dependent on the nutritional conditions at the site of infection. Their parasitic lifestyle on the one hand, and their nutritionally exacting nature on the other, typically causes *Mycoplasma* to exhibit a rather strict host, organ, and tissue specificity (Razin, 2006). They are therefore reliant on the nutritional milieu provided by the host or the microbial community at the site of infection, which would in turn influence their biosynthetic capacity or requirements. We note, for example, that those species with the most complete pathways mainly have non-mammalian hosts. It is possible that in these hosts the ability to obtain CoA *via* truncated pathways is limited due to low levels of circulating CoA biosynthesis intermediates. This observation suggests that instead of correlating with evolutionary relationships, the combination of CoA synthesis genes is more likely to correlate with the mycoplasmas’ immediate nutritional environment. However, testing such a proposal will only be possible once more information becomes available on the levels of circulating metabolites in the various hosts.

Despite the variation in the CoA synthesis capacity of *Mycoplasma*, there is the potential for the CoA biosynthetic pathway as a drug/vaccine target. A comprehensive investigation into the CoA synthesis pathway of the targeted *Mycoplasma* will, however, have to be performed in order to identify the genetic variation present within the genome of the specific species (or within strains of the same species, as these might also show differences). The most consistently present enzyme-encoding gene was that of DPCK, suggesting that the development of a broad-spectrum anti-mycoplasmal agent acting on CoA biosynthesis should target this enzyme. The potential of DPCK as a selective target is further underscored by the fact that the main DPCK activity of the human host is found on a bifunctional PPAT-DPCK protein called CoA synthase (COASY), indicating that selective targeting may be possible. On the other hand, for those species in which no CoA biosynthesis enzyme-encoding genes could be identified, the likely requirement for a specific CoA transporter also offers an opportunity for the development of new therapies that would target this protein and/or its function. Overall, our findings suggest that CoA biosynthesis in *Mycoplasma* holds many significant opportunities for further study and investigation, specifically in the search for new treatments of *Mycoplasma* infections.

## Data Availability Statement

The original contributions presented in the study are included in the article/Supplementary Material, further inquiries can be directed to the corresponding author.

## Author Contributions

AB and ES contributed to conception and design of the study. TR performed all experimental work and analysis and wrote first draft of the manuscript. AB supervised the project with the assistance of ES and revised the manuscript for submission with support from ES. All authors contributed to the article and approved the submitted version.

## Funding

This research received no specific grant from any funding agency in the public, commercial, or not-for-profit sectors. Postgraduate student funding was received from the National Research Foundation and the Faculty of Science, Stellenbosch University.

## Conflict of Interest

The authors declare that the research was conducted in the absence of any commercial or financial relationships that could be construed as a potential conflict of interest.

## Publisher’s Note

All claims expressed in this article are solely those of the authors and do not necessarily represent those of their affiliated organizations, or those of the publisher, the editors and the reviewers. Any product that may be evaluated in this article, or claim that may be made by its manufacturer, is not guaranteed or endorsed by the publisher.
