# Supplementary material for: Evaluating the Genetic Capacity of Mycoplasmas for Coenzyme A Biosynthesis in a Search for New Anti-mycoplasma Targets
Source: Front Microbiol. 2021 Dec 20;12:791756. doi: 10.3389/fmicb.2021.791756 (PMC8721197; doi:10.3389/fmicb.2021.791756)
Supplement: Supplementary file 1 [file Data_Sheet_1.PDF]

Supplementary Figures

Evaluating the genetic capacity of Mycoplasmas for coenzyme A biosynthesis in a search for new anti-mycoplasma targets

Tertius Alwyn Ras[1], Erick Strauss[1], Annelise Botes[1*]

[1]Department of Biochemistry, Stellenbosch University, Stellenbosch, South Africa.

Supplementary Figure 1

PanK - CLUSTAL O(1.2.4) multiple sequence alignment
Use file: clustalomega-I20161206-094932-0767-86989524-pg_woClostridium.fasta

MEME Motifs highlighted in grey.
Regions with possible activity based on UniProtKB information for *M. crocodyli* MP145 (D5E4L7): blue=nucleotide binding site (DIGNSFIK); yellow=substrate binding sites (F and T); green=proton acceptor (D); magenta=ATP binding site (T); underlined sequences represent Pan-motif unique to $PanK_{III}$.
Red blocks: MEME motifs – parameters set to max 8 motifs

```
                                          Motif 1/Motif 1      Motif 2

M_pulmonis        ------------MKIFIDLGNSFAKFALKNENKAHF-LFRLKTQSVVDPSFEIKSF----     43
M_molare          -------------MLFLDLGNSLLKIGYYNKNKKLL--IKKIKSHNLNKEKIINEI----     41
M_mobile          ------------MNLYLDIGNTNLKFGYEIEN--QFHFYTLPTLENYTCDMLSKNL----     42
M_testudinis      ----------MIKNLYIDIGNTFTKFGIYDQIQKTWHICKLRTSEYETSSTVYVFL----     46
M_buteonis        ----------MKQTIVLDIGNTTIDVAFFNENTLYK-TAKFYHEDIEQE--FLYAI----     43
M_sturni          ----------MQEIAVFDIGNTNIKLSAYKVKTLEEYQIILSTTVVGPTEKLLTDPDLIR     50
M_columborale     ----------MNRLVIIDAGNTNIKGTHVKLGYFD--MDAIEEEIICSSQLLIDDLQKAV     48
M_cricetuli       ----------MKKIIIFDIGNTNIKCFLVSLINSN--FLIEKESIISKSNQILKDPQNVI     48
M_anatis          -----MK--SNNIYMVIDIGNTNCKINVMDYETNVINNTKIEKHNKYDIE-TIMNN----     48
M_gallinaceum     ---------MKRKTIVYDVGNTYVKCGLFENK-KLIKVEQIHTESFNDD--SVAHL----     44
M_synoviae        -----MKNNQSKIKLVIDVGNSYLKIGVFEDL-ELIELKKFKTKYFKIS--YFENF----     48
M_iowae           ---------MVSNIIVVDIGNSYIKIAIVNSNLNVLNKLMFKTTEKISK-RFISKK----     46
M_penetrans       -----MK-NNQEYILVVDIGNSYTKIGIFEKEKENTTSIILFPTDSETN-IATLSK----     49
M_alligatoris     -----MK-KNNEKFLTIDIGNSYIKMAVFGQNVELLEYSVFPTKLQSIKQIT---K----     47
M_crocodyli       MISKNFK-TNIKSYLTIDIGNSFIKMGLFNENKELIDFSMFPSKKIDLDKII---S----     52
M_alvi            -----MK-KKEKNVLVIDIGNSYTKFALFNINDEIFFSFSYPTKIISTKNNFLFQI----     50
M_pirum           -----MN-SLLSNVLVIDVGNSYTKIAILNDKKKILKKITLLTKNIKS-TNYVKKI----     49
                                   * **:  .

                         Motif 2                                 Motif 3
M_pulmonis        -----NLF-EFNKLDVKEILICSVRNAKENQILEFKLKSIFKNAKI-DFFIHKS-QSLVK     95
M_molare          ---------LNENIQFKKALLSSVKPKLNKLIIESLNEIGIKTTII-KNDFIIN-NYSLK     90
M_mobile          -----E-S-FIKKQKFNYLIVSSVVPSLNLVIKDFASIH----LKS-KVMFIDK-IKKEI     89
M_testudinis      -----K-N-NLETLKFDYLIISSVVPYMDIVMKELNSQY----LKV-KMIFLNG-DSDLP     93
M_buteonis        ---------ASEISSNSKLVVGYVNKTAFNLVYKYLKNF----FSS--IQIIND-YTKWD     87
M_sturni          EKIKEIIG-DK-LLNNFVFLVGSSIHSLSVILSDILKEL----QVA-Y--KIIDHDYNFK     101
M_columborale     KAFDKWMK-NNNLGDKDHFVIMSPSKLYENKIEDILIYN----FGI-KSGFSMSHDDFRY     102
M_cricetuli       NQFVQIAS-KWDFDSKMQFYIASPSREYVVQWMKLLKKH----FKN-YKINIIDSSYPFN     102
M_anatis          -----LIE-LIKKYNTKTILIGSVVLNIAEKIIKKLKIQ----FIGIDIYNMDSLRKFIS     98
M_gallinaceum     -----NSV-F-EILPEDNVVYGSVVKRVSKILKDFYSTD----FKN--LFEINS-SLKFN     90
M_synoviae        -----YKKVFAKYKFNLFIVFGSVVPSIEQKFLDFVKEN----NLENNFFLINN-YLKLS     98
M_iowae           -----LLS-LSENINIDGAIIGSVVSHITNKFFCLIKKI----FNV-TPFLIDQENVKVS     95
M_penetrans       -----KMN-VFKKYNIKHSIVGSVVPKLKPTYFKTIKKM----FNI-EPYYISE-TTKYS     97
M_alligatoris     -----TIE-SYKKLEIKQAILGSVVKDFDLIIQSIIKEN----LKI-IPYKINQ-QTKFN     95
M_crocodyli       -----KLE-TYKMKNIVSVIMGCVVKSYWEKLNLVLKNV----LSI-EAYRINE-KTKFS     100
M_alvi            -----LKQ-KLKSINIDSIILGSVVPKLNSFFIKTFKEK----LLL-TPHVINK-KTKFI     98
M_pirum           -----IKT-YFNNKHLRYGILGSVVLDKNLFFINAFEKN----E-L-KLYLINK-KTKFN     96

                                        Motif 2/Motif 4             Motif 3…/Motif 5…

M_pulmonis        FC-Q-KELTSEIGLDIVANAYYVLHKSK-NA---IFISLGTAIVITQIKNSSIEGISIYP     149 H-P-TD
M_molare          LN-S-SIDISKVGSDILLNALFVSEKFK-SG---IIVSLGTATVISKIQNNILEGVIIMP     144 H-P-TD
M_mobile          LNLN-GREHSSIGSDIIANALYVSSRYE-DA---IVISLGTATVIFHVVSKRLEGAIIAP     144 H-PCTD
M_testudinis      IRLD-NINKHTIGADILACAIYASNYHK-EA---VVVSFGTATVLTHMKDKKIIGTAIAP     148 P-PCTD
M_buteonis        FNVAAGLKFDELGIDIKAICQYLKDNKIAKS---HIFMLGSANVKLNYTDFNLKSVSISP     144 H-P-TD
M_sturni          ISSS-KREKKHIGLDILGYSNYVGSLAK-TT---LAIMLGTACVSLFIDENEIQSASIMP     156
M_columborale     I-QT-KFPKENYGLDILALASYASIESN-NC---MAFMFGTASVAIKIYNKTIEATSIAP     156
M_cricetuli       V-KT-KIDKSKIGIDILAAVNYANFLSD-NH---YLFMFGTASVAVKTKNKTIQGVSIAP     156
M_anatis          YEID-EKLLNQKGIDLIGNTEFLFNKYR-NCQQKGIFLFGTASIFISLNDNKYNEIIITL     156 H-PCTD
M_gallinaceum     FSFG-KIDKQKVGTDILGASYYACKEQK-DS---MIFLFGTAAVAIKIKDYKIKGVSIAP     145
M_synoviae        FKVP-QEKLGLIGNDLLGAMEYASKETS-NA---LIFLFGTASVALLLEKLNFSGAIIAP     153
M_iowae           FKFN-LPNKKTIGQDLLALSEYCSLKNK-NA---VGFSFGTAIFAVLLIENEFLGASIAP     150
M_penetrans       FLID-ESPNKELGDDLKALCEYCVSVNK-NC---IGISFGTAIASVYLKNNSLVGASIAA     152
M_alligatoris     FDQN-GYVEKGIGHDLLALAQYSTLKNK-NA---IAFSFGTSSVGLFIKNNKLVGVSIAG     150
M_crocodyli       FDSS-GFIAKGIGDDLLALSEYAVRKNE-NS---IAFSFGTSNVGLFIVDKKLIGVSISA     155
M_alvi            FKLSSDMKLNEIGNDILAFATYCAFKNK-NV---IGFSFGTALVAILINNNVLKGVSIVS     154
M_pirum           FEFDKKIKEKEVGNDILALATYCSYKAK-NV---LGFSFGTATVAILLINNVFKGASIIS     152
                              * *:     :              .  *::       .       :

                    …Motif 3/…Motif 5                  Motif 4…/Motif 6

M_pulmonis        GIYQSFKNFFNVV----AKIESNFNIIKIP-----PILGKTTLESISS------SLVRGS     194
M_molare          GIETNLISLFSSA----SKIKKISLNYDKT-----LKLGTNTKDAISI------GILKGH     189
M_mobile          GVKNSYLSLIQSA----KKLSEVILKL-PR-----KNLGGNTQEAISL------GILKGN     188
M_testudinis      GVYSSYLNLVSKA----AKLSNTPLKK-VK-----STSATNTQEALSI------GFINGF     192
M_buteonis        GLELQFNALKDYI----YAFNSINFNDISQLITDELSSGKNTKEAIAL----------GL     190
M_sturni          GISLSFEALLQRLSKSKSGNQNWKYQIMQS-----LEYGYSTSSALNF----------GY     201
M_columborale     GIGLSFDALQNKLKKSSSGNREWNYRVDYS-----NKMGTNTQEALNS----------GA     201
M_cricetuli       GVSFSFQQLQQFLQKSKSGLKKWKYSIKPN-----QKLGNNTNEALNS----------GF     201
M_anatis          GIVRTIVNLIKDA----SILRKFYYKSIS---------KMSNQEIIEFTKVNIPNVYKGA     203
M_gallinaceum     GIGFAFNKLLEQA----DGLKHIQFSYTKQ-----IAMGTNTIDALKM----------GF     186
M_synoviae        GMNFSFNNLLSKA----KKLKGFKLHKENV-----SLWNLNTQDALES----------GY     194
M_iowae           GIGTSFDEFINRV----HMIDVINLNKKEL-----TFFGDNTIKALES----------GV     191
M_penetrans       GLGFGLNKLIEKA----SLLKKSKIDKFSS-----DFFGTNTISALES----------GI     193
M_alligatoris     GLDSSYETLVKKA----SLLNKIEIDKNSM-----LPYGTDNKECLES----------GY     191
M_crocodyli       GLESSYNSLISKA----SLLKKTKIDRASL-----LSYGQDTSGALES----------GY     196
M_alvi            GLKTSLDALVDKT----ALINIKNLELSKN-----FDYGHDTLTALNS----------GI     195
M_pirum           GLKINVESLFSRT----YLINKIQFDFRKK-----NLLGNDTRTAIES----------GL     193
                   :        .                              .   :            *
```

```
                          …Motif 4                     Motif 7                 Motif 8
M_pulmonis      VFLLKGYIDEI---------DK--TSDIFITGGTITFSKIF------------------     224
M_molare        YYSIKSLIKEN---------NE-ENLPIFYTGGNIKYLKSF-----------IKEKMINE     228
M_mobile        FHLINGFINEL---------DPKGKSKILITGGNYGMLKDVL----------KDYEYVDN     229
M_testudinis    KHLACGLAKAM---------SP--NAKLLVTGGDASLIKDI-----------ENAEYIEN     230
M_buteonis      KNSLIELVL------------PYNSDDILITGCNLKYLPIII--------KNNPNKYDYN     230
M_sturni        TNMILGIILGSYFALKNE--DRKKIQTIYLSGMDIQNFEIILSLFKKATDSKVEIIYDAN     259
M_columborale   YNMFLGFIITHIFSNIEI--IGRGTLDVYITGGDLQNEELIKK-DLQNFFKSVNFHVGSS     258
M_cricetuli     YHQISGFIISHLLKDKIF-LMMNKKVKIILTGGDFSKMDSLTI-FLRELFPKNEFIYVNN     259
M_anatis        LVSANGFINECHNTYLDN----NNSSNYLISGGDANLID-----------PMHHKFIEQE     248
M_gallinaceum   NMLRRGFIQAHLDILKTS--EKENYTRPYVSGGDIKNIC-----------K-TKHKVVDN     232
M_synoviae      ENLKNGFIKQIICKS-------NSNYPVYISGGDISNLS-----------PEISHQFVDN     236
M_iowae         NNIRSGFVNSFYKQAKLK--VENQTMACIISGGECHDII-----------SDFDYIIDEN     238
M_penetrans     NNLRSGFAYNFYNQAKKD--NLNSDLKCIITGGESYNINI----------SSFEYEINKE     241
M_alligatoris   HHLRNGFALSFISSLEKE--LNIAQTFIVASGNAAGNFK-----------NNYKINVDKY     238
M_crocodyli     FHHRNGFLLSFVNSIKAK--YDDKNFYIVASGNAATSFN-----------NNHFVEINKY     243
M_alvi          YHFKNGFMSSFYNMALKT--IKSNELLIILTGFEASNSE-----------LNFKYVIDKN     242
M_pirum         SNLKNGFVSYLYDSVKSLYKFKCKNLMCIVCGHDINKLN-----------VNFKYIKNND     242
                                                   *

M_pulmonis      --------------------    224
M_molare        MVIKSLILTNYKGE------    242
M_mobile        MVILGLKDYYEIFK------    243
M_testudinis    LVLQGLEIFFNKMVLKNG--    248
M_buteonis      LVLKAYFWVSTITYKK----    246
M_sturni        LITLGYLKAYLETK------    273
M_columborale   MVTKGVLYSLSKQKK-----    273
M_cricetuli     AVTLGYLLTLKKD-------    272
M_anatis        TTSKGYLLIFKNFKRTEEIF    268
M_gallinaceum   IVLKGYYQIFEDNFNL----    248
M_synoviae      IVLKGYLLIYLKNC------    250
M_iowae         AIIIGFANIFFLNTNKKATN    258
M_penetrans     AILLGFKKIYFLNN------    255
M_alligatoris   AILEGYLNILITNSK-----    253
M_crocodyli     AILEGYLNILTINIK-----    258
M_alvi          AILIGYYLIHKTNNW-----    257
M_pirum         AILIGYFLILKINL------    256
```

Supplementary Figure 2

CoaBC - CLUSTAL O(1.2.4) multiple sequence alignment

MEME Motifs highlighted in grey.
Regions with possible activity based on UniProtKB information for *Clostridium innocuum* (A0A099I6T6): blue=proton donor; yellow=CTP binding site; magenta=CTP binding site via amide nitrogen; green=nucleotide binding site.
Red blocks= MEME motifs when max 6 motifs selected.

```
M_arginini       ---------------MKILLLVTSSIAIVKLGDFLNLLKKDSKNQITVVLSKNASKYKLN   45
M_synoviae       ---------------MKILLLVTSSIAIVKLGDFLNLLKKDSKNQITIVLSKNASKYKLN   45
M_columborale    ---------------MKILLLITGSVASIKAKKLYDLLIRNNH-EVRVAISESAHNFVP-   43
M_cricetuli      ---------------MKILVLITGSIASIKSKKLINLLEKNNH-EVKYALTDSAYNFVN-   43
M_gallinaceum    ---------------MKILIIASSSVAIKKLESLISNLEELDW-EIKILFTNKAGEIFT-   43
M_anatis         ---------------MKILILSSASIALKKTNQLIKVFSENNH-EVKFALTDNALKMNL-   43
M_sturni         ---------------MNLLVLVTGSIAAIKSHLLVKKLIEQKH-TVKVAFTEAGKEFVS-   43
M_mobile         --------------MKQIGIIVTSSVAIIKIKTLKEILFLKGF-EVFIILTTN------A   39
M_testudinis     MNTMVLNDKCIGEYMANIIVYACSSVAIIKTNKLIKKLEEQNH-QVKVLVSQNVNKNVVE   59
                               :: :   .*:*  *   : . :       :   .:
                                                   Motif 1…
M_arginini       FPKEDENL-KYL-----YSESYIKKDPQHVYLARDNDLTILFAATYNTITKFARGIADNF   99
M_synoviae       FPKEDENL-KYL-----YSESYIKKDPQHVYLARDNDLTILFAATYNTITKFARGIADNF   99
M_columborale    -KH--IFE-NSL-----EFKWYENATSVHIEAPKWADRIIVYPASFNTIGKVSNAIADNF   94
M_cricetuli      -QS--DFP-EAL-----NKIWYQKGILEHIEAVLWADLIIVYPITYNTLNKIALGINDNF   94
M_gallinaceum    -FSTERYN-DYVLNQNKDMEDFVHNPSFHVELAKEFDHILIYPATFNTINKYANGIADSL   101
M_anatis         -VK--LDH-NYV-----VDDDFNNLTTKHIELAKWADKIIVYPATFNTINKFANGITDNF   94
M_sturni         -LK--DFT-NYY-----DNTWYKNKKSDHIDLFKWADRIIVYPATYNTINKVANGINDNF   94
M_mobile         LIH--YKNIQELNPV--LLNQDLTKQSEHIAFVKRIDLFIMAPASANSISKFVNGICDNM   95
M_testudinis     LEN--YFNITYLNEKISLNKENKDFPSDHIKLAKETDLVIVAPATTNFLAKYNSGICDEI   117
                                              *:      *  ::   : * : *    .* *.:
                 …Motif 1                                          Motif 2…
M_arginini       LTSILAVNKK-PVVILPAMNSNMWENPINLEHVSKLKKY-GHIFIGPDCGTLYCNDFGIG   157
M_synoviae       LTSILAVNKK-PVVILPAMNSNMWENPINLEHVSKLKKY-GHIFIGPDCGTLYCNDFGIG   157
M_columborale    ITSVMSVAPFDKTIFCPAMNTKMYHNPFLQANIVKLKQQ-GAIFLGPAKGLLKDGEIGIG   153
M_cricetuli      ITAIFSTQCFQKTIMCPAMNKNMYYSEVLQKNIKKLINN-GVIFIGPESGQLKDGMIGIG   153
M_gallinaceum    ATTILAMGFHNKTIIAPAMNFNMYQNPILQASIEKLKKI-GVTFIGPNYGILQCGDIGIG   160
M_anatis         VLSILSIGFFNKIYVCPAMNYRMFDNLILQENINKLKKL-GVSFIGPEFGQLYCGEEGYG   153
M_sturni         VTNLLSIGGFNKWIVCPAMNTKMYANPILQENIIKLKKL-GVTFLGPDAGMLYDGDNGIG   153
M_mobile         ALAMLM-ATNKPILFAPSMNDLMYESILKRNHIETLENW-GHYIIGPMYGKLSEGYDAIG   153
M_testudinis     NLAFLY-AYDGPILFAPAMNDVMWKSLNARNIVENLKNMSNHFFIGPNFGMLYEGYEGIG   176
                    .:          . *:**   *: .       : .* :   ::**  * *   .   . *
                 …Motif 2                            Motif 3
M_arginini       KMVKISDIYERLFG-----KNKPKLLLSFGYTKSPIDSVRSLMVPSSGKMGLALISELAF   212
M_synoviae       KMAKVSDIYERLFG-----KNKPKLLLSFGYTKSPIDSVRSLMVPSSGKMGLALISELAF   212
M_columborale    RVVEPQAVIDFLEN-----KNPKKILLTFGYTQVKLDPVRSVAVHSSGKSGLALIQTLSE   208
M_cricetuli      RVSEPERVIDFIEN-----KKGPKILLTYGYTTVKLDPVRVVSVPSSGKTGILLIHELAK   208
M_gallinaceum    RVIEPYEVYGFLTN-----QNKPKLLISIGYTNVYLDDVRTVSVKSSGQMGIFLAKELSK   215
M_anatis         RVSEPCDVLNFFNN-----ENKKKLLITIGYTEVKLDDVRTVSVRSSGKMGLSLINELKN   208
M_sturni         RVVEPDFVYDFLSC-----KPKKKVLITFGFTKVYLDPVRTVSVFSSGKSGLALIQELAK   208
M_mobile         RMAEPEEIADFASNILNNQKTSKKVIVSYGASKIYLDPIRFISNDSSGLFGKLIIKELKL   213
M_testudinis     RMSEVDEIIEIANNLLNIK-KDKRIVISYGASKIWLDQIRYITSGATGIFAKAINKELSK   235
                 :: :    :                   ::::: * :   :* :* :    ::*  .   :    *
                                        Motif 4
M_arginini       D-FDLTVINASLQHLNSKIPANVKKVVNVNYIDEYKEAVFSEIKNSDGFISVAAVSDFIFE   271
M_synoviae       D-FDLTVINASLQHLNSKIPANVKKVINVNYINEYKEAVFSEIKNSDGFISVAAVSDFIFE   271
M_columborale    G-YDVTVINGNLPHLNEYIPSHVKVVNISSVNEYYKAVDEHIVNNDCFISLCAVSDLIFE   267
M_cricetuli      F-ANLTVISGNLKHLNNKIPSSVKVINIETVNQYYEAVDKYIVENDFFISLCAVSDLIFK   267
M_gallinaceum    Y-FDLTIINANISQLNNYFDSRTKIINVDSVFEYQEAVFKYIKKNDVFVSVAAVSDFIFE   274
M_anatis         T-FDLTVINSNLPELNKYINNDIKIINVSTVDEYFEAVKNSIESQDIYLSVAAVSDLIFD   267
M_sturni         S-FDVTVINGNLEHLNTYIPLQVKVINVKLVDEYFKAVQDNIKKHDIYISLAAVSDLIFE   267
M_mobile         LGFYVDYVDASKN---------------------SNQQILDKVKDYDIYISSAAISDFLVE   253
M_testudinis     QGYQSDLINVSLM---------------------TNEMLSEKISKYDIYIASGAIANFNAV   275
                          :. .                          : : . : . * :::  *::::
```

```
M_arginini          KIDNKISKNNSSGVLKYHIGSDVLKEVSNKYPNKIMVGFSLGDSNNYKEKAKEKLINKKL      331
M_synoviae          KIDNKISKNNSSGVLKYHIGSDVLKEVSNKYPNKIMVGFSLGDSNNYKEKAKEKLINKKL      331
M_columborale       SQEHKIKKNAP-VDFKYQIGRDVLKDISHRYPQKLKVGYALET-NDILDNGLKKLTSKNL      325
M_cricetuli         PKLTKIKKNEK-IAFEYKIGRDVLKSIALKYPNKIKIGFALES-NDLLKNGLEKLDEKKL      325
M_gallinaceum       KHEAKIKKSDN-VSFEYKIGMDVLKEVSILYPDKIKIGFALES-QNLLENGTKKLKTKKL      332
M_anatis            KFDGKIKKDSE-FQFTYKIGVDVLKWVSENYPDKVRIGFALES-QNVLENGLKKLKNKNL      325
M_sturni            KQKQKIKKNED-IKFNFHQGVDVLKWVSDNYPNKIKIGYALES-SNLIENGRSKFNNKKL      325
M_mobile            KSERKIKKNSI-KTLELKNNIDVLTELRKLNKNIKILGFKLDE--DIQNAKDKMQK-LNL      309
M_testudinis        QHDGKYSDSKE-WELHLFKNIDVIDQATKLNQKLKVFAFKYDN--DINKAYIKLQHNEQI      332
                        * ...     :    . **:        .   ..:      :  .   .     ::

M_arginini          SLIVANSISSLNNENINASLLDKNLNEIYFENVSKYDFAKEIKNQLWKIIKAK-    384
M_synoviae          SLIVANSISSLNNKNINASLLDKNLNEIYFENVSKYDFAKEIKNQLWKIIKAK-    384
M_columborale       NMIVINDSSSLANNTSSGYIVVKDNSPLEFKNLNKHELAQQIKDVLDKIWID--    377
M_cricetuli         NAIVINDSNSLKNNTSSGYLVIKNEKPIFFENLNKNELAQKIAKMVEQLWKK--    377
M_gallinaceum       DLIVVNNKNTLKSQFSTGFLIEKD-RFNEFAQISKHNLAQLISEKVYRIWKEKQ    385
M_anatis            NMIIVNNKETLKSNLSSGYIITNN-SQREFNNLSKTELAKKIVREIKNEIK---    375
M_sturni            DLLVVNNSLTLGSNTSTGFFITKE-NEEAFKDLDKTKLAQIICQKIEEICKK--    376
M_mobile            DGILYNQINSMNTSKINGTLFINN-QEIQFQNKAKYELAKIIAKEVAKWIYT--    360
M_testudinis        KAILYNEIGAMGFNKITGALINKA-GKKEVINKSKSEVAELIVEEVLSW-----    380
                    . :: *.  ::  .  .. :. :      . :  * ..*: *   :
```

Supplementary Figure 3

PPAT- CLUSTAL O(1.2.4) multiple sequence alignment
Use file: PPAT without pdb.clustal

MEME Motifs highlighted in grey.
Regions with possible activity based on UniProtKB information for *M. pulmonis*(Q98RB3): yellow=substrate/nucleotide/ATP binding site; magenta=substrate binding via amide bond;

```
                                                                  Motif 1                    Motif 2…
M_mobile                              --MKIIKEETKNALFVGSFTYFKMGHFDVVKRALKVFDYVYIAPSINEEKNNENSLEERY    58
M_iowae                               -----MNNKKNAAIFPGTFEVFHLGHVDILKKALKLFKKVYVVVSNNPEKDP-KTLDQRF    54
M_leonicaptivi                        -------MNKTIAAFPGSFDPFHNGHLHILEKALKIFDEIYIVIAQNPKKDK-NNFQKNV    52
M_canis                               ------MQKVKKAIYPGSFNPFHLGHKEVLKKALKIFDEIYILVAQNPDKEK-NDIEQNI    53
M_pulmonis                            -----MSMNKKIAIFPGSFDPFHNGHKHILNKALALFDLVYLVITINPDKITKTSFDQRK    55
M_felis                               --------MKNSAIFPGSFNPFHKGHLNVLIKALKIFDKVYLVISQNENKET-NNFKENY    51
M_molare                              ---------MNKAIFPGSFDPFHRGHLSILEKALKLFDFVYLVITLNPDKNQRSSFESKR    51
M_buteonis                            -----MQENKKIALFPGSFNPWHSGHELILKKALKLFDFIYVVVTYNPDKNNLTDNDVNY    55
M_iners                               --------MKKIGIYPGSFDPLHEGHLAIIKKALSLLDKLYVVVTYNPDKDNLSFINERF    52
M_alligatoris                         ------MNKQKKAIYAGSFDPLHDGHVSILLKALKLFDYVYLVVSINPDKDNYSNLDLRY    54
M_crocodyli                           -----MKAKSKKAIYAGSFDPFHKGHLELLKKSLKLFDYVYLVVSINPDKNNALNLDRRY    55
M_bovigenitalium                      ---------MRKAIYAGSFDPLHNGHKNVIEKALKIVDELIIVVSQNPDKNNLKNIEERF    51
M_californicum                        ---------MKKAIYAGSFDPLHSGHKAVIIKALKLVEHLIIVVSNNPDKNNLSNLHQRY    51
M_simbae                              ---------MRKAIYAGSFDPLHSGHQAVITKALKLVDHLIIVVSNNPDKNNLDNINERF    51
M_conjunctivae                        MLSNPLNHKEKQAIFAGSFDPLHEGHLSIINKALKIFDHLFVVVTINPDKSDASDIEARF    60
M_gallinaceum                         ------MKTDKKAIYAGSFDPFHDGHFQILQKAARLFDFVYVIVSINPDKPGASDIDIRY    54
M_felifaucium                         -------MSKKVALYPGSFDPFHLGHLSVLKKALRIFDEIHVVVSINPDKENLDNIDERY    53
M_sturni                              -------MKSKIAIYPGSFDPLHKGHIQILKKALKLFDLIYLVVTNNPDKIEQSNLETRL    53
M_columborale                         -------MKNNIAIYPGSFDPFHEGHKQILKKAIDLFEHVYVIVTNNPDKDNQSNIIDRY    53
M_cricetuli                           -------MKSKIAIYPGSFDPIHKGHIEILKKALNLFDKIYVIVSVNPDKLHQNDIQIRY    53
M_anatis                              -------MNKKYAIYPGSFDPWHKGHMEILKKALKIFDAIFIIVSINPDKTNQSDILERY    53
M_gallinarum                          --------MEKVAIYPGSFDPLHKGHIAVIQKALKLFDKLIVVVSINPDKDNLINIEERY    52
M_synoviae                            ---------MKKAIYPGSFDPIHKGHINIIEKAVKLFDYVYVIVSINPDKNNLTGIKTRF    51
M_agalactiae                          ---------MKSAIYPGSFDSMHEGHIAIVKKALKIFDKLFVIVSVNPDKESVSDIDKRF    51
M_bovis                               ---------MKAAIYPGSFDPLHEGHIAIVKKALKIFDKLFVIVSVNPDKERINDIDTRF    51
M_fermentans                          --------MIKKAIFPGSFDPIHKGHISVIEKALKLFDELIVIVSINPDKNNLGNLEERY    52
M_opalescens                          ---------MKKAIYAGSFDPIHDGHISVIKKALKLFDFLYVIVSFNPDKDNSSNIVKRF    51
M_lipofaciens                         ---------MKKAIYAGSFDPLHDGHISVIKKSLKIFDKLVVIVSINPDKNNLENIEQRF    51
M_columbinum                          --------MKKIAIYPGSFDPMHEGHLSVIDKALKLFDKLFVIVSINPDKNNLENIDERY    52
M_primatum                            ---------MKKAIYAGSFDPLHEGHIEIIKKALKIFDKLFVIVSINPDKDNLENIEQRY    51
M_penetrans                           ---MSLNQKKKACIFPGTFEVFHDGHLNILKRALKLFDFVYIVVAINNTKT-SSDLEKRY    56
M_putrefaciens                        ---------MKIAIYPGSFDPFHQGHLNVVRKASLLFDRLYIVVSRNINKTTSFKLDSRV    51
M_yeatsii                             ---------MRIAIYPGSFDPFHDGHLNIVKKASSLFDKLIIVVSKNINKSSLLSFEKRI    51
M_feriruminatoris                     ---------MKTAIYPGSFNPFHKGHLNILKKASLLFDKLYIVVTKNVNKSLDPDLNSRV    51
M_mycoides_subsp_mycoides_SC_str_PG1  ---------MKTAIYPGSFNPFHNGHLNILKKAILLFDKVYVVVSKNINKSLDPDLQSRV    51
M_leachii                             ---------MKTAIYPGSFNPFHKGHLNILKKAILLFDKVYVVVSKNVNKSLDPDLQSRV    51
M_mycoides_subsp_capri_LC_str_95010   ---------MKIAIYPGSFNPFHKGHLNILKKAILLFDKVYVVVSKNVNKSLEPDLQSRV    51
M_capricolum                          ---------MKIAIYPGSFNPFHKGHLNILKKAILLFDKVYVVVSKNVNKSLDPDLQSRV    51
Candidatus_M_girerdii                 ---------MRIAIFPGSFDPMHEGHIDIIKRASKLFDQLIVTISINTSKQHQTSLNERY    51
M_testudinis                          --------MNKIAIFPGSFDPFHIGHLHVLKKALKLFDEVVLAVSINIKKTNASDMNIRV    52
M_alvi                                -------MKKRIALFPGSFDPIHSGHIHIIKKAIDLFDELILCVSINVDKLYDLSLLERK    53
M_pirum                               -------MSKKIALFPGSFDPIHIGHIHIIEKASKLFDEVIVCVSINIDKQQKTSLHKRK    53
                                                    : *:*   : **  :: ::  :.. : :  : *   *        .

                                      …Motif 2          Motif 3           Motif 4…
M_mobile                              LKLKNKFKNFK--RIEIIK--NTGDSSSLAKKLNAS-IVRGVRIDDENIFIRDSLYEKKL   113
M_iowae                               LRTKIVLQQLKLANVEVVK--NEGRTIDIANKLECYYIIRGVRNNFDL------DYEIKL   106
M_leonicaptivi                        INIKNKTKHL--KNVFVLI-NKNELTANFIKKYNINFLIRSLRDEKDY------WYEVNL   103
M_canis                               KNIKLQINEL--QNVFIIENHKNKLTADIAKEMNINFLIRSARNNADY------SYEFQL   105
M_pulmonis                            TLLENEILDFDKSRVQVLV-NKDSLTAEIAKKLGAKFIIRSARNDIDY------QYELVL   108
M_felis                               LLIKKMIGNL--KNVEVLI-NQNKYTAELAKNLNVNFIIRSARNNLDY------EYELDL   102
M_molare                              KIVEQQIKHL--KNVEILI-NQDKLTAELAKELNVNFLIRSARNDIDF------NYEIEL   102
M_buteonis                            QFIKNELKKINSTKIKVLQ-NKNKMTAELAKELKCEFLIRGIRNAEDF------EYEKKL   108
M_iners                               QKVSKALKNED--KIVVLL-NKNKLTADLAKEIEAKFLIRSARNNLDY------QYELEL   103
M_alligatoris                         QKILQTIKKYNFTNVEVLL-NKDDFIANIAKKYQVNFLIRSARNDVDF------SYELEL   107
M_crocodyli                           KNVCDIVEKEKLLNVEVLL-NTNDLIANVAKKYGVNFLIRSSRNENDF------QYEMEL   108
M_bovigenitalium                      EHVKKIYQNNS--KIIVIC-NKDELIGNIAKKMNVKLLIRSARNDLDY------KVELDI   102
M_californicum                        EEIKAMYANNT--KITVIK-NENELIGNIAKQHNVNFLIRSARNNIDY------ELELDI   102
M_simbae                              EHIQNVYQTNP--NVTVLC-NKNELIGNIAKREGVNFLIRSARNNVDY------QIELDI   102
M_conjunctivae                        LSVKEQLSDFK--NVTVIK-NSNKLTAELAKELNIKFLVRSARNNLDL------NYEMSL   111
M_gallinaceum                         NNTKKLLSNLT--NVQVLK-NKDRFVAEIAKELDVKYLVRSARDVIDF------NYELEL   105
M_felifaucium                         LNVKNELKNFK--NVFVSI-NKDDLTANIAKKNGANFIIRSARNDIDY------NYELQL   104
M_sturni                              NFAKEQLKNLP--NIIVIS-NPNKLTGELAKELKANFLIRSARNITDF------NYEIEL   104
M_columborale                         NFVNQQLQKFK--NVTVLI-NENELTAVFAKKLKARYIIRSARNIKDF------NYEIEL   104
M_cricetuli                           QLVKEKTKQFK--NIEILI-NENELTALVARKLKANFIIRSARNILDY------NYEIEL   104
M_anatis                              NDVKDKVKSLT--NVKVLI-NKNGFIADIAKELNVNFIIRSARNNTDF------NYELEL   104
M_gallinarum                          QTIKNQLASFK--NVEVLL-NRNQLIGELSKDLNVQFLVRSARNQIDY------DYELDL   103
M_synoviae                            LNTKKKLSKFE--NVEVLL-NEDDFIANIAKKHDCNYLIRSARNIKDY------SYEIEL   102
M_agalactiae                          VEAKEKLKEFK--NVEVLI-NKDGLIAEIAKKLGANFLVRSARNNIDF------QYELVL   102
M_bovis                               AEAKMKLKDFK--NVDVLI-NKNELIAEMAKKLGANFLIRSARNDTDF------KYELTL   102
M_fermentans                          QSVKKTLKDYK--TVTVVK-NENDLIAQIAKEKNVKFLVRSARNNLDY------DYELVL   103
M_opalescens                          DHVKTILSNIS-PNIEVLI-NKDAFIADIAREKKVNFLVRSARNDTDY------NLEVDM   103
M_lipofaciens                         KNVKEKLKNFD--NVDVIL-NKNNLIANIASELDINFLIRSARNQIDY------EDELVL   102
M_columbinum                          LETKKMLSHYG-DRIDVLI-NKNNLIGQIAKNLNVNFLVRSARNDTDY------KYELEL   104
M_primatum                            DYVKKQLSSFN--NVEVLI-NKNELIGNIAEELGVNFLVRSARNDTDY------KYEIEL   102
M_penetrans                           QKVEEKIDSLSIKNVEVIK--WDSKISDFAKIKTIYFIIRGIRDVNDF------KFEKYI   108
M_putrefaciens                        RLITEVTKDI--KNVEVIR-NDDKLTTDIAKLVDAKYIIRGLRNKNDF------DYELAY   102
M_yeatsii                             EIITNLTKDF--KNISVIA-NKDQLTTDIAKKLNAKYIVRGLRSQQDF------DYELNY   102
M_feriruminatoris                     ENIKNLTKDL--KNVEIII-NENELTTNIAKKLNASFIIRGLRSQADF------EYELKY   102
M_mycoides_subsp_mycoides_SC_str_PG1  KNIKNLIRDF--NNVEIII-NENKLTTTIAKELNASFIIRGLRSQTDF------EYEIKY   102
M_leachii                             ENIKNLIKDF--DNVEIII-NENKLTTTIAKELNACFIIRGLRSQTDF------EYEIKY   102
M_mycoides_subsp_capri_LC_str_95010   ENIKNFIKDF--SNVEIII-NENKLTTTIAKELNASFIIRGLRSQADF------EYEIKY   102
M_capricolum                          ENIKNLIKDF--SNVEIII-NENKLTTTIAKELNACFIIRGLRSQADF------EYEIKY   102
Candidatus_M_girerdii                 TKVLEQVNQLGLDNVCVKI--NNGLTVEFAKKNKANYIVRSFRDNNDI------EYEMTI   103
M_testudinis                          DLVSKKIKQLKLKNVTVQG--WTGYTVDLAKKLKAKYLIRGIRNFADF------QEELLN   104
M_alvi                                SKVEKVIKKLKLKNVKVIS--WPSYIVDAAKLYKAKYLITSVRNMDDF------QQSLHN   105
M_pirum                               IEVQKIIKSLNFKNIKVIL--WSKYIVDAAKKYKAKYLISGVRNIRDM------KQQLYN   105
                                                    : :                    :: . *   :         .
```

Supplementary Figure 4

DPCK-CLUSTAL O(1.2.4) multiple sequence alignment
Use file: 3DPCK.clustal

MEME Motifs highlighted in grey.
Regions with possible activity based on UniProtKB information for *M. pneumoniae* (A0A449A0Q7): yellow=nucleotide binding site (ATP); underlined sequences=P-loop/Walker A sequence motif (GXXXXGKT/S where X represents any residue).

```
Legionella_pneumophila                  ------------------------------------------------------------     0
M_mycoides_subsp_capri                  ------------------------------------------------------------     0
M_leachii                               ------------------------------------------------------------     0
M_mycoides_subsp_mycoides               ------------------------------------------------------------     0
M_capricolum_subsp_capricolum           ------------------------------------------------------------     0
M_capricolum_subsp_capripneumoniae      ------------------------------------------------------------     0
M_putrefaciens                          ------------------------------------------------------------     0
M_yeatsii                               ------------------------------------------------------------     0
M_sturni                                ------------------------------------------------------------     0
Ms02                                    ------------------------------------------------------------     0
M_anatis                                ------------------------------------------------------------     0
M_alligatoris                           ------------------------------------------------------------     0
M_crocodyli                             ------------------------------------------------------------     0
M_leonicaptivi                          ------------------------------------------------------------     0
M_canis                                 ------------------------------------------------------------     0
M_felis                                 ------------------------------------------------------------     0
M_buteonis                              ------------------------------------------------------------     0
M_mobile                                ------------------------------------------------------------     0
M_columborale                           ------------------------------------------------------------     0
M_cricetuli                             ------------------------------------------------------------     0
M_opalescens                            ------------------------------------------------------------     0
M_arginini                              ------------------------------------------------------------     0
M_synoviae                              ------------------------------------------------------------     0
M_ovipneumoniae                         -MFYKIAFIDLDGTLLDIGKGKNAQISDTNLHSVRKLAKECKIVISTGRKFSTDIVNIGK     59
M_hyopneumoniae                         -MFYKICFFDLDGTLLDIGRGRKAWISKKNSDAVRKLANKCIIVISTGRKFNSKVALIGR     59
M_dispar                                -MLYKIGFFDLDGTLLDIGRGRNAKISEKNRQSLNKLAKNCMVVISTGRKFTSEVAIIGQ     59
M_flocculare                            -MVYKIGFFDLDGTLLDTGRGRNAKISKKNQQAVRKLAKNCIIVISTGRKFNSTIAVFGK     59
M_bovoculi                              -MKYKITFIDLDGTLLDLGYGVKSVVSNANIRAVRKLSNSSIIVISTGRKMSKKIQSIGK     59
M_conjunctivae                          MNKKKIVFLDLDGTLLDIGYGVTANMSNINKKAVQKISQNYTIVISTGRKLSEKIKKIGQ     60
M_pulmonis                              ------------------------------------------------------------     0
M_hyorhinis                             ------------------------------------------------------------     0
M_collis                                ------------------------------------------------------------     0
M_molare                                ------------------------------------------------------------     0
M_gallinarum                            ------------------------------------------------------------     0
M_lipofaciens                           ------------------------------------------------------------     0
M_fermentans                            ------------------------------------------------------------     0
M_bovigenitalium                        ------------------------------------------------------------     0
M_californicum                          ------------------------------------------------------------     0
M_simbae                                ------------------------------------------------------------     0
M_columbinum                            ------------------------------------------------------------     0
M_iners                                 ------------------------------------------------------------     0
M_felifaucium                           ------------------------------------------------------------     0
M_primatum                              ------------------------------------------------------------     0
M_agalactiae                            ------------------------------------------------------------     0
M_bovis                                 ------------------------------------------------------------     0
M_iowae                                 ------------------------------------------------------------     0
M_penetrans                             ------------------------------------------------------------     0
M_genitalium                            ------------------------------------------------------------     0
M_pneumoniae                            ------------------------------------------------------------     0
M_gallisepticum                         ------------------------------------------------------------     0
M_imitans                               ------------------------------------------------------------     0
M_testudinis                            ------------------------------------------------------------     0
M_alvi                                  ------------------------------------------------------------     0
M_pirum                                 ------------------------------------------------------------     0


Legionella_pneumophila                  ------------------------------------------------------------     0
M_mycoides_subsp_capri                  ------------------------------------------------------------     0
M_leachii                               ------------------------------------------------------------     0
M_mycoides_subsp_mycoides               ------------------------------------------------------------     0
M_capricolum_subsp_capricolum           ------------------------------------------------------------     0
M_capricolum_subsp_capripneumoniae      ------------------------------------------------------------     0
M_putrefaciens                          ------------------------------------------------------------     0
M_yeatsii                               ------------------------------------------------------------     0
M_sturni                                ------------------------------------------------------------     0
Ms02                                    ------------------------------------------------------------     0
M_anatis                                ------------------------------------------------------------     0
M_alligatoris                           ------------------------------------------------------------     0
M_crocodyli                             ------------------------------------------------------------     0
M_leonicaptivi                          ------------------------------------------------------------     0
M_canis                                 ------------------------------------------------------------     0
M_felis                                 ------------------------------------------------------------     0
M_buteonis                              ------------------------------------------------------------     0
M_mobile                                ------------------------------------------------------------     0
M_columborale                           ------------------------------------------------------------     0
M_cricetuli                             ------------------------------------------------------------     0
M_opalescens                            ------------------------------------------------------------     0
M_arginini                              ------------------------------------------------------------     0
M_synoviae                              ------------------------------------------------------------     0
M_ovipneumoniae                         KISANFYVCQNGAEIYDQNLNLIFESTINQNIVEQILSFAKKWNVSISFDSKIVFSPPKS     119
M_hyopneumoniae                         QIKAKFYICQNGAEIFDKNFSTLFKTGIKQEIISKIIEITKRFNFVISFNSKVFFFKN-L     118
M_dispar                                KISAKFYVCQNGAQIFNENFKLLFEATIDAEIVNQIVFLVKKFKFIISFNSKIFYSKN-F     118
M_flocculare                            KILAKFYICQNGAQIFDENFNLIFQTTIKLEIVKKITELAKKLNFGISFNSQVFFTKS-I     118
M_bovoculi                              KIKAKYYICQNGGTIFDSNFKLIKHNTISKTIVEQITEIAFENKATIAFDDKTIYGKG-S     118
M_conjunctivae                          QINAKYYICQNGANIFDKNFNLLRKYEITDIIANDVIKLAFDNKSTVAFDEKYIYGDG-L     119
M_pulmonis                              ------------------------------------------------------------     0
M_hyorhinis                             ------------------------------------------------------------     0
M_collis                                ------------------------------------------------------------     0
M_molare                                ------------------------------------------------------------     0
M_gallinarum                            ------------------------------------------------------------     0
M_lipofaciens                           ------------------------------------------------------------     0
```

```
M_fermentans                            ------------------------------------------------------------      0
M_bovigenitalium                        ------------------------------------------------------------      0
M_californicum                          ------------------------------------------------------------      0
M_simbae                                ------------------------------------------------------------      0
M_columbinum                            ------------------------------------------------------------      0
M_iners                                 ------------------------------------------------------------      0
M_felifaucium                           ------------------------------------------------------------      0
M_primatum                              ------------------------------------------------------------      0
M_agalactiae                            ------------------------------------------------------------      0
M_bovis                                 ------------------------------------------------------------      0
M_iowae                                 ------------------------------------------------------------      0
M_penetrans                             ------------------------------------------------------------      0
M_genitalium                            ------------------------------------------------------------      0
M_pneumoniae                            ------------------------------------------------------------      0
M_gallisepticum                         ------------------------------------------------------------      0
M_imitans                               ------------------------------------------------------------      0
M_testudinis                            ------------------------------------------------------------      0
M_alvi                                  ------------------------------------------------------------      0
M_pirum                                 ------------------------------------------------------------      0


Legionella_pneumophila                  ------------------------------------------------------------      0
M_mycoides_subsp_capri                  ------------------------------------------------------------      0
M_leachii                               ------------------------------------------------------------      0
M_mycoides_subsp_mycoides               ------------------------------------------------------------      0
M_capricolum_subsp_capricolum           ------------------------------------------------------------      0
M_capricolum_subsp_capripneumoniae      ------------------------------------------------------------      0
M_putrefaciens                          ------------------------------------------------------------      0
M_yeatsii                               ------------------------------------------------------------      0
M_sturni                                ------------------------------------------------------------      0
Ms02                                    ------------------------------------------------------------      0
M_anatis                                ------------------------------------------------------------      0
M_alligatoris                           ------------------------------------------------------------      0
M_crocodyli                             ------------------------------------------------------------      0
M_leonicaptivi                          ------------------------------------------------------------      0
M_canis                                 ------------------------------------------------------------      0
M_felis                                 ------------------------------------------------------------      0
M_buteonis                              ------------------------------------------------------------      0
M_mobile                                ------------------------------------------------------------      0
M_columborale                           ------------------------------------------------------------      0
M_cricetuli                             ------------------------------------------------------------      0
M_opalescens                            ------------------------------------------------------------      0
M_arginini                              ------------------------------------------------------------      0
M_synoviae                              ------------------------------------------------------------      0
M_ovipneumoniae                         FLYLFSKLFSNLQVKNINKIDLPKSVKKILLFSPNIFKISKFRKFLEEFFSEKIQIYTIE      179
M_hyopneumoniae                         FIKFLNFFLKSFDFRPFRQILVPENVRKILIFSVNIWKIKKFAYVLEKIFSDNLQICLIR      178
M_dispar                                LVKFLAKFSKNFQFKSINEIFVPEKVRKILILSPCVFKIKKIKYILKKMFSQYIEISTIN      178
M_flocculare                            FIKFFRIFFKNFHFVSTTKIFIPKNVRKILIFASCSYKIKKLKYLLEKMFAEHIQISLIN      178
M_bovoculi                              WKHIFS-LFSEFRPKSSKKIQI-LDVEKILVICHSRKKILHIKKILKTHFLNNLNISWIG      176
M_conjunctivae                          WKTIFS-FFAEFKPRPISLIEV-KQIQKILIISAYKSRIRIIKNILKQRYQEQLQVSISG      177
M_pulmonis                              ------------------------------------------------------------      0
M_hyorhinis                             ------------------------------------------------------------      0
M_collis                                ------------------------------------------------------------      0
M_molare                                ------------------------------------------------------------      0
M_gallinarum                            ------------------------------------------------------------      0
M_lipofaciens                           ------------------------------------------------------------      0
M_fermentans                            ------------------------------------------------------------      0
M_bovigenitalium                        ------------------------------------------------------------      0
M_californicum                          ------------------------------------------------------------      0
M_simbae                                ------------------------------------------------------------      0
M_columbinum                            ------------------------------------------------------------      0
M_iners                                 ------------------------------------------------------------      0
M_felifaucium                           ------------------------------------------------------------      0
M_primatum                              ------------------------------------------------------------      0
M_agalactiae                            ------------------------------------------------------------      0
M_bovis                                 ------------------------------------------------------------      0
M_iowae                                 ------------------------------------------------------------      0
M_penetrans                             ------------------------------------------------------------      0
M_genitalium                            ------------------------------------------------------------      0
M_pneumoniae                            ------------------------------------------------------------      0
M_gallisepticum                         ------------------------------------------------------------      0
M_imitans                               ------------------------------------------------------------      0
M_testudinis                            ------------------------------------------------------------      0
M_alvi                                  ------------------------------------------------------------      0
M_pirum                                 ------------------------------------------------------------      0


Legionella_pneumophila                  ------------------------------------------------------------      0
M_mycoides_subsp_capri                  ------------------------------------------------------------      0
M_leachii                               ------------------------------------------------------------      0
M_mycoides_subsp_mycoides               ------------------------------------------------------------      0
M_capricolum_subsp_capricolum           ------------------------------------------------------------      0
M_capricolum_subsp_capripneumoniae      ------------------------------------------------------------      0
M_putrefaciens                          ------------------------------------------------------------      0
M_yeatsii                               ------------------------------------------------------------      0
M_sturni                                ------------------------------------------------------------      0
Ms02                                    ------------------------------------------------------------      0
M_anatis                                ------------------------------------------------------------      0
M_alligatoris                           ------------------------------------------------------------      0
M_crocodyli                             ------------------------------------------------------------      0
M_leonicaptivi                          ------------------------------------------------------------      0
M_canis                                 ------------------------------------------------------------      0
M_felis                                 ------------------------------------------------------------      0
M_buteonis                              ------------------------------------------------------------      0
M_mobile                                ------------------------------------------------------------      0
M_columborale                           ------------------------------------------------------------      0
M_cricetuli                             ------------------------------------------------------------      0
M_opalescens                            ------------------------------------------------------------      0
M_arginini                              ------------------------------------------------------------      0
M_synoviae                              ------------------------------------------------------------      0
M_ovipneumoniae                         KGFVIEITDFNASKGQAAVFISKVANISLNYSFHIGDSENDISAKNIVQTLILMKNSPRK      239
```

```
M_hyopneumoniae                      KFRVIEITDISASKGKAVEFITKFSNISLDYALHIGDSENDISTKKIVKMLIIMQSGAKN    238
M_dispar                             QGYAIEITDIQASKGKAVDFIAKFTNVSLNHTFHIGDSENDISTKNFVKTLIIMKSAKKK    238
M_flocculare                         KNYGIEITDIHASKGKAAEFIAKFNNISLTHTFHIGDSENDISTKNVVNSLIIMKSASKK    238
M_bovoculi                           KGFALEITDKNASKGLAAQFIAEKEQVSLKQTVHIGDSMNDATCKGIVGNLIAMKSGSKR    236
M_conjunctivae                       KGFALEITNSQASKGKAAKFIAELEGVDLKNTFHIGDSMNDASCSGIIGNLIAMKSGSKN    237
M_pulmonis                           ------------------------------------------------------------    0
M_hyorhinis                          ------------------------------------------------------------    0
M_collis                             ------------------------------------------------------------    0
M_molare                             ------------------------------------------------------------    0
M_gallinarum                         ------------------------------------------------------------    0
M_lipofaciens                        ------------------------------------------------------------    0
M_fermentans                         ------------------------------------------------------------    0
M_bovigenitalium                     ------------------------------------------------------------    0
M_californicum                       ------------------------------------------------------------    0
M_simbae                             ------------------------------------------------------------    0
M_columbinum                         ------------------------------------------------------------    0
M_iners                              ------------------------------------------------------------    0
M_felifaucium                        ------------------------------------------------------------    0
M_primatum                           ------------------------------------------------------------    0
M_agalactiae                         ------------------------------------------------------------    0
M_bovis                              ------------------------------------------------------------    0
M_iowae                              ------------------------------------------------------------    0
M_penetrans                          ------------------------------------------------------------    0
M_genitalium                         ------------------------------------------------------------    0
M_pneumoniae                         ------------------------------------------------------------    0
M_gallisepticum                      ------------------------------------------------------------    0
M_imitans                            ------------------------------------------------------------    0
M_testudinis                         ------------------------------------------------------------    0
M_alvi                               ------------------------------------------------------------    0
M_pirum                              ------------------------------------------------------------    0

                                                                     Motif 1           Motif 2…
Legionella_pneumophila               --------------------------MVYSVGLTGNIASGKSTVAEFFSE-LG-------    26
M_mycoides_subsp_capri               ----------------------------MIIGIYGKIGSGKTYISNKFIN-FH-----PE    26
M_leachii                            ----------------------------MIIGIYGKIGSGKTYISNKFIN-FH-----PE    26
M_mycoides_subsp_mycoides            ------------------------MVYKMIIGIYGKIGSGKTYISNKFIN-FH-----PE    30
M_capricolum_subsp_capricolum        ------------------------MVYKMIIGIYGKIGSGKTYISNKFIN-FH-----PE    30
M_capricolum_subsp_capripneumoniae   ----------------------------MIIGIYGKIGSGKTYILNKFIN-FH-----PE    26
M_putrefaciens                       ----------------------------MIIAVFGKIGSGKTTVCNKFVK-FH-----PS    26
M_yeatsii                            ----------------------------MIIGIFGKIGSGKTTISSKFTN-FH-----PK    26
M_sturni                             -----------------------------MIALVGQICSGKSFLAKELQK-RG-------    23
Ms02                                 -----------------------------MIAITGLLKVGKTTFLKRLEQ-EG-------    23
M_anatis                             -----------------------------MVAIIGEVASGKTFFVNQLKE-MG-------    23
M_alligatoris                        -----------------------------MIAIIGQVAAGKSLLLHNLNK-LG-------    23
M_crocodyli                          -----------------------------MIAIIGRIASGKSYLLNQMKK-LG-------    23
M_leonicaptivi                       -----------------------------MVAIVGKISSGKTTLLNYFQK-LG-------    23
M_canis                              -----------------------------MIAIVGKISAGKTTLLNWLAN-KG-------    23
M_felis                              ---------------------------MKRIAIVGKIGVGKTWLLNKLKE-LN-------    25
M_buteonis                           -----------------------------MIAVVGHACAGKTTALSSLEI-QN-------    23
M_mobile                             -----------------------------MIAITGKSGAGKTYYSTKLRK-LG-------    23
M_columborale                        -----------------------------MIIVTGLISSGKSTLLSKLNT-LG-------    23
M_cricetuli                          -----------------------------MIAITGKPCTGKTYFLNLVQK-LG-------    23
M_opalescens                         -----------------------------MIAIIGKIGSGKSTFINFLQK-NK-------    23
M_arginini                           -----------------------------MIAVVGKVCSGKTTFLKKLEQ-EG-------    23
M_synoviae                           -----------------------------MIAVVGKVCSGKTTFLKKLEQ-EG-------    23
M_ovipneumoniae                      LKKHAHIIGYKRKF-GVAKALENFIFNPKSIAIVGFYASGKTTFLKAVEK-FG-------    290
M_hyopneumoniae                      AKKNADFIGYKRKF-GVAKVINNFILEPKSAAIVGKYGSGKTTFLKKVEK-FG-------    289
M_dispar                             IKKIAHFVGYRRKLGGVAKSLENLIFRPKSVAVVGYYASGKTTFLKNIEK-SG-------    290
M_flocculare                         VKKNAHFIGYKRKF-GVAKAVNNLILSLKSVAIVGSYASGKTTFLKKIEK-FG-------    289
M_bovoculi                           LKNIADTIGFGK-HRGVAKTIEQFILKKPSIAIIGKYASGKTTFLKEVEK-YG-------    287
M_conjunctivae                       LQKIADNVGFAK-YGGVAKAIEKFIDKNISVAVVGQYGSGKTTFLKEVEK-FG-------    288
M_pulmonis                           -----------------------------MIAITGKAGVGKTTFLKKMEK-KG-------    23
M_hyorhinis                          -----------------------MENKHKTYAVIGKYAVGKTTFLNLLQE-YSKKVLKKE    36
M_collis                             -----------------------------MIAITGKYGSGKTTLLNKIAK-YN-------    23
M_molare                             -----------------------------MIAIIGKYGSGKTTFLKKIES-YG-------    23
M_gallinarum                         -----------------------------MIAIIGKIGVGKTKFINSLN--KK-------    22
M_lipofaciens                        -----------------------------MIAVIGKTCVGKTTFLNYLKN-KG-------    23
M_fermentans                         -----------------------------MIALIGQIGVGKSTFLENFKK-LG-------    23
M_bovigenitalium                     -----------------------------MIAVIGKIGVGKTTFLKNLGI-DN-------    23
M_californicum                       -----------------------------MIALIGKVGVGKSTFLRNSGL-KK-------    23
M_simbae                             -----------------------------MIAIIGKIGVGKTTFLKNCGI-EL-------    23
M_columbinum                         -----------------------------MIAVIGKIGVGKTTFLSEIKK-MG-------    23
M_iners                              -----------------------------MIAIIGKSGAGKSTLLNFLTK-QG-------    23
M_felifaucium                        -----------------------------MIAIIGKVASGKSTLLNKLKH-RG-------    23
M_primatum                           -----------------------------MIAIIGKIAVGKTFFSNKLIQ-KG-------    23
M_agalactiae                         -----------------------------MIAIIGKIGVGKTTFSKKLIE-RG-------    23
M_bovis                              -----------------------------MIAIIGKLGVGKTTFCKKLIE-KG-------    23
M_iowae                              -------------------MSKTKKDRSKLICVTGLVNKGKSTAMKIISS-YG-------    33
M_penetrans                          ----------------MFKMPKEEINQSKLICVTGFMGSGKSTFVNFLKQ-MG-------    36
M_genitalium                         ----------------------------MLIAIVGKPGVGKTSLLQYLKDNYH-------    25
M_pneumoniae                         ----------------------------MLIAVVGKAGVGKTTVLQYIADYFH-------    25
M_gallisepticum                      ---------------------MRNVKNRILVCLSGKSSSGKSMLINRLKQ-DG-------    31
M_imitans                            ---------------------MKNAKSKILVCLSGKASSGKTMLIDKLKQ-DG-------    31
M_testudinis                         ----------------------------MLVCVTGLSGSGKSTILKSSLL-SK-------    24
M_alvi                               ----------------------------MIVCITGISGTGKTTILNLL-S-ND-------    23
M_pirum                              -----------------------MPNFIMIICVTGQSGVGKTTILNLL-S-KK-------    28
                                                                     : *    **:
```

```
                                            …Motif 2                         Motif 3
Legionella_pneumophila                  INVIYADKIAKELTSKNTPCYQDIISHFGSSVVLNNGELDRKRIRDIIFSNS-NERLWLE    85
M_mycoides_subsp_capri                  FKIINADNVSKKLLEDSK--IKSKLFEIDNNFIKN-SNVDKKYLRKKLFTNK-KLKQQVD    82
M_leachii                               FKIINADDVSKKILENQE--IKSKLFEIDNNIIRD-DKVDKKYLRKTLFTNK-KLKQQVD    82
M_mycoides_subsp_mycoides               FKIINADDVSKKMLENQE--IKSKLFEIDNNIIRD-DKVDKKYLRKTLFTNK-KLKQQVD    86
M_capricolum_subsp_capricolum           FKIINADDVSKKVLENQE--IKSKLFEIDNSIIKD-NKVDKKYLRKKLFTNK-KLKQKVD    86
M_capricolum_subsp_capripneumoniae      FKIINADDVSKKVLENQE--IKSKLFEIDNSVIKD-NKVDKKYLRKKLFTNK-KIKQQVD    82
M_putrefaciens                          FKIINADLIAKELLNRQD--VKESLKKIDKSIITSKNQVDRKYLRKIIFANK-ELGEKVD    83
M_yeatsii                               FKIINADLVARKCLEQEE--VKNKLKKIDENILDNDNNIDRKYLREKIFSNK-EFGQKID    83
M_sturni                                FKIFNADIFVEELYFHNLDFVNFLRTLNLGYLIED-NKVSKPKIKALLKTKH-SDFFILE    81
Ms02                                    FKVLYLDDYVNLLYATNQFVIDTFQKQFGSQVIEQ-NQVSKSALKKILEDDF-SKIYEIE    81
M_anatis                                YKVFISDEYVNFLYEKNDEIKNNFRKVFGDLVIQN-NKISKEFLKQKIRENF-EYIYTIE    81
M_alligatoris                           YKTFSCDVFVHELYEKEFF-----IEQINNLINKK-TKHLKEEIIAWLKTDK-NNIYKLE    76
M_crocodyli                             YKTFSCDEFVGQLYEKSSFFADQISLEISSNLVVN-GFVSKDKVKQWLLEEN-SNIFKLE    81
M_leonicaptivi                          YKTFNSDIFVNQLYNNSEFCFNARKKISYDIFTND-NLISKIKIKNIIFKDT-NLLFELE    81
M_canis                                 HKTLEVDKFVINLYKDA-SFVEYVKNSLDYDLITN-NYVDKEKIKKWIIEDS-SNLNLIG    80
M_felis                                 YLTINLDEYINNLYSNDSVLINLFKNQLDNFLIVE-NKINKNKIKEWILLDN-RNFNKIE    83
M_buteonis                              YKILYADNFVSNLYQSNSAFLSKALKVLPATCFEN-NELSKVKIKAELKNNN-LILKELE    81
M_mobile                                YKVFIGDEFNNIIYSFNNEGYNLVKNHISGTLVNE-KGVDKPKLVAWILEDFKNNLEKLK    82
M_columborale                           FRVFNADVFVQNLYHDR-DFVNEINNSKWNFLIEN-SSVSKTKILNLLNNNY-QDFKVFE    80
M_cricetuli                             YTIFNGDDFVLNLYKDI-SFVHFLKETKFNFLIEH-EKVSKEKIRQIVINDY-KLFDEFQ    80
M_opalescens                            YKTLVCDDFLAISYLKDNSTYNAVNSHFGTKLNNA-YGLDKNKIRTLYQTDD-KEADKFN    81
M_arginini                              KKVFIADEFVKSLYENKDFCQ-KIQKLINFDLLTK-NKLDKFKIKKLFSENK-DLFEEFE    80
M_synoviae                              KKVFIADEFVKSLYENKDFCQ-KMQKLINFDLLTK-NKLDKFKIKKLFSENK-DLFEEFE    80
M_ovipneumoniae                         YSVLYTDEFYFNCFSENNPCFEIVK-KFKPDFIHN-NVLDKNKLRDFMVESQ-QNRDFIE    347
M_hyopneumoniae                         YSVLYTDEFFHNCYLASGECFKIIK-KIRPDFINE-NIVNKNKIRNFMLKSK-QNRDLIE    346
M_dispar                                YSVLYTDDFFANCYSTNGKCFEAIK-EIRADFVNE-NYLDKNKIRDFMLENE-GNRNLIE    347
M_flocculare                            YSVLYTDNFFKNCYLLNGDCFQAIK-KIRPDFICK-NVVDKEKIRDFMVKNE-KNRALIE    346
M_bovoculi                              YSVLYTDEFFKNSYLVGQLGYNVIK-QIDPNLVTK-EGVNKDKLRVFITQSE-LNRNLIE    344
M_conjunctivae                          YQVLYTDDFFASCYQNGNPCYFVVK-SINEDFVTT-DYVKKDKIRDFMLEKK-ENKDFLE    345
M_pulmonis                              YKILYSDNFFNQDYEKGNQSYQLIKDNLGQDFVND-QRVDKKRLKNWIKEDL-SNLDRLE    81
M_hyorhinis                             QKILFSDEFFQMCYLKDNPCYLALK-NYNPAFVKE-MKVNKEKLREFIRSNP-DNINIIE    93
M_collis                                YKVLNCDDFIKECYKKNNLCYLKIKKKLGEDFINH-DEVDKNKLREFILKDK-SNINIIE    81
M_molare                                FSVLYTDDFFKKCYEKNEECYCIIKESLGQEFVDD-KSVLKNKLREFIIENK-NNINIIE    81
M_gallinarum                            YKIFISDDFITRIYKKNEIGYFLIKKNIGSFVLNE-SGVDKSKLFLWLKKDK-KHWILLE    80
M_lipofaciens                           FSIFILDEFVNDLYEKSEKFYTFILNNLGEKYCIN-NKIDKLKIRTLFNQKP-EIIDLLE    81
M_fermentans                            LKTFNCDEFINREYQKNGKIYHKINQELGDFLNDK-NGISKEKIKLWIDQKA-HNLGELE    81
M_bovigenitalium                        SKIFFADEFVAKNYLLGSKLCQKIREEIGDFLLDE-NGVSKTKIKEWIGQNI-DNLERLE    81
M_californicum                          EKIFICDEFVAKEYKKYGILYNEIKQKIGTFLLDE-KGVSKKKILKWLFENT-DNIDKLE    81
M_simbae                                EQIFFADVFVAQNYEKNGLFYEPIKQKIGEFLIDE-NGVSKPKILAWLSQNT-ENILKLE    81
M_columbinum                            YKVFNSDEFIKSEYKTDGLLYKAIKKEFGTLLLDE-FGVNKEKIRQWIKDDI-KNLDLLE    81
M_iners                                 YQTFNCDNFIEKEYKKDGFLFKAINQEIGTFLNDD-NGISKDKIKTWIAQNN-NNINVLE    81
M_felifaucium                           FKIFNCDKFFIEEYKKNGFFYNEINNKIGSFLNDE-NGISKQKIKEWISQNP-NSLDVLE    81
M_primatum                              FTVFNCDDFFNHSYLKNNDCYMKINSEIGSFLNDE-NGISKTKIKSWISQNP-NNIDLLE    81
M_agalactiae                            FSVFNCDEFVQKSYQKGNEGYEAIKNQIGDFLCDE-NGVSKRKIKSWISQNP-YNIDLLE    81
M_bovis                                 FSVFNCDDFVQESYLKGNDCYKEIKRQIGDFLCDE-KGVSKSKIKTWIAQNP-YNIDILE    81
M_iowae                                 YDVFIMDEYIHKIYKKNDIGYNAIKTNFGSDYVNE-QAVDRDKLRELILNDI-KYREKLN    91
M_penetrans                             CETFVADEFVHNSYLKGNIGYKIIKDNFGTDYVND-ECVDRPKLRELILNNQ-EKKFLLE    94
M_genitalium                            FSVFYADSFIHEQYQKNNPGYQLIMDHFGKEFVNQ-TEVDRKKLANYVFSDD-KLIEKLS    83
M_pneumoniae                            FPVFFADRFIHQQYANGQAGYAIVKQQFGAQFVNH-EAVDRKQLAQYVFNQP-DELKRLS    83
M_gallisepticum                         YYTINLDELIHQYYQKNQSGYDFVVESFGLEYVDE-NQVNRKKLGQLVFANP-DKLKLLS    89
M_imitans                               YYTIVLDQLIHQYYQKGKEGYQFVLDNFGSEYVDE-NQVIRQKLGQLVFGNP-EKLKLLS    89
M_testudinis                            YSVFHMDEIIHQLYQPDYPGYNIIKKTFGPGFITE-QGVDRKKLGKLVFSNS-VELAKLN    82
M_alvi                                  FSIKILDNEIHSYYKYDKIGYKLIQKHFGNEYVNS-KEVDRQKLGTLVFNDK-KKLIYLN    81
M_pirum                                 YYVEFLDNLIHTEYKVNNQGYNLIKEHFGIKYVDS-NKVNRKKLGQLVFSDK-HLLNKLN    86
                                              *                                 :  :      .       .

                                                            Motif 4
Legionella_pneumophila                  SLLHPVIRKKIEEQLIVCTSPYCLIEIPLLFNKHHY------PYLQKVLLVIAPLESQLD    139
M_mycoides_subsp_capri                  SLLWPLISKEIQKELKNNPNTNYIIEAALLFELN-L------TNLDLIVKVKSSLLKSIF    135
M_leachii                               SLLWPLISKQIQKEIKNNPNTNYIIEAALLFELN-L------TNLDLIVKVKSSLLKSIF    135
M_mycoides_subsp_mycoides               SLLWPLISKQIQKEIKNNPNTNYIIEAALLFELN-L------TNLDLIVKVKSSLLKSIF    139
M_capricolum_subsp_capricolum           SLLWPLISREIQKEISNNPSSNYIIEAALLFELN-L------TNLDLTVKVKSSLLKSIF    139
M_capricolum_subsp_capripneumoniae      SLLWPLISREIQKEISNNPSSNYIIEAALLFELN-L------TNLDLTVKVKSSLLKSIF    135
M_putrefaciens                          MLLWPMITEEINQQISNQP-ADYIIEVALLDQLK-L------EHVDLKIKVKSNIFKTLF    135
M_yeatsii                               DLLWPIISDEINKEISFDPTANYIIEAALLNKLK-I------PNIDFKVKVKSNLFKIAY    136
M_sturni                                RIVHTYIWLHLR------QHVYDFVELPVLNSPYVD----FSVFFSHIINIDNSDQQRWT    131
Ms02                                    KIVFPLIYKHLE------ENQYDLVEIPILKNDFVN----FEKFFNLIINVKKSKKKTEK    131
M_anatis                                DMVFPHIFSHFK------TQKYDFAEIPVLVSKNIN----FLTFFNKVINVVPSTEKLDL    131
M_alligatoris                           NIVFPHVYRHLQ------NNSYDFVEIPVLQSLNWD----FSTFFKAIIKVVISEEKREQ    126
M_crocodyli                             DIIYPIIFTHLE------THTYDFVEIPILLTKKWD----FTSFFDITINVVISEEKRQK    131
M_leonicaptivi                          TLVFEEIYKHLK------ENKYDFVEIPILFNAPKK----VIKLFNEIFFLNNNFFKIEK    131
M_canis                                 SLVEKFLFYHLK------NSDYHFVEFPTIFKASQK----TLNLFAKIWNVEIDENLRKN    130
M_felis                                 NILYPYLLEKLK------KEKYHFVEIPILNTEVVD----FSILFDEIWNLTICPNFRKE    133
M_buteonis                              SLVFEFLYEHLL------KHKYDFVEIANFHKCEKK----LVGLFSKIIVVETQKEIRQK    131
M_mobile                                NLIEPLHFEHLK------KNKYDFAELALI-TKESN----FRDLFLEIIFLNISENKRKK    131
M_columborale                           ELVHLKVFKHLQ------DNEYDYAEIPVLKNSQVP----FWSLAEQIIVLNLKKPIRIA    130
M_cricetuli                             AIVHKRVYQHLN------KNKYDFIEIAALHSLHSN----FIKFVTKIIFLHTDEKTRLE    130
M_opalescens                            QIIEKIIKEELI------KNNYDFVEIPLLIDKNID----YSDLFSSIIWINNSEKQRQQ    131
M_arginini                              KQVHLEVFKYLS------ENKFDFAEIPALNSKHAN----FCSLISKIYVHKVDENTRIK    130
M_synoviae                              KQVHLEVFKYLS------ENKFDFAEIPALNSKHAN----FCSLISKIYVHKVDENTRIK    130
M_ovipneumoniae                         QKIYPILEEHLR------NNYYHFVEIPNLWTKNAD----FQAFFWKTVWISASRKQLLL    397
M_hyopneumoniae                         KSIYPFLENHLS------KNHYHFVEIPNLWTKNAN----FGKFFSKVVWINTSKKQQLL    396
M_dispar                                KTVYPFLEEHLS------KNHYHFVEIPNLWTENAN----FLQFFSKVVWIKTSKKQQLL    397
M_flocculare                            KAVYGFLENHLT------KNHYHFVEIPNLWTKNAN----FLKFFSKIVWINTSKEQQLL    396
M_bovoculi                              SKVYEILENYLS------QNHFDFVEIPNIDSPNAN----FRKFFSKVILISTSEEQRVI    394
M_conjunctivae                          KKLYKILSNHLK------SRRYDFVEIPNLYTKNAN----FTEFFQKIVLVSTNPEQRQK    395
M_pulmonis                              KLIYPLLKTHLE------QNFYDFVEIPVLGSKNVD----FYPLFSKIYNIEISESQRLE    131
M_hyorhinis                             KLVYPFLEEHLK------NNKYDFVEIPNLYSKNAN----FAVYFDKVIRVFSSEEQRVK    143
M_collis                                KLVYPILEEHLK------NNKYDFVEIANIKGANLD----FSKYFTKIFVLITPEEQRQK    131
M_molare                                KLVYPVLEDHLK------NNKYDFVEIPNLFTQNAN----FAKYFEQIFNIEISEEQRLK    131
M_gallinarum                            KLIYPILKKELE------NGDYDFVEIPKLKSENFD----FSSLFSLILCLSTTAEKHKK    130
M_lipofaciens                           KGIYPLIFEHFQ------SYSYDFVEIPNLVNQKND----FSSFFSSILCLETSNKIRMK    131
M_fermentans                            KIIYPLIFEEIK------DGNFFIVEIPNLLPKNFK----FISLFSAVLCLETSQKNRLK    131
M_bovigenitalium                        KIVYQEIFTTLK------NGKYELAELPNLSNKYCD----FLSLISVVLCLSNNPKKRQK    131
M_californicum                          RVVFPKIFDAIK------NGNFAIVEIPVLVNKNFN----FLSLFSAVLCLSTSEQKRWK    131
```

```
M_simbae                             KLIYPIIYEHLK------SNEYKLAEMPVLVNKNVN----FLPLISGVLCLSTSDEIRVK      131
M_columbinum                         KCIYPIIFDHLN------TYCYDFAEVPNLTTKNGN----FAKLFKIVLCLETSEKNQVK      131
M_iners                              KCIYPILGNALS------SQKFDFVEIPNLDSKNYD----FFSLFSGVLCVSIFEKNRLK      131
M_felifaucium                        KCTYPVLFQYLK------INSFDFVEIPKLISKNFD----FSSLFSGIICLENSASKFLQ      131
M_primatum                           KAIYPILFNKLK------IGKFDFVEIPKIIGKNFD----FSKLFDNIVCLETPDKIRAK      131
M_agalactiae                         KAIFPIVDKAIK------MGKFDFVEIPKLIGKNYD----FSKLFDIILCLETPEKIRGK      131
M_bovis                              KVIFPIVYEAIK------IGKFDFVEIPKLVGKNYD----FSQLFDTILCLESPEKIRAK      131
M_iowae                              SIMFPIMLDKLAKLKKES-KGLVFVELGIYIYNPKY----FKSTFDFIIGINRDDEITEN      146
M_penetrans                          KLMNKVIYDKIFELKKEN--RQIIVELGTYLFFEEY----FKDLFYKVVVVDSSDKNYKK      148
M_genitalium                         LVTKPLLIAWIKSLKTQFQKKLALIEIAVMLN----YWNEYRSLFDYVIKLERDDQLVNL      139
M_pneumoniae                         NLTKPLVQEWLNQLKAQFQDKIALVEIAVMLN----YWNDYRPFFDEVIQIERDAKIVKQ      139
M_gallisepticum                      DFAGKIAKNHLK---NLDYHGLVVVEGAAIYNNQQR----YLDIFDYFVLVERDEKLIQA      142
M_imitans                            NFAEKIVKNHLN---NLNYNGLVVVEGAAIYNAQEK----YLNLFDYFILVKRDNKLIQD      142
M_testudinis                         QCLQPLISMILWSLNVQSNNKVYLVEIAASSFNLEN----YQRFFDASVLIKCDLKTIKK      138
M_alvi                               FILQPLIKSIILSLKNKYKGKILLVESASALNEFNNYYNDYLYLFDKFILINAPLNFIKK      141
M_pirum                              NILEPLIIKIILNIKKKYKNSLVLVEGGAILNNFEK----YANLFDKFILIKAPKKFIKE      142
                                               :              *

Legionella_pneumophila               RIV-KRDHCTKKQALAILATQPNLEQR---LEAADDVLINESGLSELKAKVNKLHQKYLR      195
M_mycoides_subsp_capri               RVL-KRDKTNIRDVLRIRRNQNKSIKR---KK--PDLVISNFYQLEFYIQKNRLFI----      185
M_leachii                            RVL-KRDKTNIRDILRIRRSQNKSIKR---KK--PDLAISDFYQLEFYIQKNRLLE----      185
M_mycoides_subsp_mycoides            RVL-KRDKTNIRDILRIRRSQNKSIKR---KK--PDLAISDFYQLEFYIQKNRLLE----      189
M_capricolum_subsp_capricolum        RVL-KRDKTNIRDILRIRRSQNKSIKR---KK--PDLVISNFYQLEFYIQKNRLL-----      188
M_capricolum_subsp_capripneumoniae   RVL-KRDKTNIRDILRIRRSQNKSIKR---KK--PDLVISNFYQLEFYIQKNRLL-----      184
M_putrefaciens                       RVI-KRDNASLSNVFKIWWKQTKLLRK---TK--YDISIRHFYELEFYLQKNKLIDW---      186
M_yeatsii                            RVK-KRDGVSLFNIFKIWLKQTRLLKK---VK--SDITIKYFYELELYIQKNKIIVD---      187
M_sturni                             WCQ-KRGVDKSTFELINKKNSFKWGKI-AFFKKVPIVNISWINQLNEAKV-EELLQKVK-      187
Ms02                                 IWK-SMSVNNSLILKLVDKNGDFFGKN-RTFANIPIVNISSGNLRWKWYF-RKIKTKYIC      188
M_anatis                             QRK-KRNVDNSFFDALNNKNASFLAKN-TLFMNIDIVNIYEYNHQNTELI-KKFLIDNNI      188
M_alligatoris                        NLK-FRNVNNSLYDELNQKNHTFLNKN-ELFNAIPIVNISYENVKTIQEN-DLFFRHIKH      183
M_crocodyli                          NLS-IRNVDKYQRDFLNQKNKEISKEN-ELFGKINIVNISYDNIETICEN-MEFISLIKQ      188
M_leonicaptivi                       NNVIQERIK----KLLNTLNSFDWDSY-ETFFEIPIYNVVFSKNKNNTKCIKIQIKKRKK      186
M_canis                              YMW-KKYGDNSIIKILDRENSYNWGLK-DQLHNLKIVNISSLNIDILYEN-EKYNCNCSI      187
M_felis                              YIK-TLSLNNPIITILDKKNSYNWKKD-VNFKNLPIVNISRRNRDNIKKL-IKY------      184
M_buteonis                           LCQ-LKGVDNYWFNWFNNQNAFNWSEK-ETFCQIPVVNILINNLEELNNLPNKIVEYYYT      189
M_mobile                             NLIEKRKISLE-----QYLNINKKIADHSEGKHFFNIKFEIDSVKEEDVL-NYLINKSKN      185
M_columborale                        YAL-KRGMDLGRFNQLDTINTLEFLRQ-LPFKSIKQIWLSSSEEIS------DFLAK---      179
M_cricetuli                          NCR-KRNVSDKQMKILDTLFNDHYTNK-NNYQNIQIIQLNLKDINTLKDI-KKILKYNVF      187
M_opalescens                         NII-KALKKPETVKIMDLKNDPKEIFK-KLKGEKNLIKITTDNIKLTLQS-IGTDTFYT-      187
M_arginini                           FCK-KRNVDSEALKILDALNSYNWTDL-KNYNGILVVDKL--------------------      168
M_synoviae                           FCK-KRNVDSEALKILDALNSYNWTDL-KNYNGILVVDKL--------------------      168
M_ovipneumoniae                      NIK-AKKVKKEVWQKNRALNGN--------KIKFYNVKISNSRWKRPSFL-PKFFTKIFK      447
M_hyopneumoniae                      NIK-RKKVKNDIKLKNQALNTG--------KIQFYDVKISNRKWKKPGFF-LKFFSKIFK      446
M_dispar                             NIE-NKKVKNSVSHKNQALNSN--------KIKFYDVKISHRKWKKRHFF-TKFFHKIFK      447
M_flocculare                         NIK-NKKVKKSVWMKNQALNSN--------KIKFYDVKISSQKWKKRRFF-PKFFHKIFK      446
M_bovoculi                           NIG-KKNVENLAAKLNNSLNKK--------EIANFDVEIKNEEWKKDGFF-LTFFQNIFN      444
M_conjunctivae                       NIL-NKNVALNVSQKNEQLNQI--------VYKNIDFEVHGDEWKNPEFF-NHFFNEIFK      445
M_pulmonis                           QLK-KRGVDNSDISFFESINKG--------VIGKKVVNISLENLEKIEKF----------      172
M_hyorhinis                          NIV-NKNVNNSISSLNDFLNKG--------FCHKVDVNISNSEWKNEDFF-PKFFSELNK      193
M_collis                             NIN-SKSVDKKIQNINNSLNSG--------IVGVPTVNIMWKDVDKENFF-VTFFKKAFD      181
M_molare                             NIE-KKYVDKKTSKLNNKLNNG--------KKGKKVVNIMWEDTHKKDFF-IDFLKSLKL      181
M_gallinarum                         NLK-KRNVDNSKINAINEKNAPKSIIN-ALFTKIPIVNIYGNKLWKYAKN-KKFLQFLFS      187
M_lipofaciens                        NAQ-KRNVDKLFIQEIDRKNDPKTIKN-LLFGKIPIVDIYANNFTDIEKM-QKIFQLLLP      188
M_fermentans                         NFK-KRNVDKTKIKAIDEKNDPFSIKN-QLFGSIPIVDFYGHNFTNNENI-LDFLKLLFF      188
M_bovigenitalium                     NLV-KRNVDKSTILLIDAKNDPKSIKK-SLFSLKPVVDIYINNCERLEQN-QKILKLVKS      188
M_californicum                       NIQ-KRSVDKLTIKAIDQKNSTILAKN-QLFGQIPVVDIYLENFESYDRN-KKILDLVKL      188
M_simbae                             NLQ-KRNVDKATLKQLDKKNDTKKAIK-SLFGKKLIVEMYGDNFESTDQN-KKILSLLN-      187
M_columbinum                         NLK-KRNVNKLTIRLLNAKNDPKLIKN-QLFKEKIIVDIYGQNFKNWARN-KKFLECLFS      188
M_iners                              NLY-KKGVDKFTIKWINQKNDPKRIKK-ALFGKKPIVDIYAYNISTILRN-QKKMDYLIS      188
M_felifaucium                        NYI-KRSVDNFLKNKISEKNDPFYIKN-LLFNKIPIVDIYEYNPENDKI-----IDVFLQ      184
M_primatum                           NLE-KRNVDNFTKKAISAQNAPFLIKN-ALFGTIPIVNIYANNFNNDVL-----FELILK      184
M_agalactiae                         NMT-KRGVDNLTKMAISEKNAPKLMKN-AIFGQIPIVNIFANNLCDDAL-----FELILT      184
M_bovis                              NMA-ARGVDNFTKKAISEKNAPKLMKN-TIFGQVPIVNIFANNLCDNAL-----FELILT      184
M_iowae                              NPF-EKIKNVIKFSTKDVENSKNIDNT-KTILVDFIVDN-NSSLKDFENNIKKILEYLDK      203
M_penetrans                          NNF-KKFSNIEKFSTKPVGNLENPQKE-GVFYTDFLVGN-CGNLFDLESKAKDFLKVLTH      205
M_genitalium                         ALQ-QRNSHKKVKDLIKE-PN---C-------KIDTIFN-NDSIATAALKLIKLLETFLE      186
M_pneumoniae                         ALK-ARGVDVEQVQKLIADPT---Y-------PILTVIN-NSTVAECALHVTQFLESIAK      187
M_gallisepticum                      SIL-QKFAYLKDFDLKKWNPI---KEN-KEFKADLVIQN-NGEIETAYQELLKFLKKISS      196
M_imitans                            SIM-QKFAYLKDFDLKKWNPI---KEN-TNFKADICIEN-NGDINDAYKELKKFLEKISG      196
M_testudinis                         RLT-QRFSHLSTQSQESLIYK---FK---EKDFQYVLCN-NYSIDTAANELLSYLKTIKV      190
M_alvi                               NNL-NKFKYLKKDIVDLFDLD---VN---LHNFDLVVQN-NTTPEEAAKKIKEFLIKLDK      193
M_pirum                              NNN-IKFAHIDNDIYKYLKVN---IS---NSFFDLIILN-NKSPELCAKKIEIFLNKLKK      194

Legionella_pneumophila               EAKIKQ-----------  201
M_mycoides_subsp_capri               -----------------  185
M_leachii                            -----------------  185
M_mycoides_subsp_mycoides            -----------------  189
M_capricolum_subsp_capricolum        -----------------  188
M_capricolum_subsp_capripneumoniae   -----------------  184
M_putrefaciens                       -----------------  186
M_yeatsii                            -----------------  187
M_sturni                             -----------------  187
Ms02                                 PNLK-------------  192
M_anatis                             LN---------------  190
M_alligatoris                        YL---------------  185
M_crocodyli                          YL---------------  190
M_leonicaptivi                       Y----------------  187
M_canis                              KI---------------  189
M_felis                              -----------------  184
M_buteonis                           -----------------  189
M_mobile                             SN---------------  187
M_columborale                        LQPKNS-----------  185
M_cricetuli                          NSKK-------------  191
M_opalescens                         -----------------  187
M_arginini                           -----------------  168
M_synoviae                           -----------------  168
M_ovipneumoniae                      -----------------  447
M_hyopneumoniae                      -----------------  446
M_dispar                             -----------------  447
M_flocculare                         E----------------  447
```

…Motif 4

```
M_mobile                                          IQYYEEHGVECFVVYNKSNSEEFFPENYQIDY----------------------  145
M_iowae                                           YDSYK-------EINHNFETILLISDYEFRKLSSTRILRSLKNEK---------  144
M_leonicaptivi                                    AKINK-------EINPNIETILFLADEEYLNISSTNIKNKE-------------  137
M_canis                                           ASINK-------SINKKLETILIIPDNQFLKYSSTEIRKIKEKNV---------  143
M_pulmonis                                        AAGNK-------KINNEVETILIFPDYDKIEINSTLIRHQKFIENLKK------  149
M_felis                                           ASGNK-------LLNHNLETILIIPDQEYKTYSSTLERTKNV------------  137
M_molare                                          AVGNK-------AINNSLETILFFPDENNIKISSTLERHKNFYKNKGK------  143
M_buteonis                                        AYANK-------HLNKDLETVFFLTDFQYQGYRSILIRHQEKIKESK-------  148
M_iners                                           AAANK-------SLNSSLETILIFPNYKDINYSSTLERHLKKIRRLDVKVS---  147
M_alligatoris                                     AAGNK-------HLNEDLETVLLVPDHQFINFSSTLIRHKEKLNK---------  145
M_crocodyli                                       AAGNK-------HVNNDLETVILIPDYEYIEYSSTLERHKEKLGK---------  146
M_bovigenitalium                                  AAGNN-------QVYPGLETILIIPDYEMIGISSTLIRHKKALEK---------  140
M_californicum                                    AAGNH-------QVNPDLETILIIPDYEMIGISSTLIRHKKALDKNNV------  143
M_simbae                                          AAGNH-------EVNPDLETILIIPDYDMIGISSTLIRHKKALIKEN-------  142
M_conjunctivae                                    AAGNH-------QLNNDLETILFFPDYELIEYSSTLERHKKFYK----------  148
M_gallinaceum                                     AAGNN-------SLNNELETVLLIPSYEYIKYSSTLIRHKEKMKKDV-------  145
M_felifaucium                                     AAGNN-------LLNPELETVLIVPDYDKIEYSSTLVRHMEKLKKCENS-----  146
M_sturni                                          AAGNK-------KINKNLETILIMPNYRSINYSSTLIRHMKKLKQNV-------  144
M_columborale                                     AAGNK-------HLNKHLETILIMPNYKSIKYSSTLIRHKERLKI---------  142
M_cricetuli                                       AAGNK-------HLNKNLETILIMPNYQHINYSSTLMRHKKRLNK---------  142
M_anatis                                          AAGNK-------TLNNELETILIMPNYEDINYSSTLERHKEKLKNV--------  143
M_gallinarum                                      ASGHH-------TINPDLETILIIPDYEMLGVSSTLERHLKSLKK---------  141
M_synoviae                                        AAGNK-------FLNSNLETILIMPDYENINYSSTLLRHGKKLNKNSV------  143
M_agalactiae                                      AAGHN-------SMNKDLETILIMPDYDMIEYSSTVIRHKNKLGK---------  140
M_bovis                                           AAGHN-------SINNDLETILIMPDYNMIEYSSTVIRHKQKLGK---------  140
M_fermentans                                      AAANH-------HLNSELETILIVPDYENIDYSSTLLRHKQKLGKK--------  142
M_opalescens                                      AAGNK-------LINNDLETVLIIPDYSMLGVSSTLERHKSKLQKEKNV-----  145
M_lipofaciens                                     AAGSH-------SINNKIETILIIPDYEMINYSSTLIRHKNKLGR---------  140
M_columbinum                                      AAGNH-------EVNNELETILIIPDYKLIEYSSTLLRHKKKLGI---------  142
M_primatum                                        AAGNH-------CVNNQLETVLIMPDYSMINYSSTLLRHKKKLGK---------  140
M_penetrans                                       ADIYK-------QEWDKLEVVYFFSEKKLENISSRKIINLNKGKNDYEN-----  150
M_putrefaciens                                    YDGFK-------SLDPNIEVIYFISSSQTRALSSSTIKEIEFFKKD--------  141
M_yeatsii                                         YDGFK-------SLDPNIEIVYFISDVEKRQLSSSAIKEIQFYKN---------  140
M_feriruminatoris                                 YDGFK-------SLDPNIEVVYFVSDADKRSLSSTILREIEFYKN---------  140
M_mycoides_subsp_mycoides_SC_str_PG1              YDGFK-------SLYPNIEVIYFISDYDKRSLSSTILREIEFYKK---------  140
M_leachii                                         YDGFK-------SLDPNIEVVYFISDYDKRSLSSTILREIEFYKK---------  140
M_mycoides_subsp_capri_LC_str_95010               YDGFK-------SLDPNIEVVYFISDYDKRSLSSTILREIEFYKK---------  140
M_capricolum                                      YDGFK-------SLDPNIEVVYFISDYDKRSLSSTILREIEFYKK---------  140
Candidatus_M_girerdii                             ASANY-------YLDKTIETILFAAEKDLKSKSSSSIKKMQQEIAILKNKK---  147
M_testudinis                                      ASVNK-------KLNPKIETVFFLSDQAYQSVSSTAIKKVIDNHKKYYQAKKTK  151
M_alvi                                            ASINK-------KLDKKLETIVFFSDVAYQSISSSDIKKTLKKHNKFFKSNKN-  151
M_pirum                                           ASVNK-------ELNPDLETIVLFSDNVYKSISSSEIKKTILKHKNFFKQKTI-  151
                                                                   :   :  .
```
