# Supplementary material for: Evaluating the Genetic Capacity of Mycoplasmas for Coenzyme A Biosynthesis in a Search for New Anti-mycoplasma Targets
Source: Front Microbiol. 2021 Dec 20;12:791756. doi: 10.3389/fmicb.2021.791756 (PMC8721197; doi:10.3389/fmicb.2021.791756)
Supplement: Supplementary file 2 [file Data_Sheet_2.pdf]

Supplementary Tables

Evaluating the genetic capacity of Mycoplasmas for coenzyme A biosynthesis in a search for new anti-mycoplasma targets

Tertius Alwyn Ras[1], Erick Strauss[1], Annelise Botes[1*]

[1]Department of Biochemistry, Stellenbosch University, Stellenbosch, South Africa.

Supplementary Table 1 NCBI accession numbers for the nucleotide sequences of *Mycoplasma* genomes analysed and the genomic location of their annotated CoA biosynthetic genes

Mycoplasma species	NCBI accession number	Genomic location			
		**PanK type III**	**CoaBC**	**PPAT**	**DPCK**
M. agalactiae PG2	NC_009497.1^			161810-162232<br>(locus tag: MAG_RS00720)	complement (784004-784576)<br>(locus tag: MAG_RS03375)
M. alligatoris A21JP2	NZ_ADNC00000000.1*	37520-38281<br>(locus tag: MALL_RS02820)<br>(contig: NZ_ADNC01000027.1)		complement (40704-41141)<br>(locus tag: MALL_RS00640)<br>(contig: NZ_ADNC01000004.1)	10188-10745<br>(locus tag: MALL_RS04280)<br>(contig: NZ_ADNC01000022.1)
M. alvi ATCC 29626	NZ_JNJU00000000.1*	complement (52254-53027)<br>(locus tag: T383_RS0101300)<br>(contig: NZ_JNJU01000002.1)		complement (50239-50694)<br>(locus tag: T383_RS0101285)<br>(contig: NZ_JNJU01000002.1)	138685-139275<br>(locus tag: T383_RS0100510)<br>(contig: NZ_JNJU01000001.1)
M. anatis 1340	NZ_AFVJ00000000.1*	7192-7998<br>(locus tag: GIG_RS00500)<br>(contig: NZ_AFVJ01000007.1)	6078-7205<br>(locus tag: GIG_RS00495)<br>(contig: NZ_AFVJ01000007.1)	21008-21439<br>(locus tag: GIG_RS03275)<br>(contig: NZ_AFVJ01000035.1)	complement (15831-16403)<br>(locus tag: GIG_RS04180)<br>(contig: NZ_AFVJ01000038.1)
M. arginini ATCC 23838	NZ_AUAH00000000.1*	28237-28989<br>(locus tag: F805_RS0103520)<br>(contig: NZ_KE386772.1)	27107-28261<br>(locus tag: F805_RS0103515)<br>(contig: NZ_KE386772.1)	18799-19230<br>(locus tag: F805_RS0101715)<br>(contig: NZ_AUAH01000004.1)	complement (65779-66285)<br>(locus tag: F805_RS0100895)<br>(contig: NZ_AUAH01000002.1)
M. bovigenitalium 51080	NZ_AP017902.1^			133462-133884<br>(locus tag: MBVG596_RS00500)	complement (403329-403901)<br>(locus tag: MBVG596_RS01755)
M. bovis 08M	NZ_CP019639.1^			176421-176843<br>(locus tag: B0W43_RS00780)	complement (910577-911149)<br>(locus tag: B0W43_RS03820)
M. bovoculi M165/69	NZ_CP007154.1^				467626-468960<br>(locus tag: MYB_RS01885)
M. buteonis ATCC 51371	NZ_JPOK00000000.1*	complement (27614-28354)<br>(locus tag: EI91_RS01795)<br>(contig: NZ_JPOK01000006.1)		complement (32640-33086)<br>(locus tag: EI91_RS02235)<br>(contig: NZ_JPOK01000007.1)	complement (146386-146955)<br>(locus tag: EI91_RS00590)<br>(contig: NZ_JPOK01000004.1)
M. californicum ST-6	NZ_CP007521.1^			complement (380505-380936)<br>(locus tag: MCFN_RS01545)	complement (81250-81822)<br>(locus tag: MCFN_RS00370)
M. canis UF33	NZ_AJFS00000000.1*			complement (30033-30464)<br>(locus tag: MCANUF33_RS00905)<br>(contig: NZ_AJFS01000003.1)	complement (270341-270910)<br>(locus tag: MCANUF33_RS02435)<br>(contig: NZ_AJFS01000004.1)
M. capricolum subsp. capricolum ATCC 27343	NC_007633.1^			281447-281869<br>(locus tag: MCAP_RS01170)	68116-68670<br>(locus tag: MCAP_RS00290)
M. capricolum subsp. capripneumoniae	NZ_CP019061.1^			309851-310272<br>(locus tag: BVA24_RS01255)	83588-84141<br>(locus tag: BVA24_RS00310)
M. collis ATCC 35278	NZ_JNJY00000000.1*			13860-14303<br>(locus tag: T403_RS0103270)<br>(contig: NZ_JNJY01000016.1)	complement (93474-94022)<br>(locus tag: T403_RS0100515)<br>(contig: NZ_JNJY01000001.1)
M. columbinum ATCC 29257	NZ_JONY00000000.1*			complement (198287-198715)<br>(locus tag: T406_RS0100840)	295043-295615<br>(locus tag: T406_RS0101290)

Mycoplasma species	NCBI accession number	Genomic location			
		PanK type III	CoaBC	PPAT	DPCK
				(contig: NZ_JONY01000003.1)	(contig: NZ_JONY01000003.1)
M. columborale ATCC 29258	NZ_JNJZ00000000.1*	complement (1358-2179) (locus tag: T404_RS02275) (contig: NZ_JNJZ01000008.1)	complement (2167-3300) (locus tag: T404_RS02280) (contig: NZ_JNJZ01000008.1)	44390-44818 (locus tag: T404_RS02420) (contig: NZ_JNJZ01000008.1)	78644-79201 (locus tag: T404_RS01040) (contig: NZ_JNJZ01000004.1)
M. conjunctivae HRC/581T	NC_012806.1^			90586-91032 (locus tag: MCJ_RS00410)	697230-698567 (locus tag: MCJ_RS02820)
M. cricetuli ATCC 35279	NZ_JAHB00000000.1*	complement (56507-57325) (locus tag: U744_RS0100350) (contig: NZ_JAHB01000001.1)	complement (57313-58446) (locus tag: U744_RS0100355) (contig: NZ_JAHB01000001.1)	complement (45679-46107) (locus tag: U744_RS0100285) (contig: NZ_JAHB01000001.1)	complement (32973-33548) (locus tag: U744_RS0102240) (contig: NZ_JAHB01000006.1)
M. crocodyli MP145	NC_014014.1^	complement (29628-30404) (locus tag: MCRO_RS00130)		complement (624066-624506) (locus tag: MCRO_RS02700)	658005-658577 (locus tag: MCRO_RS02845)
M. dispar ATCC 27140	NZ_CP007229.1^				complement (504206-505549) (locus tag: MDIS_RS01920)
M. felifaucium ATCC 43428	NZ_JHXS00000000.1*			complement (20875-21315) (locus tag: T390_RS0102395) (contig: NZ_JHXS01000011.1)	4781-5353 (locus tag: T390_RS0100040) (contig: NZ_JHXS01000001.1)
M. felis ATCC 23391	NZ_JNKA00000000.1*			complement (7696-8109) (locus tag: T405_RS01240) (contig: NZ_JNKA01000007.1)	complement (22907-23461) (locus tag: T405_RS01860) (contig: NZ_JNKA01000013.1)
M. fermentans PG18	NC_021002.1*			complement (240477-240905) (locus tag: MBIO_RS01255)	136897-137469 (locus tag: MBIO_RS00730)
M. flocculare ATCC 27399	NZ_CP007585.1^				complement (336307-337650) (locus tag: MYF_RS01250)
M. gallinaceum B2096 8B	CP011021.1^	755465-756211 (locus tag: VO56_02800)	754326-755483 (locus tag: VO56_02795)	585022-585459 (locus tag: VO56_02005)	complement (322424—323005) overlaps with a disrupted hypothetical protein (322682..323005)
M. gallinarum Mgn_IPT	NZ_LVLH00000000.1*			complement (52410-52835) (locus tag: MGALLINA_RS01585) (contig: NZ_LVLH01000028.1)	28062-28631 (locus tag: MGALLINA_RS01505) (contig: NZ_LVLH01000028.1)
M. gallisepticum str. R(low)	NC_004829.2^				346439-347041 (locus tag: MGA_RS01410)
M. genitalium G37	NC_000908.2^				321046-321642 (locus tag: MG_RS01570)
M. hyopneumoniae J	NC_007295.1^				complement (420694-422034) (locus tag: MHJ_RS01875)
M. hyorhinis HUB-1	NC_014448.1^				775007-775594 (locus tag: MHR_RS03640)
M. imitans ATCC 51306	NZ_JADI00000000.1*				16155-16745 (locus tag: P690_RS0100995) (contig: NZ_JADI01000008.1)

<table>
<tr><th rowspan="2">Mycoplasma species</th><th rowspan="2">NCBI accession number</th><th colspan="5">Genomic location</th></tr>
<tr><th>PanK type III</th><th colspan="2">CoaBC</th><th>PPAT</th><th>DPCK</th></tr>
<tr><td>M. iners ATCC 19705</td><td>NZ_JNJW00000000.1*</td><td></td><td colspan="2"></td><td>complement (3298-3741)
(locus tag: T395_RS0102600)
(contig: NZ_JNJW01000012.1)</td><td>complement (13826-14398)
(locus tag: T395_RS0102195)
(contig: NZ_JNJW01000007.1)</td></tr>
<tr><td rowspan="2">M. iowae 695</td><td rowspan="2">CP033512^</td><td rowspan="2">Complement (670158-670934)
(locus tag: EER00_02695)</td><td>PPCS</td><td>PPCDC</td><td rowspan="2">910601-911035
(locus tag: EER00_03620)</td><td rowspan="2">958358-958969
(locus tag: EER00_03920)</td></tr>
<tr><td>complement
(753430-754140)
(locus tag:
EER00_03025)</td><td>complement
(752889-753425)
(locus tag:
EER00_03020)</td></tr>
<tr><td>M. leachii PG50</td><td>NC_014751.1^</td><td></td><td colspan="2"></td><td>329605-330027
(locus tag: MSB_RS01360)</td><td>109349-109906
(locus tag: MSB_RS00465)</td></tr>
<tr><td>M. leonicaptivi ATCC 49890</td><td>NZ_JHWE00000000.1*</td><td></td><td colspan="2"></td><td>2568-2981
(locus tag: BU00_RS0101800)
(contig: NZ_JHWE01000024.1)</td><td>complement (7331-7894)
(locus tag: BU00_RS04890)
(contig: NZ_JHWE01000024.1)</td></tr>
<tr><td>M. lipofaciens ATCC 35015</td><td>NZ_JMKY00000000.1*</td><td></td><td colspan="2"></td><td>complement (123928-124350)
(locus tag: T388_RS0100600)
(contig: NZ_JMKY01000001.1)</td><td>66707-67279
(locus tag: T388_RS0102550)
(contig: NZ_JMKY01000006.1)</td></tr>
<tr><td>M. mobile 163K</td><td>NC_006908.1^</td><td>complement (725838-726569)
(locus tag: MMOB_RS03205)</td><td colspan="2">complement (726554-727636)
(locus tag: MMOB_RS03210)</td><td>712814-713251
(locus tag: MMOB_RS03530)</td><td>713315-713878
(locus tag: MMOB_RS03135)</td></tr>
<tr><td>M. molare ATCC 27746</td><td>NZ_JHWG00000000.1*</td><td>80872-81600
(locus tag: BU19_RS0101285)
(contig: NZ_JHWG01000002.1)</td><td colspan="2"></td><td>20255-20686
(locus tag: BU19_RS0102135)
(contig: NZ_JHWG01000009.1)</td><td>complement (101826-102374)
(locus tag: BU19_RS0100565)
(contig: NZ_JHWG01000001.1)</td></tr>
<tr><td>M. mycoides subsp. capri LC str. 95010</td><td>NC_015431.1^</td><td></td><td colspan="2"></td><td>complement (349967-350389)
(locus tag: MLC_RS01385)</td><td>128078-128647
(locus tag: MLC_RS00500)</td></tr>
<tr><td>M. mycoides subsp. mycoides SC str. PG1</td><td>NC_005364.2^</td><td></td><td colspan="2"></td><td>312742-313164
(locus tag: MSC_0272)</td><td>114548-115117
(locus tag: MSC_0096)</td></tr>
<tr><td>M. opalescens ATCC 27921</td><td>NZ_JOOB00000000.1*</td><td></td><td colspan="2"></td><td>21433-21870
(locus tag: T385_RS0103170)
(contig: NZ_KL544020.1)</td><td>108837-109400
(locus tag: T385_RS0102295)
(contig: NZ_KL544019.1)</td></tr>
<tr><td>M. ovipneumoniae SC01</td><td>NZ_AFHO00000000.1*</td><td></td><td colspan="2"></td><td></td><td>complement (11215-12558)
(locus tag: MOSC01_RS0103605)
(contig: NZ_AFHO01000022.1)</td></tr>
<tr><td>M. penetrans HF-2</td><td>NC_004432.1^</td><td>complement (1332546-1333313)
(locus tag: MYPE_RS05220)</td><td colspan="2"></td><td>complement (1285856-1286308)
(locus tag: MYPE_RS04950)</td><td>253894-254514
(locus tag: MYPE_RS05360)</td></tr>
<tr><td>M. pirum MPI25960</td><td>NZ_AZHZ00000000.1*</td><td>complement (179214-179984)
(locus tag: X558_RS0103155)
(contig: NZ_KK365982.1)</td><td colspan="2"></td><td>complement (175096-175551)
(locus tag: X558_RS0103135)
(contig: NZ_KK365982.1)</td><td>171820-172404
(locus tag: X558_RS04315)
(contig: NZ_KK365981.1)</td></tr>
<tr><td>M. pneumoniae M129</td><td>NC_000912.1^</td><td></td><td colspan="2"></td><td></td><td>458998-459600</td></tr>
</table>

Mycoplasma species	NCBI accession number	Genomic location			
		**PanK type III**	**CoaBC**	**PPAT**	**DPCK**
					(locus tag: MPN382)
M. primatum ATCC 25948	NZ_JNJV00000000.1*			complement (4243-4665)<br>(locus tag: T386_RS0103800)<br>(contig: NZ_JNJV01000038.1)	23196-23768<br>(locus tag: T386_RS0100120)<br>(contig: NZ_JNJV01000001.1)
M. pulmonis UAB CTIP	NC_002771.1^	complement (570749-571423)<br>(locus tag: MYPU_RS02370)		102808-103257<br>(locus tag: MYPU_RS00470)	882270-882788<br>(locus tag: MYPU_RS03650)
M. putrefaciens KS1	NC_015946.1^			complement (615746-616171)<br>(locus tag: MPUT_RS02695)	503051-503611<br>(locus tag: MPUT_RS02170)
M. simbae ATCC 49888	NZ_JNJG00000000.1*			23328-23756<br>(locus tag: T329_RS0101755)<br>(contig: NZ_JNJG01000006.1)	complement (125082-125645)<br>(locus tag: T329_RS0101475)<br>(contig: NZ_JNJG01000005.1)
M. sturni DSM 22021	NZ_JNIZ00000000.1*	242058-242879<br>(locus tag: Q349_RS01120)<br>(contig: NZ_JNIZ01000003.1)	240940-242070<br>(locus tag: Q349_RS01115)<br>(contig: NZ_JNIZ01000003.1)	complement (280184-280618)<br>(locus tag: Q349_RS01320)<br>(contig: NZ_JNIZ01000003.1)	complement (143690-144253)<br>(locus tag: Q349_RS02155)<br>(contig: NZ_KL370785.1)
M. synoviae 53	NC_007294.1^	complement (8415-9167)<br>(locus tag: MS53_RS00030)	complement (9143-10297)<br>(locus tag: MS53_RS00035)	complement (756186-756617)<br>(locus tag: MS53_RS03405)	complement (622193-622699)<br>(locus tag: MS53_RS02725)
M. testudinis ATCC 43263	NZ_JHXT00000000.1*	1820-2566<br>(locus tag: T384_RS0104425)<br>(contig: NZ_JHXT01000017.1)	684-1826<br>(locus tag: T384_RS0104420)<br>(contig: NZ_JHXT01000017.1)	42922-43377<br>(locus tag: T384_RS06450)<br>(contig: NZ_JHXT01000013.1)	13100-13717<br>(locus tag: T384_RS0102290)<br>(contig: NZ_JHXT01000007.1)
M. yeatsii GM274B	NZ_CP007520.1^			complement (512943-513365)<br>(locus tag: MYE_RS02185)	complement (301476-302039)<br>(locus tag: MYE_RS01275)
Mycoplasma sp. Ms02	CP081302^				858351-858929<br>(locus tag: K4L35_03330))

^Complete genome (deposited to NCBI)
*Incomplete genome (deposited to NCBI)

Supplementary Table 2 NCBI accession numbers for 16S rRNA gene sequences and protein sequences of the CoA biosynthesis pathway enzymes

16S rRNA gene		PanK Type III protein		CoaBC protein		PPAT protein		DPCK protein	
***Mycoplasma* species**	**NCBI accession number**	***Mycoplasma* species**	**NCBI accession number**	***Mycoplasma* species**	**NCBI accession number**	***Mycoplasma* species**	**NCBI accession number**	***Mycoplasma* species**	**NCBI accession number**
M. agalactiae	NR_118811.1					*M. agalactiae* PG2	WP_011949321.1	*M. agalactiae* PG2	WP_011949819.1
M. alligatoris	NR_041844.1	*M. alligatoris* A21JP2	WP_005683755.1			*M. alligatoris* A21JP2	WP_005683206.1	*M. alligatoris* A21JP2	WP_005683611.1[a]
M. alvi	NR_025985.1	*M. alvi* ATCC 29626	WP_033159884.1			*M. alvi* ATCC 29626	WP_033159881.1	*M. alvi* ATCC 29626	WP_033159717.1
M. anatis	NR_113689.1	*M. anatis* 1340	WP_006886261.1	*M. anatis* 1340	WP_006886260.1	*M. anatis* 1340	WP_040544544.1	*M. anatis* 1340	WP_006886900.1
M. arginini	NR_041743.1	*M. arginini* ATCC 23838	WP_020003050.1	*M. arginini* ATCC 23838	WP_020003049.1	*M. arginini* ATCC 23838	WP_011283787.1	*M. arginini* ATCC 23838	WP_020002982.1
M. arthritidis	NR_113688.1								
M. bovigenitalium	NR_113690.1					*M. bovigenitalium* 51080	WP_004419352.1	*M. bovigenitalium* 51080	WP_004420621.1
M. bovis	NR_102850.1					*M. bovis* 08M	WP_013456618.1	*M. bovis* 08M	WP_014829984.1
M. bovoculi	NR_121731.1							*M. bovoculi* M165/69	WP_022935460.1 (HAD)[c]
M. buteonis	NR_025177.1	*M. buteonis* ATCC 51371	WP_036452417.1			*M. buteonis* ATCC 51371	WP_036452642.1	*M. buteonis* ATCC 51371	WP_036451916.1[a]
M. californicum	NR_029166.1					*M. californicum* ST-6	WP_038561568.1	*M. californicum* ST-6	WP_038561049.1
M. canadense	NR_025988.1							*M. bovigenitalium* 51080	WP_004420621.1
M. canis	NR_113676.1					*M. canis* UF33	WP_004796179.1	*M. canis* UF33	WP_004796406.1[a]
M. capricolum subsp. *capricolum*	NR_074664.1					*M. capricolum*	WP_011387120.1	*M. capricolum subsp. capricolum* ATCC 27343	ABC01831.1
M. capricolum subsp. *capripneumoniae*	NR_118795.1							*M. capricolum subsp. capripneumoniae*	WP_045076023.1
M. collis	NR_114636.1					*M. collis* ATCC 35278	WP_033161390.1	*M. collis* ATCC 35278	WP_033160863.1
M. columbinum	NR_025063.1					*M. columbinum* ATCC 29257	WP_006608789.1	*M. columbinum* ATCC 29257	WP_029891912.1
M. columborale	NR_025179.1	*M. columborale* ATCC 29258	WP_036434680.1	*M. columborale* ATCC 29258	WP_036434682.1	*M. columborale* ATCC 29258	WP_036434731.1	*M. columborale* ATCC 29258	WP_036434144.1
M. conjunctivae	NR_044781.1					*M. conjunctivae* HRC/581T	WP_041594472.1	*M. conjunctivae* HRC/581T	WP_012751785.1 (HAD)[c]
M. cricetuli	NR_025180.1	*M. cricetuli* ATCC 35279	WP_025755140.1	*M. cricetuli* ATCC 35279	WP_025755141.1	*M. cricetuli* ATCC 35279	WP_025755127.1	*M. cricetuli* ATCC 35279	WP_025755469.1[a]
M. crocodyli	NR_074301.1	*M. crocodyli* MP145	WP_013054512.1			*M. crocodyli* MP145	WP_013054470.1	*M. crocodyli* MP145	WP_013054324.1

16S rRNA gene		PanK Type III protein		CoaBC protein		PPAT protein		DPCK protein	
***Mycoplasma* species**	**NCBI accession number**	***Mycoplasma* species**	**NCBI accession number**	***Mycoplasma* species**	**NCBI accession number**	***Mycoplasma* species**	**NCBI accession number**	***Mycoplasma* species**	**NCBI accession number**
M. dispar	NR_025182.1							*M. dispar* ATCC 27140	WP_044635407.1 (HAD)[c]
M. felifaucium	NR_025963.1					*M. felifaucium* ATCC 43428	WP_027334914.1	*M. felifaucium* ATCC 43428	WP_027334523.1
M. felis	NR_029174.1					*M. felis* ATCC 23391	WP_036430302.1	*M. felis* ATCC 23391	WP_036430513.1
M. fermentans	NR_113683.1					*M. fermentans* PG18	WP_013526711.1	*M. fermentans* PG18	WP_013354344.1
M. flocculare	NR_036954.1							*M. flocculare* ATCC 27399	WP_002557681.1 (HAD)[ac]
M. gallinaceum	NR_025913.1	*M. gallinaceum* B2096 8B	AKA50150.1[a]	*M. gallinaceum* B2096 8B	AKA50149.1[a]	*M. gallinaceum* B2096 8B	AKA50018.1	*M. gallinaceum* B2096 8B	Incorrect ORF (see Supplementary Table 1)[a]
M. gallinarum	NR_113687.1					*M. gallinarum* Mgn_IPT	WP_063626090.1	*M. gallinarum* Mgn_IPT	WP_063626074.1
M. gallisepticum	NR_104952.1							*M. gallisepticum* str. R(low)	WP_011113477.1
M. genitalium	NR_074611.1							*M. genitalium* G37	WP_009885895.1
M. haemocanis	AY529641.1								
M. hominis	NR_041881.1								
M. hyopneumoniae	NR_121689.1							*M. hyopneumoniae* J	WP_011284119.1 (HAD)[c]
M. hyorhinis	NR_041845.1							*M. hyorhinis* HUB-1	WP_041363606.1
M. imitans	NR_025912.1							*M. imitans* ATCC 51306	WP_027121942.1
M. iners	NR_025064.1					*M. iners* ATCC 19705	WP_029512930.1	*M. iners* ATCC 19705	WP_029512864.1
M. iowae	NR_044669.2	*M. iowae* 695	QHG89784	*M. iowae* 695	QHG89842 (PPCS)<br>QHG89841.1 (PPCDC)	*M. iowae* 695	QHG89956	*M. iowae* 695	QHG90013
M. leachii	NR_044773.1					*M. leachii* PG50	WP_013447590.1	*M. leachii* PG50	WP_013447415.1
M. leonicaptivi	NR_025965.1					*M. leonicaptivi* ATCC 49890	WP_027121244.1	*M. leonicaptivi* ATCC 49890	WP_051521842.1
M. lipofaciens	NR_025065.1					*M. lipofaciens* ATCC 35015	WP_027120410.1	*M. lipofaciens* ATCC 35015	WP_027120753.1
M. mobile	NR_074620.1	*M. mobile* 163K	WP_011265111.1	*M. mobile* 163K	WP_011265112.1	*M. mobile* 163K	WP_011265096.1[b]	*M. mobile* 163K	WP_011265097.1
M. molare	NR_114637.1	*M. molare* ATCC 27746	WP_027123244.1			*M. molare* ATCC 27746	WP_027123383.1	*M. molare* ATCC 27746	WP_027123123.1

16S rRNA gene		PanK Type III protein		CoaBC protein		PPAT protein		DPCK protein	
***Mycoplasma* species**	**NCBI accession number**	***Mycoplasma* species**	**NCBI accession number**	***Mycoplasma* species**	**NCBI accession number**	***Mycoplasma* species**	**NCBI accession number**	***Mycoplasma* species**	**NCBI accession number**
M. mycoides subsp. *capri* LC	NR_118794.1					*M. mycoides* subsp. *capri* LC str. 95010	WP_013729412.1	*M. mycoides* subsp. *capri* LC str. 95010	WP_013729251.1
M. mycoides subsp. *mycoides* SC	NR_074703.1					*M. mycoides* subsp. *mycoides* SC str. PG1	NP_975271.1	*M. mycoides* subsp. *mycoides* SC str. PG1	NP_975106.1
M. opalescens	NR_025067.1					*M. opalescens* ATCC 27921	WP_029906491.1	*M. opalescens* ATCC 27921	WP_029906184.1
M. ovipneumoniae	NR_025989.1							*M. ovipneumoniae* SC01	WP_010321428.1 (HAD)[c]
M. ovis	AF338268.1								
M. parvum	AB610850.1								
M. penetrans	NR_118664.1	*M. penetrans* HF-2	WP_011077834.1			*M. penetrans* HF-2	WP_011077780.1	*M. penetrans* HF-2	WP_011077028.1
M. pirum	NR_029165.1	*M. pirum* MPI25960	WP_027124235.1			*M. pirum* MPI25960	WP_027124232.1	*M. pirum* MPI25960	WP_052663051.1
M. pneumoniae	NR_041751.1							*M. pneumoniae* M129	NP_110070.1
M. primatum	NR_025068.1					*M. primatum* ATCC 25948	WP_029513805.1	*M. primatum* ATCC 25948	WP_029513151.1
M. pulmonis	NR_041744.1	*M. pulmonis* UAB CTIP	WP_010925274.1			*M. pulmonis* UAB CTIP	WP_010924901.1	*M. pulmonis* UAB CTIP	WP_010925522.1
M. putrefaciens	NR_025971.1					*M. putrefaciens* KS1	WP_014035258.1	*M. putrefaciens* KS1	WP_014035169.1
M. simbae	NR_025964.1					*M. simbae* ATCC 49888	WP_029608728.1	*M. simbae* ATCC 49888	WP_029608675.1
M. sturni	NR_025968.1	*M. sturni* DSM 22021	WP_036464058.1	*M. sturni* DSM 22021	WP_036464056.1	*M. sturni* DSM 22021	WP_036464160.1	*M. sturni* DSM 22021	WP_036464601.1[a]
M. suis	EU603330.1								
M. synoviae	NR_044811.1	*M. synoviae* 53	WP_041351793.1	*M. synoviae* 53	WP_011283173.1	*M. synoviae* 53	AAZ44058.2[b]	*M. synoviae* 53	WP_041352058.1[a]
M. sp. Ms02	DQ223546.1							*M.* sp. Ms02	QZE12339.1
M. testudinis	NR_029175.1	*M. testudinis* ATCC 43263	WP_027120000.1	*M. testudinis* ATCC 43263	WP_084266141.1[a]	*M. testudinis* ATCC 43263	WP_036499102.1	*M. testudinis* ATCC 43263	WP_027119674.1
M. wenyonii	KX171205.1								
M. yeatsii	NR_026037.1					*M. yeatsii* GM274B	WP_042733350.1	*M. yeatsii* GM274B	WP_004427570.1

[a]Annotated as hypothetical protein
[b]Annotated as putative protein
[c]Bifunctional HAD-like/dephospho-coenzyme A kinase protein
ORF = open reading frame

Supplementary Table 3: The related organisms used in the phylogenetic analyses and the NCBI accession numbers of their respective CoA biosynthetic proteins

Organism	NCBI accession number			
	**PanK type III**	**CoaBC**	**PPAT**	**DPCK**
B. coahuilensis m2-6	WP_010169518.1	WP_082688052.1	WP_059282518.1	WP_059283260.1
C. innocuum I46	ASU20925.1	ASU20926.1	ASU18107.1	ASU18339.1
L. fermentum IFO 3956	-	WP_012391452.1	BAG26942.1	BAG27651.1
S. pneumoniae N	CKG77743.1	-	CKE32357.1	CKG66021.1

Supplementary Table 4 PanK CDD results

Query	Hit type	ID region start	ID region end	E-Value	Accession	Short name	Superfamily	Definition
M. alligatoris	non-specific	9	190	8.15e-17	pfam03309	Pan_kinase	cl17037	Type III pantothenate kinase
	superfamily	9	190	8.15e-17	cl17037	NBD_sugar-kinase_HSP70_actin superfamily	-	Nucleotide-Binding Domain of the sugar kinase/HSP70/actin superfamily
	non-specific	9	249	1.80e-15	PRK13320	PRK13320	cl17037	pantothenate kinase
	non-specific	8	190	5.16e-11	COG1521	CoaX	cl17037	Pantothenate kinase type III
	non-specific	7	253	3.09e-10	PRK13321	PRK13321	cl17037	pantothenate kinase
	non-specific	8	190	7.94e-10	TIGR00671	baf	cl17037	pantothenate kinase, type III
	non-specific	9	190	2.26e-05	PRK13318	PRK13318	cl17037	pantothenate kinase
	non-specific	8	229	4.00e-05	PRK13324	PRK13324	cl17037	pantothenate kinase
M. alvi	non-specific	9	197	2.86e-26	pfam03309	Pan_kinase	cl17037	Type III pantothenate kinase
	superfamily	9	197	2.86e-26	cl17037	NBD_sugar-kinase_HSP70_actin superfamily	-	Nucleotide-Binding Domain of the sugar kinase/HSP70/actin superfamily
	non-specific	9	247	1.01e-17	PRK13320	PRK13320	cl17037	pantothenate kinase
	non-specific	9	255	1.05e-17	COG1521	CoaX	cl17037	Pantothenate kinase type III

	non-specific	8	196	7.61e-17	PRK13321	PRK13321	cl17037	pantothenate kinase
	non-specific	9	255	6.31e-13	PRK13318	PRK13318	cl17037	pantothenate kinase
	non-specific	9	225	8.06e-12	TIGR00671	baf	cl17037	pantothenate kinase, type III
	non-specific	8	256	3.44e-07	PRK13324	PRK13324	cl17037	pantothenate kinase
	specific	9	156	5.96e-05	cd00012	NBD_sugar-kinase_HSP70_actin	cl17037	Nucleotide-Binding Domain of the sugar kinase/HSP70/actin superfamily
	non-specific	1	225	0.000472	PRK13326	PRK13326	cl17037	pantothenate kinase
	non-specific	1	247	0.00078	PRK13331	PRK13331	cl17037	pantothenate kinase
M. anatis	-	-	-	-	-	-	-	-
M. arginini	non-specific	10	249	8.45e-22	COG1521	CoaX	cl17037	Pantothenate kinase type III
	superfamily	10	249	8.45e-22	cl17037	NBD_sugar-kinase_HSP70_actin superfamily	-	Nucleotide-Binding Domain of the sugar kinase/HSP70/actin superfamily
	non-specific	10	205	9.42e-21	pfam03309	Pan_kinase	cl17037	Type III pantothenate kinase
	non-specific	10	246	3.67e-17	PRK13320	PRK13320	cl17037	pantothenate kinase
	non-specific	10	241	5.97e-14	TIGR00671	baf	cl17037	pantothenate kinase, type III
	non-specific	10	250	1.08e-11	PRK13318	PRK13318	cl17037	pantothenate kinase
	non-specific	8	239	4.15e-06	PRK13326	PRK13326	cl17037	pantothenate kinase
	non-specific	10	249	0.000186	PRK13324	PRK13324	cl17037	pantothenate kinase
M. buteonis	non-specific	5	198	1.60e-06	pfam03309	Pan_kinase	cl17037	Type III pantothenate kinase
	superfamily	5	198	1.60e-06	cl17037	NBD_sugar-kinase_HSP70_actin superfamily	-	Nucleotide-Binding Domain of the sugar kinase/HSP70/actin superfamily
	non-specific	5	234	3.26e-06	PRK13320	PRK13320	cl17037	pantothenate kinase
	non-specific	5	235	6.44e-06	TIGR00671	baf	cl17037	pantothenate kinase, type III
	non-specific	5	234	8.74e-06	COG1521	CoaX	cl17037	Pantothenate kinase type III
M. columborale	non-specific	6	209	1.18e-12	pfam03309	Pan_kinase	cl17037	Type III pantothenate kinase

	superfamily	6	209	1.18e-12	cl17037	NBD_sugar-kinase_HSP70_actin superfamily	-	Nucleotide-Binding Domain of the sugar kinase/HSP70/actin superfamily
	non-specific	3	232	5.53e-10	COG1521	CoaX	cl17037	Pantothenate kinase type III
	non-specific	4	233	1.26e-06	TIGR00671	baf	cl17037	pantothenate kinase, type III
	non-specific	1	235	0.000122	PRK13320	PRK13320	cl17037	pantothenate kinase
	non-specific	4	223	0.00372	PRK13321	PRK13321	cl17037	pantothenate kinase
M. cricetuli	non-specific	4	233	5.58e-10	COG1521	CoaX	cl17037	Pantothenate kinase type III
	superfamily	4	233	5.58e-10	cl17037	NBD_sugar-kinase_HSP70_actin superfamily	-	Nucleotide-Binding Domain of the sugar kinase/HSP70/actin superfamily
	non-specific	112	234	1.08e-06	TIGR00671	baf	cl17037	pantothenate kinase, type III
	non-specific	5	209	9.25e-05	pfam03309	Pan_kinase	cl17037	Type III pantothenate kinase
	non-specific	189	249	0.004086	PRK13320	PRK13320	cl17037	pantothenate kinase
M. crocodyli	non-specific	14	195	3.44e-15	pfam03309	Pan_kinase	cl17037	Type III pantothenate kinase
	superfamily	14	195	3.44e-15	cl17037	NBD_sugar-kinase_HSP70_actin superfamily	-	Nucleotide-Binding Domain of the sugar kinase/HSP70/actin superfamily
	non-specific	14	253	1.86e-13	TIGR00671	baf	cl17037	pantothenate kinase, type III
	non-specific	13	256	1.91e-13	COG1521	CoaX	cl17037	Pantothenate kinase type III
	non-specific	12	257	6.11e-13	PRK13320	PRK13320	cl17037	pantothenate kinase
	non-specific	14	253	1.58e-07	PRK13321	PRK13321	cl17037	pantothenate kinase
	non-specific	14	248	5.79e-06	PRK13324	PRK13324	cl17037	pantothenate kinase
M. gallinaceum	non-specific	9	195	5.46e-16	pfam03309	Pan_kinase	cl17037	Type III pantothenate kinase
	superfamily	9	195	5.46e-16	cl17037	NBD_sugar-kinase_HSP70_actin superfamily	-	Nucleotide-Binding Domain of the sugar kinase/HSP70/actin superfamily
	non-specific	6	245	3.79e-15	COG1521	CoaX	cl17037	Pantothenate kinase type III
	non-specific	6	245	1.22e-09	PRK13320	PRK13320	cl17037	pantothenate kinase
	non-specific	9	196	7.81e-08	TIGR00671	baf	cl17037	pantothenate kinase, type III

	non-specific	6	248	4.54e-07	PRK13318	PRK13318	cl17037	pantothenate kinase
	non-specific	7	245	3.06e-06	PRK13321	PRK13321	cl17037	pantothenate kinase
	non-specific	6	212	0.000497	PRK13324	PRK13324	cl17037	pantothenate kinase
M. iowae	non-specific	6	204	3.30e-26	pfam03309	Pan_kinase	cl17037	Type III pantothenate kinase
	superfamily	6	204	3.30e-26	cl17037	NBD_sugar-kinase_HSP70_actin superfamily	-	Nucleotide-Binding Domain of the sugar kinase/HSP70/actin superfamily
	non-specific	4	251	1.33e-20	COG1521	CoaX	cl17037	Pantothenate kinase type III
	non-specific	5	191	3.98e-17	PRK13318	PRK13318	cl17037	pantothenate kinase
	non-specific	6	249	3.70e-15	TIGR00671	baf	cl17037	pantothenate kinase, type III
	non-specific	2	252	2.04e-13	PRK13320	PRK13320	cl17037	pantothenate kinase
	non-specific	5	198	2.95e-08	PRK13324	PRK13324	cl17037	pantothenate kinase
	non-specific	5	191	8.10e-08	PRK13321	PRK13321	cl17037	pantothenate kinase
M. mobile	specific	3	197	4.16e-45	pfam03309	Pan_kinase	cl17037	Type III pantothenate kinase
	superfamily	3	197	4.16e-45	cl17037	NBD_sugar-kinase_HSP70_actin superfamily	-	Nucleotide-Binding Domain of the sugar kinase/HSP70/actin superfamily
	non-specific	1	235	3.89e-38	COG1521	CoaX	cl17037	Pantothenate kinase type III
M. mobile	non-specific	1	240	1.19e-27	PRK13318	PRK13318	cl17037	pantothenate kinase
	non-specific	1	240	6.36e-26	PRK13321	PRK13321	cl17037	pantothenate kinase
	non-specific	3	236	7.86e-25	TIGR00671	baf	cl17037	pantothenate kinase, type III
	non-specific	1	235	1.87e-14	PRK13320	PRK13320	cl17037	pantothenate kinase
	non-specific	3	217	3.77e-11	PRK13326	PRK13326	cl17037	pantothenate kinase
	non-specific	1	219	5.53e-10	PRK13324	PRK13324	cl17037	pantothenate kinase
	non-specific	3	236	1.08e-07	PRK13331	PRK13331	cl17037	pantothenate kinase
	non-specific	118	241	2.91e-07	PRK13322	PRK13322	cl17037	pantothenate kinase

	non-specific	93	235	1.66e-05	PRK13328	PRK13328	cl17037	pantothenate kinase
	non-specific	6	236	0.000303	PRK13333	PRK13333	cl17037	pantothenate kinase
	non-specific	31	124	0.007289	cd14228	STKc_HIPK1	cl21453	Catalytic domain of the Serine/Threonine Kinase, Homeodomain-Interacting Protein Kinase 1
	superfamily	31	124	0.007289	cl21453	PKc_like superfamily	-	Protein Kinases, catalytic domain
M. molare	non-specific	1	231	3.20e-23	COG1521	CoaX	cl17037	Pantothenate kinase type III
	superfamily	1	231	3.20e-23	cl17037	NBD_sugar-kinase_HSP70_actin superfamily	-	Nucleotide-Binding Domain of the sugar kinase/HSP70/actin superfamily
	non-specific	2	198	2.50e-19	pfam03309	Pan_kinase	cl17037	Type III pantothenate kinase
	non-specific	1	228	7.15e-18	TIGR00671	baf	cl17037	pantothenate kinase, type III
	non-specific	37	213	1.39e-15	PRK13318	PRK13318	cl17037	pantothenate kinase
	non-specific	116	231	6.27e-10	PRK13333	PRK13333	cl17037	pantothenate kinase
	non-specific	2	212	8.31e-08	PRK13321	PRK13321	cl17037	pantothenate kinase
	non-specific	1	229	1.20e-05	PRK13324	PRK13324	cl17037	pantothenate kinase
	non-specific	2	222	1.45e-05	PRK13320	PRK13320	cl17037	pantothenate kinase
	non-specific	2	226	0.00397	PRK13326	PRK13326	cl17037	pantothenate kinase
M. penetrans	specific	9	193	1.56e-33	pfam03309	Pan_kinase	cl17037	Type III pantothenate kinase
	superfamily	9	193	1.56e-33	cl17037	NBD_sugar-kinase_HSP70_actin superfamily	-	Nucleotide-Binding Domain of the sugar kinase/HSP70/actin superfamily
	non-specific	8	254	4.34e-21	COG1521	CoaX	cl17037	Pantothenate kinase type III
	non-specific	8	255	2.72e-16	PRK13320	PRK13320	cl17037	pantothenate kinase
	non-specific	9	226	3.94e-16	TIGR00671	baf	cl17037	pantothenate kinase, type III
	non-specific	8	224	2.33e-15	PRK13318	PRK13318	cl17037	pantothenate kinase
	non-specific	7	254	3.06e-15	PRK13321	PRK13321	cl17037	pantothenate kinase
	non-specific	8	255	1.80e-11	PRK13324	PRK13324	cl17037	pantothenate kinase

	non-specific	9	227	0.00134	PRK13326	PRK13326	cl17037	pantothenate kinase
	non-specific	126	193	0.001658	PRK13331	PRK13331	cl17037	pantothenate kinase
M. pirum	non-specific	9	193	1.71e-16	pfam03309	Pan_kinase	cl17037	Type III pantothenate kinase
	superfamily	9	193	1.71e-16	cl17037	NBD_sugar-kinase_HSP70_actin superfamily	-	Nucleotide-Binding Domain of the sugar kinase/HSP70/actin superfamily
	non-specific	9	217	5.01e-13	COG1521	CoaX	cl17037	Pantothenate kinase type III
	non-specific	9	227	1.27e-12	TIGR00671	baf	cl17037	pantothenate kinase, type III
	non-specific	9	253	3.76e-12	PRK13320	PRK13320	cl17037	pantothenate kinase
	non-specific	8	193	1.85e-06	PRK13321	PRK13321	cl17037	pantothenate kinase
	non-specific	9	193	1.12e-05	PRK13318	PRK13318	cl17037	pantothenate kinase
M. pulmonis	specific	1	224	7.06e-60	COG1521	CoaX	cl17037	Pantothenate kinase type III
	superfamily	1	224	7.06e-60	cl17037	NBD_sugar-kinase_HSP70_actin superfamily	-	Nucleotide-Binding Domain of the sugar kinase/HSP70/actin superfamily
	non-specific	5	216	1.95e-18	TIGR00671	baf	cl17037	pantothenate kinase, type III
	non-specific	3	203	1.78e-13	pfam03309	Pan_kinase	cl17037	Type III pantothenate kinase
	non-specific	1	216	1.45e-11	PRK13321	PRK13321	cl17037	pantothenate kinase
	non-specific	1	216	4.25e-10	PRK13320	PRK13320	cl17037	pantothenate kinase
	non-specific	1	224	6.07e-07	PRK13318	PRK13318	cl17037	pantothenate kinase
	non-specific	121	216	0.000398	PRK13333	PRK13333	cl17037	pantothenate kinase
M. sturni	non-specific	6	209	5.47e-09	pfam03309	Pan_kinase	cl17037	Type III pantothenate kinase
	superfamily	6	209	5.47e-09	cl17037	NBD_sugar-kinase_HSP70_actin superfamily	-	Nucleotide-Binding Domain of the sugar kinase/HSP70/actin superfamily
	non-specific	4	211	2.00e-05	PRK13320	PRK13320	cl17037	pantothenate kinase
	non-specific	6	264	2.17e-05	COG1521	CoaX	cl17037	Pantothenate kinase type III
	non-specific	6	260	7.24e-05	TIGR00671	baf	cl17037	pantothenate kinase, type III

M. synoviae	non-specific	10	249	2.74e-21	COG1521	CoaX	cl17037	Pantothenate kinase type III
	superfamily	10	249	2.74e-21	cl17037	NBD_sugar-kinase_HSP70_actin superfamily	-	Nucleotide-Binding Domain of the sugar kinase/HSP70/actin superfamily
	non-specific	10	205	3.32e-21	pfam03309	Pan_kinase	cl17037	Type III pantothenate kinase
	non-specific	10	246	1.13e-17	PRK13320	PRK13320	cl17037	pantothenate kinase
	non-specific	10	241	2.91e-14	TIGR00671	baf	cl17037	pantothenate kinase, type III
	non-specific	10	250	1.46e-11	PRK13318	PRK13318	cl17037	pantothenate kinase
	non-specific	8	239	4.27e-06	PRK13326	PRK13326	cl17037	pantothenate kinase
	non-specific	10	249	0.000354	PRK13324	PRK13324	cl17037	pantothenate kinase
M. testudinis	non-specific	5	242	1.90e-38	PRK13318	PRK13318	cl17037	pantothenate kinase
	superfamily	5	242	1.90e-38	cl17037	NBD_sugar-kinase_HSP70_actin superfamily	-	Nucleotide-Binding Domain of the sugar kinase/HSP70/actin superfamily
	non-specific	5	238	1.17e-37	PRK13321	PRK13321	cl17037	pantothenate kinase
	specific	5	201	1.90e-37	pfam03309	Pan_kinase	cl17037	Type III pantothenate kinase
	non-specific	5	242	3.20e-36	COG1521	CoaX	cl17037	Pantothenate kinase type III
	non-specific	5	241	1.68e-26	TIGR00671	baf	cl17037	pantothenate kinase, type III
	non-specific	4	242	2.53e-19	PRK13320	PRK13320	cl17037	pantothenate kinase
	non-specific	5	246	6.96e-17	PRK13326	PRK13326	cl17037	pantothenate kinase
	non-specific	5	240	8.26e-17	PRK13324	PRK13324	cl17037	pantothenate kinase
	non-specific	9	236	1.30e-12	PRK13331	PRK13331	cl17037	pantothenate kinase
	non-specific	1	240	5.88e-10	PRK13322	PRK13322	cl17037	pantothenate kinase
	non-specific	8	243	6.43e-10	PRK13333	PRK13333	cl17037	pantothenate kinase
	non-specific	100	236	0.000424	PRK13328	PRK13328	cl17037	pantothenate kinase

Supplementary Table 5 PanK InterPro results

Mycoplasma species	Amino acid region	Database	Database ID	Database signature description	ID region start	ID region end	InterPro ID[b]	ID type[a]	Gene Ontology (GO) term[c]
M. alligatoris	253	Gene3D	G3DSA:3.30.420.40		4	134			
		PANTHER	PTHR34265		9	251	IPR004619	F	GO:0004594
		Pfam	PF03309	Type III pantothenate kinase	8	193	IPR004619	F	GO:0004594
		SUPERFAMILY	SSF53067		123	244			
		SUPERFAMILY	SSF53067		8	116			
		TIGRFAM	TIGR00671	baf: pantothenate kinase, type III	8	224	IPR004619	F	GO:0004594
M. alvi	257	CDD	cd00012	NBD_sugar-kinase_HSP70_actin	9	156			
		Gene3D	G3DSA:3.30.420.40		1	84			
		Gene3D	G3DSA:3.30.420.40		102	257			
		Hamap	MF_01274	Type III pantothenate kinase [coaX].	7	256	IPR004619		GO:0004594
		PANTHER	PTHR34265		8	255	IPR004619	F	GO:0004594
		Pfam	PF03309	Type III pantothenate kinase	9	202	IPR004619	F	GO:0004594
		SUPERFAMILY	SSF53067		9	121			
		SUPERFAMILY	SSF53067		127	252			
		TIGRFAM	TIGR00671	baf: pantothenate kinase, type III	9	228	IPR004619	F	GO:0004594
M. anatis	268	CDD	cd00012	NBD_sugar-kinase_HSP70_actin	8	172			
		Gene3D	G3DSA:3.30.420.40		3	143			
		Pfam	PF03309	Type III pantothenate kinase	8	173	IPR004619	F	GO:0004594
		SUPERFAMILY	SSF53067		7	114			
M. arginini	250	Gene3D	G3DSA:3.30.420.40		94	250			
		Hamap	MF_01274	Type III pantothenate kinase [coaX].	8	250	IPR004619	F	GO:0004594
		PANTHER	PTHR34265		10	249	IPR004619	F	GO:0004594
		Pfam	PF03309	Type III pantothenate kinase	10	206	IPR004619	F	GO:0004594
		SUPERFAMILY	SSF53067		126	249			
		SUPERFAMILY	SSF53067		9	120			
		TIGRFAM	TIGR00671	baf: pantothenate kinase, type III	10	234	IPR004619	F	GO:0004594
M. buteonis	246	Gene3D	G3DSA:3.30.420.40		1	100			
		Gene3D	G3DSA:3.30.420.40		101	240			
		Pfam	PF03309	Type III pantothenate kinase	5	198	IPR004619	F	GO:0004594
M. columborale	273	Gene3D	G3DSA:3.30.420.40		104	273			
		PANTHER	PTHR34265		5	271	IPR004619	F	GO:0004594
		Pfam	PF03309	Type III pantothenate kinase	5	209	IPR004619	F	GO:0004594
		SUPERFAMILY	SSF53067		129	235			
M. cricetuli	272	Gene3D	G3DSA:3.30.420.40		97	272			
		PANTHER	PTHR34265		4	242	IPR004619	F	GO:0004594
		Pfam	PF03309	Type III pantothenate kinase	4	209	IPR004619	F	GO:0004594
		SUPERFAMILY	SSF53067		133	242			
		SUPERFAMILY	SSF53067		4	124			
M. crocodyli	258	Gene3D	G3DSA:3.30.420.40		108	258			
		Hamap	MF_01274	Type III pantothenate kinase [coaX].	12	257	IPR004619	F	GO:0004594
M. crocodyli	258	PANTHER	PTHR34265		14	256	IPR004619	F	GO:0004594
		Pfam	PF03309	Type III pantothenate kinase	13	203	IPR004619	F	GO:0004594

Mycoplasma species	Amino acid region	Database	Database ID	Database signature description	ID region start	ID region end	InterPro ID[b]	ID type[a]	Gene Ontology (GO) term[c]
		SUPERFAMILY	SSF53067		128	250			
		SUPERFAMILY	SSF53067		13	123			
		TIGRFAM	TIGR00671	baf: pantothenate kinase, type III	14	241	IPR004619	F	GO:0004594
M. gallinaceum	248	Gene3D	G3DSA:3.30.420.40		90	247			
		PANTHER	PTHR34265		6	245	IPR004619	F	GO:0004594
		Pfam	PF03309	Type III pantothenate kinase	6	196	IPR004619	F	GO:0004594
		SUPERFAMILY	SSF53067		6	113			
		SUPERFAMILY	SSF53067		119	245			
		TIGRFAM	TIGR00671	baf: pantothenate kinase, type III	7	206	IPR004619	F	GO:0004594
M. iowae	258	Gene3D	G3DSA:3.30.420.40		89	258			
		Hamap	MF_01274	Type III pantothenate kinase [coaX].	4	252	IPR004619	F	GO:0004594
		PANTHER	PTHR34265		5	251	IPR004619	F	GO:0004594
		Pfam	PF03309	Type III pantothenate kinase	5	204	IPR004619	F	GO:0004594
		SUPERFAMILY	SSF53067		5	114			
		SUPERFAMILY	SSF53067		123	252			
		TIGRFAM	TIGR00671	baf: pantothenate kinase, type III	6	236	IPR004619	F	GO:0004594
M. mobile	243	Gene3D	G3DSA:3.30.420.40		88	242			
		Hamap	MF_01274	Type III pantothenate kinase [coaX].	1	243	IPR004619	F	GO:0004594
		PANTHER	PTHR34265		1	236	IPR004619	F	GO:0004594
		Pfam	PF03309	Type III pantothenate kinase	3	197	IPR004619	F	GO:0004594
		SUPERFAMILY	SSF53067		1	115			
		SUPERFAMILY	SSF53067		117	240			
		TIGRFAM	TIGR00671	baf: pantothenate kinase, type III	3	235	IPR004619	F	GO:0004594
M. molare	242	Gene3D	G3DSA:3.30.420.40		90	238			
		Hamap	MF_01274	Type III pantothenate kinase [coaX].	1	242	IPR004619	F	GO:0004594
		PANTHER	PTHR34265		2	236	IPR004619	F	GO:0004594
		Pfam	PF03309	Type III pantothenate kinase	1	198	IPR004619	F	GO:0004594
		SUPERFAMILY	SSF53067		117	228			
		SUPERFAMILY	SSF53067		1	115			
		TIGRFAM	TIGR00671	baf: pantothenate kinase, type III	1	228	IPR004619	F	GO:0004594
M. penetrans	255	Gene3D	G3DSA:3.30.420.40		4	138			
		Hamap	MF_01274	Type III pantothenate kinase [coaX].	7	255	IPR004619	F	GO:0004594
		PANTHER	PTHR34265		8	253	IPR004619	F	GO:0004594
		Pfam	PF03309	Type III pantothenate kinase	8	203	IPR004619	F	GO:0004594
		SUPERFAMILY	SSF53067		124	253			
		SUPERFAMILY	SSF53067		7	119			
		TIGRFAM	TIGR00671	baf: pantothenate kinase, type III	9	229	IPR004619	F	GO:0004594
M. pirum	256	Gene3D	G3DSA:3.30.420.40		93	255			
		PANTHER	PTHR34265		8	248	IPR004619	F	GO:0004594
		Pfam	PF03309	Type III pantothenate kinase	9	204	IPR004619	F	GO:0004594
		SUPERFAMILY	SSF53067		125	254			
M. pirum	256	SUPERFAMILY	SSF53067		9	120			
		TIGRFAM	TIGR00671	baf: pantothenate kinase, type III	9	231	IPR004619	F	GO:0004594

Mycoplasma species	Amino acid region	Database	Database ID	Database signature description	ID region start	ID region end	InterPro ID[b]	ID type[a]	Gene Ontology (GO) term[c]
M. pulmonis	224	Gene3D	G3DSA:3.30.420.40		96	222			
		PANTHER	PTHR34265		1	217	IPR004619	F	GO:0004594
		Pfam	PF03309	Type III pantothenate kinase	3	203	IPR004619	F	GO:0004594
		SUPERFAMILY	SSF53067		121	218			
		SUPERFAMILY	SSF53067		1	118			
		TIGRFAM	TIGR00671	baf: pantothenate kinase, type III	4	217	IPR004619	F	GO:0004594
M. sturni	273	Gene3D	G3DSA:3.30.420.40		100	273			
		Pfam	PF03309	Type III pantothenate kinase	6	210	IPR004619	F	GO:0004594
		Phobius	CYTOPLASMIC_DOMAIN	Region of a membrane-bound protein predicted to be outside the membrane, in the cytoplasm.	217	273			
		Phobius	TRANSMEMBRANE	Region of a membrane-bound protein predicted to be embedded in the membrane.	196	216			
		Phobius	NON_CYTOPLASMIC_DOMAIN	Region of a membrane-bound protein predicted to be outside the membrane, in the extracellular region.	1	195			
		SUPERFAMILY	SSF53067		130	237			
		TMHMM	TMhelix	Region of a membrane-bound protein predicted to be embedded in the membrane.	194	216			
M. synoviae	250	Gene3D	G3DSA:3.30.420.40		94	250			
		Hamap	MF_01274	Type III pantothenate kinase [coaX].	8	250	IPR004619	F	GO:0004594
		PANTHER	PTHR34265		10	249	IPR004619	F	GO:0004594
		Pfam	PF03309	Type III pantothenate kinase	10	206	IPR004619	F	GO:0004594
		SUPERFAMILY	SSF53067		9	120			
		SUPERFAMILY	SSF53067		126	249			
		TIGRFAM	TIGR00671	baf: pantothenate kinase, type III	10	234	IPR004619	F	GO:0004594
M. testudinis	248	Gene3D	G3DSA:3.30.420.40		92	246			
		Hamap	MF_01274	Type III pantothenate kinase [coaX].	3	244	IPR004619	F	GO:0004594
		PANTHER	PTHR34265		5	242	IPR004619	F	GO:0004594
		Pfam	PF03309	Type III pantothenate kinase	5	196	IPR004619	F	GO:0004594
		SUPERFAMILY	SSF53067		4	119			
		SUPERFAMILY	SSF53067		121	240			
		TIGRFAM	TIGR00671	baf: pantothenate kinase, type III	5	240	IPR004619	F	GO:0004594

[a]ID type abbreviations – F, Family

[b]InterPro ID – IPR004619: Type III pantothenate kinase

[c]GO term (Molecular Function) – GO:0004594: pantothenate kinase activity

Supplementary Table 6 PanK MEME + motif locations

Mycoplasma species	*p*-value	Motif locations			
		Motif 1	Motif 2	Motif 3	Motif 4

M. alligatoris	4.92e-61	9-23	106-134	140-168	179-199
M. alvi	1.57e-56	9-23	110-138	144-172	183-203
M. anatis	4.52e-15	8-22	-	146-174	-
M. arginini	3.89e-62	10-24	109-137	143-171	182-202
M. buteonis	2.10e-16	5-19	-	134-162	-
M. columborale	9.16e-54	5-19	112-140	146-174	189-209
M. cricetuli	2.89e-52	5-19	112-140	146-174	189-209
M. crocodyli	5.10e-58	14-28	111-139	145-173	184-204
M. gallinaceum	2.33e-56	6-20	101-129	135-163	174-194
M. iowae	4.98e-57	6-20	106-134	140-168	179-199
M. mobile	9.91e-41	3-17	100-128	134-162	-
M. molare	4.05e-34	2-16	100-128	134-162	-
M. penetrans	1.23e-54	9-23	108-136	142-170	181-201
M. pirum	1.09e-53	9-23	108-136	142-170	181-201
M. pulmonis	1.02e-33	3-17	105-133	139-167	-
M. sturni	2.48e-45	5-19	112-140	146-174	189-209
M. synoviae	1.86e-62	10-24	109-137	143-171	182-202
M. testudinis	8.30e-46	5-19	104-132	138-166	-

Supplementary Table 7 CoaBC CDD results

Query	Hit type	ID region start	ID region end	E-Value	Accession	Short name	Superfamily	Definition
M. anatis	non-specific	2	375	6.90e-78	PRK05579	PRK05579	cl27193	bifunctional phosphopantothenoylcysteine decarboxylase/phosphopantothenate synthase
	superfamily	2	375	6.90e-78	cl27193	DFP superfamily	-	DNA / pantothenate metabolism flavoprotein
	non-specific	1	371	5.97e-71	TIGR00521	coaBC_dfp	cl27193	phosphopantothenoylcysteine decarboxylase / phosphopantothenate--cysteine ligase
	specific	1	375	5.33e-61	COG0452	CoaBC	cl27193	Phosphopantothenoylcysteine synthetase/decarboxylase
	non-specific	1	172	9.30e-37	PRK07313	PRK07313	cl19190	phosphopantothenoylcysteine decarboxylase
	superfamily	1	172	9.30e-37	cl19190	Flavoprotein superfamily	-	Flavoprotein
	non-specific	2	315	8.63e-32	PRK13982	PRK13982	cl27193	bifunctional SbtC-like/phosphopantothenoylcysteine decarboxylase/phosphopantothenate synthase
	specific	1	139	1.04e-27	pfam02441	Flavoprotein	cl19190	Flavoprotein
	non-specific	171	332	2.78e-25	pfam04127	DFP	cl27193	DNA / pantothenate metabolism flavoprotein

	non-specific	2	162	8.50e-23	TIGR02113	coaC_strep	cl19190	phosphopantothenoylcysteine decarboxylase, streptococcal
	non-specific	2	158	2.63e-21	PLN02496	PLN02496	cl19190	probable phosphopantothenoylcysteine decarboxylase
	non-specific	171	372	3.59e-09	PRK09620	PRK09620	cl27193	hypothetical protein
	non-specific	172	372	2.26e-07	PRK06732	PRK06732	cl27193	phosphopantothenate--cysteine ligase
	non-specific	172	371	2.59e-05	TIGR02114	coaB_strep	cl27193	phosphopantothenate--cysteine ligase, streptococcal
	non-specific	71	96	0.000464	PRK08305	spoVFB	cl19190	dipicolinate synthase subunit B
M. arginini	non-specific	2	373	5.24e-81	PRK05579	PRK05579	cl27193	bifunctional phosphopantothenoylcysteine decarboxylase/phosphopantothenate synthase
	superfamily	2	373	5.24e-81	cl27193	DFP superfamily	-	DNA / pantothenate metabolism flavoprotein
	specific	1	373	1.99e-68	COG0452	CoaBC	cl27193	Phosphopantothenoylcysteine synthetase/decarboxylase
	non-specific	1	340	9.94e-68	TIGR00521	coaBC_dfp	cl27193	phosphopantothenoylcysteine decarboxylase / phosphopantothenate--cysteine ligase
	non-specific	2	340	6.57e-42	PRK13982	PRK13982	cl27193	bifunctional SbtC-like/phosphopantothenoylcysteine decarboxylase/phosphopantothenate synthase
	non-specific	1	169	2.33e-36	PRK07313	PRK07313	cl19190	phosphopantothenoylcysteine decarboxylase
	superfamily	1	169	2.33e-36	cl19190	Flavoprotein superfamily	-	Flavoprotein
	specific	1	173	9.83e-35	pfam02441	Flavoprotein	cl19190	Flavoprotein
	non-specific	169	340	7.83e-30	pfam04127	DFP	cl27193	DNA / pantothenate metabolism flavoprotein
	non-specific	2	169	1.61e-25	TIGR02113	coaC_strep	cl19190	phosphopantothenoylcysteine decarboxylase, streptococcal
	non-specific	2	176	3.30e-19	PLN02496	PLN02496	cl19190	probable phosphopantothenoylcysteine decarboxylase
	non-specific	176	368	1.25e-06	TIGR02114	coaB_strep	cl27193	phosphopantothenate--cysteine ligase, streptococcal
	non-specific	176	342	3.52e-05	PRK09620	PRK09620	cl27193	hypothetical protein
	non-specific	176	368	4.15e-05	PRK06732	PRK06732	cl27193	phosphopantothenate--cysteine ligase
M. columborale	non-specific	2	374	5.64e-92	PRK05579	PRK05579	cl27193	bifunctional phosphopantothenoylcysteine decarboxylase/phosphopantothenate synthase
	superfamily	2	374	5.64e-92	cl27193	DFP superfamily	-	DNA / pantothenate metabolism flavoprotein
	non-specific	1	371	1.57e-80	TIGR00521	coaBC_dfp	cl27193	phosphopantothenoylcysteine decarboxylase / phosphopantothenate--cysteine ligase
M. columborale	specific	1	375	3.89e-74	COG0452	CoaBC	cl27193	Phosphopantothenoylcysteine synthetase/decarboxylase

	non-specific	2	334	3.07e-44	PRK13982	PRK13982	cl27193	bifunctional SbtC-like/phosphopantothenoylcysteine decarboxylase/phosphopantothenate synthase
	non-specific	1	167	1.76e-38	PRK07313	PRK07313	cl19190	phosphopantothenoylcysteine decarboxylase
	superfamily	1	167	1.76e-38	cl19190	Flavoprotein superfamily	-	Flavoprotein
	specific	1	169	1.61e-35	pfam02441	Flavoprotein	cl19190	Flavoprotein
	non-specific	171	332	2.35e-35	pfam04127	DFP	cl27193	DNA / pantothenate metabolism flavoprotein
	non-specific	2	153	3.16e-25	TIGR02113	coaC_strep	cl19190	phosphopantothenoylcysteine decarboxylase, streptococcal
	non-specific	2	168	3.40e-23	PLN02496	PLN02496	cl19190	probable phosphopantothenoylcysteine decarboxylase
	non-specific	172	263	3.28e-06	TIGR02114	coaB_strep	cl27193	phosphopantothenate--cysteine ligase, streptococcal
	non-specific	172	263	4.86e-06	PRK06732	PRK06732	cl27193	phosphopantothenate--cysteine ligase
	non-specific	1	102	3.18e-05	COG0163	UbiX	cl19190	3-polyprenyl-4-hydroxybenzoate decarboxylase
	non-specific	1	102	0.000164	PRK05920	PRK05920	cl19190	aromatic acid decarboxylase
	non-specific	2	140	0.007562	TIGR00421	ubiX_pad	cl19190	polyprenyl P-hydroxybenzoate and phenylacrylic acid decarboxylases
	non-specific	1	102	0.009011	PRK06029	PRK06029	cl19190	3-octaprenyl-4-hydroxybenzoate carboxy-lyase
M. cricetuli	non-specific	2	377	8.21e-88	PRK05579	PRK05579	cl27193	bifunctional phosphopantothenoylcysteine decarboxylase/phosphopantothenate synthase
	superfamily	2	377	8.21e-88	cl27193	DFP superfamily	-	DNA / pantothenate metabolism flavoprotein
	non-specific	1	367	2.67e-73	TIGR00521	coaBC_dfp	cl27193	phosphopantothenoylcysteine decarboxylase / phosphopantothenate--cysteine ligase
	specific	1	376	7.41e-72	COG0452	CoaBC	cl27193	Phosphopantothenoylcysteine synthetase/decarboxylase
	non-specific	2	371	2.81e-41	PRK13982	PRK13982	cl27193	bifunctional SbtC-like/phosphopantothenoylcysteine decarboxylase/phosphopantothenate synthase
	specific	169	332	1.02e-37	pfam04127	DFP	cl27193	DNA / pantothenate metabolism flavoprotein
	non-specific	1	167	1.27e-33	PRK07313	PRK07313	cl19190	phosphopantothenoylcysteine decarboxylase
	superfamily	1	167	1.27e-33	cl19190	Flavoprotein superfamily	-	Flavoprotein
	non-specific	1	168	8.96e-31	pfam02441	Flavoprotein	cl19190	Flavoprotein
	non-specific	2	158	4.89e-17	PLN02496	PLN02496	cl19190	probable phosphopantothenoylcysteine decarboxylase

	non-specific	2	167	7.40e-17	TIGR02113	coaC_strep	cl19190	phosphopantothenoylcysteine decarboxylase, streptococcal
	non-specific	169	374	3.40e-07	PRK09620	PRK09620	cl27193	hypothetical protein
	non-specific	172	372	1.44e-06	PRK06732	PRK06732	cl27193	phosphopantothenate--cysteine ligase
	non-specific	1	81	0.003177	pfam02525	Flavodoxin_2	cl00438	Flavodoxin-like fold
	superfamily	1	81	0.003177	cl00438	FMN_red superfamily	-	NADPH-dependent FMN reductase
M. gallinaceum	non-specific	2	339	2.03e-74	PRK05579	PRK05579	cl27193	bifunctional phosphopantothenoylcysteine decarboxylase/phosphopantothenate synthase
	superfamily	2	339	2.03e-74	cl27193	DFP superfamily	-	DNA / pantothenate metabolism flavoprotein
	specific	1	340	1.50e-59	COG0452	CoaBC	cl27193	Phosphopantothenoylcysteine synthetase/decarboxylase
	non-specific	1	378	1.60e-59	TIGR00521	coaBC_dfp	cl27193	phosphopantothenoylcysteine decarboxylase / phosphopantothenate--cysteine ligase
	non-specific	1	179	1.07e-38	PRK07313	PRK07313	cl19190	phosphopantothenoylcysteine decarboxylase
	superfamily	1	179	1.07e-38	cl19190	Flavoprotein superfamily	-	Flavoprotein
M. gallinaceum	non-specific	4	339	1.22e-33	PRK13982	PRK13982	cl27193	bifunctional SbtC-like/phosphopantothenoylcysteine decarboxylase/phosphopantothenate synthase
	non-specific	2	160	8.43e-32	TIGR02113	coaC_strep	cl19190	phosphopantothenoylcysteine decarboxylase, streptococcal
	specific	1	172	4.18e-26	pfam02441	Flavoprotein	cl19190	Flavoprotein
	non-specific	179	339	6.89e-26	pfam04127	DFP	cl27193	DNA / pantothenate metabolism flavoprotein
	non-specific	2	165	1.12e-17	PLN02496	PLN02496	cl19190	probable phosphopantothenoylcysteine decarboxylase
	non-specific	179	377	4.29e-06	PRK06732	PRK06732	cl27193	phosphopantothenate--cysteine ligase
	non-specific	1	103	0.000137	COG0163	UbiX	cl19190	3-polyprenyl-4-hydroxybenzoate decarboxylase
	non-specific	179	381	0.000214	TIGR02114	coaB_strep	cl27193	phosphopantothenate--cysteine ligase, streptococcal
	non-specific	77	109	0.000224	COG1036	COG1036	cl27425	Archaeal flavoprotein
	superfamily	77	109	0.000224	cl27425	COG1036 superfamily	-	Archaeal flavoprotein
	non-specific	179	356	0.000777	PRK09620	PRK09620	cl27193	hypothetical protein
	non-specific	76	107	0.001221	TIGR02700	flavo_MJ0208	cl25361	archaeoflavoprotein, MJ0208 family
	superfamily	76	107	0.001221	cl25361	NuoI superfamily	-	Formate hydrogenlyase subunit 6/NADH:ubiquinone oxidoreductase 23 kD subunit (chain I)

	non-specific	77	114	0.002808	TIGR02699	archaeo_AfpA	cl27425	archaeoflavoprotein AfpA
M. mobile	non-specific	1	356	7.51e-80	PRK05579	PRK05579	cl27193	bifunctional phosphopantothenoylcysteine decarboxylase/phosphopantothenate synthase
	superfamily	1	356	7.51e-80	cl27193	DFP superfamily	-	DNA / pantothenate metabolism flavoprotein
	non-specific	2	355	8.99e-63	TIGR00521	coaBC_dfp	cl27193	phosphopantothenoylcysteine decarboxylase / phosphopantothenate--cysteine ligase
	specific	1	356	8.69e-61	COG0452	CoaBC	cl27193	Phosphopantothenoylcysteine synthetase/decarboxylase
	non-specific	2	317	1.03e-45	PRK13982	PRK13982	cl27193	bifunctional SbtC-like/phosphopantothenoylcysteine decarboxylase/phosphopantothenate synthase
	non-specific	1	173	1.98e-32	PRK07313	PRK07313	cl19190	phosphopantothenoylcysteine decarboxylase
	superfamily	1	173	1.98e-32	cl19190	Flavoprotein superfamily	-	Flavoprotein
	specific	2	164	5.41e-32	pfam02441	Flavoprotein	cl19190	Flavoprotein
	non-specific	176	318	2.58e-26	pfam04127	DFP	cl27193	DNA / pantothenate metabolism flavoprotein
	non-specific	2	169	5.12e-21	TIGR02113	coaC_strep	cl19190	phosphopantothenoylcysteine decarboxylase, streptococcal
	non-specific	64	161	5.43e-15	PLN02496	PLN02496	cl19190	probable phosphopantothenoylcysteine decarboxylase
	non-specific	177	356	8.29e-10	PRK06732	PRK06732	cl27193	phosphopantothenate--cysteine ligase
	non-specific	177	356	9.90e-10	TIGR02114	coaB_strep	cl27193	phosphopantothenate--cysteine ligase, streptococcal
	non-specific	22	115	1.38e-05	TIGR02852	spore_dpaB	cl19190	dipicolinic acid synthetase, B subunit
	non-specific	2	115	0.000155	PRK08305	spoVFB	cl19190	dipicolinate synthase subunit B
	non-specific	70	112	0.002594	COG1036	COG1036	cl27425	Archaeal flavoprotein
	superfamily	70	112	0.002594	cl27425	COG1036 superfamily	-	Archaeal flavoprotein
	non-specific	1	95	0.008873	PRK06029	PRK06029	cl19190	3-octaprenyl-4-hydroxybenzoate carboxy-lyase
M. sturni	non-specific	2	376	1.13e-104	PRK05579	PRK05579	cl27193	bifunctional phosphopantothenoylcysteine decarboxylase/phosphopantothenate synthase
	superfamily	2	376	1.13e-104	cl27193	DFP superfamily	-	DNA / pantothenate metabolism flavoprotein
	non-specific	1	370	7.06e-81	TIGR00521	coaBC_dfp	cl27193	phosphopantothenoylcysteine decarboxylase / phosphopantothenate--cysteine ligase
M. sturni	specific	1	375	1.29e-74	COG0452	CoaBC	cl27193	Phosphopantothenoylcysteine synthetase/decarboxylase
	non-specific	2	372	2.43e-49	PRK13982	PRK13982	cl27193	bifunctional SbtC-like/phosphopantothenoylcysteine decarboxylase/phosphopantothenate synthase

	non-specific	1	165	6.92e-40	PRK07313	PRK07313	cl19190	phosphopantothenoylcysteine decarboxylase
	superfamily	1	165	6.92e-40	cl19190	Flavoprotein superfamily	-	Flavoprotein
	specific	1	158	6.94e-37	pfam02441	Flavoprotein	cl19190	Flavoprotein
	non-specific	171	346	1.82e-36	pfam04127	DFP	cl27193	DNA / pantothenate metabolism flavoprotein
	non-specific	2	153	4.43e-28	TIGR02113	coaC_strep	cl19190	phosphopantothenoylcysteine decarboxylase, streptococcal
	non-specific	3	161	1.51e-22	PLN02496	PLN02496	cl19190	probable phosphopantothenoylcysteine decarboxylase
	non-specific	172	373	5.67e-09	PRK06732	PRK06732	cl27193	phosphopantothenate--cysteine ligase
	non-specific	171	376	2.66e-07	PRK09620	PRK09620	cl27193	hypothetical protein
	non-specific	172	371	1.08e-05	TIGR02114	coaB_strep	cl27193	phosphopantothenate--cysteine ligase, streptococcal
	non-specific	17	111	0.000138	TIGR02700	flavo_MJ0208	cl25361	archaeoflavoprotein, MJ0208 family
	superfamily	17	111	0.000138	cl25361	NuoI superfamily	-	Formate hydrogenlyase subunit 6/NADH:ubiquinone oxidoreductase 23 kD subunit (chain I)
	non-specific	71	103	0.001128	COG1036	COG1036	cl27425	Archaeal flavoprotein
	superfamily	71	103	0.001128	cl27425	COG1036 superfamily	-	Archaeal flavoprotein
M. synoviae	non-specific	2	373	5.66e-80	PRK05579	PRK05579	cl27193	bifunctional phosphopantothenoylcysteine decarboxylase/phosphopantothenate synthase
	superfamily	2	373	5.66e-80	cl27193	DFP superfamily	-	DNA / pantothenate metabolism flavoprotein
	specific	1	373	7.79e-67	COG0452	CoaBC	cl27193	Phosphopantothenoylcysteine synthetase/decarboxylase
	non-specific	1	340	2.68e-66	TIGR00521	coaBC_dfp	cl27193	phosphopantothenoylcysteine decarboxylase / phosphopantothenate--cysteine ligase
	non-specific	2	340	5.08e-41	PRK13982	PRK13982	cl27193	bifunctional SbtC-like/phosphopantothenoylcysteine decarboxylase/phosphopantothenate synthase
	non-specific	1	169	5.76e-36	PRK07313	PRK07313	cl19190	phosphopantothenoylcysteine decarboxylase
	superfamily	1	169	5.76e-36	cl19190	Flavoprotein superfamily	-	Flavoprotein
	specific	1	173	3.14e-34	pfam02441	Flavoprotein	cl19190	Flavoprotein
	non-specific	169	340	8.87e-30	pfam04127	DFP	cl27193	DNA / pantothenate metabolism flavoprotein
	non-specific	2	169	6.90e-26	TIGR02113	coaC_strep	cl19190	phosphopantothenoylcysteine decarboxylase, streptococcal

	non-specific	2	176	1.45e-19	PLN02496	PLN02496	cl19190	probable phosphopantothenoylcysteine decarboxylase
	non-specific	176	368	2.41e-07	TIGR02114	coaB_strep	cl27193	phosphopantothenate--cysteine ligase, streptococcal
	non-specific	176	368	1.73e-05	PRK06732	PRK06732	cl27193	phosphopantothenate--cysteine ligase
	non-specific	176	342	6.05e-05	PRK09620	PRK09620	cl27193	hypothetical protein
M. testudinis	non-specific	17	379	2.14e-53	PRK05579	PRK05579	cl27193	bifunctional phosphopantothenoylcysteine decarboxylase/phosphopantothenate synthase
	superfamily	17	379	2.14e-53	cl27193	DFP superfamily	-	DNA / pantothenate metabolism flavoprotein
	non-specific	17	378	2.82e-51	TIGR00521	coaBC_dfp	cl27193	phosphopantothenoylcysteine decarboxylase / phosphopantothenate--cysteine ligase
	specific	17	377	1.07e-45	COG0452	CoaBC	cl27193	Phosphopantothenoylcysteine synthetase/decarboxylase
	non-specific	25	379	1.75e-33	PRK13982	PRK13982	cl27193	bifunctional SbtC-like/phosphopantothenoylcysteine decarboxylase/phosphopantothenate synthase
	non-specific	15	196	1.91e-28	PRK07313	PRK07313	cl19190	phosphopantothenoylcysteine decarboxylase
M. testudinis	superfamily	15	196	1.91e-28	cl19190	Flavoprotein superfamily	-	Flavoprotein
	specific	17	162	3.39e-22	pfam02441	Flavoprotein	cl19190	Flavoprotein
	non-specific	16	192	7.99e-14	TIGR02113	coaC_strep	cl19190	phosphopantothenoylcysteine decarboxylase, streptococcal
	non-specific	18	140	7.92e-09	PLN02496	PLN02496	cl19190	probable phosphopantothenoylcysteine decarboxylase
	non-specific	196	327	1.21e-06	pfam04127	DFP	cl27193	DNA / pantothenate metabolism flavoprotein
	non-specific	199	377	0.000432	TIGR02114	coaB_strep	cl27193	phosphopantothenate--cysteine ligase, streptococcal
	non-specific	94	161	0.001286	PRK08305	spoVFB	cl19190	dipicolinate synthase subunit B
	non-specific	200	377	0.003129	PRK06732	PRK06732	cl27193	phosphopantothenate--cysteine ligase
M. iowae (PPCDC)	non-specific	3	177	9.47e-70	PRK07313	PRK07313	cl19190	phosphopantothenoylcysteine decarboxylase
	superfamily	3	177	9.47e-70	cl19190	Flavoprotein superfamily	-	Flavoprotein
	non-specific	1	177	6.25e-64	PRK05579	PRK05579	cl27193	bifunctional phosphopantothenoylcysteine decarboxylase/phosphopantothenate synthase
	superfamily	1	177	6.25e-64	cl27193	DFP superfamily	-	DNA / pantothenate metabolism flavoprotein
	non-specific	3	176	3.91e-58	TIGR02113	coaC_strep	cl19190	phosphopantothenoylcysteine decarboxylase, streptococcal
	specific	1	177	1.86e-53	COG0452	CoaBC	cl27193	Phosphopantothenoylcysteine synthetase/decarboxylase

	non-specific	2	171	1.76e-51	TIGR00521	coaBC_dfp	cl27193	phosphopantothenoylcysteine decarboxylase / phosphopantothenate--cysteine ligase
	specific	3	174	2.56e-41	pfam02441	Flavoprotein	cl19190	Flavoprotein
	non-specific	3	165	6.77e-25	PRK13982	PRK13982	cl27193	bifunctional SbtC-like/phosphopantothenoylcysteine decarboxylase/phosphopantothenate synthase
	non-specific	5	166	6.90e-24	PLN02496	PLN02496	cl19190	probable phosphopantothenoylcysteine decarboxylase
	non-specific	1	174	1.40e-07	COG0163	UbiX	cl19190	3-polyprenyl-4-hydroxybenzoate decarboxylase
	non-specific	79	176	3.10e-06	COG1036	COG1036	cl27425	Archaeal flavoprotein
	superfamily	79	176	3.10e-06	cl27425	COG1036 superfamily	-	Archaeal flavoprotein
	non-specific	79	121	2.99e-05	TIGR02699	archaeo_AfpA	cl27425	archaeoflavoprotein AfpA
	non-specific	79	174	3.73e-05	TIGR00421	ubiX_pad	cl19190	polyprenyl P-hydroxybenzoate and phenylacrylic acid decarboxylases
	non-specific	2	101	0.000127	PRK08305	spoVFB	cl19190	dipicolinate synthase subunit B
	non-specific	78	149	0.00026	PRK06029	PRK06029	cl19190	3-octaprenyl-4-hydroxybenzoate carboxy-lyase
	non-specific	1	115	0.000576	PRK05920	PRK05920	cl19190	aromatic acid decarboxylase
M. iowae (PPCS)	non-specific	1	235	4.40e-46	PRK06732	PRK06732	cl27193	phosphopantothenate--cysteine ligase
	superfamily	1	235	4.40e-46	cl27193	DFP superfamily	-	DNA / pantothenate metabolism flavoprotein
	non-specific	2	235	2.55e-32	TIGR02114	coaB_strep	cl27193	phosphopantothenate--cysteine ligase, streptococcal
	non-specific	2	233	4.06e-16	PRK09620	PRK09620	cl27193	hypothetical protein
	non-specific	2	194	6.27e-13	pfam04127	DFP	cl27193	DNA / pantothenate metabolism flavoprotein
	non-specific	2	194	9.56e-12	PRK05579	PRK05579	cl27193	bifunctional phosphopantothenoylcysteine decarboxylase/phosphopantothenate synthase
	non-specific	1	210	5.70e-10	COG0452	CoaBC	cl27193	Phosphopantothenoylcysteine synthetase/decarboxylase
	non-specific	2	194	7.80e-10	TIGR00521	coaBC_dfp	cl27193	phosphopantothenoylcysteine decarboxylase / phosphopantothenate--cysteine ligase
	non-specific	1	112	0.005721	COG0451	WcaG	cl25660	Nucleoside-diphosphate-sugar epimerase
	superfamily	1	112	0.005721	cl25660	Epimerase superfamily	-	NAD dependent epimerase/dehydratase family

Supplementary Table 8 CoaBC InterPro results

Mycoplasma species	Amino acid region	Database	Database ID	Database signature description	ID region start	ID region end	InterPro ID[b]	ID type[a]	Gene Ontology (GO) term[c]
M. anatis	375	Gene3D	G3DSA:3.40.50.1950		1	165	IPR036551	H	GO:0003824
		Gene3D	G3DSA:3.40.50.10300		166	375	IPR035929	H	
		PANTHER	PTHR14359:SF6		2	334			
		PANTHER	PTHR14359		2	334			
		Pfam	PF02441	Flavoprotein	1	163	IPR003382	D	GO:0003824
		Pfam	PF04127	DNA / pantothenate metabolism flavoprotein	170	335	IPR007085	D	
		Phobius	SIGNAL_PEPTIDE_N_REGION	N-terminal region of a signal peptide.	1	2			
		Phobius	SIGNAL_PEPTIDE_H_REGION	Hydrophobic region of a signal peptide.	3	13			
		Phobius	SIGNAL_PEPTIDE_C_REGION	C-terminal region of a signal peptide.	14	18			
		Phobius	SIGNAL_PEPTIDE	Signal peptide region	1	18			
		Phobius	NON_CYTOPLASMIC_D	Region of a membrane-bound protein predicted to be outside the membrane, in the extracellular region.	19	375			
		SUPERFAMILY	SSF52507		1	165	IPR036551	H	GO:0003824
		SUPERFAMILY	SSF102645		170	373	IPR035929	H	
		TIGRFAM	TIGR00521	coaBC_dfp: phosphopantothenoylcysteine decarboxylase / phosphopantothenate--cysteine ligase	1	371	IPR005252	F	GO:0004632; GO:0004633; GO:0010181; GO:0015937; GO:0015941
M. arginini	384	Gene3D	G3DSA:3.40.50.1950		1	168	IPR036551	H	GO:0003824
		Gene3D	G3DSA:3.40.50.10300		169	384	IPR035929	H	
		PANTHER	PTHR14359		2	344			
		PANTHER	PTHR14359:SF6		2	344			
		Pfam	PF04127	DNA / pantothenate metabolism flavoprotein	176	342	IPR007085	D	
		Pfam	PF02441	Flavoprotein	1	171	IPR003382	D	GO:0003824
		Phobius	NON_CYTOPLASMIC_D	Region of a membrane-bound protein predicted to be outside the membrane, in the extracellular region.	17	384			
		Phobius	SIGNAL_PEPTIDE_C_REGION	C-terminal region of a signal peptide.	12	16			
		Phobius	SIGNAL_PEPTIDE	Signal peptide region	1	16			
		Phobius	SIGNAL_PEPTIDE_N_REGION	N-terminal region of a signal peptide.	1	2			
		Phobius	SIGNAL_PEPTIDE_H_REGION	Hydrophobic region of a signal peptide.	3	11			
		SignalP_EUK	SignalP-noTM		1	17			
		SUPERFAMILY	SSF102645		176	381	IPR035929	H	
		SUPERFAMILY	SSF52507		1	169	IPR036551	H	GO:0003824
		TIGRFAM	TIGR00521	coaBC_dfp: phosphopantothenoylcysteine decarboxylase / phosphopantothenate--cysteine ligase	1	376	IPR005252	F	GO:0004632; GO:0004633; GO:0010181; GO:0015937; GO:0015941

Mycoplasma species	Amino acid region	Database	Database ID	Database signature description	ID region start	ID region end	InterPro ID[b]	ID type[a]	Gene Ontology (GO) term[c]
M. columborale	377	Gene3D	G3DSA:3.40.50.10300		165	376	IPR035929	H	
		Gene3D	G3DSA:3.40.50.1950		1	164	IPR036551	H	GO:0003824
		PANTHER	PTHR14359:SF6		2	341			
		PANTHER	PTHR14359		2	341			
		Pfam	PF04127	DNA / pantothenate metabolism flavoprotein	171	335	IPR007085	D	
		Pfam	PF02441	Flavoprotein	1	167	IPR003382	D	GO:0003824
		Phobius	SIGNAL_PEPTIDE	Signal peptide region	1	16			
		Phobius	SIGNAL_PEPTIDE_C_REGION	C-terminal region of a signal peptide.	12	16			
		Phobius	SIGNAL_PEPTIDE_N_REGION	N-terminal region of a signal peptide.	1	2			
		Phobius	SIGNAL_PEPTIDE_H_REGION	Hydrophobic region of a signal peptide.	3	11			
		Phobius	NON_CYTOPLASMIC_D	Region of a membrane-bound protein predicted to be outside the membrane, in the extracellular region.	17	377			
		SUPERFAMILY	SSF52507		1	166	IPR036551	H	GO:0003824
		SUPERFAMILY	SSF102645		170	373	IPR035929	H	
		TIGRFAM	TIGR00521	coaBC_dfp: phosphopantothenoylcysteine decarboxylase / phosphopantothenate--cysteine ligase	1	370	IPR005252	F	GO:0004632; GO:0004633; GO:0010181; GO:0015937; GO:0015941
M. cricetuli	377	Gene3D	G3DSA:3.40.50.10300		169	377	IPR035929	H	
		Gene3D	G3DSA:3.40.50.1950		1	168	IPR036551	H	GO:0003824
		PANTHER	PTHR14359		2	344			
		PANTHER	PTHR14359:SF6		2	344			
		Pfam	PF02441	Flavoprotein	1	167	IPR003382	D	GO:0003824
		Pfam	PF04127	DNA / pantothenate metabolism flavoprotein	169	336	IPR007085	D	
		Phobius	SIGNAL_PEPTIDE	Signal peptide region	1	16			
		Phobius	SIGNAL_PEPTIDE_C_REGION	C-terminal region of a signal peptide.	12	16			
		Phobius	NON_CYTOPLASMIC_D	Region of a membrane-bound protein predicted to be outside the membrane, in the extracellular region.	17	377			
		Phobius	SIGNAL_PEPTIDE_H_REGION	Hydrophobic region of a signal peptide.	3	11			
		Phobius	SIGNAL_PEPTIDE_N_REGION	N-terminal region of a signal peptide.	1	2			
		SUPERFAMILY	SSF102645		169	376	IPR035929	H	
		SUPERFAMILY	SSF52507		1	167	IPR036551	H	GO:0003824
		TIGRFAM	TIGR00521	coaBC_dfp: phosphopantothenoylcysteine decarboxylase / phosphopantothenate--cysteine ligase	1	371	IPR005252	F	GO:0004632; GO:0004633; GO:0010181; GO:0015937; GO:0015941
M. gallinaceum	385	Gene3D	G3DSA:3.40.50.1950		1	176	IPR036551	H	GO:0003824
		Gene3D	G3DSA:3.40.50.10300		177	385	IPR035929	H	
		PANTHER	PTHR14359		2	342			
		PANTHER	PTHR14359:SF6		2	342			

Mycoplasma species	Amino acid region	Database	Database ID	Database signature description	ID region start	ID region end	InterPro ID[b]	ID type[a]	Gene Ontology (GO) term[c]
M. gallinaceum	385	Pfam	PF04127	DNA / pantothenate metabolism flavoprotein	178	348	IPR007085	D	
		Pfam	PF02441	Flavoprotein	1	173	IPR003382	D	GO:0003824
		SUPERFAMILY	SSF52507		1	173	IPR036551	H	GO:0003824
		SUPERFAMILY	SSF102645		178	382	IPR035929	H	
		TIGRFAM	TIGR00521	coaBC_dfp: phosphopantothenoylcysteine decarboxylase / phosphopantothenate--cysteine ligase	1	377	IPR005252	F	GO:0004632; GO:0004633; GO:0010181; GO:0015937; GO:0015941
M. iowae (PPCS)	236	Gene3D	G3DSA:3.40.50.10300		1	236	IPR035929	H	
		PANTHER	PTHR14359:SF22		1	226			
		PANTHER	PTHR14359		1	226			
		Pfam	PF04127	DNA / pantothenate metabolism flavoprotein	2	195	IPR007085	D	
		SUPERFAMILY	SSF102645		1	232	IPR035929	H	
M. iowae (PPCDC)	178	Gene3D	G3DSA:3.40.50.1950		1	177	IPR036551	H	GO:0003824
		PANTHER	PTHR14359		2	174			
		PANTHER	PTHR14359:SF6		2	174			
		Pfam	PF02441	Flavoprotein	3	175	IPR003382	D	GO:0003824
		ProSiteProfiles	PS51257	Prokaryotic membrane lipoprotein lipid attachment site profile.	1	15			
		SUPERFAMILY	SSF52507		2	175	IPR036551	H	GO:0003824
M. mobile	360	Gene3D	G3DSA:3.40.50.1950		1	171	IPR036551	H	GO:0003824
		Gene3D	G3DSA:3.40.50.10300		172	230	IPR035929	H	
		Gene3D	G3DSA:3.40.50.10300		231	360	IPR035929	H	
		PANTHER	PTHR14359		3	323			
		PANTHER	PTHR14359:SF6		3	323			
		Pfam	PF02441	Flavoprotein	2	164	IPR003382	D	GO:0003824
		Pfam	PF04127	DNA / pantothenate metabolism flavoprotein	175	320	IPR007085	D	
		SUPERFAMILY	SSF102645		172	358	IPR035929	H	
		SUPERFAMILY	SSF52507		2	170	IPR036551	H	GO:0003824
M. sturni	376	Gene3D	G3DSA:3.40.50.1950		1	165	IPR036551	H	GO:0003824
		Gene3D	G3DSA:3.40.50.10300		166	376	IPR035929	H	
		PANTHER	PTHR14359:SF6		2	334			
		PANTHER	PTHR14359		2	334			
		Pfam	PF02441	Flavoprotein	1	164	IPR003382	D	GO:0003824
		Pfam	PF04127	DNA / pantothenate metabolism flavoprotein	170	334	IPR007085	D	
		Phobius	SIGNAL_PEPTIDE_N_REGION	N-terminal region of a signal peptide.	1	2			
		Phobius	SIGNAL_PEPTIDE_C_REGION	C-terminal region of a signal peptide.	15	18			
		Phobius	SIGNAL_PEPTIDE	Signal peptide region	1	18			
		Phobius	NON_CYTOPLASMIC_D	Region of a membrane-bound protein predicted to be outside the membrane, in the extracellular region.	19	376			
		Phobius	SIGNAL_PEPTIDE_H_REGION	Hydrophobic region of a signal peptide.	3	14			

Mycoplasma species	Amino acid region	Database	Database ID	Database signature description	ID region start	ID region end	InterPro ID[b]	ID type[a]	Gene Ontology (GO) term[c]
M. sturni	376	SUPERFAMILY	SSF102645		170	375	IPR035929	H	
		SUPERFAMILY	SSF52507		1	164	IPR036551	H	GO:0003824
		TIGRFAM	TIGR00521	coaBC_dfp: phosphopantothenoylcysteine decarboxylase / phosphopantothenate--cysteine ligase	2	370	IPR005252	F	GO:0004632; GO:0004633; GO:0010181; GO:0015937; GO:0015941
M. synoviae	384	Gene3D	G3DSA:3.40.50.10300		169	384	IPR035929	H	
		Gene3D	G3DSA:3.40.50.1950		1	168	IPR036551	H	GO:0003824
		PANTHER	PTHR14359:SF6		2	344			
		PANTHER	PTHR14359		2	344			
		Pfam	PF04127	DNA / pantothenate metabolism flavoprotein	176	342	IPR007085	D	
		Pfam	PF02441	Flavoprotein	1	171	IPR003382	D	GO:0003824
		Phobius	SIGNAL_PEPTIDE_H_REGION	Hydrophobic region of a signal peptide.	3	11			
		Phobius	SIGNAL_PEPTIDE	Signal peptide region	1	16			
		Phobius	SIGNAL_PEPTIDE_N_REGION	N-terminal region of a signal peptide.	1	2			
		Phobius	NON_CYTOPLASMIC_D	Region of a membrane-bound protein predicted to be outside the membrane, in the extracellular region.	17	384			
		Phobius	SIGNAL_PEPTIDE_C_REGION	C-terminal region of a signal peptide.	12	16			
		SignalP_EUK	SignalP-noTM		1	17			
		SUPERFAMILY	SSF102645		176	381	IPR035929	H	
		SUPERFAMILY	SSF52507		1	169	IPR036551	H	GO:0003824
		TIGRFAM	TIGR00521	coaBC_dfp: phosphopantothenoylcysteine decarboxylase / phosphopantothenate--cysteine ligase	1	376	IPR005252	F	GO:0004632; GO:0004633; GO:0010181; GO:0015937; GO:0015941
M. testudinis	380	Gene3D	G3DSA:3.40.50.1950		13	195	IPR036551	H	GO:0003824
		Gene3D	G3DSA:3.40.50.10300		196	246	IPR035929	H	
		Gene3D	G3DSA:3.40.50.10300		247	380	IPR035929	H	
		PANTHER	PTHR14359		17	242			
		Pfam	PF02441	Flavoprotein	17	186	IPR003382	D	GO:0003824
		Pfam	PF04127	DNA / pantothenate metabolism flavoprotein	196	347	IPR007085	D	
		SUPERFAMILY	SSF52507		17	190	IPR036551	H	GO:0003824
		SUPERFAMILY	SSF102645		196	379	IPR035929	H	

[a]ID type abbreviations – H, Homologous superfamily; F, Family; D, Domain

[b]InterPro ID – IPR036551: Flavin prenyltransferase-like; IPR035929: CoaB-like superfamily; IPR005252: Coenzyme A biosynthesis bifunctional protein, CoaBC; IPR003382: Flavoprotein; IPR007085: DNA/pantothenate metabolism flavoprotein, C-terminal

[c]GO term (Biological Process) – GO:0015937: Coenzyme A biosynthetic process; GO:0015941: Pantothenate catabolic process

GO term (Molecular Function) – GO:0003824: catalytic activity; GO:0004632: Phosphopantothenate--cysteine ligase activity; GO:0004633: Phosphopantothenoylcysteine decarboxylase activity; GO:0010181: FMN binding

Supplementary Table 9 CoaBC MEME + motif locations

Mycoplasma species	*p*-value	Motif locations			
		**Motif 1**	**Motif 2**	**Motif 3**	**Motif 4**
M. anatis	2.69e-117	61-100	134-161	171-199	212-261
M. arginini	1.95e-121	66-105	138-165	175-203	216-265
M. columborale	6.19e-120	61-100	134-161	171-199	212-261
M. cricetuli	8.35e-115	61-100	134-161	171-199	212-261
M. gallinaceum	1.20e-112	68-107	141-168	178-206	219-268
M. mobile	1.77e-60	62-101	134-161	176-204	-
M. sturni	5.66e-122	61-100	134-161	171-199	212-261
M. synoviae	3.78e-121	66-105	138-165	175-205	216-265
M. testudinis	1.42e-56	84-123	157-184	198-226	-
M. iowae (PPCDC)	5.76e-35	69-108	143-170	-	-
M. iowae (PPCS)	5.34e-14	-	-	1-29	-

Supplementary Table 10 PPAT CDD results

Query	Hit type	ID region start	ID region end	E-Value	Accession	Short name	Superfamily	Definition
M. agalactiae	specific	1	140	7.72e-76	PRK13964	coaD	cl00015	phosphopantetheine adenylyltransferase
	superfamily	1	140	7.72e-76	cl00015	nt_trans superfamily	-	nucleotidyl transferase superfamily
	non-specific	1	134	1.14e-44	COG0669	CoaD	cl00015	Phosphopantetheine adenylyltransferase
	non-specific	4	133	1.13e-40	cd02163	PPAT	cl00015	Phosphopantetheine adenylyltransferase
	non-specific	4	133	5.88e-39	TIGR01510	coaD_prev_kdtB	cl00015	pantetheine-phosphate adenylyltransferase
	non-specific	1	133	6.60e-38	PRK00168	coaD	cl00015	phosphopantetheine adenylyltransferase
	specific	5	135	8.39e-18	pfam01467	CTP_transf_like	cl00015	Cytidylyltransferase-like
	non-specific	4	133	1.35e-09	cd02039	cytidylyltransferase_like	cl00015	Cytidylyltransferase-like domain
	non-specific	4	60	1.67e-07	TIGR00125	cyt_tran_rel	cl00015	cytidyltransferase-like domain
	non-specific	4	66	1.13e-06	cd02165	NMNAT	cl00015	Nicotinamide/nicotinate mononucleotide adenylyltransferase
	non-specific	1	51	2.22e-06	PRK00071	nadD	cl00015	nicotinic acid mononucleotide adenylyltransferase
	non-specific	1	66	1.31e-05	PRK07152	nadD	cl28367	putative nicotinate-nucleotide adenylyltransferase
	superfamily	1	66	1.31e-05	cl28367	nadD superfamily	-	putative nicotinate-nucleotide adenylyltransferase
	non-specific	4	60	2.15e-05	cd02156	nt_trans	cl00015	nucleotidyl transferase superfamily

Query	Hit type	ID region start	ID region end	E-Value	Accession	Short name	Superfamily	Definition
	non-specific	5	51	2.74e-05	TIGR00482	TIGR00482	cl00015	nicotinate (nicotinamide) nucleotide adenylyltransferase
	non-specific	1	87	4.49e-05	cd02170	cytidylyltransferase	cl00015	cytidylyltransferase
	non-specific	1	41	4.94e-05	COG1057	NadD	cl00015	Nicotinic acid mononucleotide adenylyltransferase
	non-specific	1	59	0.000196	COG1056	NadR	cl00015	Nicotinamide mononucleotide adenylyltransferase
	non-specific	5	73	0.00042	cd02167	NMNAT_NadR	cl00015	Nicotinamide/nicotinate mononucleotide adenylyltransferase
	non-specific	14	39	0.000487	pfam02569	Pantoate_ligase	cl00015	Pantoate-beta-alanine ligase
	non-specific	1	68	0.002358	cd02171	G3P_Cytidylyltransferase	cl00015	glycerol-3-phosphate cytidylyltransferase
	non-specific	13	39	0.002895	PRK00380	panC	cl00015	pantoate--beta-alanine ligase
	non-specific	8	36	0.005163	COG0615	TagD	cl00015	Glycerol-3-phosphate cytidylyltransferase, cytidylyltransferase family
	non-specific	5	42	0.00664	cd02173	ECT	cl00015	CTP:phosphoethanolamine cytidylyltransferase (ECT)
	non-specific	14	67	0.006924	cd00560	PanC	cl00015	Pantoate-beta-alanine ligase
	non-specific	4	35	0.00929	PRK01153	PRK01153	cl00015	nicotinamide-nucleotide adenylyltransferase
	non-specific	14	39	0.009687	COG0414	PanC	cl00015	Panthothenate synthetase
	non-specific	5	145	6.82e-61	PRK13964	coaD	cl00015	phosphopantetheine adenylyltransferase
	superfamily	5	145	6.82e-61	cl00015	nt_trans superfamily	-	nucleotidyl transferase superfamily
	non-specific	4	139	5.23e-43	COG0669	CoaD	cl00015	Phosphopantetheine adenylyltransferase
	non-specific	6	138	3.36e-39	cd02163	PPAT	cl00015	Phosphopantetheine adenylyltransferase
M. alligatoris	non-specific	5	138	4.23e-37	PRK00168	coaD	cl00015	phosphopantetheine adenylyltransferase
	non-specific	6	138	4.91e-36	TIGR01510	coaD_prev_kdtB	cl00015	pantetheine-phosphate adenylyltransferase
	specific	8	140	4.61e-19	pfam01467	CTP_transf_like	cl00015	Cytidylyltransferase-like
	non-specific	6	138	2.11e-11	cd02039	cytidylyltransferase_like	cl00015	Cytidylyltransferase-like domain
	non-specific	6	65	1.92e-09	TIGR00125	cyt_tran_rel	cl00015	cytidyltransferase-like domain
	non-specific	8	138	3.57e-08	cd02167	NMNAT_NadR	cl00015	Nicotinamide/nicotinate mononucleotide adenylyltransferase
	non-specific	1	55	3.89e-08	PRK00071	nadD	cl00015	nicotinic acid mononucleotide adenylyltransferase
	non-specific	7	145	1.33e-06	cd02165	NMNAT	cl00015	Nicotinamide/nicotinate mononucleotide adenylyltransferase
	non-specific	2	55	7.19e-06	COG1057	NadD	cl00015	Nicotinic acid mononucleotide adenylyltransferase
	non-specific	6	61	8.85e-06	cd02156	nt_trans	cl00015	nucleotidyl transferase superfamily
	non-specific	8	74	1.38e-05	TIGR00482	TIGR00482	cl00015	nicotinate (nicotinamide) nucleotide adenylyltransferase
	non-specific	4	71	1.89e-05	PRK07152	nadD	cl28367	putative nicotinate-nucleotide adenylyltransferase
	superfamily	4	71	1.89e-05	cl28367	nadD superfamily	-	putative nicotinate-nucleotide adenylyltransferase
M. alligatoris	non-specific	1	139	1.92e-05	PRK08099	PRK08099	cl28365	bifunctional DNA-binding transcriptional repressor/ NMN adenylyltransferase
	superfamily	1	139	1.92e-05	cl28365	PRK08099 superfamily	-	bifunctional DNA-binding transcriptional repressor/ NMN adenylyltransferase
	non-specific	8	138	2.08e-05	TIGR01526	nadR_NMN_Atrans	cl28365	nicotinamide-nucleotide adenylyltransferase
	non-specific	3	76	4.92e-05	COG1056	NadR	cl00015	Nicotinamide mononucleotide adenylyltransferase
	non-specific	5	35	0.000102	PRK00777	PRK00777	cl00015	phosphopantetheine adenylyltransferase
	non-specific	8	45	0.000144	cd02174	CCT	cl00015	CTP:phosphocholine cytidylyltransferase
	non-specific	11	44	0.000385	TIGR02199	rfaE_dom_II	cl00015	rfaE bifunctional protein, domain II
	non-specific	5	39	0.000474	COG0615	TagD	cl00015	Glycerol-3-phosphate cytidylyltransferase, cytidylyltransferase family
	non-specific	5	39	0.000767	cd02170	cytidylyltransferase	cl00015	cytidylyltransferase

Query	Hit type	ID region start	ID region end	E-Value	Accession	Short name	Superfamily	Definition
	non-specific	1	36	0.001749	COG1019	CAB4	cl00015	Phosphopantetheine adenylyltransferase
	non-specific	6	45	0.002804	cd02173	ECT	cl00015	CTP:phosphoethanolamine cytidylyltransferase (ECT)
	non-specific	11	44	0.002804	COG2870	RfaE	cl28454	ADP-heptose synthase, bifunctional sugar kinase/adenylyltransferase
	superfamily	11	44	0.002804	cl28454	RfaE superfamily	-	ADP-heptose synthase, bifunctional sugar kinase/adenylyltransferase
	non-specific	7	38	0.003547	PRK05379	PRK05379	cl28366	bifunctional nicotinamide mononucleotide adenylyltransferase
	superfamily	7	38	0.003547	cl28366	PRK05379 superfamily	-	bifunctional nicotinamide mononucleotide adenylyltransferase
	non-specific	5	46	0.003932	cd02171	G3P_Cytidylyltransferase	cl00015	glycerol-3-phosphate cytidylyltransferase
	non-specific	11	43	0.004677	PRK11316	PRK11316	cl28454	bifunctional heptose 7-phosphate kinase/heptose 1-phosphate adenyltransferase
	non-specific	5	136	1.05e-34	cd02163	PPAT	cl00015	Phosphopantetheine adenylyltransferase
	superfamily	5	136	1.05e-34	cl00015	nt_trans superfamily	-	nucleotidyl transferase superfamily
	non-specific	3	136	1.63e-33	COG0669	CoaD	cl00015	Phosphopantetheine adenylyltransferase
	non-specific	3	136	1.11e-31	PRK00168	coaD	cl00015	phosphopantetheine adenylyltransferase
M. alvi	non-specific	5	136	1.40e-31	TIGR01510	coaD_prev_kdtB	cl00015	pantetheine-phosphate adenylyltransferase
	non-specific	3	147	1.40e-27	PRK13964	coaD	cl00015	phosphopantetheine adenylyltransferase
	non-specific	7	137	4.55e-14	pfam01467	CTP_transf_like	cl00015	Cytidylyltransferase-like
	non-specific	5	37	3.10e-10	TIGR00125	cyt_tran_rel	cl00015	cytidyltransferase-like domain
	non-specific	1	40	2.38e-09	COG1057	NadD	cl00015	Nicotinic acid mononucleotide adenylyltransferase
	non-specific	1	38	4.96e-09	PRK00071	nadD	cl00015	nicotinic acid mononucleotide adenylyltransferase
	non-specific	6	37	1.43e-07	cd02166	NMNAT_Archaea	cl00015	Nicotinamide/nicotinate mononucleotide adenylyltransferase
	non-specific	1	37	1.53e-07	COG1056	NadR	cl00015	Nicotinamide mononucleotide adenylyltransferase
	non-specific	5	40	2.48e-07	cd02165	NMNAT	cl00015	Nicotinamide/nicotinate mononucleotide adenylyltransferase
	non-specific	6	37	5.59e-07	PRK01153	PRK01153	cl00015	nicotinamide-nucleotide adenylyltransferase
	non-specific	3	45	1.01e-06	PRK07152	nadD	cl28367	putative nicotinate-nucleotide adenylyltransferase
	superfamily	3	45	1.01e-06	cl28367	nadD superfamily	-	putative nicotinate-nucleotide adenylyltransferase
	non-specific	6	60	3.31e-06	cd02156	nt_trans	cl00015	nucleotidyl transferase superfamily
	non-specific	5	136	4.91e-05	cd02039	cytidylyltransferase_like	cl00015	Cytidylyltransferase-like domain
	non-specific	7	40	0.000267	TIGR00482	TIGR00482	cl00015	nicotinate (nicotinamide) nucleotide adenylyltransferase
M. alvi	non-specific	10	38	0.000627	COG0615	TagD	cl00015	Glycerol-3-phosphate cytidylyltransferase, cytidylyltransferase family
	non-specific	10	38	0.000673	cd02170	cytidylyltransferase	cl00015	cytidylyltransferase
	non-specific	3	38	0.000929	TIGR01526	nadR_NMN_Atrans	cl28365	nicotinamide-nucleotide adenylyltransferase
	superfamily	3	38	0.000929	cl28365	PRK08099 superfamily	-	bifunctional DNA-binding transcriptional repressor/ NMN adenylyltransferase
	non-specific	4	35	0.000937	PRK06973	PRK06973	cl00015	nicotinic acid mononucleotide adenylyltransferase
	non-specific	6	37	0.001266	TIGR01527	arch_NMN_Atrans	cl00015	nicotinamide-nucleotide adenylyltransferase
	non-specific	3	43	0.001379	cd02172	RfaE_N	cl00015	N-terminal domain of RfaE
	non-specific	4	37	0.003214	PTZ00308	PTZ00308	cl28626	ethanolamine-phosphate cytidylyltransferase
	superfamily	4	37	0.003214	cl28626	PLN02406 superfamily	-	ethanolamine-phosphate cytidylyltransferase
	non-specific	5	38	0.003822	cd02167	NMNAT_NadR	cl00015	Nicotinamide/nicotinate mononucleotide adenylyltransferase

Query	Hit type	ID region start	ID region end	E-Value	Accession	Short name	Superfamily	Definition
	non-specific	1	44	0.007022	PRK08099	PRK08099	cl28365	bifunctional DNA-binding transcriptional repressor/ NMN adenylyltransferase
M. anatis	non-specific	3	142	5.32e-63	PRK13964	coaD	cl00015	phosphopantetheine adenylyltransferase
	superfamily	3	142	5.32e-63	cl00015	nt_trans superfamily	-	nucleotidyl transferase superfamily
	non-specific	2	136	8.28e-44	COG0669	CoaD	cl00015	Phosphopantetheine adenylyltransferase
	non-specific	6	135	1.09e-42	TIGR01510	coaD_prev_kdtB	cl00015	pantetheine-phosphate adenylyltransferase
	non-specific	6	133	4.40e-42	cd02163	PPAT	cl00015	Phosphopantetheine adenylyltransferase
	non-specific	3	133	1.05e-39	PRK00168	coaD	cl00015	phosphopantetheine adenylyltransferase
	specific	7	137	1.87e-18	pfam01467	CTP_transf_like	cl00015	Cytidylyltransferase-like
	non-specific	6	47	4.37e-10	TIGR00125	cyt_tran_rel	cl00015	cytidyltransferase-like domain
	non-specific	3	68	1.64e-07	PRK07152	nadD	cl28367	putative nicotinate-nucleotide adenylyltransferase
	superfamily	3	68	1.64e-07	cl28367	nadD superfamily	-	putative nicotinate-nucleotide adenylyltransferase
	non-specific	5	135	2.38e-07	cd02039	cytidylyltransferase_like	cl00015	Cytidylyltransferase-like domain
	non-specific	1	57	8.37e-07	COG1057	NadD	cl00015	Nicotinic acid mononucleotide adenylyltransferase
	non-specific	6	68	1.21e-06	cd02165	NMNAT	cl00015	Nicotinamide/nicotinate mononucleotide adenylyltransferase
	non-specific	1	53	2.42e-06	PRK00071	nadD	cl00015	nicotinic acid mononucleotide adenylyltransferase
	non-specific	1	37	4.98e-06	COG1056	NadR	cl00015	Nicotinamide mononucleotide adenylyltransferase
M. anatis	non-specific	6	51	4.50e-05	cd02156	nt_trans	cl00015	nucleotidyl transferase superfamily
	non-specific	6	37	4.67e-05	PRK01153	PRK01153	cl00015	nicotinamide-nucleotide adenylyltransferase
	non-specific	6	37	0.000133	cd02166	NMNAT_Archaea	cl00015	Nicotinamide/nicotinate mononucleotide adenylyltransferase
	non-specific	7	44	0.000544	cd02173	ECT	cl00015	CTP:phosphoethanolamine cytidylyltransferase (ECT)
	non-specific	7	46	0.000563	TIGR00482	TIGR00482	cl00015	nicotinate (nicotinamide) nucleotide adenylyltransferase
	non-specific	7	135	0.001898	cd02167	NMNAT_NadR	cl00015	Nicotinamide/nicotinate mononucleotide adenylyltransferase
	non-specific	6	37	0.00322	TIGR01527	arch_NMN_Atrans	cl00015	nicotinamide-nucleotide adenylyltransferase
	non-specific	10	88	0.003576	COG0615	TagD	cl00015	Glycerol-3-phosphate cytidylyltransferase, cytidylyltransferase family
	non-specific	10	35	0.005471	PRK00777	PRK00777	cl00015	phosphopantetheine adenylyltransferase
	non-specific	9	44	0.008923	PTZ00308	PTZ00308	cl28626	ethanolamine-phosphate cytidylyltransferase
	superfamily	9	44	0.008923	cl28626	PLN02406 superfamily	-	ethanolamine-phosphate cytidylyltransferase
M. arginini	specific	1	140	3.43e-72	PRK13964	coaD	cl00015	phosphopantetheine adenylyltransferase
	superfamily	1	140	3.43e-72	cl00015	nt_trans superfamily	-	nucleotidyl transferase superfamily
	non-specific	1	134	1.29e-46	COG0669	CoaD	cl00015	Phosphopantetheine adenylyltransferase
	non-specific	3	133	5.90e-43	cd02163	PPAT	cl00015	Phosphopantetheine adenylyltransferase
	non-specific	1	133	4.85e-40	PRK00168	coaD	cl00015	phosphopantetheine adenylyltransferase
	non-specific	3	133	2.98e-38	TIGR01510	coaD_prev_kdtB	cl00015	pantetheine-phosphate adenylyltransferase
	specific	5	133	3.14e-21	pfam01467	CTP_transf_like	cl00015	Cytidylyltransferase-like
	non-specific	3	35	4.10e-12	TIGR00125	cyt_tran_rel	cl00015	cytidyltransferase-like domain
	non-specific	3	133	2.72e-11	cd02039	cytidylyltransferase_like	cl00015	Cytidylyltransferase-like domain
	non-specific	1	66	6.91e-09	COG1057	NadD	cl00015	Nicotinic acid mononucleotide adenylyltransferase
	non-specific	5	133	6.44e-08	cd02167	NMNAT_NadR	cl00015	Nicotinamide/nicotinate mononucleotide adenylyltransferase
	non-specific	3	39	7.00e-08	cd02156	nt_trans	cl00015	nucleotidyl transferase superfamily
	non-specific	4	66	7.56e-08	cd02165	NMNAT	cl00015	Nicotinamide/nicotinate mononucleotide adenylyltransferase
	non-specific	1	41	2.06e-07	PRK00071	nadD	cl00015	nicotinic acid mononucleotide adenylyltransferase

Query	Hit type	ID region start	ID region end	E-Value	Accession	Short name	Superfamily	Definition
	non-specific	1	36	3.33e-07	cd02170	cytidylyltransferase	cl00015	cytidylyltransferase
	non-specific	1	85	4.47e-07	COG0615	TagD	cl00015	Glycerol-3-phosphate cytidylyltransferase, cytidylyltransferase family
	non-specific	1	43	6.80e-07	PRK07152	nadD	cl28367	putative nicotinate-nucleotide adenylyltransferase
	superfamily	1	43	6.80e-07	cl28367	nadD superfamily	-	putative nicotinate-nucleotide adenylyltransferase
	non-specific	5	62	2.09e-06	TIGR00482	TIGR00482	cl00015	nicotinate (nicotinamide) nucleotide adenylyltransferase
	non-specific	1	35	2.18e-06	COG1056	NadR	cl00015	Nicotinamide mononucleotide adenylyltransferase
	non-specific	1	33	1.71e-05	PRK00777	PRK00777	cl00015	phosphopantetheine adenylyltransferase
	non-specific	1	36	0.000131	TIGR01526	nadR_NMN_Atrans	cl28365	nicotinamide-nucleotide adenylyltransferase
	superfamily	1	36	0.000131	cl28365	PRK08099 superfamily	-	bifunctional DNA-binding transcriptional repressor/ NMN adenylyltransferase
	non-specific	1	36	0.000394	cd02171	G3P_Cytidylyltransferase	cl00015	glycerol-3-phosphate cytidylyltransferase
	non-specific	8	41	0.000535	cd02174	CCT	cl00015	CTP:phosphocholine cytidylyltransferase
	non-specific	3	35	0.001099	cd02166	NMNAT_Archaea	cl00015	Nicotinamide/nicotinate mononucleotide adenylyltransferase
	non-specific	2	35	0.001112	PRK01153	PRK01153	cl00015	nicotinamide-nucleotide adenylyltransferase
	non-specific	7	41	0.00136	PTZ00308	PTZ00308	cl28626	ethanolamine-phosphate cytidylyltransferase
M. arginini	superfamily	7	41	0.00136	cl28626	PLN02406 superfamily	-	ethanolamine-phosphate cytidylyltransferase
	non-specific	8	57	0.002223	TIGR01518	g3p_cytidyltrns	cl00015	glycerol-3-phosphate cytidylyltransferase
	non-specific	3	41	0.002408	cd02173	ECT	cl00015	CTP:phosphoethanolamine cytidylyltransferase (ECT)
	non-specific	8	134	0.008463	PRK08099	PRK08099	cl28365	bifunctional DNA-binding transcriptional repressor/ NMN adenylyltransferase
	non-specific	1	140	1.28e-52	PRK13964	coaD	cl00015	phosphopantetheine adenylyltransferase
	superfamily	1	140	1.28e-52	cl00015	nt_trans superfamily	-	nucleotidyl transferase superfamily
	non-specific	1	134	1.75e-42	COG0669	CoaD	cl00015	Phosphopantetheine adenylyltransferase
	non-specific	3	133	1.91e-40	cd02163	PPAT	cl00015	Phosphopantetheine adenylyltransferase
	non-specific	1	133	2.71e-39	PRK00168	coaD	cl00015	phosphopantetheine adenylyltransferase
	non-specific	3	133	6.95e-37	TIGR01510	coaD_prev_kdtB	cl00015	pantetheine-phosphate adenylyltransferase
	specific	5	135	7.04e-24	pfam01467	CTP_transf_like	cl00015	Cytidylyltransferase-like
	non-specific	5	133	9.44e-14	cd02167	NMNAT_NadR	cl00015	Nicotinamide/nicotinate mononucleotide adenylyltransferase
	non-specific	3	57	1.21e-12	TIGR00125	cyt_tran_rel	cl00015	cytidylyltransferase-like domain
	non-specific	3	133	1.51e-10	cd02039	cytidylyltransferase_like	cl00015	Cytidylyltransferase-like domain
M. bovigenitalium	non-specific	1	66	2.48e-10	PRK00071	nadD	cl00015	nicotinic acid mononucleotide adenylyltransferase
	non-specific	1	66	4.65e-10	COG1057	NadD	cl00015	Nicotinic acid mononucleotide adenylyltransferase
	non-specific	1	133	1.25e-09	TIGR01526	nadR_NMN_Atrans	cl28365	nicotinamide-nucleotide adenylyltransferase
	superfamily	1	133	1.25e-09	cl28365	PRK08099 superfamily	-	bifunctional DNA-binding transcriptional repressor/ NMN adenylyltransferase
	non-specific	4	66	9.14e-09	cd02165	NMNAT	cl00015	Nicotinamide/nicotinate mononucleotide adenylyltransferase
	non-specific	1	35	3.95e-08	COG1056	NadR	cl00015	Nicotinamide mononucleotide adenylyltransferase
	non-specific	5	66	4.77e-08	TIGR00482	TIGR00482	cl00015	nicotinate (nicotinamide) nucleotide adenylyltransferase
	non-specific	1	66	7.58e-08	PRK07152	nadD	cl28367	putative nicotinate-nucleotide adenylyltransferase
	superfamily	1	66	7.58e-08	cl28367	nadD superfamily	-	putative nicotinate-nucleotide adenylyltransferase
	non-specific	1	35	3.11e-07	PRK01153	PRK01153	cl00015	nicotinamide-nucleotide adenylyltransferase
	non-specific	3	67	3.94e-07	cd02166	NMNAT_Archaea	cl00015	Nicotinamide/nicotinate mononucleotide adenylyltransferase

Query	Hit type	ID region start	ID region end	E-Value	Accession	Short name	Superfamily	Definition
	non-specific	8	134	4.62e-07	PRK08099	PRK08099	cl28365	bifunctional DNA-binding transcriptional repressor/ NMN adenylyltransferase
	non-specific	3	77	2.82e-06	TIGR01527	arch_NMN_Atrans	cl00015	nicotinamide-nucleotide adenylyltransferase
	non-specific	4	50	3.24e-06	PRK05379	PRK05379	cl28366	bifunctional nicotinamide mononucleotide adenylyltransferase
	superfamily	4	50	3.24e-06	cl28366	PRK05379 superfamily	-	bifunctional nicotinamide mononucleotide adenylyltransferase
	non-specific	1	33	3.44e-05	PRK00777	PRK00777	cl00015	phosphopantetheine adenylyltransferase
	non-specific	1	58	4.60e-05	COG0615	TagD	cl00015	Glycerol-3-phosphate cytidylyltransferase, cytidylyltransferase family
M. bovigenitalium	non-specific	1	56	4.75e-05	COG1019	CAB4	cl00015	Phosphopantetheine adenylyltransferase
	non-specific	4	50	4.99e-05	cd02168	NMNAT_Nudix	cl00015	Nicotinamide/nicotinate mononucleotide adenylyltransferase
	non-specific	7	56	5.04e-05	cd02164	PPAT_CoAS	cl00015	phosphopantetheine adenylyltransferase
	non-specific	3	85	0.000344	cd02156	nt_trans	cl00015	nucleotidyl transferase superfamily
	non-specific	1	133	0.000777	cd02170	cytidylyltransferase	cl00015	cytidylyltransferase
	non-specific	5	55	0.001778	cd02174	CCT	cl00015	CTP:phosphocholine cytidylyltransferase
	non-specific	1	57	0.00191	cd02171	G3P_Cytidylyltransferase	cl00015	glycerol-3-phosphate cytidylyltransferase
	non-specific	8	140	0.005664	COG0196	RibF	cl27514	FAD synthase
	superfamily	8	140	0.005664	cl27514	Flavokinase superfamily	-	Riboflavin kinase
	non-specific	8	64	0.007382	PRK07143	PRK07143	cl27514	hypothetical protein
M. bovis	specific	1	140	7.89e-71	PRK13964	coaD	cl00015	phosphopantetheine adenylyltransferase
	superfamily	1	140	7.89e-71	cl00015	nt_trans superfamily	-	nucleotidyl transferase superfamily
	non-specific	1	139	1.50e-44	COG0669	CoaD	cl00015	Phosphopantetheine adenylyltransferase
	non-specific	4	133	6.78e-41	cd02163	PPAT	cl00015	Phosphopantetheine adenylyltransferase
	non-specific	4	133	3.43e-38	TIGR01510	coaD_prev_kdtB	cl00015	pantetheine-phosphate adenylyltransferase
	non-specific	1	133	1.34e-37	PRK00168	coaD	cl00015	phosphopantetheine adenylyltransferase
	specific	5	135	1.14e-19	pfam01467	CTP_transf_like	cl00015	Cytidylyltransferase-like
	non-specific	4	63	2.65e-11	TIGR00125	cyt_tran_rel	cl00015	cytidyltransferase-like domain
	non-specific	4	133	3.31e-11	cd02039	cytidylyltransferase_like	cl00015	Cytidylyltransferase-like domain
	non-specific	1	97	6.97e-10	COG1056	NadR	cl00015	Nicotinamide mononucleotide adenylyltransferase
	non-specific	1	66	1.18e-08	PRK07152	nadD	cl28367	putative nicotinate-nucleotide adenylyltransferase
	superfamily	1	66	1.18e-08	cl28367	nadD superfamily	-	putative nicotinate-nucleotide adenylyltransferase
	non-specific	4	66	4.64e-08	cd02165	NMNAT	cl00015	Nicotinamide/nicotinate mononucleotide adenylyltransferase
	non-specific	1	60	2.23e-07	COG1057	NadD	cl00015	Nicotinic acid mononucleotide adenylyltransferase
	non-specific	1	66	2.33e-07	PRK00071	nadD	cl00015	nicotinic acid mononucleotide adenylyltransferase
	non-specific	4	90	1.81e-06	cd02156	nt_trans	cl00015	nucleotidyl transferase superfamily
	non-specific	5	60	4.47e-06	TIGR00482	TIGR00482	cl00015	nicotinate (nicotinamide) nucleotide adenylyltransferase
	non-specific	13	39	6.07e-05	pfam02569	Pantoate_ligase	cl00015	Pantoate-beta-alanine ligase
	non-specific	5	67	6.49e-05	cd02167	NMNAT_NadR	cl00015	Nicotinamide/nicotinate mononucleotide adenylyltransferase
	non-specific	4	35	0.000109	PRK01153	PRK01153	cl00015	nicotinamide-nucleotide adenylyltransferase
	non-specific	8	36	0.000227	cd02170	cytidylyltransferase	cl00015	cytidylyltransferase
	non-specific	4	35	0.000243	cd02166	NMNAT_Archaea	cl00015	Nicotinamide/nicotinate mononucleotide adenylyltransferase
	non-specific	3	35	0.00039	PRK05379	PRK05379	cl28366	bifunctional nicotinamide mononucleotide adenylyltransferase
	superfamily	3	35	0.00039	cl28366	PRK05379 superfamily	-	bifunctional nicotinamide mononucleotide adenylyltransferase

Query	Hit type	ID region start	ID region end	E-Value	Accession	Short name	Superfamily	Definition
	non-specific	8	43	0.000479	COG0615	TagD	cl00015	Glycerol-3-phosphate cytidylyltransferase, cytidylyltransferase family
M. bovis	non-specific	8	134	0.00069	PRK08099	PRK08099	cl28365	bifunctional DNA-binding transcriptional repressor/ NMN adenylyltransferase
	superfamily	8	134	0.00069	cl28365	PRK08099 superfamily	-	bifunctional DNA-binding transcriptional repressor/ NMN adenylyltransferase
	non-specific	1	68	0.001024	cd02171	G3P_Cytidylyltransferase	cl00015	glycerol-3-phosphate cytidylyltransferase
	non-specific	4	139	0.0013	TIGR01527	arch_NMN_Atrans	cl00015	nicotinamide-nucleotide adenylyltransferase
	non-specific	13	39	0.001386	COG0414	PanC	cl00015	Panthothenate synthetase
	non-specific	13	39	0.001429	PRK00380	panC	cl00015	pantoate--beta-alanine ligase
	non-specific	13	39	0.001477	cd00560	PanC	cl00015	Pantoate-beta-alanine ligase
	non-specific	1	49	0.001604	PRK00777	PRK00777	cl00015	phosphopantetheine adenylyltransferase
	non-specific	8	69	0.001618	COG1019	CAB4	cl00015	Phosphopantetheine adenylyltransferase
	non-specific	1	133	0.001948	TIGR01526	nadR_NMN_Atrans	cl28365	nicotinamide-nucleotide adenylyltransferase
	non-specific	5	42	0.002332	cd02173	ECT	cl00015	CTP:phosphoethanolamine cytidylyltransferase (ECT)
	non-specific	8	52	0.002498	PRK11316	PRK11316	cl28454	bifunctional heptose 7-phosphate kinase/heptose 1-phosphate adenyltransferase
	superfamily	8	52	0.002498	cl28454	RfaE superfamily	-	ADP-heptose synthase, bifunctional sugar kinase/adenylyltransferase
	non-specific	13	39	0.002915	PLN02660	PLN02660	cl00015	pantoate--beta-alanine ligase
	non-specific	8	68	0.004536	TIGR01518	g3p_cytidyltrns	cl00015	glycerol-3-phosphate cytidylyltransferase
	non-specific	7	41	0.005859	PTZ00308	PTZ00308	cl28626	ethanolamine-phosphate cytidylyltransferase
	superfamily	7	41	0.005859	cl28626	PLN02406 superfamily	-	ethanolamine-phosphate cytidylyltransferase
M. buteonis	non-specific	5	145	1.43e-47	PRK13964	coaD	cl00015	phosphopantetheine adenylyltransferase
	superfamily	5	145	1.43e-47	cl00015	nt_trans superfamily	-	nucleotidyl transferase superfamily
	non-specific	4	141	4.02e-41	COG0669	CoaD	cl00015	Phosphopantetheine adenylyltransferase
	non-specific	7	139	2.75e-38	cd02163	PPAT	cl00015	Phosphopantetheine adenylyltransferase
	non-specific	7	139	7.00e-37	TIGR01510	coaD_prev_kdtB	cl00015	pantetheine-phosphate adenylyltransferase
	non-specific	5	139	1.28e-36	PRK00168	coaD	cl00015	phosphopantetheine adenylyltransferase
	specific	9	139	8.26e-18	pfam01467	CTP_transf_like	cl00015	Cytidylyltransferase-like
	non-specific	7	48	5.44e-08	TIGR00125	cyt_tran_rel	cl00015	cytidyltransferase-like domain
	non-specific	3	51	1.76e-07	COG1057	NadD	cl00015	Nicotinic acid mononucleotide adenylyltransferase
	non-specific	4	52	1.23e-06	PRK00071	nadD	cl00015	nicotinic acid mononucleotide adenylyltransferase
	non-specific	7	139	2.50e-06	cd02039	cytidylyltransferase_like	cl00015	Cytidylyltransferase-like domain
	non-specific	5	70	4.51e-06	PRK07152	nadD	cl28367	putative nicotinate-nucleotide adenylyltransferase
	superfamily	5	70	4.51e-06	cl28367	nadD superfamily	-	putative nicotinate-nucleotide adenylyltransferase
	non-specific	7	43	2.45e-05	cd02165	NMNAT	cl00015	Nicotinamide/nicotinate mononucleotide adenylyltransferase
	non-specific	9	47	0.000178	TIGR00482	TIGR00482	cl00015	nicotinate (nicotinamide) nucleotide adenylyltransferase
	non-specific	5	39	0.000485	COG1056	NadR	cl00015	Nicotinamide mononucleotide adenylyltransferase
	non-specific	8	124	0.001859	cd02156	nt_trans	cl00015	nucleotidyl transferase superfamily
	non-specific	8	39	0.007177	cd02166	NMNAT_Archaea	cl00015	Nicotinamide/nicotinate mononucleotide adenylyltransferase
	non-specific	5	40	0.007214	PRK00777	PRK00777	cl00015	phosphopantetheine adenylyltransferase
	non-specific	8	39	0.007417	PRK01153	PRK01153	cl00015	nicotinamide-nucleotide adenylyltransferase

Query	Hit type	ID region start	ID region end	E-Value	Accession	Short name	Superfamily	Definition
M. californicum	non-specific	1	140	6.19e-55	PRK13964	coaD	cl00015	phosphopantetheine adenylyltransferase
	superfamily	1	140	6.19e-55	cl00015	nt_trans superfamily	-	nucleotidyl transferase superfamily
	non-specific	1	134	1.61e-42	COG0669	CoaD	cl00015	Phosphopantetheine adenylyltransferase
	non-specific	1	133	3.31e-37	PRK00168	coaD	cl00015	phosphopantetheine adenylyltransferase
	non-specific	3	133	7.92e-37	cd02163	PPAT	cl00015	Phosphopantetheine adenylyltransferase
	non-specific	3	133	2.43e-33	TIGR01510	coaD_prev_kdtB	cl00015	pantetheine-phosphate adenylyltransferase
	specific	5	135	1.65e-20	pfam01467	CTP_transf_like	cl00015	Cytidylyltransferase-like
	non-specific	3	56	2.09e-10	TIGR00125	cyt_tran_rel	cl00015	cytidyltransferase-like domain
	non-specific	1	66	5.55e-10	PRK00071	nadD	cl00015	nicotinic acid mononucleotide adenylyltransferase
	non-specific	1	66	3.72e-09	COG1057	NadD	cl00015	Nicotinic acid mononucleotide adenylyltransferase
	non-specific	4	66	5.98e-09	cd02165	NMNAT	cl00015	Nicotinamide/nicotinate mononucleotide adenylyltransferase
	non-specific	3	133	1.16e-08	cd02039	cytidylyltransferase_like	cl00015	Cytidylyltransferase-like domain
	non-specific	5	66	4.47e-07	TIGR00482	TIGR00482	cl00015	nicotinate (nicotinamide) nucleotide adenylyltransferase
	non-specific	4	58	8.49e-07	PRK05379	PRK05379	cl28366	bifunctional nicotinamide mononucleotide adenylyltransferase
	superfamily	4	58	8.49e-07	cl28366	PRK05379 superfamily	-	bifunctional nicotinamide mononucleotide adenylyltransferase
	non-specific	1	35	1.60e-06	COG1056	NadR	cl00015	Nicotinamide mononucleotide adenylyltransferase
	non-specific	4	65	1.77e-06	cd02168	NMNAT_Nudix	cl00015	Nicotinamide/nicotinate mononucleotide adenylyltransferase
	non-specific	5	133	1.90e-06	cd02167	NMNAT_NadR	cl00015	Nicotinamide/nicotinate mononucleotide adenylyltransferase
	non-specific	1	71	1.05e-05	PRK07152	nadD	cl28367	putative nicotinate-nucleotide adenylyltransferase
	superfamily	1	71	1.05e-05	cl28367	nadD superfamily	-	putative nicotinate-nucleotide adenylyltransferase
	non-specific	1	33	6.22e-05	PRK00777	PRK00777	cl00015	phosphopantetheine adenylyltransferase
	non-specific	1	133	0.000161	TIGR01526	nadR_NMN_Atrans	cl28365	nicotinamide-nucleotide adenylyltransferase
	superfamily	1	133	0.000161	cl28365	PRK08099 superfamily	-	bifunctional DNA-binding transcriptional repressor/ NMN adenylyltransferase
	non-specific	3	35	0.000733	cd02166	NMNAT_Archaea	cl00015	Nicotinamide/nicotinate mononucleotide adenylyltransferase
	non-specific	2	35	0.001521	PRK01153	PRK01153	cl00015	nicotinamide-nucleotide adenylyltransferase
	non-specific	3	46	0.002521	TIGR01527	arch_NMN_Atrans	cl00015	nicotinamide-nucleotide adenylyltransferase
	non-specific	3	56	0.005041	cd02156	nt_trans	cl00015	nucleotidyl transferase superfamily
	non-specific	1	36	0.006027	cd02171	G3P_Cytidylyltransferase	cl00015	glycerol-3-phosphate cytidylyltransferase
M. canis	non-specific	5	139	6.25e-44	PRK13964	coaD	cl00015	phosphopantetheine adenylyltransferase
	superfamily	5	139	6.25e-44	cl00015	nt_trans superfamily	-	nucleotidyl transferase superfamily
	non-specific	5	138	6.62e-40	COG0669	CoaD	cl00015	Phosphopantetheine adenylyltransferase
	non-specific	6	138	1.24e-39	cd02163	PPAT	cl00015	Phosphopantetheine adenylyltransferase
	non-specific	5	138	7.44e-38	PRK00168	coaD	cl00015	phosphopantetheine adenylyltransferase
	non-specific	6	138	2.10e-37	TIGR01510	coaD_prev_kdtB	cl00015	pantetheine-phosphate adenylyltransferase
	specific	8	138	1.27e-19	pfam01467	CTP_transf_like	cl00015	Cytidylyltransferase-like
	non-specific	6	136	4.07e-11	cd02039	cytidylyltransferase_like	cl00015	Cytidylyltransferase-like domain
M. canis	non-specific	6	51	2.76e-10	TIGR00125	cyt_tran_rel	cl00015	cytidyltransferase-like domain
	non-specific	2	47	2.44e-08	COG1056	NadR	cl00015	Nicotinamide mononucleotide adenylyltransferase
	non-specific	6	38	9.48e-08	cd02166	NMNAT_Archaea	cl00015	Nicotinamide/nicotinate mononucleotide adenylyltransferase
	non-specific	5	40	2.80e-07	PRK01153	PRK01153	cl00015	nicotinamide-nucleotide adenylyltransferase
	non-specific	7	68	3.94e-07	cd02165	NMNAT	cl00015	Nicotinamide/nicotinate mononucleotide adenylyltransferase
	non-specific	1	49	7.06e-06	PRK00071	nadD	cl00015	nicotinic acid mononucleotide adenylyltransferase

Query	Hit type	ID region start	ID region end	E-Value	Accession	Short name	Superfamily	Definition
	non-specific	6	38	7.70e-06	TIGR01527	arch_NMN_Atrans	cl00015	nicotinamide-nucleotide adenylyltransferase
	non-specific	6	58	3.07e-05	cd02156	nt_trans	cl00015	nucleotidyl transferase superfamily
	non-specific	5	68	4.41e-05	COG1057	NadD	cl00015	Nicotinic acid mononucleotide adenylyltransferase
	non-specific	5	68	0.000197	PRK07152	nadD	cl28367	putative nicotinate-nucleotide adenylyltransferase
	superfamily	5	68	0.000197	cl28367	nadD superfamily	-	putative nicotinate-nucleotide adenylyltransferase
	non-specific	7	47	0.000295	PRK05379	PRK05379	cl28366	bifunctional nicotinamide mononucleotide adenylyltransferase
	superfamily	7	47	0.000295	cl28366	PRK05379 superfamily	-	bifunctional nicotinamide mononucleotide adenylyltransferase
	non-specific	1	57	0.001443	COG1019	CAB4	cl00015	Phosphopantetheine adenylyltransferase
	non-specific	11	36	0.002717	PRK00777	PRK00777	cl00015	phosphopantetheine adenylyltransferase
	non-specific	7	47	0.003878	cd02168	NMNAT_Nudix	cl00015	Nicotinamide/nicotinate mononucleotide adenylyltransferase
	non-specific	6	47	0.006711	cd02173	ECT	cl00015	CTP:phosphoethanolamine cytidylyltransferase (ECT)
	non-specific	21	62	0.008447	PTZ00272	PTZ00272	cl27733	heat shock protein 83 kDa (Hsp83)
	superfamily	21	62	0.008447	cl27733	HSP90 superfamily	-	Hsp90 protein
M. capricolum	specific	1	140	2.82e-73	PRK13964	coaD	cl00015	phosphopantetheine adenylyltransferase
	superfamily	1	140	2.82e-73	cl00015	nt_trans superfamily	-	nucleotidyl transferase superfamily
	specific	3	140	1.76e-64	TIGR01510	coaD_prev_kdtB	cl00015	pantetheine-phosphate adenylyltransferase
	non-specific	1	139	2.40e-45	COG0669	CoaD	cl00015	Phosphopantetheine adenylyltransferase
	non-specific	1	135	3.82e-44	PRK00168	coaD	cl00015	phosphopantetheine adenylyltransferase
	non-specific	3	139	1.89e-43	cd02163	PPAT	cl00015	Phosphopantetheine adenylyltransferase
	specific	5	135	5.20e-20	pfam01467	CTP_transf_like	cl00015	Cytidylyltransferase-like
	non-specific	3	63	8.10e-14	TIGR00125	cyt_tran_rel	cl00015	cytidyltransferase-like domain
	non-specific	3	134	2.49e-11	cd02039	cytidylyltransferase_like	cl00015	Cytidylyltransferase-like domain
	non-specific	1	77	2.61e-10	PRK07152	nadD	cl28367	putative nicotinate-nucleotide adenylyltransferase
	superfamily	1	77	2.61e-10	cl28367	nadD superfamily	-	putative nicotinate-nucleotide adenylyltransferase
	non-specific	1	66	1.68e-08	COG1057	NadD	cl00015	Nicotinic acid mononucleotide adenylyltransferase
	non-specific	3	77	1.43e-07	cd02165	NMNAT	cl00015	Nicotinamide/nicotinate mononucleotide adenylyltransferase
	non-specific	1	46	2.31e-07	COG1056	NadR	cl00015	Nicotinamide mononucleotide adenylyltransferase
	non-specific	1	136	2.56e-07	PRK00777	PRK00777	cl00015	phosphopantetheine adenylyltransferase
	non-specific	1	66	3.58e-07	PRK00071	nadD	cl00015	nicotinic acid mononucleotide adenylyltransferase
	non-specific	1	136	6.19e-06	COG1019	CAB4	cl00015	Phosphopantetheine adenylyltransferase
	non-specific	1	71	1.09e-05	TIGR01526	nadR_NMN_Atrans	cl28365	nicotinamide-nucleotide adenylyltransferase
M. capricolum	superfamily	1	71	1.09e-05	cl28365	PRK08099 superfamily	-	bifunctional DNA-binding transcriptional repressor/ NMN adenylyltransferase
	non-specific	3	134	1.30e-05	cd02167	NMNAT_NadR	cl00015	Nicotinamide/nicotinate mononucleotide adenylyltransferase
	non-specific	4	130	4.30e-05	cd02156	nt_trans	cl00015	nucleotidyl transferase superfamily
	non-specific	4	35	7.26e-05	cd02166	NMNAT_Archaea	cl00015	Nicotinamide/nicotinate mononucleotide adenylyltransferase
	non-specific	4	68	0.00016	TIGR01527	arch_NMN_Atrans	cl00015	nicotinamide-nucleotide adenylyltransferase
	non-specific	4	35	0.000417	PRK01153	PRK01153	cl00015	nicotinamide-nucleotide adenylyltransferase
	non-specific	3	35	0.00049	PRK05379	PRK05379	cl28366	bifunctional nicotinamide mononucleotide adenylyltransferase
	superfamily	3	35	0.00049	cl28366	PRK05379 superfamily	-	bifunctional nicotinamide mononucleotide adenylyltransferase
	non-specific	5	60	0.00283	TIGR00482	TIGR00482	cl00015	nicotinate (nicotinamide) nucleotide adenylyltransferase
	non-specific	1	36	0.003701	cd02171	G3P_Cytidylyltransferase	cl00015	glycerol-3-phosphate cytidylyltransferase
	non-specific	8	57	0.004943	cd02170	cytidylyltransferase	cl00015	cytidylyltransferase

Query	Hit type	ID region start	ID region end	E-Value	Accession	Short name	Superfamily	Definition
	non-specific	1	76	0.004984	PRK08887	PRK08887	cl00015	nicotinic acid mononucleotide adenylyltransferase
M. collis	non-specific	8	145	7.72e-53	PRK13964	coaD	cl00015	phosphopantetheine adenylyltransferase
	superfamily	8	145	7.72e-53	cl00015	nt_trans superfamily	-	nucleotidyl transferase superfamily
	non-specific	6	140	4.38e-43	COG0669	CoaD	cl00015	Phosphopantetheine adenylyltransferase
	non-specific	9	137	1.17e-42	cd02163	PPAT	cl00015	Phosphopantetheine adenylyltransferase
	non-specific	9	139	1.11e-40	TIGR01510	coaD_prev_kdtB	cl00015	pantetheine-phosphate adenylyltransferase
	non-specific	7	137	7.67e-39	PRK00168	coaD	cl00015	phosphopantetheine adenylyltransferase
	non-specific	11	141	2.25e-16	pfam01467	CTP_transf_like	cl00015	Cytidylyltransferase-like
	non-specific	9	69	3.49e-10	TIGR00125	cyt_tran_rel	cl00015	cytidyltransferase-like domain
	non-specific	9	139	2.01e-08	cd02039	cytidylyltransferase_like	cl00015	Cytidylyltransferase-like domain
	non-specific	10	72	1.02e-06	cd02165	NMNAT	cl00015	Nicotinamide/nicotinate mononucleotide adenylyltransferase
	non-specific	8	41	1.43e-05	PRK01153	PRK01153	cl00015	nicotinamide-nucleotide adenylyltransferase
	non-specific	9	64	2.26e-05	cd02156	nt_trans	cl00015	nucleotidyl transferase superfamily
	non-specific	9	41	6.88e-05	cd02166	NMNAT_Archaea	cl00015	Nicotinamide/nicotinate mononucleotide adenylyltransferase
	non-specific	8	72	8.10e-05	COG1057	NadD	cl00015	Nicotinic acid mononucleotide adenylyltransferase
	non-specific	8	41	9.27e-05	COG1056	NadR	cl00015	Nicotinamide mononucleotide adenylyltransferase
	non-specific	10	57	0.000585	PRK05379	PRK05379	cl28366	bifunctional nicotinamide mononucleotide adenylyltransferase
	superfamily	10	57	0.000585	cl28366	PRK05379 superfamily	-	bifunctional nicotinamide mononucleotide adenylyltransferase
	non-specific	7	48	0.000824	PRK00071	nadD	cl00015	nicotinic acid mononucleotide adenylyltransferase
	non-specific	7	77	0.001049	cd02170	cytidylyltransferase	cl00015	cytidylyltransferase
	non-specific	11	139	0.001357	cd02167	NMNAT_NadR	cl00015	Nicotinamide/nicotinate mononucleotide adenylyltransferase
	non-specific	8	72	0.001399	PRK07152	nadD	cl28367	putative nicotinate-nucleotide adenylyltransferase
	superfamily	8	72	0.001399	cl28367	nadD superfamily	-	putative nicotinate-nucleotide adenylyltransferase
M. collis	non-specific	11	45	0.00196	TIGR00482	TIGR00482	cl00015	nicotinate (nicotinamide) nucleotide adenylyltransferase
	non-specific	13	57	0.002052	cd02173	ECT	cl00015	CTP:phosphoethanolamine cytidylyltransferase (ECT)
	non-specific	18	73	0.004238	smart00764	Citrate_ly_lig	cl00015	Citrate lyase ligase C-terminal domain
	non-specific	20	55	0.004797	COG0414	PanC	cl00015	Panthothenate synthetase
	non-specific	18	73	0.005843	cd02169	Citrate_lyase_ligase	cl00015	Citrate lyase ligase
	non-specific	14	49	0.007424	cd02174	CCT	cl00015	CTP:phosphocholine cytidylyltransferase
M. columbinum	non-specific	2	141	2.76e-62	PRK13964	coaD	cl00015	phosphopantetheine adenylyltransferase
	superfamily	2	141	2.76e-62	cl00015	nt_trans superfamily	-	nucleotidyl transferase superfamily
	non-specific	1	141	4.83e-44	COG0669	CoaD	cl00015	Phosphopantetheine adenylyltransferase
	non-specific	4	135	3.24e-38	cd02163	PPAT	cl00015	Phosphopantetheine adenylyltransferase
	non-specific	4	135	1.49e-37	TIGR01510	coaD_prev_kdtB	cl00015	pantetheine-phosphate adenylyltransferase
	non-specific	2	135	4.49e-36	PRK00168	coaD	cl00015	phosphopantetheine adenylyltransferase
	specific	6	137	6.97e-19	pfam01467	CTP_transf_like	cl00015	Cytidylyltransferase-like
	non-specific	4	135	8.19e-11	cd02039	cytidylyltransferase_like	cl00015	Cytidylyltransferase-like domain
	non-specific	4	44	1.63e-10	TIGR00125	cyt_tran_rel	cl00015	cytidyltransferase-like domain
	non-specific	1	59	2.73e-10	COG1057	NadD	cl00015	Nicotinic acid mononucleotide adenylyltransferase
	non-specific	1	68	4.32e-10	PRK00071	nadD	cl00015	nicotinic acid mononucleotide adenylyltransferase
	non-specific	4	42	7.68e-08	cd02165	NMNAT	cl00015	Nicotinamide/nicotinate mononucleotide adenylyltransferase
	non-specific	1	36	1.63e-06	COG1056	NadR	cl00015	Nicotinamide mononucleotide adenylyltransferase
	non-specific	2	49	3.02e-06	PRK07152	nadD	cl28367	putative nicotinate-nucleotide adenylyltransferase

Query	Hit type	ID region start	ID region end	E-Value	Accession	Short name	Superfamily	Definition
	superfamily	2	49	3.02e-06	cl28367	nadD superfamily	-	putative nicotinate-nucleotide adenylyltransferase
	non-specific	4	36	6.72e-06	PRK05379	PRK05379	cl28366	bifunctional nicotinamide mononucleotide adenylyltransferase
	superfamily	4	36	6.72e-06	cl28366	PRK05379 superfamily	-	bifunctional nicotinamide mononucleotide adenylyltransferase
	non-specific	6	61	1.14e-05	TIGR00482	TIGR00482	cl00015	nicotinate (nicotinamide) nucleotide adenylyltransferase
	non-specific	5	94	1.44e-05	cd02166	NMNAT_Archaea	cl00015	Nicotinamide/nicotinate mononucleotide adenylyltransferase
	non-specific	4	37	2.74e-05	cd02167	NMNAT_NadR	cl00015	Nicotinamide/nicotinate mononucleotide adenylyltransferase
	non-specific	5	65	4.03e-05	cd02156	nt_trans	cl00015	nucleotidyl transferase superfamily
	non-specific	15	40	6.53e-05	pfam02569	Pantoate_ligase	cl00015	Pantoate-beta-alanine ligase
	non-specific	5	64	8.10e-05	TIGR01527	arch_NMN_Atrans	cl00015	nicotinamide-nucleotide adenylyltransferase
	non-specific	1	38	0.000128	PRK08887	PRK08887	cl00015	nicotinic acid mononucleotide adenylyltransferase
	non-specific	2	37	0.000329	TIGR01526	nadR_NMN_Atrans	cl28365	nicotinamide-nucleotide adenylyltransferase
	superfamily	2	37	0.000329	cl28365	PRK08099 superfamily	-	bifunctional DNA-binding transcriptional repressor/ NMN adenylyltransferase
	non-specific	5	36	0.000493	PRK01153	PRK01153	cl00015	nicotinamide-nucleotide adenylyltransferase
	non-specific	1	37	0.00119	cd02170	cytidylyltransferase	cl00015	cytidylyltransferase
	non-specific	15	40	0.001861	cd00560	PanC	cl00015	Pantoate-beta-alanine ligase
	non-specific	1	37	0.00214	cd02171	G3P_Cytidylyltransferase	cl00015	glycerol-3-phosphate cytidylyltransferase
	non-specific	14	40	0.002444	PRK00380	panC	cl00015	pantoate--beta-alanine ligase
M. columbinum	non-specific	1	44	0.00366	COG0615	TagD	cl00015	Glycerol-3-phosphate cytidylyltransferase, cytidylyltransferase family
	non-specific	1	43	0.004032	PRK08099	PRK08099	cl28365	bifunctional DNA-binding transcriptional repressor/ NMN adenylyltransferase
	non-specific	2	42	0.004274	TIGR02199	rfaE_dom_II	cl00015	rfaE bifunctional protein, domain II
	non-specific	2	40	0.005422	PLN02660	PLN02660	cl00015	pantoate--beta-alanine ligase
	non-specific	4	36	0.006818	cd02168	NMNAT_Nudix	cl00015	Nicotinamide/nicotinate mononucleotide adenylyltransferase
	non-specific	9	74	0.009055	TIGR01518	g3p_cytidyltrns	cl00015	glycerol-3-phosphate cytidylyltransferase
M. columborale	non-specific	5	141	1.17e-57	PRK13964	coaD	cl00015	phosphopantetheine adenylyltransferase
	superfamily	5	141	1.17e-57	cl00015	nt_trans superfamily	-	nucleotidyl transferase superfamily
	non-specific	5	136	1.15e-41	COG0669	CoaD	cl00015	Phosphopantetheine adenylyltransferase
	non-specific	5	135	6.32e-39	cd02163	PPAT	cl00015	Phosphopantetheine adenylyltransferase
	non-specific	5	135	2.39e-36	PRK00168	coaD	cl00015	phosphopantetheine adenylyltransferase
	non-specific	5	135	4.94e-35	TIGR01510	coaD_prev_kdtB	cl00015	pantetheine-phosphate adenylyltransferase
	specific	7	137	6.56e-23	pfam01467	CTP_transf_like	cl00015	Cytidylyltransferase-like
	non-specific	5	59	3.64e-10	TIGR00125	cyt_tran_rel	cl00015	cytidylyltransferase-like domain
	non-specific	5	135	1.53e-08	cd02039	cytidylyltransferase_like	cl00015	Cytidylyltransferase-like domain
	non-specific	1	53	2.08e-07	PRK00071	nadD	cl00015	nicotinic acid mononucleotide adenylyltransferase
	non-specific	5	68	2.50e-07	cd02165	NMNAT	cl00015	Nicotinamide/nicotinate mononucleotide adenylyltransferase
	non-specific	5	68	1.42e-06	PRK07152	nadD	cl28367	putative nicotinate-nucleotide adenylyltransferase
	superfamily	5	68	1.42e-06	cl28367	nadD superfamily	-	putative nicotinate-nucleotide adenylyltransferase
	non-specific	1	54	2.17e-05	COG1057	NadD	cl00015	Nicotinic acid mononucleotide adenylyltransferase
	non-specific	6	46	0.000119	cd02156	nt_trans	cl00015	nucleotidyl transferase superfamily
	non-specific	1	38	0.000121	COG1019	CAB4	cl00015	Phosphopantetheine adenylyltransferase
	non-specific	10	38	0.000123	PRK00777	PRK00777	cl00015	phosphopantetheine adenylyltransferase

Query	Hit type	ID region start	ID region end	E-Value	Accession	Short name	Superfamily	Definition
	non-specific	1	37	0.000197	COG1056	NadR	cl00015	Nicotinamide mononucleotide adenylyltransferase
	non-specific	5	135	0.000343	cd02167	NMNAT_NadR	cl00015	Nicotinamide/nicotinate mononucleotide adenylyltransferase
	non-specific	4	135	0.001285	TIGR01526	nadR_NMN_Atrans	cl28365	nicotinamide-nucleotide adenylyltransferase
	superfamily	4	135	0.001285	cl28365	PRK08099 superfamily	-	bifunctional DNA-binding transcriptional repressor/ NMN adenylyltransferase
	non-specific	1	136	0.001697	PRK08099	PRK08099	cl28365	bifunctional DNA-binding transcriptional repressor/ NMN adenylyltransferase
	non-specific	7	62	0.003172	TIGR00482	TIGR00482	cl00015	nicotinate (nicotinamide) nucleotide adenylyltransferase
	non-specific	1	41	0.003186	PRK05379	PRK05379	cl28366	bifunctional nicotinamide mononucleotide adenylyltransferase
	superfamily	1	41	0.003186	cl28366	PRK05379 superfamily	-	bifunctional nicotinamide mononucleotide adenylyltransferase
	non-specific	10	135	0.003564	cd02164	PPAT_CoAS	cl00015	phosphopantetheine adenylyltransferase
	non-specific	5	64	0.006841	PRK01170	PRK01170	cl00866	phosphopantetheine adenylyltransferase
	superfamily	5	64	0.006841	cl00866	NTPase_I-T superfamily	-	Protein of unknown function DUF84
M. columborale	non-specific	6	37	0.008472	cd02166	NMNAT_Archaea	cl00015	Nicotinamide/nicotinate mononucleotide adenylyltransferase
	specific	10	148	6.49e-74	PRK13964	coaD	cl00015	phosphopantetheine adenylyltransferase
	superfamily	10	148	6.49e-74	cl00015	nt_trans superfamily	-	nucleotidyl transferase superfamily
	non-specific	10	148	6.38e-45	COG0669	CoaD	cl00015	Phosphopantetheine adenylyltransferase
	non-specific	13	140	2.18e-41	cd02163	PPAT	cl00015	Phosphopantetheine adenylyltransferase
	non-specific	13	140	7.70e-39	PRK00168	coaD	cl00015	phosphopantetheine adenylyltransferase
	non-specific	13	142	1.21e-37	TIGR01510	coaD_prev_kdtB	cl00015	pantetheine-phosphate adenylyltransferase
	non-specific	14	144	8.39e-15	pfam01467	CTP_transf_like	cl00015	Cytidylyltransferase-like
	non-specific	13	45	4.87e-10	TIGR00125	cyt_tran_rel	cl00015	cytidyltransferase-like domain
	non-specific	11	75	1.08e-09	PRK00071	nadD	cl00015	nicotinic acid mononucleotide adenylyltransferase
	non-specific	13	75	1.12e-09	cd02165	NMNAT	cl00015	Nicotinamide/nicotinate mononucleotide adenylyltransferase
	non-specific	13	142	1.57e-09	cd02039	cytidylyltransferase_like	cl00015	Cytidylyltransferase-like domain
	non-specific	11	75	7.49e-08	COG1057	NadD	cl00015	Nicotinic acid mononucleotide adenylyltransferase
	non-specific	11	81	1.44e-06	PRK07152	nadD	cl28367	putative nicotinate-nucleotide adenylyltransferase
	superfamily	11	81	1.44e-06	cl28367	nadD superfamily	-	putative nicotinate-nucleotide adenylyltransferase
M. conjunctivae	non-specific	14	64	7.14e-06	TIGR00482	TIGR00482	cl00015	nicotinate (nicotinamide) nucleotide adenylyltransferase
	non-specific	14	75	1.52e-05	cd02167	NMNAT_NadR	cl00015	Nicotinamide/nicotinate mononucleotide adenylyltransferase
	non-specific	13	99	1.59e-05	cd02156	nt_trans	cl00015	nucleotidyl transferase superfamily
	non-specific	9	44	3.75e-05	COG1056	NadR	cl00015	Nicotinamide mononucleotide adenylyltransferase
	non-specific	10	45	8.92e-05	cd02170	cytidylyltransferase	cl00015	cytidylyltransferase
	non-specific	22	48	9.34e-05	pfam02569	Pantoate_ligase	cl00015	Pantoate-beta-alanine ligase
	non-specific	13	44	0.000168	PRK05379	PRK05379	cl28366	bifunctional nicotinamide mononucleotide adenylyltransferase
	superfamily	13	44	0.000168	cl28366	PRK05379 superfamily	-	bifunctional nicotinamide mononucleotide adenylyltransferase
	non-specific	11	44	0.001029	PRK01153	PRK01153	cl00015	nicotinamide-nucleotide adenylyltransferase
	non-specific	17	45	0.001618	PRK00777	PRK00777	cl00015	phosphopantetheine adenylyltransferase
	non-specific	13	44	0.001775	cd02166	NMNAT_Archaea	cl00015	Nicotinamide/nicotinate mononucleotide adenylyltransferase
	non-specific	22	48	0.002492	COG0414	PanC	cl00015	Panthothenate synthetase
	non-specific	14	80	0.00287	TIGR01526	nadR_NMN_Atrans	cl28365	nicotinamide-nucleotide adenylyltransferase
	superfamily	14	80	0.00287	cl28365	PRK08099 superfamily	-	bifunctional DNA-binding transcriptional repressor/ NMN adenylyltransferase

Query	Hit type	ID region start	ID region end	E-Value	Accession	Short name	Superfamily	Definition
	non-specific	10	45	0.002877	cd02171	G3P_Cytidylyltransferase	cl00015	glycerol-3-phosphate cytidylyltransferase
	non-specific	22	48	0.003383	PRK00380	panC	cl00015	pantoate--beta-alanine ligase
	non-specific	25	80	0.00395	smart00764	Citrate_ly_lig	cl00015	Citrate lyase ligase C-terminal domain
	non-specific	22	48	0.004388	cd00560	PanC	cl00015	Pantoate-beta-alanine ligase
	non-specific	17	51	0.004435	TIGR02199	rfaE_dom_II	cl00015	rfaE bifunctional protein, domain II
	non-specific	25	80	0.005019	cd02169	Citrate_lyase_ligase	cl00015	Citrate lyase ligase
M. conjunctivae	non-specific	13	44	0.006585	cd02168	NMNAT_Nudix	cl00015	Nicotinamide/nicotinate mononucleotide adenylyltransferase
	non-specific	17	96	0.007066	PRK05627	PRK05627	cl27514	bifunctional riboflavin kinase/FMN adenylyltransferase
	superfamily	17	96	0.007066	cl27514	Flavokinase superfamily	-	Riboflavin kinase
	non-specific	2	50	0.009592	PRK08099	PRK08099	cl28365	bifunctional DNA-binding transcriptional repressor/ NMN adenylyltransferase
M. cricetuli	non-specific	3	142	2.92e-62	PRK13964	coaD	cl00015	phosphopantetheine adenylyltransferase
	superfamily	3	142	2.92e-62	cl00015	nt_trans superfamily	-	nucleotidyl transferase superfamily
	non-specific	4	136	8.86e-48	COG0669	CoaD	cl00015	Phosphopantetheine adenylyltransferase
	non-specific	5	135	1.57e-44	cd02163	PPAT	cl00015	Phosphopantetheine adenylyltransferase
	non-specific	5	135	9.74e-44	TIGR01510	coaD_prev_kdtB	cl00015	pantetheine-phosphate adenylyltransferase
	non-specific	4	135	1.86e-43	PRK00168	coaD	cl00015	phosphopantetheine adenylyltransferase
	specific	7	137	1.52e-21	pfam01467	CTP_transf_like	cl00015	Cytidylyltransferase-like
	non-specific	5	65	1.46e-11	TIGR00125	cyt_tran_rel	cl00015	cytidyltransferase-like domain
	non-specific	1	68	1.52e-11	PRK00071	nadD	cl00015	nicotinic acid mononucleotide adenylyltransferase
	non-specific	5	68	1.17e-10	cd02165	NMNAT	cl00015	Nicotinamide/nicotinate mononucleotide adenylyltransferase
	non-specific	1	57	7.02e-09	COG1057	NadD	cl00015	Nicotinic acid mononucleotide adenylyltransferase
	non-specific	4	68	2.03e-08	PRK07152	nadD	cl28367	putative nicotinate-nucleotide adenylyltransferase
	superfamily	4	68	2.03e-08	cl28367	nadD superfamily	-	putative nicotinate-nucleotide adenylyltransferase
	non-specific	5	135	4.94e-08	cd02039	cytidylyltransferase_like	cl00015	Cytidylyltransferase-like domain
	non-specific	1	37	3.32e-07	COG1056	NadR	cl00015	Nicotinamide mononucleotide adenylyltransferase
	non-specific	6	48	1.27e-06	cd02156	nt_trans	cl00015	nucleotidyl transferase superfamily
	non-specific	5	135	2.47e-06	cd02167	NMNAT_NadR	cl00015	Nicotinamide/nicotinate mononucleotide adenylyltransferase
	non-specific	1	136	1.48e-05	PRK08099	PRK08099	cl28365	bifunctional DNA-binding transcriptional repressor/ NMN adenylyltransferase
	superfamily	1	136	1.48e-05	cl28365	PRK08099 superfamily	-	bifunctional DNA-binding transcriptional repressor/ NMN adenylyltransferase
	non-specific	7	57	1.51e-05	TIGR00482	TIGR00482	cl00015	nicotinate (nicotinamide) nucleotide adenylyltransferase
	non-specific	4	44	2.42e-05	PTZ00308	PTZ00308	cl28626	ethanolamine-phosphate cytidylyltransferase
	superfamily	4	44	2.42e-05	cl28626	PLN02406 superfamily	-	ethanolamine-phosphate cytidylyltransferase
	non-specific	10	38	0.000143	COG0615	TagD	cl00015	Glycerol-3-phosphate cytidylyltransferase, cytidylyltransferase family
	non-specific	10	38	0.000178	cd02170	cytidylyltransferase	cl00015	cytidylyltransferase
	non-specific	6	37	0.000211	PRK01153	PRK01153	cl00015	nicotinamide-nucleotide adenylyltransferase
	non-specific	10	35	0.00061	PRK00777	PRK00777	cl00015	phosphopantetheine adenylyltransferase
	non-specific	7	43	0.000627	cd02173	ECT	cl00015	CTP:phosphoethanolamine cytidylyltransferase (ECT)
	non-specific	6	37	0.000779	cd02166	NMNAT_Archaea	cl00015	Nicotinamide/nicotinate mononucleotide adenylyltransferase
	non-specific	4	74	0.001273	TIGR01526	nadR_NMN_Atrans	cl28365	nicotinamide-nucleotide adenylyltransferase

Query	Hit type	ID region start	ID region end	E-Value	Accession	Short name	Superfamily	Definition
	non-specific	4	38	0.005008	cd02171	G3P_Cytidylyltransferase	cl00015	glycerol-3-phosphate cytidylyltransferase
	non-specific	4	43	0.005313	TIGR02199	rfaE_dom_II	cl00015	rfaE bifunctional protein, domain II
M. cricetuli	non-specific	1	37	0.006315	PRK05379	PRK05379	cl28366	bifunctional nicotinamide mononucleotide adenylyltransferase
	superfamily	1	37	0.006315	cl28366	PRK05379 superfamily	-	bifunctional nicotinamide mononucleotide adenylyltransferase
	non-specific	10	37	0.007769	COG1019	CAB4	cl00015	Phosphopantetheine adenylyltransferase
M. crocodyli	specific	5	146	5.89e-66	PRK13964	coaD	cl00015	phosphopantetheine adenylyltransferase
	superfamily	5	146	5.89e-66	cl00015	nt_trans superfamily	-	nucleotidyl transferase superfamily
	non-specific	6	140	1.71e-39	COG0669	CoaD	cl00015	Phosphopantetheine adenylyltransferase
	non-specific	7	137	2.40e-34	cd02163	PPAT	cl00015	Phosphopantetheine adenylyltransferase
	non-specific	7	146	2.85e-34	TIGR01510	coaD_prev_kdtB	cl00015	pantetheine-phosphate adenylyltransferase
	non-specific	6	137	8.79e-31	PRK00168	coaD	cl00015	phosphopantetheine adenylyltransferase
	non-specific	9	141	8.01e-14	pfam01467	CTP_transf_like	cl00015	Cytidylyltransferase-like
	non-specific	7	58	5.67e-06	TIGR00125	cyt_tran_rel	cl00015	cytidyltransferase-like domain
	non-specific	7	139	0.000547	cd02039	cytidylyltransferase_like	cl00015	Cytidylyltransferase-like domain
	non-specific	8	71	0.008926	cd02165	NMNAT	cl00015	Nicotinamide/nicotinate mononucleotide adenylyltransferase
M. felifaucium	non-specific	3	142	8.93e-66	PRK13964	coaD	cl00015	phosphopantetheine adenylyltransferase
	superfamily	3	142	8.93e-66	cl00015	nt_trans superfamily	-	nucleotidyl transferase superfamily
	non-specific	3	136	1.15e-51	COG0669	CoaD	cl00015	Phosphopantetheine adenylyltransferase
	non-specific	5	142	3.50e-47	TIGR01510	coaD_prev_kdtB	cl00015	pantetheine-phosphate adenylyltransferase
	non-specific	5	135	1.94e-46	cd02163	PPAT	cl00015	Phosphopantetheine adenylyltransferase
	non-specific	3	135	3.41e-46	PRK00168	coaD	cl00015	phosphopantetheine adenylyltransferase
	specific	7	135	1.99e-24	pfam01467	CTP_transf_like	cl00015	Cytidylyltransferase-like
	non-specific	5	65	5.00e-14	TIGR00125	cyt_tran_rel	cl00015	cytidyltransferase-like domain
	non-specific	5	135	6.32e-14	cd02039	cytidylyltransferase_like	cl00015	Cytidylyltransferase-like domain
	non-specific	1	37	1.77e-10	COG1056	NadR	cl00015	Nicotinamide mononucleotide adenylyltransferase
	non-specific	1	57	6.34e-09	PRK00071	nadD	cl00015	nicotinic acid mononucleotide adenylyltransferase
	non-specific	6	135	1.07e-08	cd02166	NMNAT_Archaea	cl00015	Nicotinamide/nicotinate mononucleotide adenylyltransferase
	non-specific	1	57	3.39e-08	COG1057	NadD	cl00015	Nicotinic acid mononucleotide adenylyltransferase
	non-specific	6	70	1.04e-07	TIGR01527	arch_NMN_Atrans	cl00015	nicotinamide-nucleotide adenylyltransferase
	non-specific	6	37	1.65e-07	PRK01153	PRK01153	cl00015	nicotinamide-nucleotide adenylyltransferase
	non-specific	5	135	3.51e-07	cd02167	NMNAT_NadR	cl00015	Nicotinamide/nicotinate mononucleotide adenylyltransferase
	non-specific	5	44	8.59e-07	cd02165	NMNAT	cl00015	Nicotinamide/nicotinate mononucleotide adenylyltransferase
	non-specific	3	69	1.08e-06	PRK07152	nadD	cl28367	putative nicotinate-nucleotide adenylyltransferase
	superfamily	3	69	1.08e-06	cl28367	nadD superfamily	-	putative nicotinate-nucleotide adenylyltransferase
	non-specific	7	54	1.57e-06	TIGR00482	TIGR00482	cl00015	nicotinate (nicotinamide) nucleotide adenylyltransferase
	non-specific	3	71	3.90e-06	COG1019	CAB4	cl00015	Phosphopantetheine adenylyltransferase
	non-specific	1	136	3.35e-05	PRK08099	PRK08099	cl28365	bifunctional DNA-binding transcriptional repressor/ NMN adenylyltransferase
M. felifaucium	superfamily	1	136	3.35e-05	cl28365	PRK08099 superfamily	-	bifunctional DNA-binding transcriptional repressor/ NMN adenylyltransferase
	non-specific	5	41	3.99e-05	cd02156	nt_trans	cl00015	nucleotidyl transferase superfamily
	non-specific	3	65	6.86e-05	PRK00777	PRK00777	cl00015	phosphopantetheine adenylyltransferase
	non-specific	1	37	0.00019	PRK05379	PRK05379	cl28366	bifunctional nicotinamide mononucleotide adenylyltransferase

Query	Hit type	ID region start	ID region end	E-Value	Accession	Short name	Superfamily	Definition
	superfamily	1	37	0.00019	cl28366	PRK05379 superfamily	-	bifunctional nicotinamide mononucleotide adenylyltransferase
	non-specific	3	38	0.000316	TIGR01526	nadR_NMN_Atrans	cl28365	nicotinamide-nucleotide adenylyltransferase
	non-specific	4	44	0.000686	cd02173	ECT	cl00015	CTP:phosphoethanolamine cytidylyltransferase (ECT)
	non-specific	10	57	0.000689	cd02174	CCT	cl00015	CTP:phosphocholine cytidylyltransferase
	non-specific	3	38	0.000828	cd02170	cytidylyltransferase	cl00015	cytidylyltransferase
	non-specific	2	45	0.001162	COG0615	TagD	cl00015	Glycerol-3-phosphate cytidylyltransferase, cytidylyltransferase family
	non-specific	3	43	0.001447	cd02172	RfaE_N	cl00015	N-terminal domain of RfaE
	non-specific	5	27	0.002937	cd02168	NMNAT_Nudix	cl00015	Nicotinamide/nicotinate mononucleotide adenylyltransferase
	non-specific	4	56	0.002975	PTZ00308	PTZ00308	cl28626	ethanolamine-phosphate cytidylyltransferase
	superfamily	4	56	0.002975	cl28626	PLN02406 superfamily	-	ethanolamine-phosphate cytidylyltransferase
	non-specific	10	88	0.005345	PRK07143	PRK07143	cl27514	hypothetical protein
	superfamily	10	88	0.005345	cl27514	Flavokinase superfamily	-	Riboflavin kinase
	non-specific	16	48	0.006602	pfam02569	Pantoate_ligase	cl00015	Pantoate-beta-alanine ligase
	non-specific	5	41	0.008476	cd02064	FAD_synthetase_N	cl00015	FAD synthetase, N-terminal domain of the bifunctional enzyme
M. felis	non-specific	2	136	6.63e-51	PRK13964	coaD	cl00015	phosphopantetheine adenylyltransferase
	superfamily	2	136	6.63e-51	cl00015	nt_trans superfamily	-	nucleotidyl transferase superfamily
	non-specific	1	133	4.27e-41	COG0669	CoaD	cl00015	Phosphopantetheine adenylyltransferase
	non-specific	5	133	2.40e-39	TIGR01510	coaD_prev_kdtB	cl00015	pantetheine-phosphate adenylyltransferase
	non-specific	5	131	4.17e-38	cd02163	PPAT	cl00015	Phosphopantetheine adenylyltransferase
	non-specific	5	131	1.97e-34	PRK00168	coaD	cl00015	phosphopantetheine adenylyltransferase
	specific	6	135	1.82e-19	pfam01467	CTP_transf_like	cl00015	Cytidylyltransferase-like
	non-specific	5	48	5.61e-10	TIGR00125	cyt_tran_rel	cl00015	cytidyltransferase-like domain
	non-specific	1	76	3.69e-07	COG1057	NadD	cl00015	Nicotinic acid mononucleotide adenylyltransferase
	non-specific	5	134	3.99e-07	cd02039	cytidylyltransferase_like	cl00015	Cytidylyltransferase-like domain
	non-specific	5	76	5.29e-07	cd02165	NMNAT	cl00015	Nicotinamide/nicotinate mononucleotide adenylyltransferase
	non-specific	5	62	1.01e-06	cd02166	NMNAT_Archaea	cl00015	Nicotinamide/nicotinate mononucleotide adenylyltransferase
	non-specific	1	76	3.92e-06	PRK00071	nadD	cl00015	nicotinic acid mononucleotide adenylyltransferase
	non-specific	1	46	1.13e-05	COG1056	NadR	cl00015	Nicotinamide mononucleotide adenylyltransferase
	non-specific	5	36	1.17e-05	PRK05379	PRK05379	cl28366	bifunctional nicotinamide mononucleotide adenylyltransferase
	superfamily	5	36	1.17e-05	cl28366	PRK05379 superfamily	-	bifunctional nicotinamide mononucleotide adenylyltransferase
M. felis	non-specific	6	76	2.15e-05	TIGR00482	TIGR00482	cl00015	nicotinate (nicotinamide) nucleotide adenylyltransferase
	non-specific	5	46	2.27e-05	PRK01153	PRK01153	cl00015	nicotinamide-nucleotide adenylyltransferase
	non-specific	9	48	1.00e-04	cd02174	CCT	cl00015	CTP:phosphocholine cytidylyltransferase
	non-specific	5	68	0.000101	TIGR01527	arch_NMN_Atrans	cl00015	nicotinamide-nucleotide adenylyltransferase
	non-specific	5	66	0.000241	PRK07152	nadD	cl28367	putative nicotinate-nucleotide adenylyltransferase
	superfamily	5	66	0.000241	cl28367	nadD superfamily	-	putative nicotinate-nucleotide adenylyltransferase
	non-specific	5	36	0.000349	cd02168	NMNAT_Nudix	cl00015	Nicotinamide/nicotinate mononucleotide adenylyltransferase
	non-specific	1	37	0.001005	PRK08887	PRK08887	cl00015	nicotinic acid mononucleotide adenylyltransferase
	non-specific	5	40	0.002275	cd02156	nt_trans	cl00015	nucleotidyl transferase superfamily
	non-specific	9	86	0.006609	COG0615	TagD	cl00015	Glycerol-3-phosphate cytidylyltransferase, cytidylyltransferase family
M. fermentans	non-specific	3	141	7.03e-63	PRK13964	coaD	cl00015	phosphopantetheine adenylyltransferase

Query	Hit type	ID region start	ID region end	E-Value	Accession	Short name	Superfamily	Definition
	superfamily	3	141	7.03e-63	cl00015	nt_trans superfamily	-	nucleotidyl transferase superfamily
	non-specific	1	140	8.44e-50	COG0669	CoaD	cl00015	Phosphopantetheine adenylyltransferase
	non-specific	4	134	3.86e-47	cd02163	PPAT	cl00015	Phosphopantetheine adenylyltransferase
	non-specific	3	134	3.77e-46	PRK00168	coaD	cl00015	phosphopantetheine adenylyltransferase
	non-specific	4	134	2.60e-42	TIGR01510	coaD_prev_kdtB	cl00015	pantetheine-phosphate adenylyltransferase
	specific	6	136	5.06e-23	pfam01467	CTP_transf_like	cl00015	Cytidylyltransferase-like
	non-specific	4	56	2.61e-14	TIGR00125	cyt_tran_rel	cl00015	cytidyltransferase-like domain
	non-specific	1	68	4.23e-12	COG1057	NadD	cl00015	Nicotinic acid mononucleotide adenylyltransferase
	non-specific	4	134	6.66e-12	cd02039	cytidylyltransferase_like	cl00015	Cytidylyltransferase-like domain
	non-specific	1	67	2.65e-11	PRK00071	nadD	cl00015	nicotinic acid mononucleotide adenylyltransferase
	non-specific	5	67	8.01e-11	cd02165	NMNAT	cl00015	Nicotinamide/nicotinate mononucleotide adenylyltransferase
	non-specific	3	36	5.60e-09	COG1056	NadR	cl00015	Nicotinamide mononucleotide adenylyltransferase
	non-specific	9	134	1.07e-08	cd02167	NMNAT_NadR	cl00015	Nicotinamide/nicotinate mononucleotide adenylyltransferase
	non-specific	6	61	2.32e-08	TIGR00482	TIGR00482	cl00015	nicotinate (nicotinamide) nucleotide adenylyltransferase
	non-specific	3	36	4.28e-08	PRK01153	PRK01153	cl00015	nicotinamide-nucleotide adenylyltransferase
	non-specific	4	63	6.34e-08	cd02166	NMNAT_Archaea	cl00015	Nicotinamide/nicotinate mononucleotide adenylyltransferase
	non-specific	4	56	9.89e-08	cd02156	nt_trans	cl00015	nucleotidyl transferase superfamily
	non-specific	3	61	2.69e-07	PRK07152	nadD	cl28367	putative nicotinate-nucleotide adenylyltransferase
	superfamily	3	61	2.69e-07	cl28367	nadD superfamily	-	putative nicotinate-nucleotide adenylyltransferase
	non-specific	4	67	2.77e-07	TIGR01527	arch_NMN_Atrans	cl00015	nicotinamide-nucleotide adenylyltransferase
	non-specific	5	61	8.30e-06	PRK05379	PRK05379	cl28366	bifunctional nicotinamide mononucleotide adenylyltransferase
	superfamily	5	61	8.30e-06	cl28366	PRK05379 superfamily	-	bifunctional nicotinamide mononucleotide adenylyltransferase
	non-specific	9	73	2.49e-05	TIGR01526	nadR_NMN_Atrans	cl28365	nicotinamide-nucleotide adenylyltransferase
	superfamily	9	73	2.49e-05	cl28365	PRK08099 superfamily	-	bifunctional DNA-binding transcriptional repressor/ NMN adenylyltransferase
M. fermentans	non-specific	9	56	3.99e-05	COG0615	TagD	cl00015	Glycerol-3-phosphate cytidylyltransferase, cytidylyltransferase family
	non-specific	9	37	4.05e-05	cd02170	cytidylyltransferase	cl00015	cytidylyltransferase
	non-specific	5	78	9.88e-05	cd02168	NMNAT_Nudix	cl00015	Nicotinamide/nicotinate mononucleotide adenylyltransferase
	non-specific	3	42	0.000254	TIGR02199	rfaE_dom_II	cl00015	rfaE bifunctional protein, domain II
	non-specific	9	56	0.000745	cd02174	CCT	cl00015	CTP:phosphocholine cytidylyltransferase
	non-specific	15	40	0.000758	pfam02569	Pantoate_ligase	cl00015	Pantoate-beta-alanine ligase
	non-specific	9	57	0.000849	TIGR01518	g3p_cytidyltrns	cl00015	glycerol-3-phosphate cytidylyltransferase
	non-specific	8	55	0.000992	PTZ00308	PTZ00308	cl28626	ethanolamine-phosphate cytidylyltransferase
	superfamily	8	55	0.000992	cl28626	PLN02406 superfamily	-	ethanolamine-phosphate cytidylyltransferase
	non-specific	3	64	0.001208	cd02171	G3P_Cytidylyltransferase	cl00015	glycerol-3-phosphate cytidylyltransferase
	non-specific	9	42	0.002815	COG2870	RfaE	cl28454	ADP-heptose synthase, bifunctional sugar kinase/adenylyltransferase
	superfamily	9	42	0.002815	cl28454	RfaE superfamily	-	ADP-heptose synthase, bifunctional sugar kinase/adenylyltransferase
	non-specific	9	135	0.00309	PRK08099	PRK08099	cl28365	bifunctional DNA-binding transcriptional repressor/ NMN adenylyltransferase
	non-specific	3	69	0.00316	PRK00777	PRK00777	cl00015	phosphopantetheine adenylyltransferase

Query	Hit type	ID region start	ID region end	E-Value	Accession	Short name	Superfamily	Definition
	non-specific	9	36	0.005021	PRK11316	PRK11316	cl28454	bifunctional heptose 7-phosphate kinase/heptose 1-phosphate adenyltransferase
	non-specific	8	34	0.007501	cd02173	ECT	cl00015	CTP:phosphoethanolamine cytidylyltransferase (ECT)
M. gallinaceum	non-specific	5	143	9.54e-64	PRK13964	coaD	cl00015	phosphopantetheine adenylyltransferase
	superfamily	5	143	9.54e-64	cl00015	nt_trans superfamily	-	nucleotidyl transferase superfamily
	non-specific	3	145	1.96e-45	COG0669	CoaD	cl00015	Phosphopantetheine adenylyltransferase
	non-specific	6	145	1.02e-41	TIGR01510	coaD_prev_kdtB	cl00015	pantetheine-phosphate adenylyltransferase
	non-specific	5	145	9.37e-39	PRK00168	coaD	cl00015	phosphopantetheine adenylyltransferase
	non-specific	6	136	1.86e-38	cd02163	PPAT	cl00015	Phosphopantetheine adenylyltransferase
	specific	8	138	1.46e-19	pfam01467	CTP_transf_like	cl00015	Cytidylyltransferase-like
	non-specific	6	136	2.50e-11	cd02039	cytidylyltransferase_like	cl00015	Cytidylyltransferase-like domain
	non-specific	6	38	2.53e-09	TIGR00125	cyt_tran_rel	cl00015	cytidyltransferase-like domain
	non-specific	7	69	3.46e-08	cd02165	NMNAT	cl00015	Nicotinamide/nicotinate mononucleotide adenylyltransferase
	non-specific	5	69	9.23e-08	PRK00071	nadD	cl00015	nicotinic acid mononucleotide adenylyltransferase
	non-specific	6	95	4.66e-06	cd02156	nt_trans	cl00015	nucleotidyl transferase superfamily
	non-specific	8	69	7.41e-06	TIGR00482	TIGR00482	cl00015	nicotinate (nicotinamide) nucleotide adenylyltransferase
	non-specific	5	64	1.59e-05	COG1057	NadD	cl00015	Nicotinic acid mononucleotide adenylyltransferase
	non-specific	8	48	7.01e-05	cd02167	NMNAT_NadR	cl00015	Nicotinamide/nicotinate mononucleotide adenylyltransferase
	non-specific	8	44	0.00012	cd02174	CCT	cl00015	CTP:phosphocholine cytidylyltransferase
	non-specific	5	39	0.000131	cd02170	cytidylyltransferase	cl00015	cytidylyltransferase
	non-specific	5	38	0.000193	COG1056	NadR	cl00015	Nicotinamide mononucleotide adenylyltransferase
	non-specific	6	44	0.0002	cd02173	ECT	cl00015	CTP:phosphoethanolamine cytidylyltransferase (ECT)
M. gallinaceum	non-specific	3	44	0.000461	PTZ00308	PTZ00308	cl28626	ethanolamine-phosphate cytidylyltransferase
	superfamily	3	44	0.000461	cl28626	PLN02406 superfamily	-	ethanolamine-phosphate cytidylyltransferase
	non-specific	5	69	0.000576	PRK07152	nadD	cl28367	putative nicotinate-nucleotide adenylyltransferase
	superfamily	5	69	0.000576	cl28367	nadD superfamily	-	putative nicotinate-nucleotide adenylyltransferase
	non-specific	5	139	0.000995	PRK00777	PRK00777	cl00015	phosphopantetheine adenylyltransferase
	non-specific	5	39	0.001682	cd02171	G3P_Cytidylyltransferase	cl00015	glycerol-3-phosphate cytidylyltransferase
	non-specific	5	39	0.002016	COG0615	TagD	cl00015	Glycerol-3-phosphate cytidylyltransferase, cytidylyltransferase family
	non-specific	8	48	0.002371	TIGR01526	nadR_NMN_Atrans	cl28365	nicotinamide-nucleotide adenylyltransferase
	superfamily	8	48	0.002371	cl28365	PRK08099 superfamily	-	bifunctional DNA-binding transcriptional repressor/ NMN adenylyltransferase
	non-specific	1	66	0.006453	COG1019	CAB4	cl00015	Phosphopantetheine adenylyltransferase
M. gallinarum	non-specific	2	141	9.93e-64	PRK13964	coaD	cl00015	phosphopantetheine adenylyltransferase
	superfamily	2	141	9.93e-64	cl00015	nt_trans superfamily	-	nucleotidyl transferase superfamily
	non-specific	1	140	1.36e-47	COG0669	CoaD	cl00015	Phosphopantetheine adenylyltransferase
	non-specific	4	141	3.25e-44	TIGR01510	coaD_prev_kdtB	cl00015	pantetheine-phosphate adenylyltransferase
	non-specific	3	132	8.06e-42	PRK00168	coaD	cl00015	phosphopantetheine adenylyltransferase
	non-specific	4	132	9.66e-42	cd02163	PPAT	cl00015	Phosphopantetheine adenylyltransferase
	specific	6	132	1.87e-20	pfam01467	CTP_transf_like	cl00015	Cytidylyltransferase-like
	non-specific	4	64	8.98e-14	TIGR00125	cyt_tran_rel	cl00015	cytidyltransferase-like domain
	non-specific	3	67	3.27e-11	PRK07152	nadD	cl28367	putative nicotinate-nucleotide adenylyltransferase

Query	Hit type	ID region start	ID region end	E-Value	Accession	Short name	Superfamily	Definition
	superfamily	3	67	3.27e-11	cl28367	nadD superfamily	-	putative nicotinate-nucleotide adenylyltransferase
	non-specific	1	56	2.69e-10	COG1057	NadD	cl00015	Nicotinic acid mononucleotide adenylyltransferase
	non-specific	1	36	2.25e-09	COG1056	NadR	cl00015	Nicotinamide mononucleotide adenylyltransferase
	non-specific	4	134	3.06e-09	cd02039	cytidylyltransferase_like	cl00015	Cytidylyltransferase-like domain
	non-specific	4	67	3.78e-09	cd02165	NMNAT	cl00015	Nicotinamide/nicotinate mononucleotide adenylyltransferase
	non-specific	1	53	1.54e-08	PRK00071	nadD	cl00015	nicotinic acid mononucleotide adenylyltransferase
	non-specific	5	56	6.30e-08	PRK05379	PRK05379	cl28366	bifunctional nicotinamide mononucleotide adenylyltransferase
	superfamily	5	56	6.30e-08	cl28366	PRK05379 superfamily	-	bifunctional nicotinamide mononucleotide adenylyltransferase
	non-specific	6	56	6.69e-08	TIGR00482	TIGR00482	cl00015	nicotinate (nicotinamide) nucleotide adenylyltransferase
	non-specific	5	36	8.94e-07	PRK01153	PRK01153	cl00015	nicotinamide-nucleotide adenylyltransferase
	non-specific	4	91	9.39e-07	cd02156	nt_trans	cl00015	nucleotidyl transferase superfamily
	non-specific	5	36	1.10e-06	cd02166	NMNAT_Archaea	cl00015	Nicotinamide/nicotinate mononucleotide adenylyltransferase
	non-specific	1	57	3.21e-06	cd02170	cytidylyltransferase	cl00015	cytidylyltransferase
	non-specific	2	84	4.94e-06	TIGR02199	rfaE_dom_II	cl00015	rfaE bifunctional protein, domain II
	non-specific	4	60	5.26e-06	cd02168	NMNAT_Nudix	cl00015	Nicotinamide/nicotinate mononucleotide adenylyltransferase
	non-specific	1	72	1.88e-05	COG0615	TagD	cl00015	Glycerol-3-phosphate cytidylyltransferase, cytidylyltransferase family
M. gallinarum	non-specific	4	43	2.66e-05	cd02167	NMNAT_NadR	cl00015	Nicotinamide/nicotinate mononucleotide adenylyltransferase
	non-specific	5	67	3.72e-05	TIGR01527	arch_NMN_Atrans	cl00015	nicotinamide-nucleotide adenylyltransferase
	non-specific	1	43	5.45e-05	PRK08099	PRK08099	cl28365	bifunctional DNA-binding transcriptional repressor/ NMN adenylyltransferase
	superfamily	1	43	5.45e-05	cl28365	PRK08099 superfamily	-	bifunctional DNA-binding transcriptional repressor/ NMN adenylyltransferase
	non-specific	2	37	7.50e-05	cd02171	G3P_Cytidylyltransferase	cl00015	glycerol-3-phosphate cytidylyltransferase
	non-specific	3	37	8.20e-05	TIGR01526	nadR_NMN_Atrans	cl28365	nicotinamide-nucleotide adenylyltransferase
	non-specific	2	36	0.000151	PTZ00308	PTZ00308	cl28626	ethanolamine-phosphate cytidylyltransferase
	superfamily	2	36	0.000151	cl28626	PLN02406 superfamily	-	ethanolamine-phosphate cytidylyltransferase
	non-specific	3	64	0.00023	PRK01170	PRK01170	cl00866	phosphopantetheine adenylyltransferase
	superfamily	3	64	0.00023	cl00866	NTPase_I-T superfamily	-	Protein of unknown function DUF84
	non-specific	14	40	0.000247	pfam02569	Pantoate_ligase	cl00015	Pantoate-beta-alanine ligase
	non-specific	1	34	0.000283	PRK00777	PRK00777	cl00015	phosphopantetheine adenylyltransferase
	non-specific	2	36	0.000525	PRK11316	PRK11316	cl28454	bifunctional heptose 7-phosphate kinase/heptose 1-phosphate adenyltransferase
	superfamily	2	36	0.000525	cl28454	RfaE superfamily	-	ADP-heptose synthase, bifunctional sugar kinase/adenylyltransferase
	non-specific	4	40	0.000654	pfam06574	FAD_syn	cl00015	FAD synthetase
	non-specific	9	64	0.000754	COG1019	CAB4	cl00015	Phosphopantetheine adenylyltransferase
	non-specific	4	40	0.000868	PRK05627	PRK05627	cl27514	bifunctional riboflavin kinase/FMN adenylyltransferase
	superfamily	4	40	0.000868	cl27514	Flavokinase superfamily	-	Riboflavin kinase
	non-specific	6	34	0.000934	cd02173	ECT	cl00015	CTP:phosphoethanolamine cytidylyltransferase (ECT)
	non-specific	4	40	0.001299	cd02064	FAD_synthetase_N	cl00015	FAD synthetase, N-terminal domain of the bifunctional enzyme
	non-specific	9	42	0.001831	COG2870	RfaE	cl28454	ADP-heptose synthase, bifunctional sugar kinase/adenylyltransferase

Query	Hit type	ID region start	ID region end	E-Value	Accession	Short name	Superfamily	Definition
	non-specific	2	79	0.003548	PRK07143	PRK07143	cl27514	hypothetical protein
	non-specific	9	56	0.003727	cd02174	CCT	cl00015	CTP:phosphocholine cytidylyltransferase
	non-specific	9	64	0.004673	cd02164	PPAT_CoAS	cl00015	phosphopantetheine adenylyltransferase
	non-specific	9	54	0.006477	TIGR01518	g3p_cytidyltrns	cl00015	glycerol-3-phosphate cytidylyltransferase
	non-specific	2	42	0.009744	cd02172	RfaE_N	cl00015	N-terminal domain of RfaE
M. iners	non-specific	2	141	1.65e-55	PRK13964	coaD	cl00015	phosphopantetheine adenylyltransferase
	superfamily	2	141	1.65e-55	cl00015	nt_trans superfamily	-	nucleotidyl transferase superfamily
	non-specific	1	139	3.63e-41	COG0669	CoaD	cl00015	Phosphopantetheine adenylyltransferase
	non-specific	2	132	9.44e-39	PRK00168	coaD	cl00015	phosphopantetheine adenylyltransferase
	non-specific	4	132	2.28e-38	cd02163	PPAT	cl00015	Phosphopantetheine adenylyltransferase
	non-specific	4	136	7.71e-33	TIGR01510	coaD_prev_kdtB	cl00015	pantetheine-phosphate adenylyltransferase
	specific	6	141	3.41e-17	pfam01467	CTP_transf_like	cl00015	Cytidylyltransferase-like
	non-specific	1	67	7.10e-12	COG1057	NadD	cl00015	Nicotinic acid mononucleotide adenylyltransferase
	non-specific	4	64	1.05e-11	TIGR00125	cyt_tran_rel	cl00015	cytidyltransferase-like domain
M. iners	non-specific	1	135	1.44e-11	PRK08099	PRK08099	cl28365	bifunctional DNA-binding transcriptional repressor/ NMN adenylyltransferase
	superfamily	1	135	1.44e-11	cl28365	PRK08099 superfamily	-	bifunctional DNA-binding transcriptional repressor/ NMN adenylyltransferase
	non-specific	1	67	4.81e-11	PRK00071	nadD	cl00015	nicotinic acid mononucleotide adenylyltransferase
	non-specific	4	134	2.44e-10	cd02167	NMNAT_NadR	cl00015	Nicotinamide/nicotinate mononucleotide adenylyltransferase
	non-specific	2	67	4.93e-10	PRK07152	nadD	cl28367	putative nicotinate-nucleotide adenylyltransferase
	superfamily	2	67	4.93e-10	cl28367	nadD superfamily	-	putative nicotinate-nucleotide adenylyltransferase
	non-specific	4	134	2.26e-09	cd02039	cytidylyltransferase_like	cl00015	Cytidylyltransferase-like domain
	non-specific	6	67	2.80e-09	TIGR00482	TIGR00482	cl00015	nicotinate (nicotinamide) nucleotide adenylyltransferase
	non-specific	1	63	4.74e-09	COG1056	NadR	cl00015	Nicotinamide mononucleotide adenylyltransferase
	non-specific	4	67	8.32e-09	cd02165	NMNAT	cl00015	Nicotinamide/nicotinate mononucleotide adenylyltransferase
	non-specific	2	36	3.02e-06	TIGR01526	nadR_NMN_Atrans	cl28365	nicotinamide-nucleotide adenylyltransferase
	non-specific	5	63	3.59e-06	PRK01153	PRK01153	cl00015	nicotinamide-nucleotide adenylyltransferase
	non-specific	6	91	3.62e-06	cd02156	nt_trans	cl00015	nucleotidyl transferase superfamily
	non-specific	5	63	1.28e-05	cd02166	NMNAT_Archaea	cl00015	Nicotinamide/nicotinate mononucleotide adenylyltransferase
	non-specific	4	36	0.000109	PRK05379	PRK05379	cl28366	bifunctional nicotinamide mononucleotide adenylyltransferase
	superfamily	4	36	0.000109	cl28366	PRK05379 superfamily	-	bifunctional nicotinamide mononucleotide adenylyltransferase
	non-specific	9	37	0.000179	PRK00777	PRK00777	cl00015	phosphopantetheine adenylyltransferase
	non-specific	1	37	0.000229	cd02170	cytidylyltransferase	cl00015	cytidylyltransferase
	non-specific	5	61	0.000865	TIGR01527	arch_NMN_Atrans	cl00015	nicotinamide-nucleotide adenylyltransferase
	non-specific	3	30	0.001251	PRK06973	PRK06973	cl00015	nicotinic acid mononucleotide adenylyltransferase
	non-specific	9	40	0.002132	pfam06574	FAD_syn	cl00015	FAD synthetase
	non-specific	9	37	0.003561	COG1019	CAB4	cl00015	Phosphopantetheine adenylyltransferase
	non-specific	9	40	0.003964	PRK05627	PRK05627	cl27514	bifunctional riboflavin kinase/FMN adenylyltransferase
	superfamily	9	40	0.003964	cl27514	Flavokinase superfamily	-	Riboflavin kinase
	non-specific	3	42	0.004603	PTZ00308	PTZ00308	cl28626	ethanolamine-phosphate cytidylyltransferase
	superfamily	3	42	0.004603	cl28626	PLN02406 superfamily	-	ethanolamine-phosphate cytidylyltransferase
	non-specific	1	37	0.004662	cd02171	G3P_Cytidylyltransferase	cl00015	glycerol-3-phosphate cytidylyltransferase

Query	Hit type	ID region start	ID region end	E-Value	Accession	Short name	Superfamily	Definition
	non-specific	1	36	0.004933	COG0615	TagD	cl00015	Glycerol-3-phosphate cytidylyltransferase, cytidylyltransferase family
	non-specific	2	40	0.006074	PLN02660	PLN02660	cl00015	pantoate--beta-alanine ligase
M. iowae	non-specific	8	141	5.58e-31	PRK13964	coaD	cl00015	phosphopantetheine adenylyltransferase
	superfamily	8	141	5.58e-31	cl00015	nt_trans superfamily	-	nucleotidyl transferase superfamily
	non-specific	8	136	1.04e-28	COG0669	CoaD	cl00015	Phosphopantetheine adenylyltransferase
	non-specific	8	140	9.33e-27	TIGR01510	coaD_prev_kdtB	cl00015	pantetheine-phosphate adenylyltransferase
	non-specific	8	136	7.25e-26	cd02163	PPAT	cl00015	Phosphopantetheine adenylyltransferase
	non-specific	8	136	1.01e-23	PRK00168	coaD	cl00015	phosphopantetheine adenylyltransferase
M. iowae	non-specific	9	138	2.07e-07	pfam01467	CTP_transf_like	cl00015	Cytidylyltransferase-like
	non-specific	8	93	0.001153	cd02156	nt_trans	cl00015	nucleotidyl transferase superfamily
	non-specific	8	64	0.001154	TIGR00125	cyt_tran_rel	cl00015	cytidyltransferase-like domain
	non-specific	8	136	0.001185	cd02039	cytidylyltransferase_like	cl00015	Cytidylyltransferase-like domain
M. leachii	specific	1	140	3.91e-73	PRK13964	coaD	cl00015	phosphopantetheine adenylyltransferase
	superfamily	1	140	3.91e-73	cl00015	nt_trans superfamily	-	nucleotidyl transferase superfamily
	non-specific	3	140	4.52e-64	TIGR01510	coaD_prev_kdtB	cl00015	pantetheine-phosphate adenylyltransferase
	non-specific	1	139	8.52e-46	COG0669	CoaD	cl00015	Phosphopantetheine adenylyltransferase
	non-specific	1	135	4.75e-44	PRK00168	coaD	cl00015	phosphopantetheine adenylyltransferase
	non-specific	3	139	1.50e-43	cd02163	PPAT	cl00015	Phosphopantetheine adenylyltransferase
	specific	5	135	8.49e-21	pfam01467	CTP_transf_like	cl00015	Cytidylyltransferase-like
	non-specific	4	63	1.33e-13	TIGR00125	cyt_tran_rel	cl00015	cytidyltransferase-like domain
	non-specific	4	134	6.52e-12	cd02039	cytidylyltransferase_like	cl00015	Cytidylyltransferase-like domain
	non-specific	1	77	1.09e-09	PRK07152	nadD	cl28367	putative nicotinate-nucleotide adenylyltransferase
	superfamily	1	77	1.09e-09	cl28367	nadD superfamily	-	putative nicotinate-nucleotide adenylyltransferase
	non-specific	1	66	1.65e-07	COG1057	NadD	cl00015	Nicotinic acid mononucleotide adenylyltransferase
	non-specific	3	77	4.15e-07	cd02165	NMNAT	cl00015	Nicotinamide/nicotinate mononucleotide adenylyltransferase
	non-specific	1	136	5.19e-07	PRK00777	PRK00777	cl00015	phosphopantetheine adenylyltransferase
	non-specific	1	46	1.35e-06	COG1056	NadR	cl00015	Nicotinamide mononucleotide adenylyltransferase
	non-specific	1	66	1.57e-06	PRK00071	nadD	cl00015	nicotinic acid mononucleotide adenylyltransferase
	non-specific	1	136	6.25e-06	COG1019	CAB4	cl00015	Phosphopantetheine adenylyltransferase
	non-specific	3	130	8.03e-06	cd02156	nt_trans	cl00015	nucleotidyl transferase superfamily
	non-specific	3	134	2.76e-05	cd02167	NMNAT_NadR	cl00015	Nicotinamide/nicotinate mononucleotide adenylyltransferase
	non-specific	4	68	4.23e-05	cd02166	NMNAT_Archaea	cl00015	Nicotinamide/nicotinate mononucleotide adenylyltransferase
	non-specific	1	71	7.22e-05	TIGR01526	nadR_NMN_Atrans	cl28365	nicotinamide-nucleotide adenylyltransferase
	superfamily	1	71	7.22e-05	cl28365	PRK08099 superfamily	-	bifunctional DNA-binding transcriptional repressor/ NMN adenylyltransferase
	non-specific	4	68	0.00016	TIGR01527	arch_NMN_Atrans	cl00015	nicotinamide-nucleotide adenylyltransferase
	non-specific	4	35	0.000363	PRK01153	PRK01153	cl00015	nicotinamide-nucleotide adenylyltransferase
	non-specific	4	35	0.00079	PRK05379	PRK05379	cl28366	bifunctional nicotinamide mononucleotide adenylyltransferase
	superfamily	4	35	0.00079	cl28366	PRK05379 superfamily	-	bifunctional nicotinamide mononucleotide adenylyltransferase
	non-specific	5	66	0.001253	TIGR00482	TIGR00482	cl00015	nicotinate (nicotinamide) nucleotide adenylyltransferase
	non-specific	1	89	0.00256	cd02170	cytidylyltransferase	cl00015	cytidylyltransferase
	non-specific	1	36	0.002969	cd02171	G3P_Cytidylyltransferase	cl00015	glycerol-3-phosphate cytidylyltransferase

Query	Hit type	ID region start	ID region end	E-Value	Accession	Short name	Superfamily	Definition
M. leonicaptivi	non-specific	2	134	1.38e-46	COG0669	CoaD	cl00015	Phosphopantetheine adenylyltransferase
	superfamily	2	134	1.38e-46	cl00015	nt_trans superfamily	-	nucleotidyl transferase superfamily
	non-specific	5	134	7.67e-46	cd02163	PPAT	cl00015	Phosphopantetheine adenylyltransferase
	non-specific	3	134	3.09e-44	PRK00168	coaD	cl00015	phosphopantetheine adenylyltransferase
M. leonicaptivi	non-specific	3	137	4.65e-42	PRK13964	coaD	cl00015	phosphopantetheine adenylyltransferase
	non-specific	5	134	2.30e-41	TIGR01510	coaD_prev_kdtB	cl00015	pantetheine-phosphate adenylyltransferase
	specific	8	136	9.01e-24	pfam01467	CTP_transf_like	cl00015	Cytidylyltransferase-like
	non-specific	5	135	1.45e-12	cd02039	cytidylyltransferase_like	cl00015	Cytidylyltransferase-like domain
	non-specific	5	48	1.59e-11	TIGR00125	cyt_tran_rel	cl00015	cytidyltransferase-like domain
	non-specific	1	46	2.03e-10	PRK00071	nadD	cl00015	nicotinic acid mononucleotide adenylyltransferase
	non-specific	1	48	5.65e-09	COG1057	NadD	cl00015	Nicotinic acid mononucleotide adenylyltransferase
	non-specific	6	48	9.93e-08	PRK01153	PRK01153	cl00015	nicotinamide-nucleotide adenylyltransferase
	non-specific	1	48	2.84e-07	COG1056	NadR	cl00015	Nicotinamide mononucleotide adenylyltransferase
	non-specific	6	48	5.20e-07	cd02166	NMNAT_Archaea	cl00015	Nicotinamide/nicotinate mononucleotide adenylyltransferase
	non-specific	1	86	8.65e-07	COG0615	TagD	cl00015	Glycerol-3-phosphate cytidylyltransferase, cytidylyltransferase family
	non-specific	5	46	1.25e-06	cd02165	NMNAT	cl00015	Nicotinamide/nicotinate mononucleotide adenylyltransferase
	non-specific	10	137	1.71e-06	COG1019	CAB4	cl00015	Phosphopantetheine adenylyltransferase
	non-specific	4	48	5.31e-06	PRK05379	PRK05379	cl28366	bifunctional nicotinamide mononucleotide adenylyltransferase
	superfamily	4	48	5.31e-06	cl28366	PRK05379 superfamily	-	bifunctional nicotinamide mononucleotide adenylyltransferase
	non-specific	3	88	1.99e-05	PRK07152	nadD	cl28367	putative nicotinate-nucleotide adenylyltransferase
	superfamily	3	88	1.99e-05	cl28367	nadD superfamily	-	putative nicotinate-nucleotide adenylyltransferase
	non-specific	5	48	2.27e-05	cd02168	NMNAT_Nudix	cl00015	Nicotinamide/nicotinate mononucleotide adenylyltransferase
	non-specific	1	38	3.74e-05	cd02170	cytidylyltransferase	cl00015	cytidylyltransferase
	non-specific	10	137	5.81e-05	PRK00777	PRK00777	cl00015	phosphopantetheine adenylyltransferase
	non-specific	8	49	6.14e-05	TIGR00482	TIGR00482	cl00015	nicotinate (nicotinamide) nucleotide adenylyltransferase
	non-specific	6	132	9.09e-05	cd02156	nt_trans	cl00015	nucleotidyl transferase superfamily
	non-specific	8	69	0.00066	TIGR01527	arch_NMN_Atrans	cl00015	nicotinamide-nucleotide adenylyltransferase
	non-specific	1	135	0.00071	PRK08099	PRK08099	cl28365	bifunctional DNA-binding transcriptional repressor/ NMN adenylyltransferase
	superfamily	1	135	0.00071	cl28365	PRK08099 superfamily	-	bifunctional DNA-binding transcriptional repressor/ NMN adenylyltransferase
	non-specific	10	137	0.001165	PRK01170	PRK01170	cl00866	phosphopantetheine adenylyltransferase
	superfamily	10	137	0.001165	cl00866	NTPase_I-T superfamily	-	Protein of unknown function DUF84
	non-specific	3	57	0.001427	TIGR01526	nadR_NMN_Atrans	cl28365	nicotinamide-nucleotide adenylyltransferase
	non-specific	12	38	0.005519	pfam05636	HIGH_NTase1	cl27012	HIGH Nucleotidyl Transferase
	superfamily	12	38	0.005519	cl27012	HIGH_NTase1 superfamily	-	HIGH Nucleotidyl Transferase
	non-specific	2	37	0.006823	cd02173	ECT	cl00015	CTP:phosphoethanolamine cytidylyltransferase (ECT)
	non-specific	7	135	0.006888	cd02164	PPAT_CoAS	cl00015	phosphopantetheine adenylyltransferase
	non-specific	2	80	0.007169	cd02169	Citrate_lyase_ligase	cl00015	Citrate lyase ligase
	non-specific	10	38	0.008445	cd09286	NMNAT_Eukarya	cl00015	Nicotinamide/nicotinate mononucleotide adenylyltransferase
M. lipofaciens	non-specific	1	140	9.74e-63	PRK13964	coaD	cl00015	phosphopantetheine adenylyltransferase
M. lipofaciens	superfamily	1	140	9.74e-63	cl00015	nt_trans superfamily	-	nucleotidyl transferase superfamily

Query	Hit type	ID region start	ID region end	E-Value	Accession	Short name	Superfamily	Definition
	non-specific	1	134	4.02e-43	COG0669	CoaD	cl00015	Phosphopantetheine adenylyltransferase
	non-specific	3	133	1.48e-38	cd02163	PPAT	cl00015	Phosphopantetheine adenylyltransferase
	non-specific	3	133	1.16e-37	TIGR01510	coaD_prev_kdtB	cl00015	pantetheine-phosphate adenylyltransferase
	non-specific	1	133	2.28e-37	PRK00168	coaD	cl00015	phosphopantetheine adenylyltransferase
	non-specific	5	135	1.17e-15	pfam01467	CTP_transf_like	cl00015	Cytidylyltransferase-like
	non-specific	3	63	6.62e-12	TIGR00125	cyt_tran_rel	cl00015	cytidyltransferase-like domain
	non-specific	1	66	1.19e-09	COG1057	NadD	cl00015	Nicotinic acid mononucleotide adenylyltransferase
	non-specific	3	133	4.38e-08	cd02039	cytidylyltransferase_like	cl00015	Cytidylyltransferase-like domain
	non-specific	3	90	7.99e-08	cd02156	nt_trans	cl00015	nucleotidyl transferase superfamily
	non-specific	5	133	1.98e-07	cd02167	NMNAT_NadR	cl00015	Nicotinamide/nicotinate mononucleotide adenylyltransferase
	non-specific	4	66	2.34e-07	cd02165	NMNAT	cl00015	Nicotinamide/nicotinate mononucleotide adenylyltransferase
	non-specific	1	66	1.08e-06	PRK07152	nadD	cl28367	putative nicotinate-nucleotide adenylyltransferase
	superfamily	1	66	1.08e-06	cl28367	nadD superfamily	-	putative nicotinate-nucleotide adenylyltransferase
	non-specific	1	35	1.39e-06	COG1056	NadR	cl00015	Nicotinamide mononucleotide adenylyltransferase
	non-specific	1	133	1.77e-05	cd02170	cytidylyltransferase	cl00015	cytidylyltransferase
	non-specific	1	66	2.74e-05	PRK00071	nadD	cl00015	nicotinic acid mononucleotide adenylyltransferase
	non-specific	5	52	3.21e-05	TIGR00482	TIGR00482	cl00015	nicotinate (nicotinamide) nucleotide adenylyltransferase
	non-specific	1	43	8.20e-05	COG0615	TagD	cl00015	Glycerol-3-phosphate cytidylyltransferase, cytidylyltransferase family
	non-specific	13	39	9.36e-05	pfam02569	Pantoate_ligase	cl00015	Pantoate-beta-alanine ligase
	non-specific	1	133	0.000121	TIGR01526	nadR_NMN_Atrans	cl28365	nicotinamide-nucleotide adenylyltransferase
	superfamily	1	133	0.000121	cl28365	PRK08099 superfamily	-	bifunctional DNA-binding transcriptional repressor/ NMN adenylyltransferase
	non-specific	2	35	0.000121	PRK01153	PRK01153	cl00015	nicotinamide-nucleotide adenylyltransferase
	non-specific	1	72	0.000135	cd02171	G3P_Cytidylyltransferase	cl00015	glycerol-3-phosphate cytidylyltransferase
	non-specific	34	94	0.000149	PRK10422	PRK10422	cl10013	lipopolysaccharide core biosynthesis protein
	superfamily	34	94	0.000149	cl10013	Glycosyltransferase_GTB_type superfamily	-	Glycosyltransferases catalyze the transfer of sugar moieties from activated donor molecules to specific acceptor molecules, forming glycosidic bonds.
	non-specific	5	61	0.000181	PLN02388	PLN02388	cl00015	phosphopantetheine adenylyltransferase
	non-specific	3	35	0.000184	cd02166	NMNAT_Archaea	cl00015	Nicotinamide/nicotinate mononucleotide adenylyltransferase
	non-specific	1	63	0.000198	PRK00777	PRK00777	cl00015	phosphopantetheine adenylyltransferase
	non-specific	4	35	0.00022	PRK05379	PRK05379	cl28366	bifunctional nicotinamide mononucleotide adenylyltransferase
	superfamily	4	35	0.00022	cl28366	PRK05379 superfamily	-	bifunctional nicotinamide mononucleotide adenylyltransferase
	non-specific	3	46	0.000235	TIGR01527	arch_NMN_Atrans	cl00015	nicotinamide-nucleotide adenylyltransferase
	non-specific	1	69	0.000255	COG1019	CAB4	cl00015	Phosphopantetheine adenylyltransferase
	non-specific	7	67	0.001261	cd02164	PPAT_CoAS	cl00015	phosphopantetheine adenylyltransferase
	non-specific	13	80	0.001583	PLN02660	PLN02660	cl00015	pantoate--beta-alanine ligase
M. lipofaciens	non-specific	13	39	0.002191	COG0414	PanC	cl00015	Panthothenate synthetase
	non-specific	5	72	0.002316	TIGR01518	g3p_cytidyltrns	cl00015	glycerol-3-phosphate cytidylyltransferase
	non-specific	13	39	0.003169	cd00560	PanC	cl00015	Pantoate-beta-alanine ligase
	non-specific	13	39	0.005229	PRK00380	panC	cl00015	pantoate--beta-alanine ligase
M. mobile	non-specific	9	96	1.63e-14	PRK13964	coaD	cl00015	phosphopantetheine adenylyltransferase

Query	Hit type	ID region start	ID region end	E-Value	Accession	Short name	Superfamily	Definition
	superfamily	9	96	1.63e-14	cl00015	nt_trans superfamily	-	nucleotidyl transferase superfamily
	non-specific	11	96	8.75e-14	TIGR01510	coaD_prev_kdtB	cl00015	pantetheine-phosphate adenylyltransferase
	non-specific	8	96	1.02e-12	COG0669	CoaD	cl00015	Phosphopantetheine adenylyltransferase
	non-specific	11	96	3.11e-12	cd02163	PPAT	cl00015	Phosphopantetheine adenylyltransferase
	non-specific	8	96	1.73e-11	PRK00168	coaD	cl00015	phosphopantetheine adenylyltransferase
	non-specific	11	58	2.49e-05	TIGR00125	cyt_tran_rel	cl00015	cytidyltransferase-like domain
M. molare	non-specific	1	140	2.91e-62	PRK13964	coaD	cl00015	phosphopantetheine adenylyltransferase
	superfamily	1	140	2.91e-62	cl00015	nt_trans superfamily	-	nucleotidyl transferase superfamily
	non-specific	1	140	3.14e-45	COG0669	CoaD	cl00015	Phosphopantetheine adenylyltransferase
	non-specific	3	131	5.35e-42	cd02163	PPAT	cl00015	Phosphopantetheine adenylyltransferase
	non-specific	1	131	2.39e-40	PRK00168	coaD	cl00015	phosphopantetheine adenylyltransferase
	non-specific	3	133	2.95e-38	TIGR01510	coaD_prev_kdtB	cl00015	pantetheine-phosphate adenylyltransferase
	specific	5	135	8.78e-22	pfam01467	CTP_transf_like	cl00015	Cytidylyltransferase-like
	non-specific	3	63	7.16e-12	TIGR00125	cyt_tran_rel	cl00015	cytidyltransferase-like domain
	non-specific	3	133	1.09e-09	cd02039	cytidylyltransferase_like	cl00015	Cytidylyltransferase-like domain
	non-specific	1	44	7.06e-07	PRK00071	nadD	cl00015	nicotinic acid mononucleotide adenylyltransferase
	non-specific	3	90	1.32e-06	cd02156	nt_trans	cl00015	nucleotidyl transferase superfamily
	non-specific	1	40	1.97e-06	COG1056	NadR	cl00015	Nicotinamide mononucleotide adenylyltransferase
	non-specific	4	66	2.69e-06	COG1057	NadD	cl00015	Nicotinic acid mononucleotide adenylyltransferase
	non-specific	4	66	4.66e-06	cd02165	NMNAT	cl00015	Nicotinamide/nicotinate mononucleotide adenylyltransferase
	non-specific	1	66	8.56e-06	PRK07152	nadD	cl28367	putative nicotinate-nucleotide adenylyltransferase
	superfamily	1	66	8.56e-06	cl28367	nadD superfamily	-	putative nicotinate-nucleotide adenylyltransferase
	non-specific	3	35	1.19e-05	PRK01153	PRK01153	cl00015	nicotinamide-nucleotide adenylyltransferase
	non-specific	3	35	2.27e-05	cd02166	NMNAT_Archaea	cl00015	Nicotinamide/nicotinate mononucleotide adenylyltransferase
	non-specific	5	66	5.12e-05	TIGR00482	TIGR00482	cl00015	nicotinate (nicotinamide) nucleotide adenylyltransferase
	non-specific	7	41	0.000146	cd02173	ECT	cl00015	CTP:phosphoethanolamine cytidylyltransferase (ECT)
	non-specific	1	36	0.00028	PRK00777	PRK00777	cl00015	phosphopantetheine adenylyltransferase
	non-specific	4	35	0.000284	PRK05379	PRK05379	cl28366	bifunctional nicotinamide mononucleotide adenylyltransferase
	superfamily	4	35	0.000284	cl28366	PRK05379 superfamily	-	bifunctional nicotinamide mononucleotide adenylyltransferase
	non-specific	12	67	0.000349	smart00764	Citrate_ly_lig	cl00015	Citrate lyase ligase C-terminal domain
	non-specific	12	66	0.000385	pfam08218	Citrate_ly_lig	cl00015	Citrate lyase ligase C-terminal domain
	non-specific	8	44	0.000398	TIGR02199	rfaE_dom_II	cl00015	rfaE bifunctional protein, domain II
	non-specific	1	85	0.000414	COG0615	TagD	cl00015	Glycerol-3-phosphate cytidylyltransferase, cytidylyltransferase family
M. molare	non-specific	12	71	0.000594	cd02169	Citrate_lyase_ligase	cl00015	Citrate lyase ligase
	non-specific	1	57	0.001273	cd02170	cytidylyltransferase	cl00015	cytidylyltransferase
	non-specific	8	41	0.001836	cd02174	CCT	cl00015	CTP:phosphocholine cytidylyltransferase
	non-specific	7	41	0.001934	PTZ00308	PTZ00308	cl28626	ethanolamine-phosphate cytidylyltransferase
	superfamily	7	41	0.001934	cl28626	PLN02406 superfamily	-	ethanolamine-phosphate cytidylyltransferase
	non-specific	8	133	0.002042	COG2870	RfaE	cl28454	ADP-heptose synthase, bifunctional sugar kinase/adenylyltransferase
	superfamily	8	133	0.002042	cl28454	RfaE superfamily	-	ADP-heptose synthase, bifunctional sugar kinase/adenylyltransferase

Query	Hit type	ID region start	ID region end	E-Value	Accession	Short name	Superfamily	Definition
	non-specific	11	64	0.002251	COG3053	CitC	cl28578	Citrate lyase synthetase [Energy production and conversion]
	superfamily	11	64	0.002251	cl28578	CitC superfamily	-	Citrate lyase synthetase [Energy production and conversion]
	non-specific	1	55	0.005139	cd02171	G3P_Cytidylyltransferase	cl00015	glycerol-3-phosphate cytidylyltransferase
M. mycoides subsp. *capri*	specific	1	140	2.94e-71	PRK13964	coaD	cl00015	phosphopantetheine adenylyltransferase
	superfamily	1	140	2.94e-71	cl00015	nt_trans superfamily	-	nucleotidyl transferase superfamily
	non-specific	3	140	1.91e-62	TIGR01510	coaD_prev_kdtB	cl00015	pantetheine-phosphate adenylyltransferase
	non-specific	1	139	1.17e-45	COG0669	CoaD	cl00015	Phosphopantetheine adenylyltransferase
	non-specific	1	135	6.52e-44	PRK00168	coaD	cl00015	phosphopantetheine adenylyltransferase
	non-specific	3	139	1.02e-42	cd02163	PPAT	cl00015	Phosphopantetheine adenylyltransferase
	specific	5	135	7.19e-20	pfam01467	CTP_transf_like	cl00015	Cytidylyltransferase-like
	non-specific	3	63	2.98e-13	TIGR00125	cyt_tran_rel	cl00015	cytidyltransferase-like domain
	non-specific	3	134	8.76e-12	cd02039	cytidylyltransferase_like	cl00015	Cytidylyltransferase-like domain
	non-specific	1	77	6.01e-10	PRK07152	nadD	cl28367	putative nicotinate-nucleotide adenylyltransferase
	superfamily	1	77	6.01e-10	cl28367	nadD superfamily	-	putative nicotinate-nucleotide adenylyltransferase
	non-specific	1	66	1.52e-08	COG1057	NadD	cl00015	Nicotinic acid mononucleotide adenylyltransferase
	non-specific	1	136	4.04e-08	PRK00777	PRK00777	cl00015	phosphopantetheine adenylyltransferase
	non-specific	3	77	1.56e-07	cd02165	NMNAT	cl00015	Nicotinamide/nicotinate mononucleotide adenylyltransferase
	non-specific	1	44	2.58e-07	COG1056	NadR	cl00015	Nicotinamide mononucleotide adenylyltransferase
	non-specific	1	136	6.67e-07	COG1019	CAB4	cl00015	Phosphopantetheine adenylyltransferase
	non-specific	1	66	1.60e-06	PRK00071	nadD	cl00015	nicotinic acid mononucleotide adenylyltransferase
	non-specific	1	71	3.91e-05	TIGR01526	nadR_NMN_Atrans	cl28365	nicotinamide-nucleotide adenylyltransferase
	superfamily	1	71	3.91e-05	cl28365	PRK08099 superfamily	-	bifunctional DNA-binding transcriptional repressor/ NMN adenylyltransferase
	non-specific	4	61	5.48e-05	cd02166	NMNAT_Archaea	cl00015	Nicotinamide/nicotinate mononucleotide adenylyltransferase
	non-specific	3	134	6.30e-05	cd02167	NMNAT_NadR	cl00015	Nicotinamide/nicotinate mononucleotide adenylyltransferase
	non-specific	4	130	6.91e-05	cd02156	nt_trans	cl00015	nucleotidyl transferase superfamily
	non-specific	4	68	7.26e-05	TIGR01527	arch_NMN_Atrans	cl00015	nicotinamide-nucleotide adenylyltransferase
	non-specific	3	35	0.00049	PRK05379	PRK05379	cl28366	bifunctional nicotinamide mononucleotide adenylyltransferase
M. mycoides subsp. *capri*	superfamily	3	35	0.00049	cl28366	PRK05379 superfamily	-	bifunctional nicotinamide mononucleotide adenylyltransferase
	non-specific	4	35	0.000534	PRK01153	PRK01153	cl00015	nicotinamide-nucleotide adenylyltransferase
	non-specific	1	76	0.000596	PRK08887	PRK08887	cl00015	nicotinic acid mononucleotide adenylyltransferase
	non-specific	8	89	0.001492	cd02170	cytidylyltransferase	cl00015	cytidylyltransferase
	non-specific	2	67	0.001944	PRK01170	PRK01170	cl00866	phosphopantetheine adenylyltransferase
	superfamily	2	67	0.001944	cl00866	NTPase_I-T superfamily	-	Protein of unknown function DUF84
	non-specific	8	133	0.002054	cd02164	PPAT_CoAS	cl00015	phosphopantetheine adenylyltransferase
	non-specific	1	36	0.003852	cd02171	G3P_Cytidylyltransferase	cl00015	glycerol-3-phosphate cytidylyltransferase
	non-specific	5	36	0.003895	TIGR00482	TIGR00482	cl00015	nicotinate (nicotinamide) nucleotide adenylyltransferase
M. mycoides subsp. *mycoides*	specific	1	140	4.77e-72	PRK13964	coaD	cl00015	phosphopantetheine adenylyltransferase
	superfamily	1	140	4.77e-72	cl00015	nt_trans superfamily	-	nucleotidyl transferase superfamily
	non-specific	3	140	8.20e-62	TIGR01510	coaD_prev_kdtB	cl00015	pantetheine-phosphate adenylyltransferase
	non-specific	1	139	1.19e-45	COG0669	CoaD	cl00015	Phosphopantetheine adenylyltransferase
	non-specific	1	135	3.07e-43	PRK00168	coaD	cl00015	phosphopantetheine adenylyltransferase

Query	Hit type	ID region start	ID region end	E-Value	Accession	Short name	Superfamily	Definition
	non-specific	3	139	1.96e-42	cd02163	PPAT	cl00015	Phosphopantetheine adenylyltransferase
	specific	5	135	1.38e-20	pfam01467	CTP_transf_like	cl00015	Cytidylyltransferase-like
	non-specific	4	63	8.39e-13	TIGR00125	cyt_tran_rel	cl00015	cytidylyltransferase-like domain
	non-specific	4	134	2.80e-12	cd02039	cytidylyltransferase_like	cl00015	Cytidylyltransferase-like domain
	non-specific	1	124	2.63e-10	PRK07152	nadD	cl28367	putative nicotinate-nucleotide adenylyltransferase
	superfamily	1	124	2.63e-10	cl28367	nadD superfamily	-	putative nicotinate-nucleotide adenylyltransferase
	non-specific	1	66	7.45e-08	COG1057	NadD	cl00015	Nicotinic acid mononucleotide adenylyltransferase
	non-specific	1	66	1.14e-06	PRK00071	nadD	cl00015	nicotinic acid mononucleotide adenylyltransferase
	non-specific	3	77	3.95e-06	cd02165	NMNAT	cl00015	Nicotinamide/nicotinate mononucleotide adenylyltransferase
	non-specific	3	134	4.26e-06	cd02167	NMNAT_NadR	cl00015	Nicotinamide/nicotinate mononucleotide adenylyltransferase
	non-specific	1	136	8.25e-06	PRK00777	PRK00777	cl00015	phosphopantetheine adenylyltransferase
	non-specific	1	35	1.04e-05	COG1056	NadR	cl00015	Nicotinamide mononucleotide adenylyltransferase
	non-specific	3	130	1.73e-05	cd02156	nt_trans	cl00015	nucleotidyl transferase superfamily
	non-specific	1	71	0.000106	TIGR01526	nadR_NMN_Atrans	cl28365	nicotinamide-nucleotide adenylyltransferase
	superfamily	1	71	0.000106	cl28365	PRK08099 superfamily	-	bifunctional DNA-binding transcriptional repressor/ NMN adenylyltransferase
	non-specific	5	119	0.000126	TIGR00482	TIGR00482	cl00015	nicotinate (nicotinamide) nucleotide adenylyltransferase
	non-specific	4	35	0.000356	PRK01153	PRK01153	cl00015	nicotinamide-nucleotide adenylyltransferase
	non-specific	1	136	0.000358	COG1019	CAB4	cl00015	Phosphopantetheine adenylyltransferase
	non-specific	4	35	0.000496	cd02166	NMNAT_Archaea	cl00015	Nicotinamide/nicotinate mononucleotide adenylyltransferase
	non-specific	4	35	0.000519	PRK05379	PRK05379	cl28366	bifunctional nicotinamide mononucleotide adenylyltransferase
	superfamily	4	35	0.000519	cl28366	PRK05379 superfamily	-	bifunctional nicotinamide mononucleotide adenylyltransferase
	non-specific	1	36	0.002311	cd02171	G3P_Cytidylyltransferase	cl00015	glycerol-3-phosphate cytidylyltransferase
M. mycoides subsp. *mycoides*	non-specific	1	36	0.003558	cd02170	cytidylyltransferase	cl00015	cytidylyltransferase
	non-specific	10	35	0.006714	pfam05636	HIGH_NTase1	cl27012	HIGH Nucleotidyl Transferase
	superfamily	10	35	0.006714	cl27012	HIGH_NTase1 superfamily	-	HIGH Nucleotidyl Transferase
	non-specific	4	68	0.007739	TIGR01527	arch_NMN_Atrans	cl00015	nicotinamide-nucleotide adenylyltransferase
	non-specific	10	35	0.007951	PRK13670	PRK13670	cl27012	hypothetical protein
M. opalescens	non-specific	1	141	1.49e-55	PRK13964	coaD	cl00015	phosphopantetheine adenylyltransferase
	superfamily	1	141	1.49e-55	cl00015	nt_trans superfamily	-	nucleotidyl transferase superfamily
	non-specific	1	135	7.10e-39	COG0669	CoaD	cl00015	Phosphopantetheine adenylyltransferase
	non-specific	3	134	7.30e-35	TIGR01510	coaD_prev_kdtB	cl00015	pantetheine-phosphate adenylyltransferase
	non-specific	3	132	3.82e-34	cd02163	PPAT	cl00015	Phosphopantetheine adenylyltransferase
	non-specific	1	132	2.96e-32	PRK00168	coaD	cl00015	phosphopantetheine adenylyltransferase
	non-specific	5	136	2.16e-15	pfam01467	CTP_transf_like	cl00015	Cytidylyltransferase-like
	non-specific	3	51	2.96e-10	TIGR00125	cyt_tran_rel	cl00015	cytidyltransferase-like domain
	non-specific	1	43	7.33e-09	COG1057	NadD	cl00015	Nicotinic acid mononucleotide adenylyltransferase
	non-specific	1	67	2.07e-08	PRK07152	nadD	cl28367	putative nicotinate-nucleotide adenylyltransferase
	superfamily	1	67	2.07e-08	cl28367	nadD superfamily	-	putative nicotinate-nucleotide adenylyltransferase
	non-specific	5	67	3.93e-08	TIGR00482	TIGR00482	cl00015	nicotinate (nicotinamide) nucleotide adenylyltransferase
	non-specific	5	134	2.86e-07	cd02167	NMNAT_NadR	cl00015	Nicotinamide/nicotinate mononucleotide adenylyltransferase
	non-specific	4	67	4.08e-07	cd02165	NMNAT	cl00015	Nicotinamide/nicotinate mononucleotide adenylyltransferase
	non-specific	1	35	3.27e-06	COG1056	NadR	cl00015	Nicotinamide mononucleotide adenylyltransferase

Query	Hit type	ID region start	ID region end	E-Value	Accession	Short name	Superfamily	Definition
	non-specific	1	44	4.36e-06	PRK00071	nadD	cl00015	nicotinic acid mononucleotide adenylyltransferase
	non-specific	3	134	5.17e-06	cd02039	cytidylyltransferase_like	cl00015	Cytidylyltransferase-like domain
	non-specific	3	39	5.97e-06	cd02156	nt_trans	cl00015	nucleotidyl transferase superfamily
	non-specific	1	61	6.36e-06	cd02171	G3P_Cytidylyltransferase	cl00015	glycerol-3-phosphate cytidylyltransferase
	non-specific	1	88	2.33e-05	cd02170	cytidylyltransferase	cl00015	cytidylyltransferase
	non-specific	1	36	3.02e-05	COG0615	TagD	cl00015	Glycerol-3-phosphate cytidylyltransferase, cytidylyltransferase family
	non-specific	1	33	0.000173	PRK00777	PRK00777	cl00015	phosphopantetheine adenylyltransferase
	non-specific	1	37	0.000471	TIGR01526	nadR_NMN_Atrans	cl28365	nicotinamide-nucleotide adenylyltransferase
	superfamily	1	37	0.000471	cl28365	PRK08099 superfamily	-	bifunctional DNA-binding transcriptional repressor/ NMN adenylyltransferase
	non-specific	5	61	0.001081	TIGR01518	g3p_cytidyltrns	cl00015	glycerol-3-phosphate cytidylyltransferase
	non-specific	14	42	0.001136	pfam02569	Pantoate_ligase	cl00015	Pantoate-beta-alanine ligase
	non-specific	2	29	0.001861	PRK01153	PRK01153	cl00015	nicotinamide-nucleotide adenylyltransferase
	non-specific	8	50	0.002073	TIGR02199	rfaE_dom_II	cl00015	rfaE bifunctional protein, domain II
	non-specific	4	35	0.00227	PRK05379	PRK05379	cl28366	bifunctional nicotinamide mononucleotide adenylyltransferase
	superfamily	4	35	0.00227	cl28366	PRK05379 superfamily	-	bifunctional nicotinamide mononucleotide adenylyltransferase
	non-specific	1	42	0.002519	PRK08099	PRK08099	cl28365	bifunctional DNA-binding transcriptional repressor/ NMN adenylyltransferase
M. opalescens	non-specific	3	35	0.004583	cd02166	NMNAT_Archaea	cl00015	Nicotinamide/nicotinate mononucleotide adenylyltransferase
M. penetrans	non-specific	10	136	8.33e-36	TIGR01510	coaD_prev_kdtB	cl00015	pantetheine-phosphate adenylyltransferase
	superfamily	10	136	8.33e-36	cl00015	nt_trans superfamily	-	nucleotidyl transferase superfamily
	non-specific	6	135	1.92e-31	PRK00168	coaD	cl00015	phosphopantetheine adenylyltransferase
	non-specific	7	135	1.58e-30	COG0669	CoaD	cl00015	Phosphopantetheine adenylyltransferase
	non-specific	11	136	4.53e-29	cd02163	PPAT	cl00015	Phosphopantetheine adenylyltransferase
	non-specific	7	146	3.67e-25	PRK13964	coaD	cl00015	phosphopantetheine adenylyltransferase
	non-specific	11	139	2.00e-11	pfam01467	CTP_transf_like	cl00015	Cytidylyltransferase-like
	non-specific	14	140	2.37e-08	cd02170	cytidylyltransferase	cl00015	cytidylyltransferase
	non-specific	11	42	3.71e-08	TIGR00125	cyt_tran_rel	cl00015	cytidyltransferase-like domain
	non-specific	14	140	1.07e-06	COG0615	TagD	cl00015	Glycerol-3-phosphate cytidylyltransferase, cytidylyltransferase family
	non-specific	10	141	4.00e-06	cd02039	cytidylyltransferase_like	cl00015	Cytidylyltransferase-like domain
	non-specific	5	41	1.80e-05	COG1056	NadR	cl00015	Nicotinamide mononucleotide adenylyltransferase
	non-specific	11	95	3.02e-05	cd02156	nt_trans	cl00015	nucleotidyl transferase superfamily
	non-specific	14	76	4.52e-05	PRK00777	PRK00777	cl00015	phosphopantetheine adenylyltransferase
	non-specific	14	89	0.000143	COG1019	CAB4	cl00015	Phosphopantetheine adenylyltransferase
	non-specific	8	93	0.000157	cd02171	G3P_Cytidylyltransferase	cl00015	glycerol-3-phosphate cytidylyltransferase
	non-specific	12	41	0.001478	cd02166	NMNAT_Archaea	cl00015	Nicotinamide/nicotinate mononucleotide adenylyltransferase
	non-specific	9	41	0.00178	PRK05379	PRK05379	cl28366	bifunctional nicotinamide mononucleotide adenylyltransferase
	superfamily	9	41	0.00178	cl28366	PRK05379 superfamily	-	bifunctional nicotinamide mononucleotide adenylyltransferase
	non-specific	6	73	0.003098	PRK00071	nadD	cl00015	nicotinic acid mononucleotide adenylyltransferase
	non-specific	14	45	0.004532	cd02174	CCT	cl00015	CTP:phosphocholine cytidylyltransferase
	non-specific	10	93	0.004773	cd02165	NMNAT	cl00015	Nicotinamide/nicotinate mononucleotide adenylyltransferase

Query	Hit type	ID region start	ID region end	E-Value	Accession	Short name	Superfamily	Definition
	non-specific	2	83	0.005485	PRK07143	PRK07143	cl27514	hypothetical protein
	superfamily	2	83	0.005485	cl27514	Flavokinase superfamily	-	Riboflavin kinase
	non-specific	13	50	0.005531	cd02173	ECT	cl00015	CTP:phosphoethanolamine cytidylyltransferase (ECT)
	non-specific	6	68	0.005561	COG1057	NadD	cl00015	Nicotinic acid mononucleotide adenylyltransferase
	non-specific	11	41	0.006622	PRK01153	PRK01153	cl00015	nicotinamide-nucleotide adenylyltransferase
	non-specific	14	93	0.006986	TIGR01518	g3p_cytidyltrns	cl00015	glycerol-3-phosphate cytidylyltransferase
M. pirum	non-specific	5	136	6.06e-34	cd02163	PPAT	cl00015	Phosphopantetheine adenylyltransferase
	superfamily	5	136	6.06e-34	cl00015	nt_trans superfamily	-	nucleotidyl transferase superfamily
	non-specific	3	136	1.16e-33	COG0669	CoaD	cl00015	Phosphopantetheine adenylyltransferase
	non-specific	5	136	6.94e-30	TIGR01510	coaD_prev_kdtB	cl00015	pantetheine-phosphate adenylyltransferase
	non-specific	3	136	2.01e-29	PRK00168	coaD	cl00015	phosphopantetheine adenylyltransferase
	non-specific	3	148	1.60e-27	PRK13964	coaD	cl00015	phosphopantetheine adenylyltransferase
	non-specific	7	137	1.78e-09	pfam01467	CTP_transf_like	cl00015	Cytidylyltransferase-like
	non-specific	5	136	1.17e-05	cd02039	cytidylyltransferase_like	cl00015	Cytidylyltransferase-like domain
M. pirum	non-specific	5	64	5.58e-05	TIGR00125	cyt_tran_rel	cl00015	cytidyltransferase-like domain
	non-specific	1	139	0.003566	COG1057	NadD	cl00015	Nicotinic acid mononucleotide adenylyltransferase
M. primatum	specific	1	140	4.60e-68	PRK13964	coaD	cl00015	phosphopantetheine adenylyltransferase
	superfamily	1	140	4.60e-68	cl00015	nt_trans superfamily	-	nucleotidyl transferase superfamily
	non-specific	1	139	7.16e-45	COG0669	CoaD	cl00015	Phosphopantetheine adenylyltransferase
	non-specific	3	133	4.10e-40	cd02163	PPAT	cl00015	Phosphopantetheine adenylyltransferase
	non-specific	3	133	1.77e-39	TIGR01510	coaD_prev_kdtB	cl00015	pantetheine-phosphate adenylyltransferase
	non-specific	1	133	6.48e-39	PRK00168	coaD	cl00015	phosphopantetheine adenylyltransferase
	specific	5	135	2.92e-19	pfam01467	CTP_transf_like	cl00015	Cytidylyltransferase-like
	non-specific	3	63	1.25e-11	TIGR00125	cyt_tran_rel	cl00015	cytidyltransferase-like domain
	non-specific	4	66	5.00e-10	cd02165	NMNAT	cl00015	Nicotinamide/nicotinate mononucleotide adenylyltransferase
	non-specific	1	66	2.27e-09	PRK07152	nadD	cl28367	putative nicotinate-nucleotide adenylyltransferase
	superfamily	1	66	2.27e-09	cl28367	nadD superfamily	-	putative nicotinate-nucleotide adenylyltransferase
	non-specific	3	133	3.18e-09	cd02039	cytidylyltransferase_like	cl00015	Cytidylyltransferase-like domain
	non-specific	5	133	2.80e-08	cd02167	NMNAT_NadR	cl00015	Nicotinamide/nicotinate mononucleotide adenylyltransferase
	non-specific	1	66	2.88e-08	COG1057	NadD	cl00015	Nicotinic acid mononucleotide adenylyltransferase
	non-specific	1	66	3.34e-07	PRK00071	nadD	cl00015	nicotinic acid mononucleotide adenylyltransferase
	non-specific	1	87	4.45e-07	COG0615	TagD	cl00015	Glycerol-3-phosphate cytidylyltransferase, cytidylyltransferase family
	non-specific	1	35	4.80e-07	COG1056	NadR	cl00015	Nicotinamide mononucleotide adenylyltransferase
	non-specific	1	36	5.86e-07	cd02170	cytidylyltransferase	cl00015	cytidylyltransferase
	non-specific	5	56	1.62e-06	TIGR00482	TIGR00482	cl00015	nicotinate (nicotinamide) nucleotide adenylyltransferase
	non-specific	3	39	1.08e-05	cd02156	nt_trans	cl00015	nucleotidyl transferase superfamily
	non-specific	1	36	5.76e-05	cd02171	G3P_Cytidylyltransferase	cl00015	glycerol-3-phosphate cytidylyltransferase
	non-specific	5	58	6.21e-05	TIGR01518	g3p_cytidyltrns	cl00015	glycerol-3-phosphate cytidylyltransferase
	non-specific	1	134	9.28e-05	PRK08099	PRK08099	cl28365	bifunctional DNA-binding transcriptional repressor/ NMN adenylyltransferase
	superfamily	1	134	9.28e-05	cl28365	PRK08099 superfamily	-	bifunctional DNA-binding transcriptional repressor/ NMN adenylyltransferase

Query	Hit type	ID region start	ID region end	E-Value	Accession	Short name	Superfamily	Definition
	non-specific	13	39	0.000104	pfam02569	Pantoate_ligase	cl00015	Pantoate-beta-alanine ligase
	non-specific	1	33	0.000108	PRK00777	PRK00777	cl00015	phosphopantetheine adenylyltransferase
	non-specific	1	133	0.000184	TIGR01526	nadR_NMN_Atrans	cl28365	nicotinamide-nucleotide adenylyltransferase
	non-specific	2	35	0.000231	PRK01153	PRK01153	cl00015	nicotinamide-nucleotide adenylyltransferase
	non-specific	3	42	0.00048	cd02173	ECT	cl00015	CTP:phosphoethanolamine cytidylyltransferase (ECT)
	non-specific	3	35	0.000531	cd02166	NMNAT_Archaea	cl00015	Nicotinamide/nicotinate mononucleotide adenylyltransferase
	non-specific	1	89	0.00074	COG1019	CAB4	cl00015	Phosphopantetheine adenylyltransferase
	non-specific	3	46	0.001026	TIGR01527	arch_NMN_Atrans	cl00015	nicotinamide-nucleotide adenylyltransferase
	non-specific	13	39	0.001088	cd00560	PanC	cl00015	Pantoate-beta-alanine ligase
	non-specific	4	35	0.001237	PRK05379	PRK05379	cl28366	bifunctional nicotinamide mononucleotide adenylyltransferase
M. primatum	superfamily	4	35	0.001237	cl28366	PRK05379 superfamily	-	bifunctional nicotinamide mononucleotide adenylyltransferase
	non-specific	13	39	0.001776	COG0414	PanC	cl00015	Panthothenate synthetase
	non-specific	13	39	0.00428	PRK00380	panC	cl00015	pantoate--beta-alanine ligase
	non-specific	7	60	0.005073	cd02164	PPAT_CoAS	cl00015	phosphopantetheine adenylyltransferase
M. pulmonis	specific	5	146	9.05e-69	PRK13964	coaD	cl00015	phosphopantetheine adenylyltransferase
	superfamily	5	146	9.05e-69	cl00015	nt_trans superfamily	-	nucleotidyl transferase superfamily
	specific	4	146	8.15e-57	COG0669	CoaD	cl00015	Phosphopantetheine adenylyltransferase
	non-specific	7	139	1.27e-39	cd02163	PPAT	cl00015	Phosphopantetheine adenylyltransferase
	non-specific	5	139	5.99e-38	PRK00168	coaD	cl00015	phosphopantetheine adenylyltransferase
	non-specific	7	139	7.37e-38	TIGR01510	coaD_prev_kdtB	cl00015	pantetheine-phosphate adenylyltransferase
	specific	9	139	1.04e-19	pfam01467	CTP_transf_like	cl00015	Cytidylyltransferase-like
	non-specific	2	59	4.15e-12	PRK00071	nadD	cl00015	nicotinic acid mononucleotide adenylyltransferase
	non-specific	7	67	2.35e-11	TIGR00125	cyt_tran_rel	cl00015	cytidyltransferase-like domain
	non-specific	3	59	1.26e-09	COG1057	NadD	cl00015	Nicotinic acid mononucleotide adenylyltransferase
	non-specific	7	59	2.23e-09	cd02165	NMNAT	cl00015	Nicotinamide/nicotinate mononucleotide adenylyltransferase
	non-specific	7	139	6.78e-09	cd02039	cytidylyltransferase_like	cl00015	Cytidylyltransferase-like domain
	non-specific	5	70	6.06e-07	PRK07152	nadD	cl28367	putative nicotinate-nucleotide adenylyltransferase
	superfamily	5	70	6.06e-07	cl28367	nadD superfamily	-	putative nicotinate-nucleotide adenylyltransferase
	non-specific	9	93	2.24e-06	TIGR00482	TIGR00482	cl00015	nicotinate (nicotinamide) nucleotide adenylyltransferase
	non-specific	1	70	4.89e-06	PRK05379	PRK05379	cl28366	bifunctional nicotinamide mononucleotide adenylyltransferase
	superfamily	1	70	4.89e-06	cl28366	PRK05379 superfamily	-	bifunctional nicotinamide mononucleotide adenylyltransferase
	non-specific	8	63	8.69e-06	cd02156	nt_trans	cl00015	nucleotidyl transferase superfamily
	non-specific	3	39	0.000122	COG1056	NadR	cl00015	Nicotinamide mononucleotide adenylyltransferase
	non-specific	7	139	0.000176	cd02064	FAD_synthetase_N	cl00015	FAD synthetase, N-terminal domain of the bifunctional enzyme
	non-specific	5	71	0.000571	PRK08887	PRK08887	cl00015	nicotinic acid mononucleotide adenylyltransferase
	non-specific	8	39	0.000958	PRK01153	PRK01153	cl00015	nicotinamide-nucleotide adenylyltransferase
	non-specific	4	46	0.001423	PTZ00308	PTZ00308	cl28626	ethanolamine-phosphate cytidylyltransferase
	superfamily	4	46	0.001423	cl28626	PLN02406 superfamily	-	ethanolamine-phosphate cytidylyltransferase
	non-specific	12	47	0.001912	cd02174	CCT	cl00015	CTP:phosphocholine cytidylyltransferase
	non-specific	7	146	0.00194	cd02167	NMNAT_NadR	cl00015	Nicotinamide/nicotinate mononucleotide adenylyltransferase
	non-specific	5	139	0.003724	PRK00777	PRK00777	cl00015	phosphopantetheine adenylyltransferase
	non-specific	8	39	0.004703	cd02166	NMNAT_Archaea	cl00015	Nicotinamide/nicotinate mononucleotide adenylyltransferase
	non-specific	7	29	0.004914	cd02168	NMNAT_Nudix	cl00015	Nicotinamide/nicotinate mononucleotide adenylyltransferase

Query	Hit type	ID region start	ID region end	E-Value	Accession	Short name	Superfamily	Definition
	non-specific	6	67	0.005299	PRK01170	PRK01170	cl00866	phosphopantetheine adenylyltransferase
	superfamily	6	67	0.005299	cl00866	NTPase_I-T superfamily	-	Protein of unknown function DUF84
M. pulmonis	non-specific	1	139	0.005711	COG1019	CAB4	cl00015	Phosphopantetheine adenylyltransferase
	non-specific	6	60	0.007006	cd02171	G3P_Cytidylyltransferase	cl00015	glycerol-3-phosphate cytidylyltransferase
M. putrefaciens	non-specific	3	141	1.92e-54	TIGR01510	coaD_prev_kdtB	cl00015	pantetheine-phosphate adenylyltransferase
	superfamily	3	141	1.92e-54	cl00015	nt_trans superfamily	-	nucleotidyl transferase superfamily
	non-specific	1	140	1.67e-53	PRK13964	coaD	cl00015	phosphopantetheine adenylyltransferase
	non-specific	1	141	1.78e-53	PRK00168	coaD	cl00015	phosphopantetheine adenylyltransferase
	non-specific	3	141	1.79e-50	cd02163	PPAT	cl00015	Phosphopantetheine adenylyltransferase
	non-specific	1	141	1.96e-50	COG0669	CoaD	cl00015	Phosphopantetheine adenylyltransferase
	non-specific	5	135	4.78e-15	pfam01467	CTP_transf_like	cl00015	Cytidylyltransferase-like
	non-specific	3	134	2.73e-12	cd02039	cytidylyltransferase_like	cl00015	Cytidylyltransferase-like domain
	non-specific	3	63	4.33e-12	TIGR00125	cyt_tran_rel	cl00015	cytidyltransferase-like domain
	non-specific	1	66	1.60e-09	COG1057	NadD	cl00015	Nicotinic acid mononucleotide adenylyltransferase
	non-specific	1	66	4.28e-08	PRK07152	nadD	cl28367	putative nicotinate-nucleotide adenylyltransferase
	superfamily	1	66	4.28e-08	cl28367	nadD superfamily	-	putative nicotinate-nucleotide adenylyltransferase
	non-specific	3	66	2.30e-07	cd02165	NMNAT	cl00015	Nicotinamide/nicotinate mononucleotide adenylyltransferase
	non-specific	1	35	2.44e-07	COG1056	NadR	cl00015	Nicotinamide mononucleotide adenylyltransferase
	non-specific	1	66	1.14e-06	PRK00071	nadD	cl00015	nicotinic acid mononucleotide adenylyltransferase
	non-specific	1	64	1.61e-05	TIGR01526	nadR_NMN_Atrans	cl28365	nicotinamide-nucleotide adenylyltransferase
	superfamily	1	64	1.61e-05	cl28365	PRK08099 superfamily	-	bifunctional DNA-binding transcriptional repressor/ NMN adenylyltransferase
	non-specific	8	134	1.66e-05	COG0615	TagD	cl00015	Glycerol-3-phosphate cytidylyltransferase, cytidylyltransferase family
	non-specific	4	66	2.10e-05	TIGR01527	arch_NMN_Atrans	cl00015	nicotinamide-nucleotide adenylyltransferase
	non-specific	3	39	4.29e-05	PRK05379	PRK05379	cl28366	bifunctional nicotinamide mononucleotide adenylyltransferase
	superfamily	3	39	4.29e-05	cl28366	PRK05379 superfamily	-	bifunctional nicotinamide mononucleotide adenylyltransferase
	non-specific	4	35	0.00012	cd02166	NMNAT_Archaea	cl00015	Nicotinamide/nicotinate mononucleotide adenylyltransferase
	non-specific	8	97	0.000166	cd02170	cytidylyltransferase	cl00015	cytidylyltransferase
	non-specific	1	135	0.000191	cd02171	G3P_Cytidylyltransferase	cl00015	glycerol-3-phosphate cytidylyltransferase
	non-specific	4	35	0.000215	PRK01153	PRK01153	cl00015	nicotinamide-nucleotide adenylyltransferase
	non-specific	3	66	0.000497	cd02167	NMNAT_NadR	cl00015	Nicotinamide/nicotinate mononucleotide adenylyltransferase
	non-specific	3	24	0.000885	cd02168	NMNAT_Nudix	cl00015	Nicotinamide/nicotinate mononucleotide adenylyltransferase
	non-specific	4	130	0.001521	cd02156	nt_trans	cl00015	nucleotidyl transferase superfamily
	non-specific	5	85	0.002241	TIGR00482	TIGR00482	cl00015	nicotinate (nicotinamide) nucleotide adenylyltransferase
	non-specific	1	33	0.002402	PRK00777	PRK00777	cl00015	phosphopantetheine adenylyltransferase
	non-specific	8	35	0.002999	cd02174	CCT	cl00015	CTP:phosphocholine cytidylyltransferase
M. simbae	non-specific	1	140	7.69e-56	PRK13964	coaD	cl00015	phosphopantetheine adenylyltransferase
	superfamily	1	140	7.69e-56	cl00015	nt_trans superfamily	-	nucleotidyl transferase superfamily
M. simbae	non-specific	1	134	2.68e-43	COG0669	CoaD	cl00015	Phosphopantetheine adenylyltransferase
	non-specific	3	133	6.47e-40	cd02163	PPAT	cl00015	Phosphopantetheine adenylyltransferase
	non-specific	1	133	1.02e-38	PRK00168	coaD	cl00015	phosphopantetheine adenylyltransferase
	non-specific	3	133	2.41e-35	TIGR01510	coaD_prev_kdtB	cl00015	pantetheine-phosphate adenylyltransferase

Query	Hit type	ID region start	ID region end	E-Value	Accession	Short name	Superfamily	Definition
	specific	5	135	3.01e-18	pfam01467	CTP_transf_like	cl00015	Cytidylyltransferase-like
	non-specific	5	133	1.67e-09	cd02167	NMNAT_NadR	cl00015	Nicotinamide/nicotinate mononucleotide adenylyltransferase
	non-specific	3	46	1.73e-09	TIGR00125	cyt_tran_rel	cl00015	cytidyltransferase-like domain
	non-specific	3	133	1.59e-07	cd02039	cytidylyltransferase_like	cl00015	Cytidylyltransferase-like domain
	non-specific	1	35	1.63e-07	COG1056	NadR	cl00015	Nicotinamide mononucleotide adenylyltransferase
	non-specific	1	66	2.64e-07	COG1057	NadD	cl00015	Nicotinic acid mononucleotide adenylyltransferase
	non-specific	1	66	2.74e-07	PRK07152	nadD	cl28367	putative nicotinate-nucleotide adenylyltransferase
	superfamily	1	66	2.74e-07	cl28367	nadD superfamily	-	putative nicotinate-nucleotide adenylyltransferase
	non-specific	4	41	5.16e-07	cd02165	NMNAT	cl00015	Nicotinamide/nicotinate mononucleotide adenylyltransferase
	non-specific	1	52	4.38e-06	PRK00071	nadD	cl00015	nicotinic acid mononucleotide adenylyltransferase
	non-specific	5	66	7.52e-06	TIGR00482	TIGR00482	cl00015	nicotinate (nicotinamide) nucleotide adenylyltransferase
	non-specific	1	133	1.05e-05	TIGR01526	nadR_NMN_Atrans	cl28365	nicotinamide-nucleotide adenylyltransferase
	superfamily	1	133	1.05e-05	cl28365	PRK08099 superfamily	-	bifunctional DNA-binding transcriptional repressor/ NMN adenylyltransferase
	non-specific	1	33	1.95e-05	PRK00777	PRK00777	cl00015	phosphopantetheine adenylyltransferase
	non-specific	4	68	2.47e-05	PRK05379	PRK05379	cl28366	bifunctional nicotinamide mononucleotide adenylyltransferase
	superfamily	4	68	2.47e-05	cl28366	PRK05379 superfamily	-	bifunctional nicotinamide mononucleotide adenylyltransferase
	non-specific	3	35	3.94e-05	cd02166	NMNAT_Archaea	cl00015	Nicotinamide/nicotinate mononucleotide adenylyltransferase
	non-specific	1	35	5.06e-05	PRK01153	PRK01153	cl00015	nicotinamide-nucleotide adenylyltransferase
	non-specific	3	56	7.86e-05	TIGR01527	arch_NMN_Atrans	cl00015	nicotinamide-nucleotide adenylyltransferase
	non-specific	8	134	0.000172	PRK08099	PRK08099	cl28365	bifunctional DNA-binding transcriptional repressor/ NMN adenylyltransferase
	non-specific	4	42	0.000416	cd02168	NMNAT_Nudix	cl00015	Nicotinamide/nicotinate mononucleotide adenylyltransferase
	non-specific	1	56	0.000768	COG1019	CAB4	cl00015	Phosphopantetheine adenylyltransferase
	non-specific	1	36	0.001184	cd02171	G3P_Cytidylyltransferase	cl00015	glycerol-3-phosphate cytidylyltransferase
	non-specific	8	27	0.002505	pfam06574	FAD_syn	cl00015	FAD synthetase
	non-specific	7	59	0.004095	cd02164	PPAT_CoAS	cl00015	phosphopantetheine adenylyltransferase
	non-specific	3	57	0.00418	cd02156	nt_trans	cl00015	nucleotidyl transferase superfamily
	non-specific	1	43	0.004248	COG0615	TagD	cl00015	Glycerol-3-phosphate cytidylyltransferase, cytidylyltransferase family
	non-specific	8	50	0.004811	PTZ00308	PTZ00308	cl28626	ethanolamine-phosphate cytidylyltransferase
	superfamily	8	50	0.004811	cl28626	PLN02406 superfamily	-	ethanolamine-phosphate cytidylyltransferase
M. sturni	non-specific	3	142	5.48e-59	PRK13964	coaD	cl00015	phosphopantetheine adenylyltransferase
	superfamily	3	142	5.48e-59	cl00015	nt_trans superfamily	-	nucleotidyl transferase superfamily
M. sturni	non-specific	4	144	8.41e-51	COG0669	CoaD	cl00015	Phosphopantetheine adenylyltransferase
	non-specific	5	144	4.45e-46	cd02163	PPAT	cl00015	Phosphopantetheine adenylyltransferase
	non-specific	4	144	2.23e-43	PRK00168	coaD	cl00015	phosphopantetheine adenylyltransferase
	non-specific	5	144	3.64e-42	TIGR01510	coaD_prev_kdtB	cl00015	pantetheine-phosphate adenylyltransferase
	specific	7	135	7.56e-27	pfam01467	CTP_transf_like	cl00015	Cytidylyltransferase-like
	non-specific	5	65	6.76e-14	TIGR00125	cyt_tran_rel	cl00015	cytidyltransferase-like domain
	non-specific	1	68	2.33e-12	PRK00071	nadD	cl00015	nicotinic acid mononucleotide adenylyltransferase
	non-specific	1	68	2.90e-11	COG1057	NadD	cl00015	Nicotinic acid mononucleotide adenylyltransferase
	non-specific	4	68	1.19e-10	PRK07152	nadD	cl28367	putative nicotinate-nucleotide adenylyltransferase

Query	Hit type	ID region start	ID region end	E-Value	Accession	Short name	Superfamily	Definition
	superfamily	4	68	1.19e-10	cl28367	nadD superfamily	-	putative nicotinate-nucleotide adenylyltransferase
	non-specific	5	135	3.63e-10	cd02039	cytidylyltransferase_like	cl00015	Cytidylyltransferase-like domain
	non-specific	5	68	4.80e-10	cd02165	NMNAT	cl00015	Nicotinamide/nicotinate mononucleotide adenylyltransferase
	non-specific	1	136	6.18e-08	PRK08099	PRK08099	cl28365	bifunctional DNA-binding transcriptional repressor/ NMN adenylyltransferase
	superfamily	1	136	6.18e-08	cl28365	PRK08099 superfamily	-	bifunctional DNA-binding transcriptional repressor/ NMN adenylyltransferase
	non-specific	7	64	2.74e-07	TIGR00482	TIGR00482	cl00015	nicotinate (nicotinamide) nucleotide adenylyltransferase
	non-specific	4	135	6.18e-07	TIGR01526	nadR_NMN_Atrans	cl28365	nicotinamide-nucleotide adenylyltransferase
	non-specific	1	37	6.44e-07	COG1056	NadR	cl00015	Nicotinamide mononucleotide adenylyltransferase
	non-specific	5	68	2.02e-06	cd02167	NMNAT_NadR	cl00015	Nicotinamide/nicotinate mononucleotide adenylyltransferase
	non-specific	6	92	2.35e-06	cd02156	nt_trans	cl00015	nucleotidyl transferase superfamily
	non-specific	10	72	3.65e-05	PRK00777	PRK00777	cl00015	phosphopantetheine adenylyltransferase
	non-specific	6	37	8.73e-05	PRK01153	PRK01153	cl00015	nicotinamide-nucleotide adenylyltransferase
	non-specific	6	37	0.000192	cd02166	NMNAT_Archaea	cl00015	Nicotinamide/nicotinate mononucleotide adenylyltransferase
	non-specific	7	37	0.000354	cd02173	ECT	cl00015	CTP:phosphoethanolamine cytidylyltransferase (ECT)
	non-specific	10	78	0.00064	COG1019	CAB4	cl00015	Phosphopantetheine adenylyltransferase
	non-specific	4	37	0.000661	PTZ00308	PTZ00308	cl28626	ethanolamine-phosphate cytidylyltransferase
	superfamily	4	37	0.000661	cl28626	PLN02406 superfamily	-	ethanolamine-phosphate cytidylyltransferase
	non-specific	4	43	0.000784	TIGR02199	rfaE_dom_II	cl00015	rfaE bifunctional protein, domain II
	non-specific	10	38	0.001375	cd02170	cytidylyltransferase	cl00015	cytidylyltransferase
	non-specific	10	42	0.003324	COG0615	TagD	cl00015	Glycerol-3-phosphate cytidylyltransferase, cytidylyltransferase family
	non-specific	1	36	0.006569	PRK05379	PRK05379	cl28366	bifunctional nicotinamide mononucleotide adenylyltransferase
	superfamily	1	36	0.006569	cl28366	PRK05379 superfamily	-	bifunctional nicotinamide mononucleotide adenylyltransferase
M. synoviae	specific	7	145	1.02e-70	PRK13964	coaD	cl00015	phosphopantetheine adenylyltransferase
	superfamily	7	145	1.02e-70	cl00015	nt_trans superfamily	-	nucleotidyl transferase superfamily
	non-specific	5	139	8.48e-46	COG0669	CoaD	cl00015	Phosphopantetheine adenylyltransferase
	non-specific	8	138	4.70e-43	cd02163	PPAT	cl00015	Phosphopantetheine adenylyltransferase
M. synoviae	non-specific	7	138	7.70e-39	PRK00168	coaD	cl00015	phosphopantetheine adenylyltransferase
	non-specific	8	138	2.29e-38	TIGR01510	coaD_prev_kdtB	cl00015	pantetheine-phosphate adenylyltransferase
	specific	10	138	1.75e-21	pfam01467	CTP_transf_like	cl00015	Cytidylyltransferase-like
	non-specific	8	40	3.49e-12	TIGR00125	cyt_tran_rel	cl00015	cytidyltransferase-like domain
	non-specific	8	138	2.88e-11	cd02039	cytidylyltransferase_like	cl00015	Cytidylyltransferase-like domain
	non-specific	7	71	5.01e-08	COG1057	NadD	cl00015	Nicotinic acid mononucleotide adenylyltransferase
	non-specific	8	44	6.67e-08	cd02156	nt_trans	cl00015	nucleotidyl transferase superfamily
	non-specific	10	138	7.22e-08	cd02167	NMNAT_NadR	cl00015	Nicotinamide/nicotinate mononucleotide adenylyltransferase
	non-specific	9	71	7.22e-08	cd02165	NMNAT	cl00015	Nicotinamide/nicotinate mononucleotide adenylyltransferase
	non-specific	3	46	1.03e-06	PRK00071	nadD	cl00015	nicotinic acid mononucleotide adenylyltransferase
	non-specific	13	41	1.37e-06	cd02170	cytidylyltransferase	cl00015	cytidylyltransferase
	non-specific	10	67	1.89e-06	TIGR00482	TIGR00482	cl00015	nicotinate (nicotinamide) nucleotide adenylyltransferase
	non-specific	13	90	2.20e-06	COG0615	TagD	cl00015	Glycerol-3-phosphate cytidylyltransferase, cytidylyltransferase family

Query	Hit type	ID region start	ID region end	E-Value	Accession	Short name	Superfamily	Definition
	non-specific	7	48	6.80e-06	PRK07152	nadD	cl28367	putative nicotinate-nucleotide adenylyltransferase
	superfamily	7	48	6.80e-06	cl28367	nadD superfamily	-	putative nicotinate-nucleotide adenylyltransferase
	non-specific	7	40	2.13e-05	COG1056	NadR	cl00015	Nicotinamide mononucleotide adenylyltransferase
	non-specific	13	38	0.000116	PRK00777	PRK00777	cl00015	phosphopantetheine adenylyltransferase
	non-specific	10	41	0.000464	TIGR01526	nadR_NMN_Atrans	cl28365	nicotinamide-nucleotide adenylyltransferase
	superfamily	10	41	0.000464	cl28365	PRK08099 superfamily	-	bifunctional DNA-binding transcriptional repressor/ NMN adenylyltransferase
	non-specific	13	46	0.000556	cd02174	CCT	cl00015	CTP:phosphocholine cytidylyltransferase
	non-specific	7	40	0.001029	PRK01153	PRK01153	cl00015	nicotinamide-nucleotide adenylyltransferase
	non-specific	8	40	0.001086	cd02166	NMNAT_Archaea	cl00015	Nicotinamide/nicotinate mononucleotide adenylyltransferase
	non-specific	12	46	0.001356	PTZ00308	PTZ00308	cl28626	ethanolamine-phosphate cytidylyltransferase
	superfamily	12	46	0.001356	cl28626	PLN02406 superfamily	-	ethanolamine-phosphate cytidylyltransferase
	non-specific	7	41	0.001661	cd02171	G3P_Cytidylyltransferase	cl00015	glycerol-3-phosphate cytidylyltransferase
	non-specific	8	46	0.002191	cd02173	ECT	cl00015	CTP:phosphoethanolamine cytidylyltransferase (ECT)
	non-specific	13	62	0.002334	TIGR01518	g3p_cytidyltrns	cl00015	glycerol-3-phosphate cytidylyltransferase
	non-specific	3	88	0.007463	COG1019	CAB4	cl00015	Phosphopantetheine adenylyltransferase
	non-specific	13	139	0.009413	PRK08099	PRK08099	cl28365	bifunctional DNA-binding transcriptional repressor/ NMN adenylyltransferase
M. testudinis	non-specific	4	138	6.38e-51	cd02163	PPAT	cl00015	Phosphopantetheine adenylyltransferase
	superfamily	4	138	6.38e-51	cl00015	nt_trans superfamily	-	nucleotidyl transferase superfamily
	non-specific	3	138	5.73e-50	PRK00168	coaD	cl00015	phosphopantetheine adenylyltransferase
	non-specific	1	138	6.30e-50	COG0669	CoaD	cl00015	Phosphopantetheine adenylyltransferase
	non-specific	4	138	4.10e-46	TIGR01510	coaD_prev_kdtB	cl00015	pantetheine-phosphate adenylyltransferase
	non-specific	3	145	6.67e-38	PRK13964	coaD	cl00015	phosphopantetheine adenylyltransferase
	specific	6	137	1.45e-21	pfam01467	CTP_transf_like	cl00015	Cytidylyltransferase-like
	non-specific	4	62	5.82e-13	TIGR00125	cyt_tran_rel	cl00015	cytidyltransferase-like domain
M. testudinis	non-specific	1	87	9.30e-13	PRK00071	nadD	cl00015	nicotinic acid mononucleotide adenylyltransferase
	non-specific	4	135	5.30e-11	cd02039	cytidylyltransferase_like	cl00015	Cytidylyltransferase-like domain
	non-specific	4	142	7.57e-11	cd02165	NMNAT	cl00015	Nicotinamide/nicotinate mononucleotide adenylyltransferase
	non-specific	1	136	3.00e-10	COG1057	NadD	cl00015	Nicotinic acid mononucleotide adenylyltransferase
	non-specific	5	36	9.11e-09	cd02166	NMNAT_Archaea	cl00015	Nicotinamide/nicotinate mononucleotide adenylyltransferase
	non-specific	5	36	2.08e-08	PRK01153	PRK01153	cl00015	nicotinamide-nucleotide adenylyltransferase
	non-specific	3	36	6.77e-08	COG1056	NadR	cl00015	Nicotinamide mononucleotide adenylyltransferase
	non-specific	1	142	1.49e-07	PRK08887	PRK08887	cl00015	nicotinic acid mononucleotide adenylyltransferase
	non-specific	6	103	8.88e-07	TIGR00482	TIGR00482	cl00015	nicotinate (nicotinamide) nucleotide adenylyltransferase
	non-specific	5	48	2.28e-06	cd02156	nt_trans	cl00015	nucleotidyl transferase superfamily
	non-specific	5	67	2.29e-05	TIGR01527	arch_NMN_Atrans	cl00015	nicotinamide-nucleotide adenylyltransferase
	non-specific	3	87	2.33e-05	PRK07152	nadD	cl28367	putative nicotinate-nucleotide adenylyltransferase
	superfamily	3	87	2.33e-05	cl28367	nadD superfamily	-	putative nicotinate-nucleotide adenylyltransferase
	non-specific	4	33	5.56e-05	PRK05379	PRK05379	cl28366	bifunctional nicotinamide mononucleotide adenylyltransferase
	superfamily	4	33	5.56e-05	cl28366	PRK05379 superfamily	-	bifunctional nicotinamide mononucleotide adenylyltransferase
	non-specific	2	36	8.10e-05	PTZ00308	PTZ00308	cl28626	ethanolamine-phosphate cytidylyltransferase
	superfamily	2	36	8.10e-05	cl28626	PLN02406 superfamily	-	ethanolamine-phosphate cytidylyltransferase

Query	Hit type	ID region start	ID region end	E-Value	Accession	Short name	Superfamily	Definition
	non-specific	9	42	8.99e-05	COG0615	TagD	cl00015	Glycerol-3-phosphate cytidylyltransferase, cytidylyltransferase family
	non-specific	9	56	9.03e-05	cd02170	cytidylyltransferase	cl00015	cytidylyltransferase
	non-specific	3	36	0.000179	cd02173	ECT	cl00015	CTP:phosphoethanolamine cytidylyltransferase (ECT)
	non-specific	4	36	0.000766	cd02168	NMNAT_Nudix	cl00015	Nicotinamide/nicotinate mononucleotide adenylyltransferase
	non-specific	3	37	0.001214	cd02171	G3P_Cytidylyltransferase	cl00015	glycerol-3-phosphate cytidylyltransferase
	non-specific	1	36	0.003957	PRK00777	PRK00777	cl00015	phosphopantetheine adenylyltransferase
	non-specific	3	139	2.57e-56	TIGR01510	coaD_prev_kdtB	cl00015	pantetheine-phosphate adenylyltransferase
	superfamily	3	139	2.57e-56	cl00015	nt_trans superfamily	-	nucleotidyl transferase superfamily
	non-specific	1	135	9.05e-55	PRK00168	coaD	cl00015	phosphopantetheine adenylyltransferase
	non-specific	1	140	1.17e-53	PRK13964	coaD	cl00015	phosphopantetheine adenylyltransferase
	non-specific	1	140	1.38e-52	COG0669	CoaD	cl00015	Phosphopantetheine adenylyltransferase
	non-specific	3	139	1.53e-52	cd02163	PPAT	cl00015	Phosphopantetheine adenylyltransferase
M. yeatsii	specific	5	135	3.84e-20	pfam01467	CTP_transf_like	cl00015	Cytidylyltransferase-like
	non-specific	3	63	1.68e-14	TIGR00125	cyt_tran_rel	cl00015	cytidylyltransferase-like domain
	non-specific	3	134	1.00e-13	cd02039	cytidylyltransferase_like	cl00015	Cytidylyltransferase-like domain
	non-specific	3	66	1.63e-09	cd02165	NMNAT	cl00015	Nicotinamide/nicotinate mononucleotide adenylyltransferase
	non-specific	1	35	5.73e-09	COG1056	NadR	cl00015	Nicotinamide mononucleotide adenylyltransferase
	non-specific	1	60	2.21e-08	COG1057	NadD	cl00015	Nicotinic acid mononucleotide adenylyltransferase
	non-specific	1	66	1.18e-07	PRK07152	nadD	cl28367	putative nicotinate-nucleotide adenylyltransferase
	superfamily	1	66	1.18e-07	cl28367	nadD superfamily	-	putative nicotinate-nucleotide adenylyltransferase
	non-specific	4	66	1.83e-07	TIGR01527	arch_NMN_Atrans	cl00015	nicotinamide-nucleotide adenylyltransferase
	non-specific	1	57	2.01e-07	cd02171	G3P_Cytidylyltransferase	cl00015	glycerol-3-phosphate cytidylyltransferase
	non-specific	4	35	3.01e-07	PRK01153	PRK01153	cl00015	nicotinamide-nucleotide adenylyltransferase
	non-specific	4	35	5.09e-07	cd02166	NMNAT_Archaea	cl00015	Nicotinamide/nicotinate mononucleotide adenylyltransferase
	non-specific	8	89	5.98e-07	cd02170	cytidylyltransferase	cl00015	cytidylyltransferase
	non-specific	1	69	7.93e-07	TIGR01526	nadR_NMN_Atrans	cl28365	nicotinamide-nucleotide adenylyltransferase
	superfamily	1	69	7.93e-07	cl28365	PRK08099 superfamily	-	bifunctional DNA-binding transcriptional repressor/ NMN adenylyltransferase
	non-specific	3	55	9.84e-07	PRK05379	PRK05379	cl28366	bifunctional nicotinamide mononucleotide adenylyltransferase
	superfamily	3	55	9.84e-07	cl28366	PRK05379 superfamily	-	bifunctional nicotinamide mononucleotide adenylyltransferase
M. yeatsii	non-specific	1	66	1.10e-06	PRK00071	nadD	cl00015	nicotinic acid mononucleotide adenylyltransferase
	non-specific	4	130	4.04e-06	cd02156	nt_trans	cl00015	nucleotidyl transferase superfamily
	non-specific	3	66	1.23e-05	cd02167	NMNAT_NadR	cl00015	Nicotinamide/nicotinate mononucleotide adenylyltransferase
	non-specific	8	138	4.20e-05	COG0615	TagD	cl00015	Glycerol-3-phosphate cytidylyltransferase, cytidylyltransferase family
	non-specific	3	76	5.40e-05	cd02168	NMNAT_Nudix	cl00015	Nicotinamide/nicotinate mononucleotide adenylyltransferase
	non-specific	1	33	0.000427	PRK00777	PRK00777	cl00015	phosphopantetheine adenylyltransferase
	non-specific	5	54	0.001052	TIGR00482	TIGR00482	cl00015	nicotinate (nicotinamide) nucleotide adenylyltransferase
	non-specific	8	54	0.001145	TIGR01518	g3p_cytidyltrns	cl00015	glycerol-3-phosphate cytidylyltransferase
	non-specific	8	134	0.001474	PRK07143	PRK07143	cl27514	hypothetical protein
	superfamily	8	134	0.001474	cl27514	Flavokinase superfamily	-	Riboflavin kinase
	non-specific	2	35	0.001999	PTZ00308	PTZ00308	cl28626	ethanolamine-phosphate cytidylyltransferase

Query	Hit type	ID region start	ID region end	E-Value	Accession	Short name	Superfamily	Definition
	superfamily	2	35	0.001999	cl28626	PLN02406 superfamily	-	ethanolamine-phosphate cytidylyltransferase
	non-specific	8	134	0.002964	COG0196	RibF	cl27514	FAD synthase
	non-specific	5	33	0.006077	cd02173	ECT	cl00015	CTP:phosphoethanolamine cytidylyltransferase (ECT)
	non-specific	12	73	0.00806	cd02169	Citrate_lyase_ligase	cl00015	Citrate lyase ligase

Supplementary Table 11 PPAT InterPro results

Mycoplasma species	Amino acid region	Database	Database ID	Database signature description	ID region start	ID region end	InterPro ID	ID type	Gene Ontology (GO) term
M. agalactiae	140	Gene3D	G3DSA:3.40.50.620		1	140	IPR014729	H	
		Hamap	MF_00151	Phosphopantetheine adenylyltransferase [coaD].	2	140	IPR001980	F	GO:0004595; GO:0015937
		PANTHER	PTHR21342:SF1		1	139	IPR001980	F	GO:0004595; GO:0015937
		PANTHER	PTHR21342		1	139			
		Pfam	PF01467	Cytidylyltransferase-like	5	134	IPR004821	D	GO:0003824; GO:0009058
		PRINTS	PR01020	Lipopolysaccharide core biosynthesis protein signature	2	20	IPR001980	F	GO:0004595; GO:0015937
		PRINTS	PR01020	Lipopolysaccharide core biosynthesis protein signature	49	73	IPR001980	F	GO:0004595; GO:0015937
		PRINTS	PR01020	Lipopolysaccharide core biosynthesis protein signature	20	41	IPR001980	F	GO:0004595; GO:0015937
		PRINTS	PR01020	Lipopolysaccharide core biosynthesis protein signature	113	135	IPR001980	F	GO:0004595; GO:0015937
		PRINTS	PR01020	Lipopolysaccharide core biosynthesis protein signature	86	102	IPR001980	F	GO:0004595; GO:0015937
		SUPERFAMILY	SSF52374		2	139			
		TIGRFAM	TIGR01510	coaD_prev_kdtB: pantetheine-phosphate adenylyltransferase	4	134	IPR001980	F	GO:0004595; GO:0015937
		TIGRFAM	TIGR00125	cyt_tran_rel: cytidyltransferase-like domain	4	58	IPR004821	D	GO:0003824; GO:0009058
M. alligatoris	145	Gene3D	G3DSA:3.40.50.620		1	144	IPR014729	H	
		Hamap	MF_00151	Phosphopantetheine adenylyltransferase [coaD].	5	145	IPR001980	F	GO:0004595; GO:0015937
		PANTHER	PTHR21342:SF1		5	140	IPR001980	F	GO:0004595; GO:0015937
		PANTHER	PTHR21342		5	140			
		Pfam	PF01467	Cytidylyltransferase-like	8	139	IPR004821	D	GO:0003824; GO:0009058

Mycoplasma species	Amino acid region	Database	Database ID	Database signature description	ID region start	ID region end	InterPro ID	ID type	Gene Ontology (GO) term
		PRINTS	PR01020	Lipopolysaccharide core biosynthesis protein signature	118	140	IPR001980	F	GO:0004595; GO:0015937
		PRINTS	PR01020	Lipopolysaccharide core biosynthesis protein signature	5	23	IPR001980	F	GO:0004595; GO:0015937
		PRINTS	PR01020	Lipopolysaccharide core biosynthesis protein signature	91	107	IPR001980	F	GO:0004595; GO:0015937
		PRINTS	PR01020	Lipopolysaccharide core biosynthesis protein signature	23	44	IPR001980	F	GO:0004595; GO:0015937
		SUPERFAMILY	SSF52374		5	141			
M. alligatoris	145	TIGRFAM	TIGR01510	coaD_prev_kdtB: pantetheine-phosphate adenylyltransferase	7	139	IPR001980	F	GO:0004595; GO:0015937
		TIGRFAM	TIGR00125	cyt_tran_rel: cytidyltransferase-like domain	7	63	IPR004821	D	GO:0003824; GO:0009058
M. alvi	151	Gene3D	G3DSA:3.40.50.620		1	149	IPR014729	H	
		Hamap	MF_00151	Phosphopantetheine adenylyltransferase [coaD].	4	151	IPR001980	F	GO:0004595; GO:0015937
		PANTHER	PTHR21342		4	140			
		PANTHER	PTHR21342:SF1		4	140	IPR001980	F	GO:0004595; GO:0015937
		Pfam	PF01467	Cytidylyltransferase-like	7	137	IPR004821	D	GO:0003824; GO:0009058
		PRINTS	PR01020	Lipopolysaccharide core biosynthesis protein signature	22	43	IPR001980	F	GO:0004595; GO:0015937
		PRINTS	PR01020	Lipopolysaccharide core biosynthesis protein signature	116	138	IPR001980	F	GO:0004595; GO:0015937
		PRINTS	PR01020	Lipopolysaccharide core biosynthesis protein signature	89	105	IPR001980	F	GO:0004595; GO:0015937
		PRINTS	PR01020	Lipopolysaccharide core biosynthesis protein signature	4	22	IPR001980	F	GO:0004595; GO:0015937
		SUPERFAMILY	SSF52374		4	143			
		TIGRFAM	TIGR00125	cyt_tran_rel: cytidyltransferase-like domain	5	39	IPR004821	D	GO:0003824; GO:0009058
		TIGRFAM	TIGR01510	coaD_prev_kdtB: pantetheine-phosphate adenylyltransferase	5	140	IPR001980	F	GO:0004595; GO:0015937
M. anatis	143	Gene3D	G3DSA:3.40.50.620		1	140	IPR014729	H	
		Hamap	MF_00151	Phosphopantetheine adenylyltransferase [coaD].	4	143	IPR001980	F	GO:0004595; GO:0015937
		PANTHER	PTHR21342:SF1		4	135	IPR001980	F	GO:0004595; GO:0015937
		PANTHER	PTHR21342		4	135			
		Pfam	PF01467	Cytidylyltransferase-like	7	135	IPR004821	D	GO:0003824; GO:0009058
		PRINTS	PR01020	Lipopolysaccharide core biosynthesis protein signature	115	137	IPR001980	F	GO:0004595; GO:0015937

Mycoplasma species	Amino acid region	Database	Database ID	Database signature description	ID region start	ID region end	InterPro ID	ID type	Gene Ontology (GO) term
		PRINTS	PR01020	Lipopolysaccharide core biosynthesis protein signature	22	43	IPR001980	F	GO:0004595; GO:0015937
		PRINTS	PR01020	Lipopolysaccharide core biosynthesis protein signature	88	104	IPR001980	F	GO:0004595; GO:0015937
		PRINTS	PR01020	Lipopolysaccharide core biosynthesis protein signature	51	75	IPR001980	F	GO:0004595; GO:0015937
		PRINTS	PR01020	Lipopolysaccharide core biosynthesis protein signature	4	22	IPR001980	F	GO:0004595; GO:0015937
		SUPERFAMILY	SSF52374		4	138			
M. anatis	143	TIGRFAM	TIGR01510	coaD_prev_kdtB: pantetheine-phosphate adenylyltransferase	6	136	IPR001980	F	GO:0004595; GO:0015937
		TIGRFAM	TIGR00125	cyt_tran_rel: cytidyltransferase-like domain	6	46	IPR004821	D	GO:0003824; GO:0009058
M. arginini	143	Gene3D	G3DSA:3.40.50.620		1	142	IPR014729	H	
		Hamap	MF_00151	Phosphopantetheine adenylyltransferase [coaD].	2	143	IPR001980	F	GO:0004595; GO:0015937
		PANTHER	PTHR21342:SF1		1	138	IPR001980	F	GO:0004595; GO:0015937
		PANTHER	PTHR21342		1	138			
		Pfam	PF01467	Cytidylyltransferase-like	5	134	IPR004821	D	GO:0003824; GO:0009058
		PRINTS	PR01020	Lipopolysaccharide core biosynthesis protein signature	86	102	IPR001980	F	GO:0004595; GO:0015937
		PRINTS	PR01020	Lipopolysaccharide core biosynthesis protein signature	2	20	IPR001980	F	GO:0004595; GO:0015937
		PRINTS	PR01020	Lipopolysaccharide core biosynthesis protein signature	113	135	IPR001980	F	GO:0004595; GO:0015937
		PRINTS	PR01020	Lipopolysaccharide core biosynthesis protein signature	20	41	IPR001980	F	GO:0004595; GO:0015937
		SUPERFAMILY	SSF52374		2	140			
		TIGRFAM	TIGR01510	coaD_prev_kdtB: pantetheine-phosphate adenylyltransferase	4	134	IPR001980	F	GO:0004595; GO:0015937
		TIGRFAM	TIGR00125	cyt_tran_rel: cytidyltransferase-like domain	4	55	IPR004821	D	GO:0003824; GO:0009058
M. bovigenitalium	140	Gene3D	G3DSA:3.40.50.620		1	139	IPR014729	H	
		PANTHER	PTHR21342:SF1		1	136	IPR001980	F	GO:0004595; GO:0015937
		PANTHER	PTHR21342		1	136			
		Pfam	PF01467	Cytidylyltransferase-like	5	134	IPR004821	D	GO:0003824; GO:0009058
		PRINTS	PR01020	Lipopolysaccharide core biosynthesis protein signature	113	135	IPR001980	F	GO:0004595; GO:0015937
		PRINTS	PR01020	Lipopolysaccharide core biosynthesis protein signature	2	20	IPR001980	F	GO:0004595; GO:0015937

Mycoplasma species	Amino acid region	Database	Database ID	Database signature description	ID region start	ID region end	InterPro ID	ID type	Gene Ontology (GO) term
		PRINTS	PR01020	Lipopolysaccharide core biosynthesis protein signature	86	102	IPR001980	F	GO:0004595; GO:0015937
		PRINTS	PR01020	Lipopolysaccharide core biosynthesis protein signature	49	73	IPR001980	F	GO:0004595; GO:0015937
		PRINTS	PR01020	Lipopolysaccharide core biosynthesis protein signature	20	41	IPR001980	F	GO:0004595; GO:0015937
		SUPERFAMILY	SSF52374		2	136			
		TIGRFAM	TIGR01510	coaD_prev_kdtB: pantetheine-phosphate adenylyltransferase	4	134	IPR001980	F	GO:0004595; GO:0015937
M. bovigenitalium	140	TIGRFAM	TIGR00125	cyt_tran_rel: cytidyltransferase-like domain	4	59	IPR004821	D	GO:0003824; GO:0009058
M. bovis	140	Gene3D	G3DSA:3.40.50.620		1	140	IPR014729	H	
		Hamap	MF_00151	Phosphopantetheine adenylyltransferase [coaD].	2	140	IPR001980	F	GO:0004595; GO:0015937
		PANTHER	PTHR21342		1	139			
		PANTHER	PTHR21342:SF1		1	139	IPR001980	F	GO:0004595; GO:0015937
		Pfam	PF01467	Cytidylyltransferase-like	5	134	IPR004821	D	GO:0003824; GO:0009058
		PRINTS	PR01020	Lipopolysaccharide core biosynthesis protein signature	49	73	IPR001980	F	GO:0004595; GO:0015937
		PRINTS	PR01020	Lipopolysaccharide core biosynthesis protein signature	2	20	IPR001980	F	GO:0004595; GO:0015937
		PRINTS	PR01020	Lipopolysaccharide core biosynthesis protein signature	20	41	IPR001980	F	GO:0004595; GO:0015937
		PRINTS	PR01020	Lipopolysaccharide core biosynthesis protein signature	113	135	IPR001980	F	GO:0004595; GO:0015937
		PRINTS	PR01020	Lipopolysaccharide core biosynthesis protein signature	86	102	IPR001980	F	GO:0004595; GO:0015937
		SUPERFAMILY	SSF52374		2	139			
		TIGRFAM	TIGR00125	cyt_tran_rel: cytidyltransferase-like domain	4	55	IPR004821	D	GO:0003824; GO:0009058
		TIGRFAM	TIGR01510	coaD_prev_kdtB: pantetheine-phosphate adenylyltransferase	4	134	IPR001980	F	GO:0004595; GO:0015937
M. buteonis	148	Coils	Coil		70	90			
		Gene3D	G3DSA:3.40.50.620		3	147	IPR014729	H	
		Hamap	MF_00151	Phosphopantetheine adenylyltransferase [coaD].	6	148	IPR001980	F	GO:0004595; GO:0015937
		PANTHER	PTHR21342:SF1		6	140	IPR001980	F	GO:0004595; GO:0015937
		PANTHER	PTHR21342		6	140			
		Pfam	PF01467	Cytidylyltransferase-like	9	140	IPR004821	D	GO:0003824; GO:0009058

Mycoplasma species	Amino acid region	Database	Database ID	Database signature description	ID region start	ID region end	InterPro ID	ID type	Gene Ontology (GO) term
		PRINTS	PR01020	Lipopolysaccharide core biosynthesis protein signature	24	45	IPR001980	F	GO:0004595; GO:0015937
		PRINTS	PR01020	Lipopolysaccharide core biosynthesis protein signature	6	24	IPR001980	F	GO:0004595; GO:0015937
		PRINTS	PR01020	Lipopolysaccharide core biosynthesis protein signature	92	108	IPR001980	F	GO:0004595; GO:0015937
		PRINTS	PR01020	Lipopolysaccharide core biosynthesis protein signature	119	141	IPR001980	F	GO:0004595; GO:0015937
		SUPERFAMILY	SSF52374		6	140			
M. buteonis	148	TIGRFAM	TIGR01510	coaD_prev_kdtB: pantetheine-phosphate adenylyltransferase	7	144	IPR001980	F	GO:0004595; GO:0015937
		TIGRFAM	TIGR00125	cyt_tran_rel: cytidylyltransferase-like domain	7	62	IPR004821	D	GO:0003824; GO:0009058
M. californicum	143	Gene3D	G3DSA:3.40.50.620		1	142	IPR014729	H	
		PANTHER	PTHR21342:SF1		1	138	IPR001980	F	GO:0004595; GO:0015937
		PANTHER	PTHR21342		1	138			
		Pfam	PF01467	Cytidylyltransferase-like	5	134	IPR004821	D	GO:0003824; GO:0009058
		PRINTS	PR01020	Lipopolysaccharide core biosynthesis protein signature	2	20	IPR001980	F	GO:0004595; GO:0015937
		PRINTS	PR01020	Lipopolysaccharide core biosynthesis protein signature	86	102	IPR001980	F	GO:0004595; GO:0015937
		PRINTS	PR01020	Lipopolysaccharide core biosynthesis protein signature	49	73	IPR001980	F	GO:0004595; GO:0015937
		PRINTS	PR01020	Lipopolysaccharide core biosynthesis protein signature	113	135	IPR001980	F	GO:0004595; GO:0015937
		PRINTS	PR01020	Lipopolysaccharide core biosynthesis protein signature	20	41	IPR001980	F	GO:0004595; GO:0015937
		SUPERFAMILY	SSF52374		2	140			
		TIGRFAM	TIGR00125	cyt_tran_rel: cytidylyltransferase-like domain	4	57	IPR004821	D	GO:0003824; GO:0009058
		TIGRFAM	TIGR01510	coaD_prev_kdtB: pantetheine-phosphate adenylyltransferase	4	134	IPR001980	F	GO:0004595; GO:0015937
M. canis	143	Coils	Coil		46	66			
		Gene3D	G3DSA:3.40.50.620		1	142	IPR014729	H	
		PANTHER	PTHR21342		5	139			
		PANTHER	PTHR21342:SF1		5	139	IPR001980	F	GO:0004595; GO:0015937
		Pfam	PF01467	Cytidylyltransferase-like	8	137	IPR004821	D	GO:0003824; GO:0009058
		PRINTS	PR01020	Lipopolysaccharide core biosynthesis protein signature	89	105	IPR001980	F	GO:0004595; GO:0015937

Mycoplasma species	Amino acid region	Database	Database ID	Database signature description	ID region start	ID region end	InterPro ID	ID type	Gene Ontology (GO) term
		PRINTS	PR01020	Lipopolysaccharide core biosynthesis protein signature	116	138	IPR001980	F	GO:0004595; GO:0015937
		PRINTS	PR01020	Lipopolysaccharide core biosynthesis protein signature	5	23	IPR001980	F	GO:0004595; GO:0015937
		PRINTS	PR01020	Lipopolysaccharide core biosynthesis protein signature	23	44	IPR001980	F	GO:0004595; GO:0015937
		SUPERFAMILY	SSF52374		5	138			
		TIGRFAM	TIGR01510	coaD_prev_kdtB: pantetheine-phosphate adenylyltransferase	7	139	IPR001980	F	GO:0004595; GO:0015937
M. canis	143	TIGRFAM	TIGR00125	cyt_tran_rel: cytidylyltransferase-like domain	7	47	IPR004821	D	GO:0003824; GO:0009058
M. capricolum	140	Gene3D	G3DSA:3.40.50.620		1	137	IPR014729	H	
		Hamap	MF_00151	Phosphopantetheine adenylyltransferase [coaD].	2	140	IPR001980	F	GO:0004595; GO:0015937
		PANTHER	PTHR21342:SF1		1	138	IPR001980	F	GO:0004595; GO:0015937
		PANTHER	PTHR21342		1	138			
		Pfam	PF01467	Cytidylyltransferase-like	5	134	IPR004821	D	GO:0003824; GO:0009058
		PRINTS	PR01020	Lipopolysaccharide core biosynthesis protein signature	113	135	IPR001980	F	GO:0004595; GO:0015937
		PRINTS	PR01020	Lipopolysaccharide core biosynthesis protein signature	2	20	IPR001980	F	GO:0004595; GO:0015937
		PRINTS	PR01020	Lipopolysaccharide core biosynthesis protein signature	49	73	IPR001980	F	GO:0004595; GO:0015937
		PRINTS	PR01020	Lipopolysaccharide core biosynthesis protein signature	86	102	IPR001980	F	GO:0004595; GO:0015937
		PRINTS	PR01020	Lipopolysaccharide core biosynthesis protein signature	20	41	IPR001980	F	GO:0004595; GO:0015937
		SUPERFAMILY	SSF52374		2	138			
		TIGRFAM	TIGR01510	coaD_prev_kdtB: pantetheine-phosphate adenylyltransferase	3	139	IPR001980	F	GO:0004595; GO:0015937
		TIGRFAM	TIGR00125	cyt_tran_rel: cytidylyltransferase-like domain	3	61	IPR004821	D	GO:0003824; GO:0009058
M. collis	147	Gene3D	G3DSA:3.40.50.620		2	143	IPR014729	H	
		Hamap	MF_00151	Phosphopantetheine adenylyltransferase [coaD].	8	147	IPR001980	F	GO:0004595; GO:0015937
		PANTHER	PTHR21342		7	139			
		PANTHER	PTHR21342:SF1		7	139	IPR001980	F	GO:0004595; GO:0015937
		Pfam	PF01467	Cytidylyltransferase-like	11	136	IPR004821	D	GO:0003824; GO:0009058
		PRINTS	PR01020	Lipopolysaccharide core biosynthesis protein signature	55	79	IPR001980	F	GO:0004595; GO:0015937

Mycoplasma species	Amino acid region	Database	Database ID	Database signature description	ID region start	ID region end	InterPro ID	ID type	Gene Ontology (GO) term
		PRINTS	PR01020	Lipopolysaccharide core biosynthesis protein signature	8	26	IPR001980	F	GO:0004595; GO:0015937
		PRINTS	PR01020	Lipopolysaccharide core biosynthesis protein signature	26	47	IPR001980	F	GO:0004595; GO:0015937
		PRINTS	PR01020	Lipopolysaccharide core biosynthesis protein signature	92	108	IPR001980	F	GO:0004595; GO:0015937
		PRINTS	PR01020	Lipopolysaccharide core biosynthesis protein signature	119	141	IPR001980	F	GO:0004595; GO:0015937
		SUPERFAMILY	SSF52374		8	141			
M. collis	147	TIGRFAM	TIGR01510	coaD_prev_kdtB: pantetheine-phosphate adenylyltransferase	10	139	IPR001980	F	GO:0004595; GO:0015937
		TIGRFAM	TIGR00125	cyt_tran_rel: cytidyltransferase-like domain	10	67	IPR004821	D	GO:0003824; GO:0009058
M. columbinum	142	Gene3D	G3DSA:3.40.50.620		1	141	IPR014729	H	
		Hamap	MF_00151	Phosphopantetheine adenylyltransferase [coaD].	3	142	IPR001980	F	GO:0004595; GO:0015937
		PANTHER	PTHR21342		1	138			
		PANTHER	PTHR21342:SF1		1	138	IPR001980	F	GO:0004595; GO:0015937
		Pfam	PF01467	Cytidylyltransferase-like	6	136	IPR004821	D	GO:0003824; GO:0009058
		PRINTS	PR01020	Lipopolysaccharide core biosynthesis protein signature	115	137	IPR001980	F	GO:0004595; GO:0015937
		PRINTS	PR01020	Lipopolysaccharide core biosynthesis protein signature	21	42	IPR001980	F	GO:0004595; GO:0015937
		PRINTS	PR01020	Lipopolysaccharide core biosynthesis protein signature	3	21	IPR001980	F	GO:0004595; GO:0015937
		PRINTS	PR01020	Lipopolysaccharide core biosynthesis protein signature	88	104	IPR001980	F	GO:0004595; GO:0015937
		SUPERFAMILY	SSF52374		3	141			
		TIGRFAM	TIGR00125	cyt_tran_rel: cytidyltransferase-like domain	4	57	IPR004821	D	GO:0003824; GO:0009058
		TIGRFAM	TIGR01510	coaD_prev_kdtB: pantetheine-phosphate adenylyltransferase	4	136	IPR001980	F	GO:0004595; GO:0015937
M. columborale	142	Gene3D	G3DSA:3.40.50.620		1	139	IPR014729	H	
		Hamap	MF_00151	Phosphopantetheine adenylyltransferase [coaD].	4	141	IPR001980	F	GO:0004595; GO:0015937
		PANTHER	PTHR21342		4	136			
		PANTHER	PTHR21342:SF1		4	136	IPR001980	F	GO:0004595; GO:0015937
		Pfam	PF01467	Cytidylyltransferase-like	7	136	IPR004821	D	GO:0003824; GO:0009058
		PRINTS	PR01020	Lipopolysaccharide core biosynthesis protein signature	115	137	IPR001980	F	GO:0004595; GO:0015937

Mycoplasma species	Amino acid region	Database	Database ID	Database signature description	ID region start	ID region end	InterPro ID	ID type	Gene Ontology (GO) term
		PRINTS	PR01020	Lipopolysaccharide core biosynthesis protein signature	4	22	IPR001980	F	GO:0004595; GO:0015937
		PRINTS	PR01020	Lipopolysaccharide core biosynthesis protein signature	88	104	IPR001980	F	GO:0004595; GO:0015937
		PRINTS	PR01020	Lipopolysaccharide core biosynthesis protein signature	51	75	IPR001980	F	GO:0004595; GO:0015937
		PRINTS	PR01020	Lipopolysaccharide core biosynthesis protein signature	22	43	IPR001980	F	GO:0004595; GO:0015937
		SUPERFAMILY	SSF52374		5	136			
M. columborale	142	TIGRFAM	TIGR00125	cyt_tran_rel: cytidylyltransferase-like domain	5	46	IPR004821	D	GO:0003824; GO:0009058
		TIGRFAM	TIGR01510	coaD_prev_kdtB: pantetheine-phosphate adenylyltransferase	5	136	IPR001980	F	GO:0004595; GO:0015937
M. conjunctivae	148	Gene3D	G3DSA:3.40.50.620		2	145	IPR014729	H	
		Hamap	MF_00151	Phosphopantetheine adenylyltransferase [coaD].	11	147	IPR001980	F	GO:0004595; GO:0015937
		PANTHER	PTHR21342:SF1		11	142	IPR001980	F	GO:0004595; GO:0015937
		PANTHER	PTHR21342		11	142			
		Pfam	PF01467	Cytidylyltransferase-like	14	142	IPR004821	D	GO:0003824; GO:0009058
		PRINTS	PR01020	Lipopolysaccharide core biosynthesis protein signature	95	111	IPR001980	F	GO:0004595; GO:0015937
		PRINTS	PR01020	Lipopolysaccharide core biosynthesis protein signature	122	144	IPR001980	F	GO:0004595; GO:0015937
		PRINTS	PR01020	Lipopolysaccharide core biosynthesis protein signature	29	50	IPR001980	F	GO:0004595; GO:0015937
		PRINTS	PR01020	Lipopolysaccharide core biosynthesis protein signature	11	29	IPR001980	F	GO:0004595; GO:0015937
		PRINTS	PR01020	Lipopolysaccharide core biosynthesis protein signature	58	82	IPR001980	F	GO:0004595; GO:0015937
		SUPERFAMILY	SSF52374		11	144			
		TIGRFAM	TIGR00125	cyt_tran_rel: cytidylyltransferase-like domain	13	64	IPR004821	D	GO:0003824; GO:0009058
		TIGRFAM	TIGR01510	coaD_prev_kdtB: pantetheine-phosphate adenylyltransferase	13	142	IPR001980	F	GO:0004595; GO:0015937
M. cricetuli	142	Gene3D	G3DSA:3.40.50.620		1	141	IPR014729	H	
		Hamap	MF_00151	Phosphopantetheine adenylyltransferase [coaD].	4	142	IPR001980	F	GO:0004595; GO:0015937
		PANTHER	PTHR21342:SF1		4	138	IPR001980	F	GO:0004595; GO:0015937
		PANTHER	PTHR21342		4	138			
		Pfam	PF01467	Cytidylyltransferase-like	7	136	IPR004821	D	GO:0003824; GO:0009058

Mycoplasma species	Amino acid region	Database	Database ID	Database signature description	ID region start	ID region end	InterPro ID	ID type	Gene Ontology (GO) term
		PRINTS	PR01020	Lipopolysaccharide core biosynthesis protein signature	88	104	IPR001980	F	GO:0004595; GO:0015937
		PRINTS	PR01020	Lipopolysaccharide core biosynthesis protein signature	115	137	IPR001980	F	GO:0004595; GO:0015937
		PRINTS	PR01020	Lipopolysaccharide core biosynthesis protein signature	51	75	IPR001980	F	GO:0004595; GO:0015937
		PRINTS	PR01020	Lipopolysaccharide core biosynthesis protein signature	22	43	IPR001980	F	GO:0004595; GO:0015937
M. cricetuli	142	PRINTS	PR01020	Lipopolysaccharide core biosynthesis protein signature	4	22	IPR001980	F	GO:0004595; GO:0015937
		SUPERFAMILY	SSF52374		4	141			
		TIGRFAM	TIGR00125	cyt_tran_rel: cytidyltransferase-like domain	5	59	IPR004821	D	GO:0003824; GO:0009058
		TIGRFAM	TIGR01510	coaD_prev_kdtB: pantetheine-phosphate adenylyltransferase	5	136	IPR001980	F	GO:0004595; GO:0015937
M. crocodyli	146	Gene3D	G3DSA:3.40.50.620		3	146	IPR014729	H	
		Hamap	MF_00151	Phosphopantetheine adenylyltransferase [coaD].	6	146	IPR001980	F	GO:0004595; GO:0015937
		PANTHER	PTHR21342		6	142			
		PANTHER	PTHR21342:SF1		6	142	IPR001980	F	GO:0004595; GO:0015937
		Pfam	PF01467	Cytidylyltransferase-like	9	139	IPR004821	D	GO:0003824; GO:0009058
		PRINTS	PR01020	Lipopolysaccharide core biosynthesis protein signature	92	108	IPR001980	F	GO:0004595; GO:0015937
		PRINTS	PR01020	Lipopolysaccharide core biosynthesis protein signature	119	141	IPR001980	F	GO:0004595; GO:0015937
		PRINTS	PR01020	Lipopolysaccharide core biosynthesis protein signature	24	45	IPR001980	F	GO:0004595; GO:0015937
		PRINTS	PR01020	Lipopolysaccharide core biosynthesis protein signature	6	24	IPR001980	F	GO:0004595; GO:0015937
		SUPERFAMILY	SSF52374		6	145			
		TIGRFAM	TIGR01510	coaD_prev_kdtB: pantetheine-phosphate adenylyltransferase	8	140	IPR001980	F	GO:0004595; GO:0015937
		TIGRFAM	TIGR00125	cyt_tran_rel: cytidyltransferase-like domain	8	62	IPR004821	D	GO:0003824; GO:0009058
M. felifaucium	146	Coils	Coil		46	66			
		Gene3D	G3DSA:3.40.50.620		1	142	IPR014729	H	
		Hamap	MF_00151	Phosphopantetheine adenylyltransferase [coaD].	4	146	IPR001980	F	GO:0004595; GO:0015937
		PANTHER	PTHR21342:SF1		4	141	IPR001980	F	GO:0004595; GO:0015937
		PANTHER	PTHR21342		4	141			

Mycoplasma species	Amino acid region	Database	Database ID	Database signature description	ID region start	ID region end	InterPro ID	ID type	Gene Ontology (GO) term
		Pfam	PF01467	Cytidylyltransferase-like	7	136	IPR004821	D	GO:0003824; GO:0009058
		PRINTS	PR01020	Lipopolysaccharide core biosynthesis protein signature	51	75	IPR001980	F	GO:0004595; GO:0015937
		PRINTS	PR01020	Lipopolysaccharide core biosynthesis protein signature	22	43	IPR001980	F	GO:0004595; GO:0015937
		PRINTS	PR01020	Lipopolysaccharide core biosynthesis protein signature	4	22	IPR001980	F	GO:0004595; GO:0015937
M. felifaucium	146	PRINTS	PR01020	Lipopolysaccharide core biosynthesis protein signature	115	137	IPR001980	F	GO:0004595; GO:0015937
		PRINTS	PR01020	Lipopolysaccharide core biosynthesis protein signature	88	104	IPR001980	F	GO:0004595; GO:0015937
		SUPERFAMILY	SSF52374		4	140			
		TIGRFAM	TIGR00125	cyt_tran_rel: cytidyltransferase-like domain	5	62	IPR004821	D	GO:0003824; GO:0009058
		TIGRFAM	TIGR01510	coaD_prev_kdtB: pantetheine-phosphate adenylyltransferase	5	141	IPR001980	F	GO:0004595; GO:0015937
M. felis	137	Gene3D	G3DSA:3.40.50.620		1	135	IPR014729	H	
		PANTHER	PTHR21342		1	133			
		PANTHER	PTHR21342:SF1		1	133	IPR001980	F	GO:0004595; GO:0015937
		Pfam	PF01467	Cytidylyltransferase-like	6	133	IPR004821	D	GO:0003824; GO:0009058
		PRINTS	PR01020	Lipopolysaccharide core biosynthesis protein signature	21	42	IPR001980	F	GO:0004595; GO:0015937
		PRINTS	PR01020	Lipopolysaccharide core biosynthesis protein signature	3	21	IPR001980	F	GO:0004595; GO:0015937
		PRINTS	PR01020	Lipopolysaccharide core biosynthesis protein signature	86	102	IPR001980	F	GO:0004595; GO:0015937
		PRINTS	PR01020	Lipopolysaccharide core biosynthesis protein signature	113	135	IPR001980	F	GO:0004595; GO:0015937
		SUPERFAMILY	SSF52374		5	134			
		TIGRFAM	TIGR00125	cyt_tran_rel: cytidyltransferase-like domain	5	49	IPR004821	D	GO:0003824; GO:0009058
		TIGRFAM	TIGR01510	coaD_prev_kdtB: pantetheine-phosphate adenylyltransferase	5	133	IPR001980	F	GO:0004595; GO:0015937
M. fermentans	142	Coils	Coil		45	65			
		Gene3D	G3DSA:3.40.50.620		2	142	IPR014729	H	
		Hamap	MF_00151	Phosphopantetheine adenylyltransferase [coaD].	3	142	IPR001980	F	GO:0004595; GO:0015937
		PANTHER	PTHR21342:SF1		1	141	IPR001980	F	GO:0004595; GO:0015937
		PANTHER	PTHR21342		1	141			

Mycoplasma species	Amino acid region	Database	Database ID	Database signature description	ID region start	ID region end	InterPro ID	ID type	Gene Ontology (GO) term
		Pfam	PF01467	Cytidylyltransferase-like	6	135	IPR004821	D	GO:0003824; GO:0009058
		PRINTS	PR01020	Lipopolysaccharide core biosynthesis protein signature	50	74	IPR001980	F	GO:0004595; GO:0015937
		PRINTS	PR01020	Lipopolysaccharide core biosynthesis protein signature	87	103	IPR001980	F	GO:0004595; GO:0015937
		PRINTS	PR01020	Lipopolysaccharide core biosynthesis protein signature	21	42	IPR001980	F	GO:0004595; GO:0015937
M. fermentans	142	PRINTS	PR01020	Lipopolysaccharide core biosynthesis protein signature	3	21	IPR001980	F	GO:0004595; GO:0015937
		PRINTS	PR01020	Lipopolysaccharide core biosynthesis protein signature	114	136	IPR001980	F	GO:0004595; GO:0015937
		SUPERFAMILY	SSF52374		3	140			
		TIGRFAM	TIGR00125	cyt_tran_rel: cytidylyltransferase-like domain	5	58	IPR004821	D	GO:0003824; GO:0009058
		TIGRFAM	TIGR01510	coaD_prev_kdtB: pantetheine-phosphate adenylyltransferase	5	137	IPR001980	F	GO:0004595; GO:0015937
M. gallinaceum	145	Gene3D	G3DSA:3.40.50.620		1	145	IPR014729	H	
		Hamap	MF_00151	Phosphopantetheine adenylyltransferase [coaD].	5	145	IPR001980	F	GO:0004595; GO:0015937
		PANTHER	PTHR21342:SF1		5	144	IPR001980	F	GO:0004595; GO:0015937
		PANTHER	PTHR21342		5	144			
		Pfam	PF01467	Cytidylyltransferase-like	8	137	IPR004821	D	GO:0003824; GO:0009058
		PRINTS	PR01020	Lipopolysaccharide core biosynthesis protein signature	52	76	IPR001980	F	GO:0004595; GO:0015937
		PRINTS	PR01020	Lipopolysaccharide core biosynthesis protein signature	5	23	IPR001980	F	GO:0004595; GO:0015937
		PRINTS	PR01020	Lipopolysaccharide core biosynthesis protein signature	116	138	IPR001980	F	GO:0004595; GO:0015937
		PRINTS	PR01020	Lipopolysaccharide core biosynthesis protein signature	23	44	IPR001980	F	GO:0004595; GO:0015937
		PRINTS	PR01020	Lipopolysaccharide core biosynthesis protein signature	89	105	IPR001980	F	GO:0004595; GO:0015937
		SUPERFAMILY	SSF52374		5	144			
		TIGRFAM	TIGR00125	cyt_tran_rel: cytidylyltransferase-like domain	7	58	IPR004821	D	GO:0003824; GO:0009058
		TIGRFAM	TIGR01510	coaD_prev_kdtB: pantetheine-phosphate adenylyltransferase	7	145	IPR001980	F	GO:0004595; GO:0015937
M. gallinarum	141	Coils	Coil		45	65			
		Gene3D	G3DSA:3.40.50.620		1	139	IPR014729	H	
		Hamap	MF_00151	Phosphopantetheine adenylyltransferase [coaD].	3	141	IPR001980	F	GO:0004595; GO:0015937

Mycoplasma species	Amino acid region	Database	Database ID	Database signature description	ID region start	ID region end	InterPro ID	ID type	Gene Ontology (GO) term
		PANTHER	PTHR21342		1	137			
		PANTHER	PTHR21342:SF1		1	137	IPR001980	F	GO:0004595; GO:0015937
		Pfam	PF01467	Cytidylyltransferase-like	6	133	IPR004821	D	GO:0003824; GO:0009058
		PRINTS	PR01020	Lipopolysaccharide core biosynthesis protein signature	87	103	IPR001980	F	GO:0004595; GO:0015937
M. gallinarum	141	PRINTS	PR01020	Lipopolysaccharide core biosynthesis protein signature	50	74	IPR001980	F	GO:0004595; GO:0015937
		PRINTS	PR01020	Lipopolysaccharide core biosynthesis protein signature	21	42	IPR001980	F	GO:0004595; GO:0015937
		PRINTS	PR01020	Lipopolysaccharide core biosynthesis protein signature	3	21	IPR001980	F	GO:0004595; GO:0015937
		PRINTS	PR01020	Lipopolysaccharide core biosynthesis protein signature	114	136	IPR001980	F	GO:0004595; GO:0015937
		SUPERFAMILY	SSF52374		3	137			
		TIGRFAM	TIGR01510	coaD_prev_kdtB: pantetheine-phosphate adenylyltransferase	4	140	IPR001980	F	GO:0004595; GO:0015937
		TIGRFAM	TIGR00125	cyt_tran_rel: cytidylyltransferase-like domain	4	60	IPR004821	D	GO:0003824; GO:0009058
M. iners	147	Gene3D	G3DSA:3.40.50.620		1	145	IPR014729	H	
		Hamap	MF_00151	Phosphopantetheine adenylyltransferase [coaD].	3	147	IPR001980	F	GO:0004595; GO:0015937
		PANTHER	PTHR21342:SF1		1	136	IPR001980	F	GO:0004595; GO:0015937
		PANTHER	PTHR21342		1	136			
		Pfam	PF01467	Cytidylyltransferase-like	6	132	IPR004821	D	GO:0003824; GO:0009058
		PRINTS	PR01020	Lipopolysaccharide core biosynthesis protein signature	3	21	IPR001980	F	GO:0004595; GO:0015937
		PRINTS	PR01020	Lipopolysaccharide core biosynthesis protein signature	114	136	IPR001980	F	GO:0004595; GO:0015937
		PRINTS	PR01020	Lipopolysaccharide core biosynthesis protein signature	50	74	IPR001980	F	GO:0004595; GO:0015937
		PRINTS	PR01020	Lipopolysaccharide core biosynthesis protein signature	87	103	IPR001980	F	GO:0004595; GO:0015937
		PRINTS	PR01020	Lipopolysaccharide core biosynthesis protein signature	21	42	IPR001980	F	GO:0004595; GO:0015937
		SUPERFAMILY	SSF52374		3	138			
		TIGRFAM	TIGR01510	coaD_prev_kdtB: pantetheine-phosphate adenylyltransferase	5	140	IPR001980	F	GO:0004595; GO:0015937
		TIGRFAM	TIGR00125	cyt_tran_rel: cytidylyltransferase-like domain	4	46	IPR004821	D	GO:0003824; GO:0009058
M. iowae	144	Gene3D	G3DSA:3.40.50.620		1	144	IPR014729	H	

Mycoplasma species	Amino acid region	Database	Database ID	Database signature description	ID region start	ID region end	InterPro ID	ID type	Gene Ontology (GO) term
		PANTHER	PTHR21342		6	136			
		PANTHER	PTHR21342:SF1		6	136	IPR001980	F	GO:0004595; GO:0015937
		Pfam	PF01467	Cytidylyltransferase-like	9	137	IPR004821	D	GO:0003824; GO:0009058
		PRINTS	PR01020	Lipopolysaccharide core biosynthesis protein signature	6	24	IPR001980	F	GO:0004595; GO:0015937
M. iowae	144	PRINTS	PR01020	Lipopolysaccharide core biosynthesis protein signature	117	139	IPR001980	F	GO:0004595; GO:0015937
		PRINTS	PR01020	Lipopolysaccharide core biosynthesis protein signature	24	45	IPR001980	F	GO:0004595; GO:0015937
		PRINTS	PR01020	Lipopolysaccharide core biosynthesis protein signature	90	106	IPR001980	F	GO:0004595; GO:0015937
		SUPERFAMILY	SSF52374		8	142			
		TIGRFAM	TIGR00125	cyt_tran_rel: cytidyltransferase-like domain	8	63	IPR004821	D	GO:0003824; GO:0009058
		TIGRFAM	TIGR01510	coaD_prev_kdtB: pantetheine-phosphate adenylyltransferase	8	136	IPR001980	F	GO:0004595; GO:0015937
M. leachii	140	Gene3D	G3DSA:3.40.50.620		1	137	IPR014729	H	
		Hamap	MF_00151	Phosphopantetheine adenylyltransferase [coaD].	2	140	IPR001980	F	GO:0004595; GO:0015937
		PANTHER	PTHR21342:SF1		1	138	IPR001980	F	GO:0004595; GO:0015937
		PANTHER	PTHR21342		1	138			
		Pfam	PF01467	Cytidylyltransferase-like	5	134	IPR004821	D	GO:0003824; GO:0009058
		PRINTS	PR01020	Lipopolysaccharide core biosynthesis protein signature	86	102	IPR001980	F	GO:0004595; GO:0015937
		PRINTS	PR01020	Lipopolysaccharide core biosynthesis protein signature	113	135	IPR001980	F	GO:0004595; GO:0015937
		PRINTS	PR01020	Lipopolysaccharide core biosynthesis protein signature	49	73	IPR001980	F	GO:0004595; GO:0015937
		PRINTS	PR01020	Lipopolysaccharide core biosynthesis protein signature	20	41	IPR001980	F	GO:0004595; GO:0015937
		PRINTS	PR01020	Lipopolysaccharide core biosynthesis protein signature	2	20	IPR001980	F	GO:0004595; GO:0015937
		SUPERFAMILY	SSF52374		2	138			
		TIGRFAM	TIGR00125	cyt_tran_rel: cytidyltransferase-like domain	4	62	IPR004821	D	GO:0003824; GO:0009058
		TIGRFAM	TIGR01510	coaD_prev_kdtB: pantetheine-phosphate adenylyltransferase	3	139	IPR001980	F	GO:0004595; GO:0015937
M. leonicaptivi	137	Gene3D	G3DSA:3.40.50.620		1	136	IPR014729	H	
		PANTHER	PTHR21342:SF1		4	135	IPR001980	F	GO:0004595; GO:0015937

Mycoplasma species	Amino acid region	Database	Database ID	Database signature description	ID region start	ID region end	InterPro ID	ID type	Gene Ontology (GO) term
		PANTHER	PTHR21342		4	135			
		Pfam	PF01467	Cytidylyltransferase-like	8	135	IPR004821	D	GO:0003824; GO:0009058
		PRINTS	PR01020	Lipopolysaccharide core biosynthesis protein signature	22	43	IPR001980	F	GO:0004595; GO:0015937
		PRINTS	PR01020	Lipopolysaccharide core biosynthesis protein signature	87	103	IPR001980	F	GO:0004595; GO:0015937
M. leonicaptivi	137	PRINTS	PR01020	Lipopolysaccharide core biosynthesis protein signature	4	22	IPR001980	F	GO:0004595; GO:0015937
		PRINTS	PR01020	Lipopolysaccharide core biosynthesis protein signature	114	136	IPR001980	F	GO:0004595; GO:0015937
		ProDom	PD016147	Ligase Lyase Citrate Pro-3S-Lyase Synthetase Transferase Cytidyltransferase-Related:Citrate:Acetate:SH-Citrate Citc Plasmid	5	67			
		SUPERFAMILY	SSF52374		5	135			
		TIGRFAM	TIGR00125	cyt_tran_rel: cytidyltransferase-like domain	5	46	IPR004821	D	GO:0003824; GO:0009058
		TIGRFAM	TIGR01510	coaD_prev_kdtB: pantetheine-phosphate adenylyltransferase	6	135	IPR001980	F	GO:0004595; GO:0015937
M. lipofaciens	140	Coils	Coil		44	64			
		Gene3D	G3DSA:3.40.50.620		1	140	IPR014729	H	
		Hamap	MF_00151	Phosphopantetheine adenylyltransferase [coaD].	2	140	IPR001980	F	GO:0004595; GO:0015937
		PANTHER	PTHR21342:SF1		1	139	IPR001980	F	GO:0004595; GO:0015937
		PANTHER	PTHR21342		1	139			
		Pfam	PF01467	Cytidylyltransferase-like	5	134	IPR004821	D	GO:0003824; GO:0009058
		PRINTS	PR01020	Lipopolysaccharide core biosynthesis protein signature	2	20	IPR001980	F	GO:0004595; GO:0015937
		PRINTS	PR01020	Lipopolysaccharide core biosynthesis protein signature	113	135	IPR001980	F	GO:0004595; GO:0015937
		PRINTS	PR01020	Lipopolysaccharide core biosynthesis protein signature	49	73	IPR001980	F	GO:0004595; GO:0015937
		PRINTS	PR01020	Lipopolysaccharide core biosynthesis protein signature	86	102	IPR001980	F	GO:0004595; GO:0015937
		PRINTS	PR01020	Lipopolysaccharide core biosynthesis protein signature	20	41	IPR001980	F	GO:0004595; GO:0015937
		SUPERFAMILY	SSF52374		2	139			
		TIGRFAM	TIGR00125	cyt_tran_rel: cytidyltransferase-like domain	4	60	IPR004821	D	GO:0003824; GO:0009058
		TIGRFAM	TIGR01510	coaD_prev_kdtB: pantetheine-phosphate adenylyltransferase	4	134	IPR001980	F	GO:0004595; GO:0015937
M. mobile	145	Coils	Coil		44	64			

Mycoplasma species	Amino acid region	Database	Database ID	Database signature description	ID region start	ID region end	InterPro ID	ID type	Gene Ontology (GO) term
		Gene3D	G3DSA:3.40.50.620		2	141	IPR014729	H	
		Pfam	PF01467	Cytidylyltransferase-like	12	72	IPR004821	D	GO:0003824; GO:0009058
		PRINTS	PR01020	Lipopolysaccharide core biosynthesis protein signature	9	27	IPR001980	F	GO:0004595; GO:0015937
		PRINTS	PR01020	Lipopolysaccharide core biosynthesis protein signature	27	48	IPR001980	F	GO:0004595; GO:0015937
M. mobile	145	PRINTS	PR01020	Lipopolysaccharide core biosynthesis protein signature	56	80	IPR001980	F	GO:0004595; GO:0015937
		SUPERFAMILY	SSF52374		9	126			
		TIGRFAM	TIGR00125	cyt_tran_rel: cytidyltransferase-like domain	11	61	IPR004821	D	GO:0003824; GO:0009058
M. molare	143	Gene3D	G3DSA:3.40.50.620		1	138	IPR014729	H	
		Hamap	MF_00151	Phosphopantetheine adenylyltransferase [coaD].	2	143	IPR001980	F	GO:0004595; GO:0015937
		PANTHER	PTHR21342:SF1		1	134	IPR001980	F	GO:0004595; GO:0015937
		PANTHER	PTHR21342		1	134			
		Pfam	PF01467	Cytidylyltransferase-like	5	133	IPR004821	D	GO:0003824; GO:0009058
		PRINTS	PR01020	Lipopolysaccharide core biosynthesis protein signature	113	135	IPR001980	F	GO:0004595; GO:0015937
		PRINTS	PR01020	Lipopolysaccharide core biosynthesis protein signature	49	73	IPR001980	F	GO:0004595; GO:0015937
		PRINTS	PR01020	Lipopolysaccharide core biosynthesis protein signature	2	20	IPR001980	F	GO:0004595; GO:0015937
		PRINTS	PR01020	Lipopolysaccharide core biosynthesis protein signature	86	102	IPR001980	F	GO:0004595; GO:0015937
		PRINTS	PR01020	Lipopolysaccharide core biosynthesis protein signature	20	41	IPR001980	F	GO:0004595; GO:0015937
		ProDom	PD016147	Ligase Lyase Citrate Pro-3S-Lyase Synthetase Transferase Cytidyltransferase-Related:Citrate:Acetate:SH-Citrate Citc Plasmid	11	75			
		SUPERFAMILY	SSF52374		4	138			
		TIGRFAM	TIGR00125	cyt_tran_rel: cytidyltransferase-like domain	4	56	IPR004821	D	GO:0003824; GO:0009058
		TIGRFAM	TIGR01510	coaD_prev_kdtB: pantetheine-phosphate adenylyltransferase	4	134	IPR001980	F	GO:0004595; GO:0015937
M. mycoides subsp. *capri*	140	Gene3D	G3DSA:3.40.50.620		1	137	IPR014729	H	
		Hamap	MF_00151	Phosphopantetheine adenylyltransferase [coaD].	2	140	IPR001980	F	GO:0004595; GO:0015937
		PANTHER	PTHR21342:SF1		1	138	IPR001980	F	GO:0004595; GO:0015937
		PANTHER	PTHR21342		1	138			

Mycoplasma species	Amino acid region	Database	Database ID	Database signature description	ID region start	ID region end	InterPro ID	ID type	Gene Ontology (GO) term
		Pfam	PF01467	Cytidylyltransferase-like	5	134	IPR004821	D	GO:0003824; GO:0009058
		PRINTS	PR01020	Lipopolysaccharide core biosynthesis protein signature	113	135	IPR001980	F	GO:0004595; GO:0015937
		PRINTS	PR01020	Lipopolysaccharide core biosynthesis protein signature	49	73	IPR001980	F	GO:0004595; GO:0015937
M. mycoides subsp. *capri*	140	PRINTS	PR01020	Lipopolysaccharide core biosynthesis protein signature	2	20	IPR001980	F	GO:0004595; GO:0015937
		PRINTS	PR01020	Lipopolysaccharide core biosynthesis protein signature	86	102	IPR001980	F	GO:0004595; GO:0015937
		PRINTS	PR01020	Lipopolysaccharide core biosynthesis protein signature	20	41	IPR001980	F	GO:0004595; GO:0015937
		SUPERFAMILY	SSF52374		2	138			
		TIGRFAM	TIGR01510	coaD_prev_kdtB: pantetheine-phosphate adenylyltransferase	3	139	IPR001980	F	GO:0004595; GO:0015937
		TIGRFAM	TIGR00125	cyt_tran_rel: cytidyltransferase-like domain	3	61	IPR004821	D	GO:0003824; GO:0009058
M. mycoides subsp. *mycoides*	140	Gene3D	G3DSA:3.40.50.620		1	137	IPR014729	H	
		Hamap	MF_00151	Phosphopantetheine adenylyltransferase [coaD].	2	140	IPR001980	F	GO:0004595; GO:0015937
		PANTHER	PTHR21342:SF1		1	138	IPR001980	F	GO:0004595; GO:0015937
		PANTHER	PTHR21342		1	138			
		Pfam	PF01467	Cytidylyltransferase-like	5	134	IPR004821	D	GO:0003824; GO:0009058
		PRINTS	PR01020	Lipopolysaccharide core biosynthesis protein signature	49	73	IPR001980	F	GO:0004595; GO:0015937
		PRINTS	PR01020	Lipopolysaccharide core biosynthesis protein signature	113	135	IPR001980	F	GO:0004595; GO:0015937
		PRINTS	PR01020	Lipopolysaccharide core biosynthesis protein signature	2	20	IPR001980	F	GO:0004595; GO:0015937
		PRINTS	PR01020	Lipopolysaccharide core biosynthesis protein signature	20	41	IPR001980	F	GO:0004595; GO:0015937
		PRINTS	PR01020	Lipopolysaccharide core biosynthesis protein signature	86	102	IPR001980	F	GO:0004595; GO:0015937
		SUPERFAMILY	SSF52374		2	138			
		TIGRFAM	TIGR00125	cyt_tran_rel: cytidyltransferase-like domain	4	62	IPR004821	D	GO:0003824; GO:0009058
		TIGRFAM	TIGR01510	coaD_prev_kdtB: pantetheine-phosphate adenylyltransferase	3	139	IPR001980	F	GO:0004595; GO:0015937
M. opalescens	145	Gene3D	G3DSA:3.40.50.620		1	143	IPR014729	H	
		Hamap	MF_00151	Phosphopantetheine adenylyltransferase [coaD].	2	145	IPR001980	F	GO:0004595; GO:0015937

Mycoplasma species	Amino acid region	Database	Database ID	Database signature description	ID region start	ID region end	InterPro ID	ID type	Gene Ontology (GO) term
		PANTHER	PTHR21342:SF1		1	139	IPR001980	F	GO:0004595; GO:0015937
		PANTHER	PTHR21342		1	139			
		Pfam	PF01467	Cytidylyltransferase-like	5	134	IPR004821	D	GO:0003824; GO:0009058
		PRINTS	PR01020	Lipopolysaccharide core biosynthesis protein signature	2	20	IPR001980	F	GO:0004595; GO:0015937
M. opalescens	145	PRINTS	PR01020	Lipopolysaccharide core biosynthesis protein signature	87	103	IPR001980	F	GO:0004595; GO:0015937
		PRINTS	PR01020	Lipopolysaccharide core biosynthesis protein signature	20	41	IPR001980	F	GO:0004595; GO:0015937
		PRINTS	PR01020	Lipopolysaccharide core biosynthesis protein signature	114	136	IPR001980	F	GO:0004595; GO:0015937
		SUPERFAMILY	SSF52374		2	140			
		TIGRFAM	TIGR00125	cyt_tran_rel: cytidylyltransferase-like domain	4	43	IPR004821	D	GO:0003824; GO:0009058
		TIGRFAM	TIGR01510	coaD_prev_kdtB: pantetheine-phosphate adenylyltransferase	4	135	IPR001980	F	GO:0004595; GO:0015937
M. penetrans	150	Coils	Coil		49	69			
		Gene3D	G3DSA:3.40.50.620		2	149	IPR014729	H	
		Hamap	MF_00151	Phosphopantetheine adenylyltransferase [coaD].	8	149	IPR001980	F	GO:0004595; GO:0015937
		PANTHER	PTHR21342		8	136			
		PANTHER	PTHR21342:SF1		8	136	IPR001980	F	GO:0004595; GO:0015937
		Pfam	PF01467	Cytidylyltransferase-like	11	140	IPR004821	D	GO:0003824; GO:0009058
		PRINTS	PR01020	Lipopolysaccharide core biosynthesis protein signature	8	26	IPR001980	F	GO:0004595; GO:0015937
		PRINTS	PR01020	Lipopolysaccharide core biosynthesis protein signature	119	141	IPR001980	F	GO:0004595; GO:0015937
		PRINTS	PR01020	Lipopolysaccharide core biosynthesis protein signature	92	108	IPR001980	F	GO:0004595; GO:0015937
		PRINTS	PR01020	Lipopolysaccharide core biosynthesis protein signature	26	47	IPR001980	F	GO:0004595; GO:0015937
		SUPERFAMILY	SSF52374		8	147			
		TIGRFAM	TIGR01510	coaD_prev_kdtB: pantetheine-phosphate adenylyltransferase	10	147	IPR001980	F	GO:0004595; GO:0015937
		TIGRFAM	TIGR00125	cyt_tran_rel: cytidylyltransferase-like domain	11	65	IPR004821	D	GO:0003824; GO:0009058
M. pirum	151	Gene3D	G3DSA:3.40.50.620		1	149	IPR014729	H	
		Hamap	MF_00151	Phosphopantetheine adenylyltransferase [coaD].	4	151	IPR001980	F	GO:0004595; GO:0015937

Mycoplasma species	Amino acid region	Database	Database ID	Database signature description	ID region start	ID region end	InterPro ID	ID type	Gene Ontology (GO) term
		PANTHER	PTHR21342:SF1		4	141	IPR001980	F	GO:0004595; GO:0015937
		PANTHER	PTHR21342		4	141			
		Pfam	PF01467	Cytidylyltransferase-like	7	137	IPR004821	D	GO:0003824; GO:0009058
		PRINTS	PR01020	Lipopolysaccharide core biosynthesis protein signature	4	22	IPR001980	F	GO:0004595; GO:0015937
M. pirum	151	PRINTS	PR01020	Lipopolysaccharide core biosynthesis protein signature	22	43	IPR001980	F	GO:0004595; GO:0015937
		PRINTS	PR01020	Lipopolysaccharide core biosynthesis protein signature	89	105	IPR001980	F	GO:0004595; GO:0015937
		PRINTS	PR01020	Lipopolysaccharide core biosynthesis protein signature	116	138	IPR001980	F	GO:0004595; GO:0015937
		SUPERFAMILY	SSF52374		4	143			
		TIGRFAM	TIGR01510	coaD_prev_kdtB: pantetheine-phosphate adenylyltransferase	5	140	IPR001980	F	GO:0004595; GO:0015937
		TIGRFAM	TIGR00125	cyt_tran_rel: cytidyltransferase-like domain	5	62	IPR004821	D	GO:0003824; GO:0009058
M. primatum	140	Gene3D	G3DSA:3.40.50.620		1	140	IPR014729	H	
		Hamap	MF_00151	Phosphopantetheine adenylyltransferase [coaD].	2	140	IPR001980	F	GO:0004595; GO:0015937
		PANTHER	PTHR21342:SF1		1	139	IPR001980	F	GO:0004595; GO:0015937
		PANTHER	PTHR21342		1	139			
		Pfam	PF01467	Cytidylyltransferase-like	5	134	IPR004821	D	GO:0003824; GO:0009058
		PRINTS	PR01020	Lipopolysaccharide core biosynthesis protein signature	113	135	IPR001980	F	GO:0004595; GO:0015937
		PRINTS	PR01020	Lipopolysaccharide core biosynthesis protein signature	49	73	IPR001980	F	GO:0004595; GO:0015937
		PRINTS	PR01020	Lipopolysaccharide core biosynthesis protein signature	86	102	IPR001980	F	GO:0004595; GO:0015937
		PRINTS	PR01020	Lipopolysaccharide core biosynthesis protein signature	2	20	IPR001980	F	GO:0004595; GO:0015937
		PRINTS	PR01020	Lipopolysaccharide core biosynthesis protein signature	20	41	IPR001980	F	GO:0004595; GO:0015937
		SUPERFAMILY	SSF52374		2	139			
		TIGRFAM	TIGR00125	cyt_tran_rel: cytidyltransferase-like domain	4	59	IPR004821	D	GO:0003824; GO:0009058
		TIGRFAM	TIGR01510	coaD_prev_kdtB: pantetheine-phosphate adenylyltransferase	4	134	IPR001980	F	GO:0004595; GO:0015937
M. pulmonis	149	Gene3D	G3DSA:3.40.50.620		2	143	IPR014729	H	
		Hamap	MF_00151	Phosphopantetheine adenylyltransferase [coaD].	6	148	IPR001980	F	GO:0004595; GO:0015937

Mycoplasma species	Amino acid region	Database	Database ID	Database signature description	ID region start	ID region end	InterPro ID	ID type	Gene Ontology (GO) term
		PANTHER	PTHR21342		6	140			
		PANTHER	PTHR21342:SF1		6	140	IPR001980	F	GO:0004595; GO:0015937
		Pfam	PF01467	Cytidylyltransferase-like	9	140	IPR004821	D	GO:0003824; GO:0009058
		PRINTS	PR01020	Lipopolysaccharide core biosynthesis protein signature	119	141	IPR001980	F	GO:0004595; GO:0015937
M. pulmonis	149	PRINTS	PR01020	Lipopolysaccharide core biosynthesis protein signature	24	45	IPR001980	F	GO:0004595; GO:0015937
		PRINTS	PR01020	Lipopolysaccharide core biosynthesis protein signature	92	108	IPR001980	F	GO:0004595; GO:0015937
		PRINTS	PR01020	Lipopolysaccharide core biosynthesis protein signature	6	24	IPR001980	F	GO:0004595; GO:0015937
		ProDom	PD016147	Ligase Lyase Citrate Pro-3S-Lyase Synthetase Transferase Cytidyltransferase-Related:Citrate:Acetate:SH-Citrate Citc Plasmid	4	103			
		SUPERFAMILY	SSF52374		6	140			
		TIGRFAM	TIGR01510	coaD_prev_kdtB: pantetheine-phosphate adenylyltransferase	7	140	IPR001980	F	GO:0004595; GO:0015937
		TIGRFAM	TIGR00125	cyt_tran_rel: cytidyltransferase-like domain	7	64	IPR004821	D	GO:0003824; GO:0009058
M. putrefaciens	141	Gene3D	G3DSA:3.40.50.620		1	137	IPR014729	H	
		Hamap	MF_00151	Phosphopantetheine adenylyltransferase [coaD].	2	141	IPR001980	F	GO:0004595; GO:0015937
		PANTHER	PTHR21342		1	139			
		PANTHER	PTHR21342:SF1		1	139	IPR001980	F	GO:0004595; GO:0015937
		Pfam	PF01467	Cytidylyltransferase-like	5	134	IPR004821	D	GO:0003824; GO:0009058
		PRINTS	PR01020	Lipopolysaccharide core biosynthesis protein signature	86	102	IPR001980	F	GO:0004595; GO:0015937
		PRINTS	PR01020	Lipopolysaccharide core biosynthesis protein signature	113	135	IPR001980	F	GO:0004595; GO:0015937
		PRINTS	PR01020	Lipopolysaccharide core biosynthesis protein signature	20	41	IPR001980	F	GO:0004595; GO:0015937
		PRINTS	PR01020	Lipopolysaccharide core biosynthesis protein signature	2	20	IPR001980	F	GO:0004595; GO:0015937
		PRINTS	PR01020	Lipopolysaccharide core biosynthesis protein signature	49	73	IPR001980	F	GO:0004595; GO:0015937
		ProDom	PD016147	Ligase Lyase Citrate Pro-3S-Lyase Synthetase Transferase Cytidyltransferase-Related:Citrate:Acetate:SH-Citrate Citc Plasmid	2	75			
		SUPERFAMILY	SSF52374		2	138			

Mycoplasma species	Amino acid region	Database	Database ID	Database signature description	ID region start	ID region end	InterPro ID	ID type	Gene Ontology (GO) term
		TIGRFAM	TIGR01510	coaD_prev_kdtB: pantetheine-phosphate adenylyltransferase	3	141	IPR001980	F	GO:0004595; GO:0015937
		TIGRFAM	TIGR00125	cyt_tran_rel: cytidyltransferase-like domain	3	62	IPR004821	D	GO:0003824; GO:0009058
M. simbae	142	Gene3D	G3DSA:3.40.50.620		1	138	IPR014729	H	
		PANTHER	PTHR21342		1	136			
		PANTHER	PTHR21342:SF1		1	136	IPR001980	F	GO:0004595; GO:0015937
M. simbae	142	Pfam	PF01467	Cytidylyltransferase-like	5	134	IPR004821	D	GO:0003824; GO:0009058
		PRINTS	PR01020	Lipopolysaccharide core biosynthesis protein signature	20	41	IPR001980	F	GO:0004595; GO:0015937
		PRINTS	PR01020	Lipopolysaccharide core biosynthesis protein signature	2	20	IPR001980	F	GO:0004595; GO:0015937
		PRINTS	PR01020	Lipopolysaccharide core biosynthesis protein signature	113	135	IPR001980	F	GO:0004595; GO:0015937
		PRINTS	PR01020	Lipopolysaccharide core biosynthesis protein signature	49	73	IPR001980	F	GO:0004595; GO:0015937
		PRINTS	PR01020	Lipopolysaccharide core biosynthesis protein signature	86	102	IPR001980	F	GO:0004595; GO:0015937
		SUPERFAMILY	SSF52374		2	135			
		TIGRFAM	TIGR01510	coaD_prev_kdtB: pantetheine-phosphate adenylyltransferase	4	134	IPR001980	F	GO:0004595; GO:0015937
		TIGRFAM	TIGR00125	cyt_tran_rel: cytidyltransferase-like domain	4	56	IPR004821	D	GO:0003824; GO:0009058
M. sturni	144	Gene3D	G3DSA:3.40.50.620		1	144	IPR014729	H	
		Hamap	MF_00151	Phosphopantetheine adenylyltransferase [coaD].	4	143	IPR001980	F	GO:0004595; GO:0015937
		PANTHER	PTHR21342:SF1		4	143	IPR001980	F	GO:0004595; GO:0015937
		PANTHER	PTHR21342		4	143			
		Pfam	PF01467	Cytidylyltransferase-like	7	136	IPR004821	D	GO:0003824; GO:0009058
		PRINTS	PR01020	Lipopolysaccharide core biosynthesis protein signature	4	22	IPR001980	F	GO:0004595; GO:0015937
		PRINTS	PR01020	Lipopolysaccharide core biosynthesis protein signature	22	43	IPR001980	F	GO:0004595; GO:0015937
		PRINTS	PR01020	Lipopolysaccharide core biosynthesis protein signature	115	137	IPR001980	F	GO:0004595; GO:0015937
		PRINTS	PR01020	Lipopolysaccharide core biosynthesis protein signature	51	75	IPR001980	F	GO:0004595; GO:0015937
		PRINTS	PR01020	Lipopolysaccharide core biosynthesis protein signature	88	104	IPR001980	F	GO:0004595; GO:0015937
		SUPERFAMILY	SSF52374		4	142			

Mycoplasma species	Amino acid region	Database	Database ID	Database signature description	ID region start	ID region end	InterPro ID	ID type	Gene Ontology (GO) term
		TIGRFAM	TIGR01510	coaD_prev_kdtB: pantetheine-phosphate adenylyltransferase	5	144	IPR001980	F	GO:0004595; GO:0015937
		TIGRFAM	TIGR00125	cyt_tran_rel: cytidyltransferase-like domain	5	62	IPR004821	D	GO:0003824; GO:0009058
M. synoviae	148	Gene3D	G3DSA:3.40.50.620		1	147	IPR014729	H	
		Hamap	MF_00151	Phosphopantetheine adenylyltransferase [coaD].	7	148	IPR001980	F	GO:0004595; GO:0015937
		PANTHER	PTHR21342		7	143			
M. synoviae	148	PANTHER	PTHR21342:SF1		7	143	IPR001980	F	GO:0004595; GO:0015937
		Pfam	PF01467	Cytidylyltransferase-like	10	139	IPR004821	D	GO:0003824; GO:0009058
		PRINTS	PR01020	Lipopolysaccharide core biosynthesis protein signature	7	25	IPR001980	F	GO:0004595; GO:0015937
		PRINTS	PR01020	Lipopolysaccharide core biosynthesis protein signature	118	140	IPR001980	F	GO:0004595; GO:0015937
		PRINTS	PR01020	Lipopolysaccharide core biosynthesis protein signature	25	46	IPR001980	F	GO:0004595; GO:0015937
		PRINTS	PR01020	Lipopolysaccharide core biosynthesis protein signature	91	107	IPR001980	F	GO:0004595; GO:0015937
		SUPERFAMILY	SSF52374		7	145			
		TIGRFAM	TIGR00125	cyt_tran_rel: cytidyltransferase-like domain	9	60	IPR004821	D	GO:0003824; GO:0009058
		TIGRFAM	TIGR01510	coaD_prev_kdtB: pantetheine-phosphate adenylyltransferase	9	139	IPR001980	F	GO:0004595; GO:0015937
M. testudinis	151	Gene3D	G3DSA:3.40.50.620		1	149	IPR014729	H	
		Hamap	MF_00151	Phosphopantetheine adenylyltransferase [coaD].	3	150	IPR001980	F	GO:0004595; GO:0015937
		PANTHER	PTHR21342:SF1		1	142	IPR001980	F	GO:0004595; GO:0015937
		PANTHER	PTHR21342		1	142			
		Pfam	PF01467	Cytidylyltransferase-like	6	136	IPR004821	D	GO:0003824; GO:0009058
		PRINTS	PR01020	Lipopolysaccharide core biosynthesis protein signature	3	21	IPR001980	F	GO:0004595; GO:0015937
		PRINTS	PR01020	Lipopolysaccharide core biosynthesis protein signature	21	42	IPR001980	F	GO:0004595; GO:0015937
		PRINTS	PR01020	Lipopolysaccharide core biosynthesis protein signature	88	104	IPR001980	F	GO:0004595; GO:0015937
		PRINTS	PR01020	Lipopolysaccharide core biosynthesis protein signature	115	137	IPR001980	F	GO:0004595; GO:0015937
		SUPERFAMILY	SSF52374		3	142			
		TIGRFAM	TIGR00125	cyt_tran_rel: cytidyltransferase-like domain	4	61	IPR004821	D	GO:0003824; GO:0009058

Mycoplasma species	Amino acid region	Database	Database ID	Database signature description	ID region start	ID region end	InterPro ID	ID type	Gene Ontology (GO) term
		TIGRFAM	TIGR01510	coaD_prev_kdtB: pantetheine-phosphate adenylyltransferase	4	140	IPR001980	F	GO:0004595; GO:0015937
M. yeatsii	140	Gene3D	G3DSA:3.40.50.620		1	138	IPR014729	H	
		Hamap	MF_00151	Phosphopantetheine adenylyltransferase [coaD].	2	140	IPR001980	F	GO:0004595; GO:0015937
		PANTHER	PTHR21342		1	138			
		PANTHER	PTHR21342:SF1		1	138	IPR001980	F	GO:0004595; GO:0015937
M. yeatsii	140	Pfam	PF01467	Cytidylyltransferase-like	5	134	IPR004821	D	GO:0003824; GO:0009058
		PRINTS	PR01020	Lipopolysaccharide core biosynthesis protein signature	86	102	IPR001980	F	GO:0004595; GO:0015937
		PRINTS	PR01020	Lipopolysaccharide core biosynthesis protein signature	20	41	IPR001980	F	GO:0004595; GO:0015937
		PRINTS	PR01020	Lipopolysaccharide core biosynthesis protein signature	2	20	IPR001980	F	GO:0004595; GO:0015937
		PRINTS	PR01020	Lipopolysaccharide core biosynthesis protein signature	49	73	IPR001980	F	GO:0004595; GO:0015937
		PRINTS	PR01020	Lipopolysaccharide core biosynthesis protein signature	113	135	IPR001980	F	GO:0004595; GO:0015937
		SUPERFAMILY	SSF52374		2	138			
		TIGRFAM	TIGR00125	cyt_tran_rel: cytidyltransferase-like domain	3	61	IPR004821	D	GO:0003824; GO:0009058
		TIGRFAM	TIGR01510	coaD_prev_kdtB: pantetheine-phosphate adenylyltransferase	3	139	IPR001980	F	GO:0004595; GO:0015937

[a]ID type abbreviations – H, Homologous superfamily; F, Family; D, Domain
[b]InterPro ID – IPR014729: Rossmann-like alpha/beta/alpha sandwich fold; IPR001980: Phosphopantetheine adenylyltransferase; IPR004821: Cytidyltransferase-like domain
[c]GO term (Biological Process) – GO:0009058: Biosynthetic process; GO:0015937: Coenzyme A biosynthetic process
GO term (Molecular Function) – GO:0003824: Catalytic activity; GO:0004595: Pantetheine-phosphate adenylyltransferase activity

Supplementary Table 12 PPAT MEME + motif locations

Mycoplasma species	*p*-value	Motif locations			
		Motif 1	Motif 2	Motif 3	Motif 4
M. agalactiae	8.04e-99	1-42	47-61	63-83	85-134
M. alligatoris	4.76e-92	4-45	50-64	68-88	90-139
M. alvi	4.46e-57	3-44	49-63	-	88-137
M. anatis	8.32e-97	3-44	49-63	65-85	87-136
M. arginini	9.84e-101	1-42	47-61	63-83	85-134
M. bovigenitalium	1.33e-88	1-42	47-61	63-83	85-134

Mycoplasma species	*p*-value	Motif locations			
		Motif 1	Motif 2	Motif 3	Motif 4
M. bovis	9.01e-101	1-42	47-61	63-83	85-134
M. buteonis	3.59e-77	5-46	51-65	69-89	91-140
M. californicum	6.44e-91	1-42	47-61	63-83	85-134
M. canis	5.39e-74	4-45	49-63	66-86	88-137
M. capricolum	1.01e-89	1-42	47-61	63-83	85-134
M. collis	1.04e-81	7-48	53-67	69-89	91-140
M. columbinum	1.37e-97	2-43	48-62	65-85	87-136
M. columborale	4.20e-89	3-44	49-63	65-85	87-136
M. conjunctivae	2.15e-95	10-51	56-70	72-92	94-143
M. cricetuli	4.57e-95	3-44	49-63	65-85	87-136
M. crocodyli	3.14e-91	5-46	51-65	69-89	91-140
M. felifaucium	7.02e-97	3-44	49-63	65-85	87-136
M. felis	9.55e-85	2-43	47-61	63-83	85-134
M. fermentans	8.34e-94	2-43	48-62	64-84	86-135
M. gallinaceum	6.43e-91	4-45	50-64	66-86	88-137
M. gallinarum	1.52e-99	2-43	48-62	64-84	86-135
M. iners	4.14e-87	2-43	48-62	64-84	86-135
M. iowae	1.23e-60	5-46	50-64	-	89-138
M. leachii	6.70e-90	1-42	47-61	63-83	85-134
M. leonicaptivi	6.91e-72	3-44	48-62	64-84	86-135
M. lipofaciens	2.75e-95	1-42	47-61	63-83	85-134
M. mobile	7.89e-27	8-49	54-68	-	-
M. molare	1.33e-89	1-42	47-61	63-83	85-134
M. mycoides subsp. *capri*	1.37e-89	1-42	47-61	63-83	85-134
M. mycoides subsp. *mycoides*	1.05e-87	1-42	47-61	63-83	85-134
M. opalescens	5.58e-90	1-42	47-61	64-84	86-135
M. penetrans	4.73e-53	7-48	52-66	-	91-140
M. pirum	1.88e-59	3-44	49-63	-	88-137
M. primatum	7.48e-105	1-42	47-61	63-83	85-134
M. pulmonis	5.26e-87	5-46	51-65	69-89	91-140
M. putrefaciens	4.92e-74	1-42	47-61	63-83	85-134
M. simbae	2.98e-93	1-42	47-61	63-83	85-134
M. sturni	1.22e-91	3-44	49-63	65-85	87-136
M. synoviae	5.65e-98	6-47	52-66	68-88	90-139

Mycoplasma species	*p*-value	Motif locations			
		Motif 1	Motif 2	Motif 3	Motif 4
M. testudinis	1.46e-63	2-43	48-62	-	87-136
M. yeatsii	1.25e-75	1-42	47-61	63-83	85-134

Supplementary Table 13 DPCK CDD results

Query	Hit type	ID region start	ID region end	E-Value	Accession	Short name	Superfamily	Definition
M. sp. Ms02	non-specific	1	122	1.50e-13	COG0237	CoaE	cl30785	Dephospho-CoA kinase
	superfamily	1	122	1.50e-13	cl30785	CoaE superfamily	-	Dephospho-CoA kinase
	non-specific	1	122	6.33e-11	cd02022	DPCK	cl17190	Dephospho-CoA kinase
	superfamily	1	122	6.33e-11	cl17190	NK superfamily	-	Nucleoside/nucleotide kinase superfamily
	non-specific	2	122	2.20e-08	TIGR00152	TIGR00152	cl17190	Dephospho-CoA kinase
	non-specific	2	120	2.87e-05	PRK00081	coaE	cl17190	Dephospho-CoA kinase
	non-specific	2	48	0.000637	cd01983	Fer4_NifH	cl28886	The Fer4_NifH superfamily
	superfamily	2	48	0.000637	cl28886	Fer4_NifH superfamily	-	The Fer4_NifH superfamily
	non-specific	1	33	0.001471	COG1936	Fap7	cl17190	Broad-specificity NMP kinase
M. agalactiae	non-specific	1	143	2.34e-23	COG0237	CoaE	cl30785	Dephospho-CoA kinase
	superfamily	1	143	2.34e-23	cl30785	CoaE superfamily	-	Dephospho-CoA kinase
	specific	1	143	8.86e-21	cd02022	DPCK	cl17190	Dephospho-CoA kinase
	superfamily	1	143	8.86e-21	cl17190	NK superfamily	-	Nucleoside/nucleotide kinase superfamily
	non-specific	2	186	2.85e-14	TIGR00152	TIGR00152	cl17190	Dephospho-CoA kinase
	non-specific	2	146	2.73e-13	PRK00081	coaE	cl17190	Dephospho-CoA kinase
	non-specific	2	142	1.59e-06	pfam01121	CoaE	cl17190	Dephospho-CoA kinase
	non-specific	1	121	9.07e-06	COG1936	Fap7	cl17190	Broad-specificity NMP kinase
	non-specific	1	92	0.004474	PLN02422	PLN02422	cl17190	Dephospho-CoA kinase
	non-specific	2	129	0.007723	COG0572	Udk	cl28606	Uridine kinase superfamily
	superfamily	2	129	0.007723	cl28606	Udk superfamily	-	Uridine kinase superfamily
	non-specific	2	35	0.009588	PRK03839	PRK03839	cl17190	putative kinase
M. alligatoris	non-specific	1	132	1.34e-11	COG0237	CoaE	cl30785	Dephospho-CoA kinase
	superfamily	1	132	1.34e-11	cl30785	CoaE superfamily	-	Dephospho-CoA kinase
	non-specific	1	132	4.77e-11	cd02022	DPCK	cl17190	Dephospho-CoA kinase
	superfamily	1	132	4.77e-11	cl17190	NK superfamily	-	Nucleoside/nucleotide kinase superfamily
	non-specific	2	132	2.79e-05	PRK00081	coaE	cl17190	Dephospho-CoA kinase
	non-specific	2	136	0.000429	TIGR00152	TIGR00152	cl17190	Dephospho-CoA kinase
M. alvi	non-specific	2	188	2.29e-24	TIGR00152	TIGR00152	cl17190	Dephospho-CoA kinase
	superfamily	2	188	2.29e-24	cl17190	NK superfamily	-	Nucleoside/nucleotide kinase superfamily

Query	Hit type	ID region start	ID region end	E-Value	Accession	Short name	Superfamily	Definition
	specific	2	151	1.45e-21	cd02022	DPCK	cl17190	Dephospho-CoA kinase
	non-specific	1	191	1.66e-20	PRK00081	coaE	cl17190	Dephospho-CoA kinase
	non-specific	1	191	5.20e-20	COG0237	CoaE	cl30785	Dephospho-CoA kinase
	superfamily	1	191	5.20e-20	cl30785	CoaE superfamily	-	Dephospho-CoA kinase
	non-specific	1	99	2.52e-12	pfam01121	CoaE	cl17190	Dephospho-CoA kinase
	non-specific	2	106	8.98e-11	PLN02422	PLN02422	cl17190	Dephospho-CoA kinase
	non-specific	5	89	1.98e-08	PRK14730	coaE	cl17190	Dephospho-CoA kinase
	non-specific	2	194	2.83e-06	PRK14732	coaE	cl17190	Dephospho-CoA kinase
	non-specific	1	21	0.002608	PRK04040	PRK04040	cl17190	adenylate kinase
M. alvi	non-specific	2	44	0.003877	cd03255	ABC_MJ0796_LolCDE_FtsE	cl25403	ATP-binding cassette domain of the transporters involved in export of lipoprotein and macrolide, and cell division protein
	superfamily	2	44	0.003877	cl25403	ABC_ATPase superfamily	-	ATP-binding cassette transporter nucleotide-binding domain
	non-specific	1	21	0.005459	COG2019	AdkA	cl17190	Archaeal adenylate kinase
	non-specific	1	21	0.005635	COG1936	Fap7	cl17190	Broad-specificity NMP kinase
	non-specific	2	44	0.00671	cd03263	ABC_subfamily_A	cl25403	ATP-binding cassette domain of the lipid transporters, subfamily A
	non-specific	2	50	0.007481	TIGR02982	heterocyst_DevA	cl28181	ABC exporter ATP-binding subunit, DevA family
	superfamily	2	50	0.007481	cl28181	AAA superfamily	-	ATPases associated with a variety of cellular activities
M. anatis	non-specific	1	137	2.61e-13	COG0237	CoaE	cl30785	Dephospho-CoA kinase
	superfamily	1	137	2.61e-13	cl30785	CoaE superfamily	-	Dephospho-CoA kinase
	non-specific	1	137	2.26e-09	cd02022	DPCK	cl17190	Dephospho-CoA kinase
	superfamily	1	137	2.26e-09	cl17190	NK superfamily	-	Nucleoside/nucleotide kinase superfamily
	non-specific	2	137	4.69e-05	TIGR00152	TIGR00152	cl17190	Dephospho-CoA kinase
	non-specific	2	137	4.71e-05	PRK00081	coaE	cl17190	Dephospho-CoA kinase
	non-specific	2	133	0.000588	pfam01121	CoaE	cl17190	Dephospho-CoA kinase
	non-specific	2	190	0.001564	PRK14733	coaE	cl17190	Dephospho-CoA kinase
M. arginini	non-specific	1	141	5.25e-13	COG0237	CoaE	cl30785	Dephospho-CoA kinase
	superfamily	1	141	5.25e-13	cl30785	CoaE superfamily	-	Dephospho-CoA kinase
	non-specific	1	147	2.86e-11	cd02022	DPCK	cl17190	Dephospho-CoA kinase
	superfamily	1	147	2.86e-11	cl17190	NK superfamily	-	Nucleoside/nucleotide kinase superfamily
	non-specific	2	166	5.29e-07	TIGR00152	TIGR00152	cl17190	Dephospho-CoA kinase
	non-specific	2	147	0.006534	PRK00081	coaE	cl17190	Dephospho-CoA kinase
	non-specific	1	76	0.009556	pfam05272	VirE	cl23993	Virulence-associated protein E (these proteins contain a P-loop motif)
	superfamily	1	76	0.009556	cl23993	VirE superfamily	-	Virulence-associated protein E (these proteins contain a P-loop motif)
M. bovigenitalium	non-specific	1	190	2.24e-17	COG0237	CoaE	cl30785	Dephospho-CoA kinase
	superfamily	1	190	2.24e-17	cl30785	CoaE superfamily	-	Dephospho-CoA kinase
	non-specific	1	178	3.64e-12	cd02022	DPCK	cl17190	Dephospho-CoA kinase
	superfamily	1	178	3.64e-12	cl17190	NK superfamily	-	Nucleoside/nucleotide kinase superfamily

Query	Hit type	ID region start	ID region end	E-Value	Accession	Short name	Superfamily	Definition
	non-specific	2	137	2.67e-07	TIGR00152	TIGR00152	cl17190	Dephospho-CoA kinase
	non-specific	2	88	6.80e-06	PRK00081	coaE	cl17190	Dephospho-CoA kinase
	non-specific	1	117	0.00034	PRK14732	coaE	cl17190	Dephospho-CoA kinase
M. bovis	non-specific	1	149	4.53e-21	COG0237	CoaE	cl30785	Dephospho-CoA kinase
	superfamily	1	149	4.53e-21	cl30785	CoaE superfamily	-	Dephospho-CoA kinase
	specific	1	146	6.23e-20	cd02022	DPCK	cl17190	Dephospho-CoA kinase
	superfamily	1	146	6.23e-20	cl17190	NK superfamily	-	Nucleoside/nucleotide kinase superfamily
	non-specific	2	177	1.36e-11	TIGR00152	TIGR00152	cl17190	Dephospho-CoA kinase
	non-specific	2	146	4.16e-11	PRK00081	coaE	cl17190	Dephospho-CoA kinase
M. bovis	non-specific	1	78	2.40e-06	COG1936	Fap7	cl17190	Broad-specificity NMP kinase
	non-specific	1	91	0.00063	PRK14731	coaE	cl17190	Dephospho-CoA kinase
	non-specific	2	33	0.001113	cd01983	Fer4_NifH	cl28886	The Fer4_NifH superfamily
	superfamily	2	33	0.001113	cl28886	Fer4_NifH superfamily	-	The Fer4_NifH superfamily
	non-specific	2	148	0.001637	pfam13238	AAA_18	cl21455	AAA domain
	superfamily	2	148	0.001637	cl21455	P-loop_NTPase superfamily	-	P-loop containing Nucleoside Triphosphate Hydrolases
M. bovoculi (HAD-DPCK)	specific	1	263	9.47e-37	COG0561	Cof	cl26787	Hydroxymethylpyrimidine pyrophosphatase and other HAD family phosphatases [Coenzyme transport and metabolism, General function prediction only]
	superfamily	1	263	9.47e-37	cl26787	Hydrolase_3 superfamily	-	haloacid dehalogenase-like hydrolase
	non-specific	7	242	7.44e-36	pfam08282	Hydrolase_3	cl26787	haloacid dehalogenase-like hydrolase
	non-specific	7	256	2.02e-33	TIGR00099	Cof-subfamily	cl26787	Cof subfamily of IIB subfamily of haloacid dehalogenase superfamily
	non-specific	7	258	7.41e-31	cd07516	HAD_Pase	cl21460	phosphatase, similar to Escherichia coli Cof and Thermotoga maritima TM0651
	superfamily	7	258	7.41e-31	cl21460	HAD_like superfamily	-	Haloacid Dehalogenase-like Hydrolases
	non-specific	1	261	1.23e-14	PRK01158	PRK01158	cl26787	phosphoglycolate phosphatase
	non-specific	4	244	6.09e-14	cd07517	HAD_HPP	cl21460	phosphatase, similar to Bacteroides thetaiotaomicron VPI-5482 BT4131 hexose phosphate phosphatase
	non-specific	8	259	3.25e-12	TIGR01482	SPP-subfamily	cl26787	sucrose-phosphate phosphatase subfamily
	non-specific	266	400	5.02e-12	COG0237	CoaE	cl30785	Dephospho-CoA kinase
	superfamily	266	400	5.02e-12	cl30785	CoaE superfamily	-	Dephospho-CoA kinase
	non-specific	266	393	2.03e-11	cd02022	DPCK	cl17190	Dephospho-CoA kinase
	superfamily	266	393	2.03e-11	cl17190	NK superfamily	-	Nucleoside/nucleotide kinase superfamily
	non-specific	7	218	1.49e-10	TIGR01484	HAD-SF-IIB	cl26787	HAD-superfamily
	non-specific	9	218	2.48e-09	cd02605	HAD_SPP	cl21460	sucrose-phosphatase, similar to Synechocystis sp PCC 6803 SPP
	non-specific	4	244	4.82e-08	TIGR01487	Pglycolate_arch	cl26787	phosphoglycolate phosphatase, TA0175-type
	non-specific	266	391	9.33e-07	TIGR00152	TIGR00152	cl17190	Dephospho-CoA kinase
	non-specific	3	240	9.70e-07	PRK10976	PRK10976	cl26787	putative hydrolase
	non-specific	1	261	1.95e-06	PRK10513	PRK10513	cl26787	sugar phosphate phosphatase
	non-specific	266	394	2.91e-05	PRK00081	coaE	cl17190	Dephospho-CoA kinase

Query	Hit type	ID region start	ID region end	E-Value	Accession	Short name	Superfamily	Definition
	non-specific	148	257	3.67e-05	cd07518	HAD_YbiV-Like	cl21460	Escherichia coli YbiV sugar phosphatase/phosphotransferase and related proteins
	non-specific	2	259	7.30e-05	PLN02887	PLN02887	cl26787	hydrolase family protein
	non-specific	5	75	0.00369	TIGR01486	HAD-SF-IIB-MPGP	cl26786	mannosyl-3-phosphoglycerate phosphatase family
	superfamily	5	75	0.00369	cl26786	YedP superfamily	-	Predicted mannosyl-3-phosphoglycerate phosphatase, HAD superfamily
	non-specific	178	218	0.004772	pfam05116	S6PP	cl26787	Sucrose-6F-phosphate phosphohydrolase
M. bovoculi (HAD-DPCK)	non-specific	175	259	0.008491	cd07514	HAD_Pase	cl21460	phosphatase, similar to Thermoplasma acidophilum TA0175 phosphoglycolate phosphatase (PCPase), and Pyrococcus horikoshii PH1421, a magnesium-dependent phosphatase
M. buteonis	non-specific	1	185	5.06e-12	COG0237	CoaE	cl30785	Dephospho-CoA kinase
	superfamily	1	185	5.06e-12	cl30785	CoaE superfamily	-	Dephospho-CoA kinase
	non-specific	1	129	2.49e-09	cd02022	DPCK	cl17190	Dephospho-CoA kinase
	superfamily	1	129	2.49e-09	cl17190	NK superfamily	-	Nucleoside/nucleotide kinase superfamily
	non-specific	2	128	2.01e-06	TIGR00152	TIGR00152	cl17190	Dephospho-CoA kinase
M. californicum	non-specific	1	189	3.53e-15	COG0237	CoaE	cl30785	Dephospho-CoA kinase
	superfamily	1	189	3.53e-15	cl30785	CoaE superfamily	-	Dephospho-CoA kinase
	non-specific	1	148	1.98e-14	cd02022	DPCK	cl17190	Dephospho-CoA kinase
	superfamily	1	148	1.98e-14	cl17190	NK superfamily	-	Nucleoside/nucleotide kinase superfamily
	non-specific	2	148	5.93e-08	PRK00081	coaE	cl17190	Dephospho-CoA kinase
	non-specific	2	189	6.62e-08	TIGR00152	TIGR00152	cl17190	Dephospho-CoA kinase
	non-specific	2	137	0.000139	pfam01121	CoaE	cl17190	Dephospho-CoA kinase
M. canis	non-specific	1	129	8.82e-10	COG0237	CoaE	cl30785	Dephospho-CoA kinase
	superfamily	1	129	8.82e-10	cl30785	CoaE superfamily	-	Dephospho-CoA kinase
	non-specific	2	173	5.03e-06	TIGR00152	TIGR00152	cl17190	Dephospho-CoA kinase
	superfamily	2	173	5.03e-06	cl17190	NK superfamily	-	Nucleoside/nucleotide kinase superfamily
	non-specific	1	129	1.37e-05	cd02022	DPCK	cl17190	Dephospho-CoA kinase
	non-specific	2	34	0.001443	cd01983	Fer4_NifH	cl28886	The Fer4_NifH superfamily
	superfamily	2	34	0.001443	cl28886	Fer4_NifH superfamily	-	The Fer4_NifH superfamily
	non-specific	1	88	0.006613	COG0488	Uup	cl28181	ATPase components of ABC transporters with duplicated ATPase domains [General function prediction only]
	superfamily	1	88	0.006613	cl28181	AAA superfamily	-	ATPases associated with a variety of cellular activities
M. capricolum subsp. *capricolum*	specific	6	179	2.24e-38	cd02022	DPCK	cl17190	Dephospho-CoA kinase
	superfamily	6	179	2.24e-38	cl17190	NK superfamily	-	Nucleoside/nucleotide kinase superfamily
	non-specific	4	174	1.85e-26	PRK00081	coaE	cl17190	Dephospho-CoA kinase
	non-specific	4	179	1.75e-23	COG0237	CoaE	cl30785	Dephospho-CoA kinase
	superfamily	4	179	1.75e-23	cl30785	CoaE superfamily	-	Dephospho-CoA kinase
	non-specific	5	174	3.75e-23	pfam01121	CoaE	cl17190	Dephospho-CoA kinase
	non-specific	6	174	7.13e-18	TIGR00152	TIGR00152	cl17190	Dephospho-CoA kinase
	non-specific	6	188	5.99e-12	PRK14730	coaE	cl17190	Dephospho-CoA kinase

Query	Hit type	ID region start	ID region end	E-Value	Accession	Short name	Superfamily	Definition
	non-specific	6	179	7.13e-11	PRK14732	coaE	cl17190	Dephospho-CoA kinase
	non-specific	7	174	4.72e-08	PRK14733	coaE	cl17190	Dephospho-CoA kinase
	non-specific	6	179	2.23e-05	PLN02422	PLN02422	cl17190	Dephospho-CoA kinase
	non-specific	7	174	0.000153	PRK14734	coaE	cl17190	Dephospho-CoA kinase
	non-specific	3	174	0.000277	PRK14731	coaE	cl17190	Dephospho-CoA kinase
M. capricolum subsp. *capripneumoniae*	specific	2	175	1.30e-36	cd02022	DPCK	cl17190	Dephospho-CoA kinase
	superfamily	2	175	1.30e-36	cl17190	NK superfamily	-	Nucleoside/nucleotide kinase superfamily
	non-specific	1	170	1.66e-24	PRK00081	coaE	cl17190	Dephospho-CoA kinase
	non-specific	1	175	1.93e-22	COG0237	CoaE	cl30785	Dephospho-CoA kinase
	superfamily	1	175	1.93e-22	cl30785	CoaE superfamily	-	Dephospho-CoA kinase
	non-specific	1	170	3.53e-22	pfam01121	CoaE	cl17190	Dephospho-CoA kinase
	non-specific	2	170	1.19e-16	TIGR00152	TIGR00152	cl17190	Dephospho-CoA kinase
	non-specific	2	184	2.12e-11	PRK14730	coaE	cl17190	Dephospho-CoA kinase
	non-specific	2	175	2.21e-11	PRK14732	coaE	cl17190	Dephospho-CoA kinase
	non-specific	3	170	5.81e-08	PRK14733	coaE	cl17190	Dephospho-CoA kinase
	non-specific	2	175	0.001038	PLN02422	PLN02422	cl17190	Dephospho-CoA kinase
	non-specific	2	170	0.001838	PRK14731	coaE	cl17190	Dephospho-CoA kinase
M. collis	specific	1	129	9.11e-25	COG0237	CoaE	cl30785	Dephospho-CoA kinase
	superfamily	1	129	9.11e-25	cl30785	CoaE superfamily	-	Dephospho-CoA kinase
	specific	1	148	1.10e-20	cd02022	DPCK	cl17190	Dephospho-CoA kinase
	superfamily	1	148	1.10e-20	cl17190	NK superfamily	-	Nucleoside/nucleotide kinase superfamily
	non-specific	2	148	3.80e-17	TIGR00152	TIGR00152	cl17190	Dephospho-CoA kinase
	non-specific	2	148	6.58e-15	PRK00081	coaE	cl17190	Dephospho-CoA kinase
	non-specific	2	150	4.58e-07	PRK14730	coaE	cl17190	Dephospho-CoA kinase
	non-specific	1	80	4.87e-07	COG1936	Fap7	cl17190	Broad-specificity NMP kinase
	non-specific	2	129	9.87e-06	pfam01121	CoaE	cl17190	Dephospho-CoA kinase
	non-specific	2	33	2.26e-05	cd01983	Fer4_NifH	cl28886	The Fer4_NifH superfamily
	superfamily	2	33	2.26e-05	cl28886	Fer4_NifH superfamily	-	The Fer4_NifH superfamily
	non-specific	2	80	0.000107	PRK03839	PRK03839	cl17190	putative kinase
	non-specific	2	92	0.000163	PRK04182	PRK04182	cl28332	cytidylate kinase
	superfamily	2	92	0.000163	cl28332	CmkB superfamily	-	Cytidylate kinase
	non-specific	1	86	0.000597	PLN02422	PLN02422	cl17190	Dephospho-CoA kinase
	specific	2	124	0.000634	smart00382	AAA	cl28181	ATPases associated with a variety of cellular activities
	superfamily	2	124	0.000634	cl28181	AAA superfamily	-	ATPases associated with a variety of cellular activities
	non-specific	2	73	0.001659	COG1102	CmkB	cl28332	Cytidylate kinase
	non-specific	2	42	0.001875	PRK04040	PRK04040	cl17190	adenylate kinase
	non-specific	1	20	0.002192	cd03255	ABC_MJ0796_LolCDE_FtsE	cl25403	ATP-binding cassette domain of the transporters involved in export of lipoprotein and macrolide, and cell division protein
	superfamily	1	20	0.002192	cl25403	ABC_ATPase superfamily	-	ATP-binding cassette transporter nucleotide-binding domain

Query	Hit type	ID region start	ID region end	E-Value	Accession	Short name	Superfamily	Definition
	non-specific	5	81	0.00335	pfam13207	AAA_17	cl21455	AAA domain
	superfamily	5	81	0.00335	cl21455	P-loop_NTPase superfamily	-	P-loop containing Nucleoside Triphosphate Hydrolases
M. collis	non-specific	1	26	0.003507	cd03116	MobB	cl28886	Molybdenum is an essential trace element in the form of molybdenum cofactor (Moco) which is associated with the metabolism of nitrogen, carbon and sulfur by redox active enzymes. In E. coli, the synthesis of Moco involves genes from several loci: moa, mob, mod, moe and mog. The mob locus contains mobA and mobB genes. MobB catalyzes the attachment of the guanine dinucleotide to molybdopterin.
	non-specific	2	26	0.003758	pfam13521	AAA_28	cl21455	AAA domain
	non-specific	2	145	0.00394	PRK14733	coaE	cl17190	Dephospho-CoA kinase
	non-specific	1	20	0.006373	COG1136	LolD	cl28181	ABC-type lipoprotein export system, ATPase component [Cell wall/membrane/envelope biogenesis]
	non-specific	2	73	0.00661	cd02034	CooC	cl28886	The accessory protein CooC, which contains a nucleotide-binding domain (P-loop) near the N-terminus, participates in the maturation of the nickel center of carbon monoxide dehydrogenase (CODH).
	non-specific	2	151	0.007951	cd01851	GBP	cl21455	Guanylate-binding protein (GBP) family (N-terminal domain)
	non-specific	1	92	0.009239	TIGR02173	cyt_kin_arch	cl28332	cytidylate kinase
	non-specific	2	28	0.009861	cd02020	CMPK	cl17190	Cytidine monophosphate kinase
M. columbinum	non-specific	1	137	1.47e-22	COG0237	CoaE	cl30785	Dephospho-CoA kinase
	superfamily	1	137	1.47e-22	cl30785	CoaE superfamily	-	Dephospho-CoA kinase
	specific	1	137	4.33e-22	cd02022	DPCK	cl17190	Dephospho-CoA kinase
	superfamily	1	137	4.33e-22	cl17190	NK superfamily	-	Nucleoside/nucleotide kinase superfamily
	non-specific	2	137	1.79e-12	PRK00081	coaE	cl17190	Dephospho-CoA kinase
	non-specific	2	140	5.95e-12	TIGR00152	TIGR00152	cl17190	Dephospho-CoA kinase
	non-specific	2	137	2.38e-07	pfam01121	CoaE	cl17190	Dephospho-CoA kinase
	non-specific	1	88	0.000169	PRK14732	coaE	cl17190	Dephospho-CoA kinase
	non-specific	2	126	0.000305	pfam13238	AAA_18	cl21455	AAA domain
	superfamily	2	126	0.000305	cl21455	P-loop_NTPase superfamily	-	P-loop containing Nucleoside Triphosphate Hydrolases
	non-specific	1	89	0.000568	PRK14731	coaE	cl17190	Dephospho-CoA kinase
	non-specific	9	136	0.007099	pfam13207	AAA_17	cl21455	AAA domain
M. columborale	non-specific	1	181	4.64e-11	COG0237	CoaE	cl30785	Dephospho-CoA kinase
	superfamily	1	181	4.64e-11	cl30785	CoaE superfamily	-	Dephospho-CoA kinase
	non-specific	1	144	1.93e-07	cd02022	DPCK	cl17190	Dephospho-CoA kinase
	superfamily	1	144	1.93e-07	cl17190	NK superfamily	-	Nucleoside/nucleotide kinase superfamily
	non-specific	1	156	2.71e-05	PRK00081	coaE	cl17190	Dephospho-CoA kinase
	non-specific	2	136	0.000544	TIGR00152	TIGR00152	cl17190	Dephospho-CoA kinase

Query	Hit type	ID region start	ID region end	E-Value	Accession	Short name	Superfamily	Definition
	non-specific	4	137	0.000723	PRK14731	coaE	cl17190	Dephospho-CoA kinase
	non-specific	1	93	0.000804	PRK14732	coaE	cl17190	Dephospho-CoA kinase
M. columborale	non-specific	1	30	0.006918	PRK14734	coaE	cl17190	Dephospho-CoA kinase
M. conjunctivae (HAD-DPCK)	specific	3	264	1.60e-34	COG0561	Cof	cl26787	Hydroxymethylpyrimidine pyrophosphatase and other HAD family phosphatases [Coenzyme transport and metabolism, General function prediction only]
	superfamily	3	264	1.60e-34	cl26787	Hydrolase_3 superfamily	-	haloacid dehalogenase-like hydrolase
	non-specific	7	257	2.72e-33	pfam08282	Hydrolase_3	cl26787	haloacid dehalogenase-like hydrolase
	non-specific	6	259	1.10e-26	cd07516	HAD_Pase	cl21460	phosphatase, similar to Escherichia coli Cof and Thermotoga maritima TM0651
	superfamily	6	259	1.10e-26	cl21460	HAD_like superfamily	-	Haloacid Dehalogenase-like Hydrolases
	non-specific	6	257	2.46e-26	TIGR00099	Cof-subfamily	cl26787	Cof subfamily of IIB subfamily of haloacid dehalogenase superfamily
	non-specific	1	260	1.17e-13	PRK01158	PRK01158	cl26787	phosphoglycolate phosphatase
	non-specific	6	220	8.35e-13	TIGR01484	HAD-SF-IIB	cl26787	HAD-superfamily
	non-specific	10	263	2.26e-12	TIGR01482	SPP-subfamily	cl26787	sucrose-phosphate phosphatase subfamily
	non-specific	5	260	2.08e-11	cd07517	HAD_HPP	cl21460	phosphatase, similar to Bacteroides thetaiotaomicron VPI-5482 BT4131 hexose phosphate phosphatase
	non-specific	267	393	8.59e-10	COG0237	CoaE	cl30785	Dephospho-CoA kinase
	superfamily	267	393	8.59e-10	cl30785	CoaE superfamily	-	Dephospho-CoA kinase
	non-specific	267	401	2.23e-09	cd02022	DPCK	cl17190	Dephospho-CoA kinase
	superfamily	267	401	2.23e-09	cl17190	NK superfamily	-	Nucleoside/nucleotide kinase superfamily
	non-specific	5	257	5.30e-09	TIGR01487	Pglycolate_arch	cl26787	phosphoglycolate phosphatase, TA0175-type
	non-specific	5	261	9.68e-09	PRK10513	PRK10513	cl26787	sugar phosphate phosphatase
	non-specific	186	260	5.79e-08	cd07514	HAD_Pase	cl21460	phosphatase, similar to Thermoplasma acidophilum TA0175 phosphoglycolate phosphatase (PCPase), and Pyrococcus horikoshii PH1421, a magnesium-dependent phosphatase
	non-specific	267	414	1.65e-07	TIGR00152	TIGR00152	cl17190	Dephospho-CoA kinase
	non-specific	6	219	2.80e-07	PRK10976	PRK10976	cl26787	putative hydrolase
	non-specific	122	258	5.35e-05	cd07518	HAD_YbiV-Like	cl21460	Escherichia coli YbiV sugar phosphatase/phosphotransferase and related proteins
	non-specific	157	260	0.000276	cd02605	HAD_SPP	cl21460	sucrose-phosphatase, similar to Synechocystis sp PCC 6803 SPP
	non-specific	3	102	0.000756	COG1877	OtsB	cl28591	Trehalose-6-phosphatase [Carbohydrate transport and metabolism]
	superfamily	3	102	0.000756	cl28591	OtsB superfamily	-	Trehalose-6-phosphatase [Carbohydrate transport and metabolism]
	non-specific	1	76	0.001366	PRK00192	PRK00192	cl26786	mannosyl-3-phosphoglycerate phosphatase
	superfamily	1	76	0.001366	cl26786	YedP superfamily	-	Predicted mannosyl-3-phosphoglycerate phosphatase, HAD superfamily
M. cricetuli	non-specific	1	183	2.25e-17	COG0237	CoaE	cl30785	Dephospho-CoA kinase
M. cricetuli	superfamily	1	183	2.25e-17	cl30785	CoaE superfamily	-	Dephospho-CoA kinase

Query	Hit type	ID region start	ID region end	E-Value	Accession	Short name	Superfamily	Definition
	non-specific	1	136	6.93e-13	cd02022	DPCK	cl17190	Dephospho-CoA kinase
	superfamily	1	136	6.93e-13	cl17190	NK superfamily	-	Nucleoside/nucleotide kinase superfamily
	non-specific	2	142	1.58e-08	TIGR00152	TIGR00152	cl17190	Dephospho-CoA kinase
	non-specific	2	136	3.11e-06	PRK00081	coaE	cl17190	Dephospho-CoA kinase
	non-specific	1	41	0.002388	COG1936	Fap7	cl17190	Broad-specificity NMP kinase
	non-specific	2	137	0.004333	COG0572	Udk	cl28606	Uridine kinase superfamily
	superfamily	2	137	0.004333	cl28606	Udk superfamily	-	Uridine kinase superfamily
M. crocodyli	non-specific	1	190	1.56e-15	COG0237	CoaE	cl30785	Dephospho-CoA kinase
	superfamily	1	190	1.56e-15	cl30785	CoaE superfamily	-	Dephospho-CoA kinase
	non-specific	1	137	1.08e-14	cd02022	DPCK	cl17190	Dephospho-CoA kinase
	superfamily	1	137	1.08e-14	cl17190	NK superfamily	-	Nucleoside/nucleotide kinase superfamily
	non-specific	2	173	6.02e-11	TIGR00152	TIGR00152	cl17190	Dephospho-CoA kinase
	non-specific	2	137	1.15e-08	PRK00081	coaE	cl17190	Dephospho-CoA kinase
	non-specific	2	137	3.04e-05	pfam01121	CoaE	cl17190	Dephospho-CoA kinase
M. dispar (HAD-DPCK)	non-specific	7	221	1.49e-29	pfam08282	Hydrolase_3	cl26787	haloacid dehalogenase-like hydrolase
	superfamily	7	221	1.49e-29	cl26787	Hydrolase_3 superfamily	-	haloacid dehalogenase-like hydrolase
	specific	1	266	4.21e-29	COG0561	Cof	cl26787	Hydroxymethylpyrimidine pyrophosphatase and other HAD family phosphatases [Coenzyme transport and metabolism, General function prediction only]
	non-specific	7	221	3.98e-24	cd07516	HAD_Pase	cl21460	phosphatase, similar to Escherichia coli Cof and Thermotoga maritima TM0651
	superfamily	7	221	3.98e-24	cl21460	HAD_like superfamily	-	Haloacid Dehalogenase-like Hydrolases
	non-specific	7	221	8.47e-23	TIGR00099	Cof-subfamily	cl26787	Cof subfamily of IIB subfamily of haloacid dehalogenase superfamily
	non-specific	7	221	5.10e-14	TIGR01484	HAD-SF-IIB	cl26787	HAD-superfamily
	non-specific	269	396	7.14e-13	COG0237	CoaE	cl30785	Dephospho-CoA kinase
	superfamily	269	396	7.14e-13	cl30785	CoaE superfamily	-	Dephospho-CoA kinase
	non-specific	4	108	1.70e-11	cd07517	HAD_HPP	cl21460	phosphatase, similar to Bacteroides thetaiotaomicron VPI-5482 BT4131 hexose phosphate phosphatase
	non-specific	269	396	3.50e-08	cd02022	DPCK	cl17190	Dephospho-CoA kinase
	superfamily	269	396	3.50e-08	cl17190	NK superfamily	-	Nucleoside/nucleotide kinase superfamily
	non-specific	1	221	4.95e-06	PRK01158	PRK01158	cl26787	phosphoglycolate phosphatase
	non-specific	269	428	8.47e-06	TIGR00152	TIGR00152	cl17190	Dephospho-CoA kinase
	non-specific	9	220	9.22e-06	TIGR01482	SPP-subfamily	cl26787	sucrose-phosphate phosphatase subfamily
	non-specific	9	220	8.38e-05	TIGR01487	Pglycolate_arch	cl26787	phosphoglycolate phosphatase, TA0175-type
	non-specific	2	92	0.000353	PRK10976	PRK10976	cl26787	putative hydrolase
	non-specific	7	49	0.000784	pfam02358	Trehalose_PPase	cl21460	Trehalose-phosphatase
	non-specific	7	48	0.001349	cd01627	HAD_TPP	cl21460	trehalose-phosphate phosphatase similar to Escherichia coli trehalose-6-phosphate phosphatase OtsB and Saccharomyces cerevisiae trehalose-phosphatase TPS2
M. dispar (HAD-DPCK)	non-specific	7	78	0.004988	COG1877	OtsB	cl28591	Trehalose-6-phosphatase [Carbohydrate transport and metabolism]

Query	Hit type	ID region start	ID region end	E-Value	Accession	Short name	Superfamily	Definition
	superfamily	7	78	0.004988	cl28591	OtsB superfamily	-	Trehalose-6-phosphatase [Carbohydrate transport and metabolism]
M. felifaucium	non-specific	1	146	3.67e-16	COG0237	CoaE	cl30785	Dephospho-CoA kinase
	superfamily	1	146	3.67e-16	cl30785	CoaE superfamily	-	Dephospho-CoA kinase
	non-specific	1	136	4.80e-15	cd02022	DPCK	cl17190	Dephospho-CoA kinase
	superfamily	1	136	4.80e-15	cl17190	NK superfamily	-	Nucleoside/nucleotide kinase superfamily
	non-specific	2	186	8.51e-11	TIGR00152	TIGR00152	cl17190	Dephospho-CoA kinase
	non-specific	2	136	2.85e-07	PRK00081	coaE	cl17190	Dephospho-CoA kinase
	non-specific	2	137	0.003639	pfam01121	CoaE	cl17190	Dephospho-CoA kinase
M. felis	non-specific	3	122	3.08e-14	cd02022	DPCK	cl17190	Dephospho-CoA kinase
	superfamily	3	122	3.08e-14	cl17190	NK superfamily	-	Nucleoside/nucleotide kinase superfamily
	non-specific	1	107	2.19e-11	COG0237	CoaE	cl30785	Dephospho-CoA kinase
	superfamily	1	107	2.19e-11	cl30785	CoaE superfamily	-	Dephospho-CoA kinase
	non-specific	4	133	3.29e-08	TIGR00152	TIGR00152	cl17190	Dephospho-CoA kinase
	non-specific	1	122	6.46e-08	PRK00081	coaE	cl17190	Dephospho-CoA kinase
	non-specific	4	107	0.001021	pfam01121	CoaE	cl17190	Dephospho-CoA kinase
	non-specific	4	107	0.002914	PRK14732	coaE	cl17190	Dephospho-CoA kinase
	non-specific	1	26	0.003529	PRK15467	PRK15467	cl21455	ethanolamine utilization protein EutP
	superfamily	1	26	0.003529	cl21455	P-loop_NTPase superfamily	-	P-loop containing Nucleoside Triphosphate Hydrolases
	non-specific	2	40	0.00391	COG1936	Fap7	cl17190	Broad-specificity NMP kinase
M. fermentans	non-specific	1	146	7.45e-23	COG0237	CoaE	cl30785	Dephospho-CoA kinase
	superfamily	1	146	7.45e-23	cl30785	CoaE superfamily	-	Dephospho-CoA kinase
	specific	1	146	2.70e-20	cd02022	DPCK	cl17190	Dephospho-CoA kinase
	superfamily	1	146	2.70e-20	cl17190	NK superfamily	-	Nucleoside/nucleotide kinase superfamily
	non-specific	2	146	1.34e-14	PRK00081	coaE	cl17190	Dephospho-CoA kinase
	non-specific	2	146	8.01e-14	TIGR00152	TIGR00152	cl17190	Dephospho-CoA kinase
	non-specific	1	187	8.48e-07	PRK14732	coaE	cl17190	Dephospho-CoA kinase
	non-specific	2	137	3.08e-06	PRK14733	coaE	cl17190	Dephospho-CoA kinase
	non-specific	2	146	3.22e-06	pfam01121	CoaE	cl17190	Dephospho-CoA kinase
M. flocculare (HAD-DPCK)	non-specific	7	258	1.96e-37	pfam08282	Hydrolase_3	cl26787	haloacid dehalogenase-like hydrolase
	superfamily	7	258	1.96e-37	cl26787	Hydrolase_3 superfamily	-	haloacid dehalogenase-like hydrolase
	specific	1	263	3.22e-34	COG0561	Cof	cl26787	Hydroxymethylpyrimidine pyrophosphatase and other HAD family phosphatases [Coenzyme transport and metabolism, General function prediction only]
	non-specific	7	260	7.20e-31	cd07516	HAD_Pase	cl21460	phosphatase, similar to Escherichia coli Cof and Thermotoga maritima TM0651
	superfamily	7	260	7.20e-31	cl21460	HAD_like superfamily	-	Haloacid Dehalogenase-like Hydrolases
	non-specific	7	257	9.73e-30	TIGR00099	Cof-subfamily	cl26787	Cof subfamily of IIB subfamily of haloacid dehalogenase superfamily
M. flocculare (HAD-DPCK)	non-specific	4	246	5.58e-17	cd07517	HAD_HPP	cl21460	phosphatase, similar to Bacteroides thetaiotaomicron VPI-5482 BT4131 hexose phosphate phosphatase

Query	Hit type	ID region start	ID region end	E-Value	Accession	Short name	Superfamily	Definition
	non-specific	268	395	1.17e-14	COG0237	CoaE	cl30785	Dephospho-CoA kinase
	superfamily	268	395	1.17e-14	cl30785	CoaE superfamily	-	Dephospho-CoA kinase
	non-specific	7	232	2.46e-12	TIGR01484	HAD-SF-IIB	cl26787	HAD-superfamily
	non-specific	1	246	5.34e-10	PRK01158	PRK01158	cl26787	phosphoglycolate phosphatase
	non-specific	268	395	1.46e-09	cd02022	DPCK	cl17190	Dephospho-CoA kinase
	superfamily	268	395	1.46e-09	cl17190	NK superfamily	-	Nucleoside/nucleotide kinase superfamily
	non-specific	9	250	4.25e-07	TIGR01482	SPP-subfamily	cl26787	sucrose-phosphate phosphatase subfamily
	non-specific	9	246	1.63e-06	TIGR01487	Pglycolate_arch	cl26787	phosphoglycolate phosphatase, TA0175-type
	non-specific	167	263	8.18e-05	PRK14502	PRK14502	cl26786	bifunctional mannosyl-3-phosphoglycerate synthase/mannosyl-3 phosphoglycerate phosphatase
	superfamily	167	263	8.18e-05	cl26786	YedP superfamily	-	Predicted mannosyl-3-phosphoglycerate phosphatase, HAD superfamily
	non-specific	1	263	0.00034	PRK10513	PRK10513	cl26787	sugar phosphate phosphatase
	non-specific	191	246	0.000356	cd07514	HAD_Pase	cl21460	phosphatase, similar to Thermoplasma acidophilum TA0175 phosphoglycolate phosphatase (PCPase), and Pyrococcus horikoshii PH1421, a magnesium-dependent phosphatase
	non-specific	268	405	0.000473	TIGR00152	TIGR00152	cl17190	Dephospho-CoA kinase
	non-specific	268	395	0.004105	PRK00081	coaE	cl17190	Dephospho-CoA kinase
	non-specific	246	285	0.004508	cd03257	ABC_NikE_OppD_transporters	cl25403	ATP-binding cassette domain of nickel/oligopeptides specific transporters
	superfamily	246	285	0.004508	cl25403	ABC_ATPase superfamily	-	ATP-binding cassette transporter nucleotide-binding domain
	non-specific	7	74	0.006724	cd01627	HAD_TPP	cl21460	trehalose-phosphate phosphatase similar to Escherichia coli trehalose-6-phosphate phosphatase OtsB and Saccharomyces cerevisiae trehalose-phosphatase TPS2
M. gallinarum	non-specific	1	145	3.41e-15	COG0237	CoaE	cl30785	Dephospho-CoA kinase
	superfamily	1	145	3.41e-15	cl30785	CoaE superfamily	-	Dephospho-CoA kinase
	non-specific	1	136	4.53e-13	cd02022	DPCK	cl17190	Dephospho-CoA kinase
	superfamily	1	136	4.53e-13	cl17190	NK superfamily	-	Nucleoside/nucleotide kinase superfamily
	non-specific	2	145	2.67e-09	TIGR00152	TIGR00152	cl17190	Dephospho-CoA kinase
	non-specific	2	151	2.01e-05	PRK00081	coaE	cl17190	Dephospho-CoA kinase
M. gallisepticum	non-specific	25	199	6.87e-19	COG0237	CoaE	cl30785	Dephospho-CoA kinase
	superfamily	25	199	6.87e-19	cl30785	CoaE superfamily	-	Dephospho-CoA kinase
	non-specific	24	194	4.11e-18	PRK00081	coaE	cl17190	Dephospho-CoA kinase
	superfamily	24	194	4.11e-18	cl17190	NK superfamily	-	Nucleoside/nucleotide kinase superfamily
	non-specific	23	186	9.11e-17	TIGR00152	TIGR00152	cl17190	Dephospho-CoA kinase
	non-specific	17	181	6.24e-12	cd02022	DPCK	cl17190	Dephospho-CoA kinase
M. gallisepticum	non-specific	23	88	5.55e-08	pfam01121	CoaE	cl17190	Dephospho-CoA kinase
	non-specific	18	195	2.61e-07	PLN02422	PLN02422	cl17190	Dephospho-CoA kinase
	non-specific	27	194	3.52e-07	PRK14732	coaE	cl17190	Dephospho-CoA kinase
M. genitalium	specific	2	182	5.10e-78	TIGR00152	TIGR00152	cl17190	Dephospho-CoA kinase
	superfamily	2	182	5.10e-78	cl17190	NK superfamily	-	Nucleoside/nucleotide kinase superfamily

Query	Hit type	ID region start	ID region end	E-Value	Accession	Short name	Superfamily	Definition
	specific	1	198	1.74e-45	COG0237	CoaE	cl30785	Dephospho-CoA kinase
	superfamily	1	198	1.74e-45	cl30785	CoaE superfamily	-	Dephospho-CoA kinase
	specific	2	169	1.84e-37	cd02022	DPCK	cl17190	Dephospho-CoA kinase
	non-specific	3	128	1.43e-18	PRK00081	coaE	cl17190	Dephospho-CoA kinase
	non-specific	3	106	8.84e-10	PRK14730	coaE	cl17190	Dephospho-CoA kinase
	non-specific	1	110	8.42e-08	pfam01121	CoaE	cl17190	Dephospho-CoA kinase
	non-specific	2	98	2.74e-05	PLN02422	PLN02422	cl17190	Dephospho-CoA kinase
	non-specific	2	114	9.20e-05	PRK14732	coaE	cl17190	Dephospho-CoA kinase
	non-specific	3	75	0.000209	pfam13521	AAA_28	cl21455	AAA domain
	superfamily	3	75	0.000209	cl21455	P-loop_NTPase superfamily	-	P-loop containing Nucleoside Triphosphate Hydrolases
	non-specific	1	172	0.000756	PTZ00451	PTZ00451	cl17190	Dephospho-CoA kinase
	non-specific	6	77	0.002063	pfam13207	AAA_17	cl21455	AAA domain
	non-specific	2	82	0.002657	PRK14731	coaE	cl17190	Dephospho-CoA kinase
	non-specific	3	50	0.003221	TIGR00231	small_GTP	cl27769	small GTP-binding protein domain
	superfamily	3	50	0.003221	cl27769	GTP_EFTU superfamily	-	Elongation factor Tu GTP binding domain
M. hyopneumoniae (HAD-DPCK)	specific	1	265	8.37e-32	COG0561	Cof	cl26787	Hydroxymethylpyrimidine pyrophosphatase and other HAD family phosphatases [Coenzyme transport and metabolism, General function prediction only]
	superfamily	1	265	8.37e-32	cl26787	Hydrolase_3 superfamily	-	haloacid dehalogenase-like hydrolase
	non-specific	7	258	1.25e-29	pfam08282	Hydrolase_3	cl26787	haloacid dehalogenase-like hydrolase
	non-specific	5	258	3.30e-27	TIGR00099	Cof-subfamily	cl26787	Cof subfamily of IIB subfamily of haloacid dehalogenase superfamily
	non-specific	7	260	9.11e-25	cd07516	HAD_Pase	cl21460	phosphatase, similar to Escherichia coli Cof and Thermotoga maritima TM0651
	superfamily	7	260	9.11e-25	cl21460	HAD_like superfamily	-	Haloacid Dehalogenase-like hydrolases
	non-specific	269	428	1.87e-13	COG0237	CoaE	cl30785	Dephospho-CoA kinase
	superfamily	269	428	1.87e-13	cl30785	CoaE superfamily	-	Dephospho-CoA kinase
	non-specific	4	246	2.03e-12	cd07517	HAD_HPP	cl21460	phosphatase, similar to Bacteroides thetaiotaomicron VPI-5482 BT4131 hexose phosphate phosphatase
	non-specific	7	221	3.80e-11	TIGR01484	HAD-SF-IIB	cl26787	HAD-superfamily
	non-specific	9	250	3.14e-09	TIGR01482	SPP-subfamily	cl26787	sucrose-phosphate phosphatase subfamily
	non-specific	269	395	1.51e-08	cd02022	DPCK	cl17190	Dephospho-CoA kinase
	superfamily	269	395	1.51e-08	cl17190	NK superfamily	-	Nucleoside/nucleotide kinase superfamily
	non-specific	1	263	3.41e-08	PRK01158	PRK01158	cl26787	phosphoglycolate phosphatase
	non-specific	269	428	2.04e-07	TIGR00152	TIGR00152	cl17190	Dephospho-CoA kinase
M. hyopneumoniae (HAD-DPCK)	non-specific	191	246	1.35e-06	cd07514	HAD_Pase	cl21460	phosphatase, similar to Thermoplasma acidophilum TA0175 phosphoglycolate phosphatase (PCPase), and Pyrococcus horikoshii PH1421, a magnesium-dependent phosphatase
	non-specific	7	215	6.16e-06	pfam02358	Trehalose_PPase	cl21460	Trehalose-phosphatase
	non-specific	9	246	3.75e-05	TIGR01487	Pglycolate_arch	cl26787	phosphoglycolate phosphatase, TA0175-type

Query	Hit type	ID region start	ID region end	E-Value	Accession	Short name	Superfamily	Definition
	non-specific	7	48	0.000556	cd01627	HAD_TPP	cl21460	trehalose-phosphate phosphatase similar to Escherichia coli trehalose-6-phosphate phosphatase OtsB and Saccharomyces cerevisiae trehalose-phosphatase TPS2
	non-specific	182	258	0.000585	PLN02887	PLN02887	cl26787	hydrolase family protein
	non-specific	183	263	0.001299	PRK10513	PRK10513	cl26787	sugar phosphate phosphatase
	non-specific	7	58	0.002792	TIGR00685	T6PP	cl21460	trehalose-phosphatase
	non-specific	183	258	0.009025	cd07518	HAD_YbiV-Like	cl21460	Escherichia coli YbiV sugar phosphatase/phosphotransferase and related proteins
M. hyorhinis	non-specific	9	141	1.14e-15	COG0237	CoaE	cl30785	Dephospho-CoA kinase
	superfamily	9	141	1.14e-15	cl30785	CoaE superfamily	-	Dephospho-CoA kinase
	non-specific	12	141	2.74e-13	cd02022	DPCK	cl17190	Dephospho-CoA kinase
	superfamily	12	141	2.74e-13	cl17190	NK superfamily	-	Nucleoside/nucleotide kinase superfamily
	non-specific	9	140	1.34e-08	TIGR00152	TIGR00152	cl17190	Dephospho-CoA kinase
	non-specific	12	141	5.89e-06	PRK00081	coaE	cl17190	Dephospho-CoA kinase
M. imitans	specific	7	196	6.69e-24	COG0237	CoaE	cl30785	Dephospho-CoA kinase
	superfamily	7	196	6.69e-24	cl30785	CoaE superfamily	-	Dephospho-CoA kinase
	non-specific	9	193	6.46e-23	TIGR00152	TIGR00152	cl17190	Dephospho-CoA kinase
	superfamily	9	193	6.46e-23	cl17190	NK superfamily	-	Nucleoside/nucleotide kinase superfamily
	specific	9	182	3.99e-20	cd02022	DPCK	cl17190	Dephospho-CoA kinase
	non-specific	12	194	1.25e-19	PRK00081	coaE	cl17190	Dephospho-CoA kinase
	non-specific	9	196	1.11e-11	PLN02422	PLN02422	cl17190	Dephospho-CoA kinase
	non-specific	8	90	3.95e-11	pfam01121	CoaE	cl17190	Dephospho-CoA kinase
	non-specific	9	97	2.22e-05	PRK14732	coaE	cl17190	Dephospho-CoA kinase
	non-specific	12	194	2.64e-05	PRK14730	coaE	cl17190	Dephospho-CoA kinase
M. iners	non-specific	1	187	2.66e-13	COG0237	CoaE	cl30785	Dephospho-CoA kinase
	superfamily	1	187	2.66e-13	cl30785	CoaE superfamily	-	Dephospho-CoA kinase
	non-specific	1	160	5.90e-12	cd02022	DPCK	cl17190	Dephospho-CoA kinase
	superfamily	1	160	5.90e-12	cl17190	NK superfamily	-	Nucleoside/nucleotide kinase superfamily
	non-specific	2	100	5.22e-07	TIGR00152	TIGR00152	cl17190	Dephospho-CoA kinase
	non-specific	1	20	4.04e-05	cd03213	ABCG_EPDR	cl25403	Eye pigment and drug resistance transporter subfamily G of the ATP-binding cassette superfamily
	superfamily	1	20	4.04e-05	cl25403	ABC_ATPase superfamily	-	ATP-binding cassette transporter nucleotide-binding domain
	non-specific	2	123	6.79e-05	PRK00081	coaE	cl17190	Dephospho-CoA kinase
M. iners	non-specific	1	19	0.000177	cd03255	ABC_MJ0796_LolCDE_FtsE	cl25403	ATP-binding cassette domain of the transporters involved in export of lipoprotein and macrolide, and cell division protein
	non-specific	1	22	0.000252	pfam00005	ABC_tran	cl21455	ABC transporter
	superfamily	1	22	0.000252	cl21455	P-loop_NTPase superfamily	-	P-loop containing Nucleoside Triphosphate Hydrolases
	non-specific	2	22	0.000398	pfam01926	MMR_HSR1	cl21455	50S ribosome-binding GTPase

Query	Hit type	ID region start	ID region end	E-Value	Accession	Short name	Superfamily	Definition
	non-specific	1	17	0.000429	COG1136	LolD	cl28181	ABC-type lipoprotein export system, ATPase component [Cell wall/membrane/envelope biogenesis]
	superfamily	1	17	0.000429	cl28181	AAA superfamily	-	ATPases associated with a variety of cellular activities
	non-specific	1	16	0.000478	cd03256	ABC_PhnC_transporter	cl25403	ATP-binding cassette domain of the binding protein-dependent phosphonate transport system
	non-specific	1	20	0.000505	PRK10771	thiQ	cl28181	thiamine transporter ATP-binding subunit
	non-specific	1	21	0.000512	cd03228	ABCC_MRP_Like	cl25403	ATP-binding cassette domain of multidrug resistance protein-like transporters
	non-specific	2	23	0.000831	PRK11174	PRK11174	cl26602	cysteine/glutathione ABC transporter membrane/ATP-binding component
	superfamily	2	23	0.000831	cl26602	SunT superfamily	-	ABC-type bacteriocin/lantibiotic exporters, contain an N-terminal double-glycine peptidase domain [Defense mechanisms]
	non-specific	2	28	0.001139	COG4987	CydC	cl26602	ABC-type transport system involved in cytochrome bd biosynthesis, fused ATPase and permease components [Energy production and conversion, Posttranslational modification, protein turnover, chaperones]
	non-specific	2	20	0.001175	COG1116	TauB	cl28181	ABC-type nitrate/sulfonate/bicarbonate transport system, ATPase component [Inorganic ion transport and metabolism]
	non-specific	1	19	0.001453	TIGR02857	CydD	cl26602	thiol reductant ABC exporter, CydD subunit
	non-specific	2	19	0.001626	COG4988	CydD	cl26602	ABC-type transport system involved in cytochrome bd biosynthesis, ATPase and permease components [Energy production and conversion, Posttranslational modification, protein turnover, chaperones]
	non-specific	2	133	0.001635	pfam01121	CoaE	cl17190	Dephospho-CoA kinase
	non-specific	1	48	0.001853	COG3638	PhnC	cl28181	ABC-type phosphate/phosphonate transport system, ATPase component [Inorganic ion transport and metabolism]
	non-specific	1	19	0.002362	PRK10535	PRK10535	cl28180	macrolide transporter ATP-binding /permease protein
	superfamily	1	19	0.002362	cl28180	PRK10535 superfamily	-	macrolide transporter ATP-binding /permease protein
	non-specific	1	21	0.002445	COG1135	AbcC	cl28181	ABC-type methionine transport system, ATPase component [Amino acid transport and metabolism]
M. iners	non-specific	1	22	0.002501	COG0488	Uup	cl28181	ATPase components of ABC transporters with duplicated ATPase domains [General function prediction only]
	non-specific	3	22	0.002813	cd00880	Era_like	cl21455	E. coli Ras-like protein (Era)-like GTPase

Query	Hit type	ID region start	ID region end	E-Value	Accession	Short name	Superfamily	Definition
	non-specific	2	28	0.003096	cd03247	ABCC_cytochrome_bd	cl25403	ATP-binding cassette domain of CydCD, subfamily C
	non-specific	3	52	0.00316	COG1119	ModF	cl28181	ABC-type molybdenum transport system, ATPase component/photorepair protein PhrA [Inorganic ion transport and metabolism]
	non-specific	2	61	0.003567	pfam01712	dNK	cl17190	Deoxynucleoside kinase
	non-specific	1	20	0.00391	TIGR00955	3a01204	cl28180	The Eye Pigment Precursor Transporter (EPP) Family protein
	non-specific	2	23	0.004193	COG3840	ThiQ	cl28181	ABC-type thiamine transport system, ATPase component [Coenzyme transport and metabolism]
	non-specific	1	19	0.004303	TIGR03608	L_ocin_972_ABC	cl28181	putative bacteriocin export ABC transporter, lactococcin 972 group
	non-specific	2	19	0.004448	PRK01889	PRK01889	cl26332	GTPase RsgA
	superfamily	2	19	0.004448	cl26332	DUF258 superfamily	-	Protein of unknown function, DUF258
	non-specific	1	20	0.004705	COG2274	SunT	cl26602	ABC-type bacteriocin/lantibiotic exporters, contain an N-terminal double-glycine peptidase domain [Defense mechanisms]
	non-specific	1	19	0.005264	TIGR02211	LolD_lipo_ex	cl28181	lipoprotein releasing system, ATP-binding protein
	non-specific	2	22	0.005457	COG0486	MnmE	cl26334	tRNA U34 5-carboxymethylaminomethyl modifying GTPase MnmE/TrmE [Translation, ribosomal structure and biogenesis]
	superfamily	2	22	0.005457	cl26334	MnmE_helical superfamily	-	MnmE helical domain
	non-specific	2	29	0.005518	PRK07261	PRK07261	cl25401	topology modulation protein
	superfamily	2	29	0.005518	cl25401	DEXDc superfamily	-	DEAD-like helicases superfamily
	non-specific	2	22	0.005561	cd04164	trmE	cl21455	trmE is a tRNA modification GTPase
	non-specific	2	20	0.005687	cd03293	ABC_NrtD_SsuB_transporters	cl25403	ATP-binding cassette domain of the nitrate and sulfonate transporters
	non-specific	1	17	0.006201	cd00267	ABC_ATPase	cl25403	ATP-binding cassette transporter nucleotide-binding domain
	non-specific	4	21	0.006699	cd03258	ABC_MetN_methionine_transporter	cl25403	ATP-binding cassette domain of methionine transporter
	non-specific	1	20	0.006877	PRK13548	hmuV	cl28181	hemin importer ATP-binding subunit
M. iners	non-specific	2	19	0.006969	cd01854	YjeQ_EngC	cl25406	Ribosomal interacting GTPase YjeQ/EngC, a circularly permuted subfamily of the Ras GTPases
	superfamily	2	19	0.006969	cl25406	YlqF_related_GTPase superfamily	-	Circularly permuted YlqF-related GTPases
	non-specific	1	17	0.00716	cd03225	ABC_cobalt_CbiO_domain1	cl25403	First domain of the ATP-binding cassette component of cobalt transport system
	non-specific	3	19	0.007294	TIGR00929	VirB4_CagE	cl26286	type IV secretion/conjugal transfer ATPase, VirB4 family
	superfamily	3	19	0.007294	cl26286	VirB4 superfamily	-	Type IV secretory pathway, VirB4 component [Intracellular trafficking, secretion, and vesicular transport]
	non-specific	1	19	0.007436	cd03229	ABC_Class3	cl25403	ATP-binding cassette domain of the binding protein-dependent transport systems

Query	Hit type	ID region start	ID region end	E-Value	Accession	Short name	Superfamily	Definition
	non-specific	2	20	0.00811	TIGR02868	CydC	cl26602	thiol reductant ABC exporter, CydC subunit
	non-specific	2	22	0.008138	PRK05291	trmE	cl26334	tRNA modification GTPase TrmE
	non-specific	1	21	0.009792	cd03251	ABCC_MsbA	cl25403	ATP-binding cassette domain of the bacterial lipid flippase and related proteins, subfamily C
M. iowae	specific	10	199	6.37e-26	COG0237	CoaE	cl30785	Dephospho-CoA kinase
	superfamily	10	199	4.84e-26	cl30785	CoaE superfamily	-	Dephospho-CoA kinase
	specific	11	189	6.65e-25	cd02022	DPCK	cl17190	Dephospho-CoA kinase
	superfamily	11	189	6.65e-25	cl17190	NK superfamily	-	Nucleoside/nucleotide kinase superfamily
	non-specific	21	201	4.13e-20	PRK00081	coaE	cl17190	Dephospho-CoA kinase
	non-specific	11	199	3.87e-18	TIGR00152	TIGR00152	cl17190	Dephospho-CoA kinase
	non-specific	11	189	1.37e-11	pfam01121	CoaE	cl17190	Dephospho-CoA kinase
	non-specific	10	199	1.22e-08	PLN02422	PLN02422	cl17190	Dephospho-CoA kinase
	non-specific	10	199	5.33e-06	PRK14730	coaE	cl17190	Dephospho-CoA kinase
	non-specific	11	203	7.07e-06	PRK14732	coaE	cl17190	Dephospho-CoA kinase
	non-specific	10	199	0.000192	PRK01184	PRK01184	cl17190	hypothetical protein
	non-specific	10	201	0.002715	PRK14734	coaE	cl17190	Dephospho-CoA kinase
M. leachii	specific	2	175	4.88e-34	cd02022	DPCK	cl17190	Dephospho-CoA kinase
	superfamily	2	175	4.88e-34	cl17190	NK superfamily	-	Nucleoside/nucleotide kinase superfamily
	non-specific	1	168	3.69e-21	PRK00081	coaE	cl17190	Dephospho-CoA kinase
	non-specific	1	155	8.39e-19	COG0237	CoaE	cl30785	Dephospho-CoA kinase
	superfamily	1	155	8.39e-19	cl30785	CoaE superfamily	-	Dephospho-CoA kinase
	non-specific	1	163	1.02e-16	pfam01121	CoaE	cl17190	Dephospho-CoA kinase
	non-specific	2	170	1.28e-13	TIGR00152	TIGR00152	cl17190	Dephospho-CoA kinase
	non-specific	2	163	1.57e-10	PRK14732	coaE	cl17190	Dephospho-CoA kinase
	non-specific	2	185	2.36e-10	PRK14730	coaE	cl17190	Dephospho-CoA kinase
	non-specific	3	144	1.59e-08	PRK14733	coaE	cl17190	Dephospho-CoA kinase
	non-specific	2	161	0.000358	PRK14731	coaE	cl17190	Dephospho-CoA kinase
	non-specific	2	168	0.000801	PLN02422	PLN02422	cl17190	Dephospho-CoA kinase
M. leonicaptivi	non-specific	1	119	5.92e-10	cd02022	DPCK	cl17190	Dephospho-CoA kinase
	superfamily	1	119	5.92e-10	cl17190	NK superfamily	-	Nucleoside/nucleotide kinase superfamily
M. leonicaptivi	non-specific	1	37	2.16e-08	COG0237	CoaE	cl30785	Dephospho-CoA kinase
	superfamily	1	37	2.16e-08	cl30785	CoaE superfamily	-	Dephospho-CoA kinase
	non-specific	2	106	4.00e-07	PRK00081	coaE	cl17190	Dephospho-CoA kinase
	non-specific	2	174	0.000202	TIGR00152	TIGR00152	cl17190	Dephospho-CoA kinase
	non-specific	2	119	0.00902	pfam01121	CoaE	cl17190	Dephospho-CoA kinase
M. lipofaciens	non-specific	1	187	2.08e-18	COG0237	CoaE	cl30785	Dephospho-CoA kinase
	superfamily	1	187	2.08e-18	cl30785	CoaE superfamily	-	Dephospho-CoA kinase
	non-specific	1	137	3.80e-13	cd02022	DPCK	cl17190	Dephospho-CoA kinase
	superfamily	1	137	3.80e-13	cl17190	NK superfamily	-	Nucleoside/nucleotide kinase superfamily
	non-specific	2	137	7.85e-12	TIGR00152	TIGR00152	cl17190	Dephospho-CoA kinase
	non-specific	11	137	2.89e-06	PRK00081	coaE	cl17190	Dephospho-CoA kinase
M. mobile	non-specific	1	147	2.34e-16	COG0237	CoaE	cl30785	Dephospho-CoA kinase
	superfamily	1	147	2.34e-16	cl30785	CoaE superfamily	-	Dephospho-CoA kinase

Query	Hit type	ID region start	ID region end	E-Value	Accession	Short name	Superfamily	Definition
	non-specific	1	147	1.90e-15	cd02022	DPCK	cl17190	Dephospho-CoA kinase
	superfamily	1	147	1.90e-15	cl17190	NK superfamily	-	Nucleoside/nucleotide kinase superfamily
	non-specific	2	172	3.00e-10	TIGR00152	TIGR00152	cl17190	Dephospho-CoA kinase
	non-specific	2	147	9.86e-09	PRK00081	coaE	cl17190	Dephospho-CoA kinase
	non-specific	1	32	0.000576	COG1936	Fap7	cl17190	Broad-specificity NMP kinase
	non-specific	2	147	0.006349	pfam01121	CoaE	cl17190	Dephospho-CoA kinase
M. molare	non-specific	1	136	4.12e-19	COG0237	CoaE	cl30785	Dephospho-CoA kinase
	superfamily	1	136	4.12e-19	cl30785	CoaE superfamily	-	Dephospho-CoA kinase
	non-specific	1	131	3.33e-14	cd02022	DPCK	cl17190	Dephospho-CoA kinase
	superfamily	1	131	3.33e-14	cl17190	NK superfamily	-	Nucleoside/nucleotide kinase superfamily
	non-specific	2	131	1.64e-09	TIGR00152	TIGR00152	cl17190	Dephospho-CoA kinase
	non-specific	2	131	1.16e-05	PRK00081	coaE	cl17190	Dephospho-CoA kinase
M. mycoides subsp. *capri*	specific	2	175	2.52e-36	cd02022	DPCK	cl17190	Dephospho-CoA kinase
	superfamily	2	175	2.52e-36	cl17190	NK superfamily	-	Nucleoside/nucleotide kinase superfamily
	non-specific	1	170	4.86e-24	PRK00081	coaE	cl17190	Dephospho-CoA kinase
	non-specific	1	175	9.60e-21	COG0237	CoaE	cl30785	Dephospho-CoA kinase
	superfamily	1	175	9.60e-21	cl30785	CoaE superfamily	-	Dephospho-CoA kinase
	non-specific	1	170	9.20e-20	pfam01121	CoaE	cl17190	Dephospho-CoA kinase
	non-specific	2	170	5.02e-15	TIGR00152	TIGR00152	cl17190	Dephospho-CoA kinase
	non-specific	2	170	3.85e-12	PRK14730	coaE	cl17190	Dephospho-CoA kinase
	non-specific	2	170	5.65e-12	PRK14732	coaE	cl17190	Dephospho-CoA kinase
	non-specific	3	170	6.95e-09	PRK14733	coaE	cl17190	Dephospho-CoA kinase
	non-specific	2	170	1.76e-06	PRK14731	coaE	cl17190	Dephospho-CoA kinase
	non-specific	2	175	3.12e-05	PLN02422	PLN02422	cl17190	Dephospho-CoA kinase
M. mycoides subsp. *mycoides*	specific	6	179	5.87e-34	cd02022	DPCK	cl17190	Dephospho-CoA kinase
	superfamily	6	179	5.87e-34	cl17190	NK superfamily	-	Nucleoside/nucleotide kinase superfamily
	non-specific	4	172	6.04e-21	PRK00081	coaE	cl17190	Dephospho-CoA kinase
	non-specific	4	159	7.48e-19	COG0237	CoaE	cl307855	Dephospho-CoA kinase
	superfamily	4	159	7.48e-19	cl30785	CoaE superfamily	-	Dephospho-CoA kinase
	non-specific	5	167	9.70e-17	pfam01121	CoaE	cl17190	Dephospho-CoA kinase
M. mycoides subsp. *mycoides*	non-specific	6	174	9.44e-14	TIGR00152	TIGR00152	cl17190	Dephospho-CoA kinase
	non-specific	6	167	9.65e-11	PRK14732	coaE	cl17190	Dephospho-CoA kinase
	non-specific	6	189	9.75e-11	PRK14730	coaE	cl17190	Dephospho-CoA kinase
	non-specific	7	148	3.64e-08	PRK14733	coaE	cl17190	Dephospho-CoA kinase
	non-specific	3	165	0.000422	PRK14731	coaE	cl17190	Dephospho-CoA kinase
	non-specific	6	172	0.001703	PLN02422	PLN02422	cl17190	Dephospho-CoA kinase
M. opalescens	non-specific	1	135	8.34e-17	COG0237	CoaE	cl30785	Dephospho-CoA kinase
	superfamily	1	135	8.34e-17	cl30785	CoaE superfamily	-	Dephospho-CoA kinase
	non-specific	1	135	3.58e-11	cd02022	DPCK	cl17190	Dephospho-CoA kinase
	superfamily	1	135	3.58e-11	cl17190	NK superfamily	-	Nucleoside/nucleotide kinase superfamily
	non-specific	2	111	7.67e-09	PRK00081	coaE	cl17190	Dephospho-CoA kinase
	non-specific	2	159	3.30e-08	TIGR00152	TIGR00152	cl17190	Dephospho-CoA kinase
	non-specific	2	129	1.34e-06	pfam01121	CoaE	cl17190	Dephospho-CoA kinase

Query	Hit type	ID region start	ID region end	E-Value	Accession	Short name	Superfamily	Definition
	non-specific	2	33	0.002374	cd01983	Fer4_NifH	cl28886	The Fer4_NifH superfamily
	superfamily	2	33	0.002374	cl28886	Fer4_NifH superfamily	-	The Fer4_NifH superfamily
	non-specific	2	21	0.003859	COG4598	HisP	cl28181	ABC-type histidine transport system, ATPase component [Amino acid transport and metabolism]
	superfamily	2	21	0.003859	cl28181	AAA superfamily	-	ATPases associated with a variety of cellular activities
	non-specific	2	135	0.007313	PRK14730	coaE	cl17190	Dephospho-CoA kinase
M. ovipneumoniae (HAD-DPCK)	non-specific	5	259	3.75e-35	TIGR00099	Cof-subfamily	cl26787	Cof subfamily of IIB subfamily of haloacid dehalogenase superfamily
	superfamily	5	259	3.75e-35	cl26787	Hydrolase_3 superfamily	-	haloacid dehalogenase-like hydrolase
	specific	1	266	3.19e-34	COG0561	Cof	cl26787	Hydroxymethylpyrimidine pyrophosphatase and other HAD family phosphatases [Coenzyme transport and metabolism, General function prediction only]
	non-specific	7	259	4.26e-34	pfam08282	Hydrolase_3	cl26787	haloacid dehalogenase-like hydrolase
	non-specific	5	261	1.17e-27	cd07516	HAD_Pase	cl21460	phosphatase, similar to Escherichia coli Cof and Thermotoga maritima TM0651
	superfamily	5	261	1.17e-27	cl21460	HAD_like superfamily	-	Haloacid Dehalogenase-like Hydrolases
	non-specific	4	262	2.92e-18	cd07517	HAD_HPP	cl21460	phosphatase, similar to Bacteroides thetaiotaomicron VPI-5482 BT4131 hexose phosphate phosphatase
	non-specific	269	394	4.40e-12	COG0237	CoaE	cl30785	Dephospho-CoA kinase
	superfamily	269	394	4.40e-12	cl30785	CoaE superfamily	-	Dephospho-CoA kinase
	non-specific	269	394	8.41e-09	cd02022	DPCK	cl17190	Dephospho-CoA kinase
	superfamily	269	394	8.41e-09	cl17190	NK superfamily	-	Nucleoside/nucleotide kinase superfamily
	non-specific	5	233	9.07e-09	TIGR01484	HAD-SF-IIB	cl26787	HAD-superfamily
	non-specific	1	262	1.87e-08	PRK01158	PRK01158	cl26787	phosphoglycolate phosphatase
	non-specific	8	251	5.77e-08	TIGR01482	SPP-subfamily	cl26787	sucrose-phosphate phosphatase subfamily
	non-specific	269	366	1.92e-07	TIGR00152	TIGR00152	cl17190	Dephospho-CoA kinase
	non-specific	1	265	1.37e-06	PRK10513	PRK10513	cl26787	sugar phosphate phosphatase
M. ovipneumoniae (HAD-DPCK)	non-specific	4	259	0.001224	TIGR01487	Pglycolate_arch	cl26787	phosphoglycolate phosphatase, TA0175-type
	non-specific	263	438	0.004457	PRK14733	coaE	cl17190	Dephospho-CoA kinase
	non-specific	7	74	0.007687	pfam02358	Trehalose_PPase	cl21460	Trehalose-phosphatase
M. penetrans	specific	14	143	1.58e-39	cd02022	DPCK	cl17190	Dephospho-CoA kinase
	superfamily	14	143	1.58e-39	cl17190	NK superfamily	-	Nucleoside/nucleotide kinase superfamily
	specific	13	142	1.53e-25	COG0237	CoaE	cl30785	Dephospho-CoA kinase
	superfamily	13	142	1.53e-25	cl30785	CoaE superfamily	-	Dephospho-CoA kinase
	non-specific	12	142	3.04e-24	PRK00081	coaE	cl17190	Dephospho-CoA kinase
	non-specific	14	143	2.66e-19	TIGR00152	TIGR00152	cl17190	Dephospho-CoA kinase
	non-specific	14	140	8.47e-15	pfam01121	CoaE	cl17190	Dephospho-CoA kinase
	non-specific	14	203	2.35e-11	PRK14731	coaE	cl17190	Dephospho-CoA kinase
	non-specific	15	142	1.05e-09	PRK14730	coaE	cl17190	Dephospho-CoA kinase
	non-specific	13	106	4.78e-09	PLN02422	PLN02422	cl17190	Dephospho-CoA kinase
	non-specific	14	203	1.78e-08	PRK14732	coaE	cl17190	Dephospho-CoA kinase

Query	Hit type	ID region start	ID region end	E-Value	Accession	Short name	Superfamily	Definition
	non-specific	11	141	5.77e-05	PRK14733	coaE	cl17190	Dephospho-CoA kinase
	non-specific	13	143	0.000212	PRK14734	coaE	cl17190	Dephospho-CoA kinase
	non-specific	15	143	0.000749	pfam13238	AAA_18	cl21455	AAA domain
	superfamily	15	143	0.000749	cl21455	P-loop_NTPase superfamily	-	P-loop containing Nucleoside Triphosphate Hydrolases
	non-specific	13	30	0.001651	PRK00349	uvrA	cl26603	excinuclease ABC subunit A
	superfamily	13	30	0.001651	cl26603	UvrA superfamily	-	Excinuclease UvrABC ATPase subunit [Replication, recombination and repair]
M. pirum	specific	4	194	9.22e-25	COG0237	CoaE	cl30785	Dephospho-CoA kinase
	superfamily	4	194	9.22e-25	cl30785	CoaE superfamily	-	Dephospho-CoA kinase
	non-specific	7	187	1.24e-22	TIGR00152	TIGR00152	cl17190	Dephospho-CoA kinase
	superfamily	7	187	1.24e-22	cl17190	NK superfamily	-	Nucleoside/nucleotide kinase superfamily
	specific	7	179	2.50e-22	cd02022	DPCK	cl17190	Dephospho-CoA kinase
	non-specific	6	192	1.75e-15	PRK00081	coaE	cl17190	Dephospho-CoA kinase
	non-specific	6	92	3.16e-09	pfam01121	CoaE	cl17190	Dephospho-CoA kinase
	non-specific	7	92	6.17e-07	PRK14730	coaE	cl17190	Dephospho-CoA kinase
	non-specific	7	92	0.000103	PRK14732	coaE	cl17190	Dephospho-CoA kinase
	non-specific	7	92	0.0002	COG1136	LolD	cl28181	ABC-type lipoprotein export system, ATPase component [Cell wall/membrane/envelope biogenesis]
	superfamily	7	92	0.0002	cl28181	AAA superfamily	-	ATPases associated with a variety of cellular activities
	non-specific	7	85	0.000317	PLN02422	PLN02422	cl17190	Dephospho-CoA kinase
	non-specific	7	25	0.000701	cd01854	YjeQ_EngC	cl25406	Ribosomal interacting GTPase YjeQ/EngC, a circularly permuted subfamily of the Ras GTPases
	superfamily	7	25	0.000701	cl25406	YlqF_related_GTPase superfamily	-	Circularly permuted YlqF-related GTPases
	non-specific	8	137	0.000793	pfam13238	AAA_18	cl21455	AAA domain
M. pirum	superfamily	8	137	0.000793	cl21455	P-loop_NTPase superfamily	-	P-loop containing Nucleoside Triphosphate Hydrolases
	non-specific	1	91	0.00103	PRK14731	coaE	cl17190	Dephospho-CoA kinase
	non-specific	6	27	0.001255	COG1936	Fap7	cl17190	Broad-specificity NMP kinase
	non-specific	6	33	0.002034	PRK04040	PRK04040	cl17190	adenylate kinase
	non-specific	7	92	0.002161	cd03255	ABC_MJ0796_LolCDE_FtsE	cl25403	ATP-binding cassette domain of the transporters involved in export of lipoprotein and macrolide, and cell division protein
	superfamily	7	92	0.002161	cl25403	ABC_ATPase superfamily	-	ATP-binding cassette transporter nucleotide-binding domain
	non-specific	1	28	0.003735	COG2019	AdkA	cl17190	Archaeal adenylate kinase
	non-specific	6	35	0.006283	PRK03839	PRK03839	cl17190	putative kinase
M. pneumoniae	specific	2	183	1.28e-81	TIGR00152	TIGR00152	cl17190	Dephospho-CoA kinase
	superfamily	2	183	1.28e-81	cl17190	NK superfamily	-	Nucleoside/nucleotide kinase superfamily
	specific	1	194	4.42e-48	COG0237	CoaE	cl30785	Dephospho-CoA kinase
	superfamily	1	194	4.42e-48	cl30785	CoaE superfamily	-	Dephospho-CoA kinase
	specific	2	156	1.15e-39	cd02022	DPCK	cl17190	Dephospho-CoA kinase

Query	Hit type	ID region start	ID region end	E-Value	Accession	Short name	Superfamily	Definition
	non-specific	3	156	1.67e-28	PRK00081	coaE	cl17190	Dephospho-CoA kinase
	non-specific	3	106	3.23e-14	PRK14730	coaE	cl17190	Dephospho-CoA kinase
	non-specific	1	156	3.67e-14	pfam01121	CoaE	cl17190	Dephospho-CoA kinase
	non-specific	2	156	6.80e-06	PRK14732	coaE	cl17190	Dephospho-CoA kinase
	non-specific	1	156	8.58e-05	PRK14734	coaE	cl17190	Dephospho-CoA kinase
	non-specific	2	185	0.000155	PRK14731	coaE	cl17190	Dephospho-CoA kinase
	non-specific	3	153	0.000692	pfam13521	AAA_28	cl21455	AAA domain
	superfamily	3	153	0.000692	cl21455	P-loop_NTPase superfamily	-	P-loop containing Nucleoside Triphosphate Hydrolases
	non-specific	1	153	0.001474	COG1428	Dck	cl17190	Deoxyadenosine/deoxycytidine kinase
	non-specific	5	90	0.003355	PLN02422	PLN02422	cl17190	Dephospho-CoA kinase
	non-specific	1	28	0.003534	PRK04182	PRK04182	cl28332	cytidylate kinase
	superfamily	1	28	0.003534	cl28332	CmkB superfamily	-	Cytidylate kinase
	non-specific	3	35	0.006008	cd01983	Fer4_NifH	cl28886	The Fer4_NifH superfamily
	superfamily	3	35	0.006008	cl28886	Fer4_NifH superfamily	-	The Fer4_NifH superfamily
	non-specific	1	37	0.009106	COG1102	CmkB	cl28332	Cytidylate kinase
M. primatum	non-specific	1	144	2.19e-18	COG0237	CoaE	cl30785	Dephospho-CoA kinase
	superfamily	1	144	2.19e-18	cl30785	CoaE superfamily	-	Dephospho-CoA kinase
	non-specific	1	148	2.85e-15	cd02022	DPCK	cl17190	Dephospho-CoA kinase
	superfamily	1	148	2.85e-15	cl17190	NK superfamily	-	Nucleoside/nucleotide kinase superfamily
	non-specific	2	137	4.48e-10	TIGR00152	TIGR00152	cl17190	Dephospho-CoA kinase
	non-specific	2	148	2.85e-05	PRK00081	coaE	cl17190	Dephospho-CoA kinase
M. pulmonis	specific	1	172	1.38e-34	COG0237	CoaE	cl30785	Dephospho-CoA kinase
	superfamily	1	172	1.38e-34	cl30785	CoaE superfamily	-	Dephospho-CoA kinase
	specific	1	170	1.51e-29	cd02022	DPCK	cl17190	Dephospho-CoA kinase
	superfamily	1	170	1.51e-29	cl17190	NK superfamily	-	Nucleoside/nucleotide kinase superfamily
M. pulmonis	non-specific	2	137	2.64e-20	TIGR00152	TIGR00152	cl17190	Dephospho-CoA kinase
	non-specific	2	136	6.79e-12	PRK00081	coaE	cl17190	Dephospho-CoA kinase
	non-specific	2	143	9.80e-06	pfam01121	CoaE	cl17190	Dephospho-CoA kinase
	non-specific	2	143	3.27e-05	pfam13238	AAA_18	cl21455	AAA domain
	superfamily	2	143	3.27e-05	cl21455	P-loop_NTPase superfamily	-	P-loop containing Nucleoside Triphosphate Hydrolases
	non-specific	1	78	0.000167	COG1936	Fap7	cl17190	Broad-specificity NMP kinase
	non-specific	2	27	0.0002	cd02034	CooC	cl28886	The accessory protein CooC, which contains a nucleotide-binding domain (P-loop) near the N-terminus, participates in the maturation of the nickel center of carbon monoxide dehydrogenase (CODH).
	superfamily	2	27	0.0002	cl28886	Fer4_NifH superfamily	-	The Fer4_NifH superfamily
	non-specific	2	140	0.001408	PRK14733	coaE	cl17190	Dephospho-CoA kinase
	non-specific	2	171	0.002499	COG1100	Gem1	cl27030	GTPase SAR1 family domain [General function prediction only]
	superfamily	2	171	0.002499	cl27030	Ras superfamily	-	Ras family
	non-specific	3	157	0.002851	TIGR01054	rgy	cl27598	reverse gyrase

Query	Hit type	ID region start	ID region end	E-Value	Accession	Short name	Superfamily	Definition
	superfamily	3	157	0.002851	cl27598	TOP1Bc superfamily	-	Bacterial DNA topoisomeraes I ATP-binding domain
	non-specific	2	146	0.003434	COG1102	CmkB	cl28332	Cytidylate kinase
	superfamily	2	146	0.003434	cl28332	CmkB superfamily	-	Cytidylate kinase
	non-specific	2	30	0.004023	COG3640	CooC	cl27521	CO dehydrogenase nickel-insertion accessory protein CooC1 [Posttranslational modification, protein turnover, chaperones]
	superfamily	2	30	0.004023	cl27521	CbiA superfamily	-	CobQ/CobB/MinD/ParA nucleotide binding domain
	non-specific	2	27	0.005486	cd01983	Fer4_NifH	cl28886	The Fer4_NifH superfamily
M. putrefaciens	specific	2	168	2.03e-25	cd02022	DPCK	cl17190	Dephospho-CoA kinase
	superfamily	2	168	2.03e-25	cl17190	NK superfamily	-	Nucleoside/nucleotide kinase superfamily
	non-specific	1	144	6.61e-20	PRK00081	coaE	cl17190	Dephospho-CoA kinase
	non-specific	1	144	1.37e-19	pfam01121	CoaE	cl17190	Dephospho-CoA kinase
	non-specific	1	144	7.97e-17	COG0237	CoaE	cl30785	Dephospho-CoA kinase
	superfamily	1	144	7.97e-17	cl30785	CoaE superfamily	-	Dephospho-CoA kinase
	non-specific	2	162	1.78e-15	TIGR00152	TIGR00152	cl17190	Dephospho-CoA kinase
	non-specific	2	145	1.48e-08	PRK14730	coaE	cl17190	Dephospho-CoA kinase
	non-specific	2	166	8.52e-08	PRK14731	coaE	cl17190	Dephospho-CoA kinase
	non-specific	3	141	2.13e-06	PRK14733	coaE	cl17190	Dephospho-CoA kinase
	non-specific	3	93	2.83e-05	PRK14734	coaE	cl17190	Dephospho-CoA kinase
	non-specific	2	163	0.000238	PRK14732	coaE	cl17190	Dephospho-CoA kinase
	non-specific	1	150	0.000584	TIGR02173	cyt_kin_arch	cl28332	cytidylate kinase
	superfamily	1	150	0.000584	cl28332	CmkB superfamily	-	Cytidylate kinase
	non-specific	1	56	0.000825	COG1102	CmkB	cl28332	Cytidylate kinase
	non-specific	2	94	0.003407	PLN02422	PLN02422	cl17190	Dephospho-CoA kinase
M. putrefaciens	non-specific	10	86	0.003775	pfam13207	AAA_17	cl21455	AAA domain
	superfamily	10	86	0.003775	cl21455	P-loop_NTPase superfamily	-	P-loop containing Nucleoside Triphosphate Hydrolases
	non-specific	2	141	0.005687	cd02023	UMPK	cl17190	Uridine monophosphate kinase
	non-specific	2	141	0.006565	TIGR00235	udk	cl17190	Uridine kinase superfamily
M. simbae	non-specific	1	137	5.63e-19	COG0237	CoaE	cl30785	Dephospho-CoA kinase
	superfamily	1	137	5.63e-19	cl30785	CoaE superfamily	-	Dephospho-CoA kinase
	non-specific	1	137	2.29e-16	cd02022	DPCK	cl17190	Dephospho-CoA kinase
	superfamily	1	137	2.29e-16	cl17190	NK superfamily	-	Nucleoside/nucleotide kinase superfamily
	non-specific	2	146	5.49e-13	TIGR00152	TIGR00152	cl17190	Dephospho-CoA kinase
	non-specific	2	137	1.57e-09	PRK00081	coaE	cl17190	Dephospho-CoA kinase
	non-specific	2	137	5.44e-05	pfam01121	CoaE	cl17190	Dephospho-CoA kinase
	non-specific	2	51	0.002071	TIGR03375	type_I_sec_LssB	cl26602	type I secretion system ATPase, LssB family
	superfamily	2	51	0.002071	cl26602	SunT superfamily	-	ABC-type bacteriocin/lantibiotic exporters, contain an N-terminal double-glycine peptidase domain [Defense mechanisms]

Query	Hit type	ID region start	ID region end	E-Value	Accession	Short name	Superfamily	Definition
	non-specific	1	17	0.007807	COG0488	Uup	cl28181	ATPase components of ABC transporters with duplicated ATPase domains [General function prediction only]
	superfamily	1	17	0.007807	cl28181	AAA superfamily	-	ATPases associated with a variety of cellular activities
M. sturni	non-specific	1	187	8.24e-10	COG0237	CoaE	cl30785	Dephospho-CoA kinase
	superfamily	1	187	8.24e-10	cl30785	CoaE superfamily	-	Dephospho-CoA kinase
	non-specific	1	129	6.98e-09	cd02022	DPCK	cl17190	Dephospho-CoA kinase
	superfamily	1	129	6.98e-09	cl17190	NK superfamily	-	Nucleoside/nucleotide kinase superfamily
	non-specific	2	110	5.18e-05	PRK00081	coaE	cl17190	Dephospho-CoA kinase
	non-specific	2	128	0.000306	TIGR00152	TIGR00152	cl17190	Dephospho-CoA kinase
M. synoviae	non-specific	1	141	1.74e-12	COG0237	CoaE	cl30785	Dephospho-CoA kinase
	superfamily	1	141	1.74e-12	cl30785	CoaE superfamily	-	Dephospho-CoA kinase
	non-specific	1	147	2.92e-11	cd02022	DPCK	cl17190	Dephospho-CoA kinase
	superfamily	1	147	2.92e-11	cl17190	NK superfamily	-	Nucleoside/nucleotide kinase superfamily
	non-specific	2	166	1.06e-06	TIGR00152	TIGR00152	cl17190	Dephospho-CoA kinase
	non-specific	1	76	0.006477	pfam05272	VirE	cl23993	Virulence-associated protein E (these proteins contain a P-loop motif)
	superfamily	1	76	0.006477	cl23993	VirE superfamily	-	Virulence-associated protein E (these proteins contain a P-loop motif)
M. testudinis	specific	2	175	2.12e-33	cd02022	DPCK	cl17190	Dephospho-CoA kinase
	superfamily	2	175	2.12e-33	cl17190	NK superfamily	-	Nucleoside/nucleotide kinase superfamily
	non-specific	5	188	4.62e-32	PRK00081	coaE	cl17190	Dephospho-CoA kinase
	specific	1	193	1.37e-31	COG0237	CoaE	cl30785	Dephospho-CoA kinase
	superfamily	1	193	1.37e-31	cl30785	CoaE superfamily	-	Dephospho-CoA kinase
	non-specific	2	143	9.59e-29	TIGR00152	TIGR00152	cl17190	Dephospho-CoA kinase
	non-specific	1	89	3.48e-16	pfam01121	CoaE	cl17190	Dephospho-CoA kinase
M. testudinis	non-specific	2	191	6.50e-13	PRK14732	coaE	cl17190	Dephospho-CoA kinase
	non-specific	5	175	1.07e-11	PLN02422	PLN02422	cl17190	Dephospho-CoA kinase
	non-specific	2	187	4.17e-11	PRK14731	coaE	cl17190	Dephospho-CoA kinase
	non-specific	7	89	8.21e-08	PRK14730	coaE	cl17190	Dephospho-CoA kinase
	non-specific	1	89	3.35e-06	PRK03333	coaE	cl30785	Dephospho-CoA kinase
	non-specific	1	89	0.00045	PRK14734	coaE	cl17190	Dephospho-CoA kinase
	non-specific	2	15	0.001737	PRK00349	uvrA	cl26603	excinuclease ABC subunit A
	superfamily	2	15	0.001737	cl26603	UvrA superfamily	-	Excinuclease UvrABC ATPase subunit [Replication, recombination and repair]
	non-specific	1	175	0.002035	PTZ00451	PTZ00451	cl17190	Dephospho-CoA kinase
	non-specific	2	19	0.003434	PRK05416	PRK05416	cl23728	glmZ(sRNA)-inactivating NTPase
	superfamily	2	19	0.003434	cl23728	ATP_bind_2 superfamily	-	P-loop ATPase protein family
	non-specific	2	21	0.007101	COG0178	UvrA	cl26603	Excinuclease UvrABC ATPase subunit [Replication, recombination and repair]
M. yeatsii	specific	2	169	2.21e-25	cd02022	DPCK	cl17190	Dephospho-CoA kinase
	superfamily	2	169	2.21e-25	cl17190	NK superfamily	-	Nucleoside/nucleotide kinase superfamily

Query	Hit type	ID region start	ID region end	E-Value	Accession	Short name	Superfamily	Definition
	non-specific	1	145	5.32e-18	PRK00081	coaE	cl17190	Dephospho-CoA kinase
	non-specific	1	99	6.31e-18	pfam01121	CoaE	cl17190	Dephospho-CoA kinase
	non-specific	1	176	6.42e-17	COG0237	CoaE	cl30785	Dephospho-CoA kinase
	superfamily	1	176	6.42e-17	cl30785	CoaE superfamily	-	Dephospho-CoA kinase
	non-specific	2	163	9.11e-14	TIGR00152	TIGR00152	cl17190	Dephospho-CoA kinase
	non-specific	3	93	1.16e-08	PRK14734	coaE	cl17190	Dephospho-CoA kinase
	non-specific	2	99	3.34e-08	PRK14730	coaE	cl17190	Dephospho-CoA kinase
	non-specific	2	98	2.12e-07	PLN02422	PLN02422	cl17190	Dephospho-CoA kinase
	non-specific	3	143	4.58e-07	PRK14733	coaE	cl17190	Dephospho-CoA kinase
	non-specific	2	183	3.61e-06	PRK14732	coaE	cl17190	Dephospho-CoA kinase
	non-specific	3	77	0.004789	PRK03333	coaE	cl30785	Dephospho-CoA kinase

Supplementary Table 14 DPCK InterPro results

Mycoplasma species	Amino acid region	Database	Database ID	Database signature description	ID region start	ID region end	InterPro ID	ID type	Gene Ontology (GO) term
M. sp. Ms02	192	Gene3D	G3DSA:3.40.50.300		1	144			
		Pfam	PF01121	Dephospho-CoA kinase	1	127	IPR001977	F	GO:0004140; GO:0005524; GO:0015937
		SUPERFAMILY	SSF52540		2	106	IPR027417	H	
M. agalactiae	190	CDD	cd02022	DPCK	1	143	IPR001977	F	GO:0004140; GO:0005524; GO:0015937
		Gene3D	G3DSA:3.40.50.300		1	163			
		PANTHER	PTHR10695		2	136			
		PANTHER	PTHR10695:SF26		2	136			
		Pfam	PF01121	Dephospho-CoA kinase	1	140	IPR001977	F	GO:0004140; GO:0005524; GO:0015937
		SUPERFAMILY	SSF52540		2	137	IPR027417	H	
		TIGRFAM	TIGR00152	TIGR00152: dephospho-CoA kinase	1	141	IPR001977	F	GO:0004140; GO:0005524; GO:0015937
M. alligatoris	185	Gene3D	G3DSA:3.40.50.300		1	183			
		SUPERFAMILY	SSF52540		2	139	IPR027417	H	
M. alvi	196	CDD	cd02022	DPCK	2	151	IPR001977	F	GO:0004140; GO:0005524; GO:0015937
		Gene3D	G3DSA:3.40.50.300		1	196			
		PANTHER	PTHR10695:SF35		2	186			

Mycoplasma species	Amino acid region	Database	Database ID	Database signature description	ID region start	ID region end	InterPro ID	ID type	Gene Ontology (GO) term
		PANTHER	PTHR10695		2	186			
		Pfam	PF01121	Dephospho-CoA kinase	1	107	IPR001977	F	GO:0004140; GO:0005524; GO:0015937
		ProSiteProfiles	PS51219	Dephospho-CoA kinase (DPCK) domain profile.	2	196	IPR001977	F	GO:0004140; GO:0005524; GO:0015937
		SUPERFAMILY	SSF52540		1	190	IPR027417	H	
		TIGRFAM	TIGR00152	TIGR00152: dephospho-CoA kinase	2	185	IPR001977	F	GO:0004140; GO:0005524; GO:0015937
M. anatis	190	Gene3D	G3DSA:3.40.50.300		1	187			
		Pfam	PF01121	Dephospho-CoA kinase	1	141	IPR001977	F	GO:0004140; GO:0005524; GO:0015937
		SUPERFAMILY	SSF52540		2	137	IPR027417	H	
M. arginini	168	Gene3D	G3DSA:3.40.50.300		1	167			
		Pfam	PF01121	Dephospho-CoA kinase	2	140	IPR001977	F	GO:0004140; GO:0005524; GO:0015937
		SUPERFAMILY	SSF52540		2	124	IPR027417	H	
M. bovigenitalium	190	Gene3D	G3DSA:3.40.50.300		1	190			
		Pfam	PF01121	Dephospho-CoA kinase	2	137	IPR001977	F	GO:0004140; GO:0005524; GO:0015937
		SUPERFAMILY	SSF52540		2	181	IPR027417	H	
M. bovis	190	CDD	cd02022	DPCK	1	146	IPR001977	F	GO:0004140; GO:0005524; GO:0015937
		Gene3D	G3DSA:3.40.50.300		1	159			
		Pfam	PF01121	Dephospho-CoA kinase	1	144	IPR001977	F	GO:0004140; GO:0005524; GO:0015937
		SUPERFAMILY	SSF52540		2	137	IPR027417	H	
M. bovoculi (HAD-DPCK)	444	Coils	Coil		398	418			
		Gene3D	G3DSA:3.40.50.1000		1	261	IPR023214	H	
		Gene3D	G3DSA:3.40.50.300		262	435			
		PANTHER	PTHR10000		1	263			
		PANTHER	PTHR10000		312	406			
		PANTHER	PTHR10000:SF47		1	263			
		PANTHER	PTHR10000:SF47		312	406			
		Pfam	PF08282	haloacid dehalogenase-like hydrolase	7	254			

Mycoplasma species	Amino acid region	Database	Database ID	Database signature description	ID region start	ID region end	InterPro ID	ID type	Gene Ontology (GO) term
		Pfam	PF01121	Dephospho-CoA kinase	266	399	IPR001977	F	GO:0004140; GO:0005524; GO:0015937
		SUPERFAMILY	SSF52540		264	420	IPR027417	H	
		SUPERFAMILY	SSF56784		1	262	IPR036412	H	
		TIGRFAM	TIGR01484	HAD-SF-IIB: HAD hydrolase, family IIB	7	218	IPR006379	F	
M. buteonis	189	Gene3D	G3DSA:3.40.50.300		1	185			
		SUPERFAMILY	SSF52540		2	179	IPR027417	H	
M. californicum	190	Gene3D	G3DSA:3.40.50.300		15	188			
		Gene3D	G3DSA:3.40.50.300		1	14			
		Pfam	PF01121	Dephospho-CoA kinase	1	146	IPR001977	F	GO:0004140; GO:0005524; GO:0015937
		SUPERFAMILY	SSF52540		2	173	IPR027417	H	
M. canis	189	Gene3D	G3DSA:3.40.50.300		1	178			
		SUPERFAMILY	SSF52540		2	141	IPR027417	H	
M. capricolum subsp. *capricolum*	188	CDD	cd02022	DPCK	6	179	IPR001977	F	GO:0004140; GO:0005524; GO:0015937
		Gene3D	G3DSA:3.40.50.300		2	183			
		Hamap	MF_00376	Dephospho-CoA kinase [coaE].	4	188	IPR001977	F	GO:0004140; GO:0005524; GO:0015937
		PANTHER	PTHR10695		5	174			
		PANTHER	PTHR10695:SF26		5	174			
		Pfam	PF01121	Dephospho-CoA kinase	5	174	IPR001977	F	GO:0004140; GO:0005524; GO:0015937
		ProSiteProfiles	PS51219	Dephospho-CoA kinase (DPCK) domain profile.	6	188	IPR001977	F	GO:0004140; GO:0005524; GO:0015937
		SUPERFAMILY	SSF52540		4	174	IPR027417	H	
		TIGRFAM	TIGR00152	TIGR00152: dephospho-CoA kinase	6	178	IPR001977	F	GO:0004140; GO:0005524; GO:0015937
M. capricolum subsp. *capripneumoniae*	184	CDD	cd02022	DPCK	2	175	IPR001977	F	GO:0004140; GO:0005524; GO:0015937
		Gene3D	G3DSA:3.40.50.300		1	179			
		PANTHER	PTHR10695:SF26		2	170			
		PANTHER	PTHR10695		2	170			
		Pfam	PF01121	Dephospho-CoA kinase	1	170	IPR001977	F	GO:0004140; GO:0005524; GO:0015937

Mycoplasma species	Amino acid region	Database	Database ID	Database signature description	ID region start	ID region end	InterPro ID	ID type	Gene Ontology (GO) term
		ProSiteProfiles	PS51219	Dephospho-CoA kinase (DPCK) domain profile.	2	184	IPR001977	F	GO:0004140; GO:0005524; GO:0015937
		SUPERFAMILY	SSF52540		1	170	IPR027417	H	
		TIGRFAM	TIGR00152	TIGR00152: dephospho-CoA kinase	2	174	IPR001977	F	GO:0004140; GO:0005524; GO:0015937
M. collis	182	CDD	cd02022	DPCK	1	148	IPR001977	F	GO:0004140; GO:0005524; GO:0015937
		Gene3D	G3DSA:3.40.50.300		1	155			
		PANTHER	PTHR10695		2	131			
		PANTHER	PTHR10695:SF26		2	131			
		Pfam	PF01121	Dephospho-CoA kinase	1	100	IPR001977	F	GO:0004140; GO:0005524; GO:0015937
M. collis	182	ProSiteProfiles	PS51219	Dephospho-CoA kinase (DPCK) domain profile.	1	92	IPR001977	F	GO:0004140; GO:0005524; GO:0015937
		SUPERFAMILY	SSF52540		2	136	IPR027417	H	
		TIGRFAM	TIGR00152	TIGR00152: dephospho-CoA kinase	1	150	IPR001977	F	GO:0004140; GO:0005524; GO:0015937
M. columbinum	190	CDD	cd02022	DPCK	1	137	IPR001977	F	GO:0004140; GO:0005524; GO:0015937
		Gene3D	G3DSA:3.40.50.300		1	183			
		Pfam	PF01121	Dephospho-CoA kinase	2	138	IPR001977	F	GO:0004140; GO:0005524; GO:0015937
		SUPERFAMILY	SSF52540		2	137	IPR027417	H	
		TIGRFAM	TIGR00152	TIGR00152: dephospho-CoA kinase	1	144	IPR001977	F	GO:0004140; GO:0005524; GO:0015937
M. columborale	185	Gene3D	G3DSA:3.40.50.300		1	181			
		Pfam	PF01121	Dephospho-CoA kinase	2	135	IPR001977	F	GO:0004140; GO:0005524; GO:0015937
		SUPERFAMILY	SSF52540		1	105	IPR027417	H	
M. conjunctivae (HAD-DPCK)	445	Gene3D	G3DSA:3.40.50.1000		1	266	IPR023214	H	
		Gene3D	G3DSA:3.40.50.300		267	439			
		PANTHER	PTHR10000:SF47		341	412			
		PANTHER	PTHR10000:SF47		4	262			
		PANTHER	PTHR10000		341	412			

Mycoplasma species	Amino acid region	Database	Database ID	Database signature description	ID region start	ID region end	InterPro ID	ID type	Gene Ontology (GO) term
		PANTHER	PTHR10000		4	262			
		Pfam	PF08282	haloacid dehalogenase-like hydrolase	7	257			
		Pfam	PF01121	Dephospho-CoA kinase	267	406	IPR001977	F	GO:0004140; GO:0005524; GO:0015937
		SUPERFAMILY	SSF52540		266	401	IPR027417	H	
		SUPERFAMILY	SSF56784		1	261	IPR036412	H	
		TIGRFAM	TIGR01484	HAD-SF-IIB: HAD hydrolase, family IIB	6	220	IPR006379	F	
M. cricetuli	191	Gene3D	G3DSA:3.40.50.300		1	186			
		SUPERFAMILY	SSF52540		2	149	IPR027417	H	
M. crocodyli	190	Gene3D	G3DSA:3.40.50.300		1	190			
		Pfam	PF01121	Dephospho-CoA kinase	1	157	IPR001977	F	GO:0004140; GO:0005524; GO:0015937
M. crocodyli	190	ProSiteProfiles	PS51219	Dephospho-CoA kinase (DPCK) domain profile.	1	190	IPR001977	F	GO:0004140; GO:0005524; GO:0015937
		SUPERFAMILY	SSF52540		2	149	IPR027417	H	
M. dispar (HAD-DPCK)	447	Gene3D	G3DSA:3.40.50.1000		2	267	IPR023214	H	
		Gene3D	G3DSA:3.40.50.300		268	431			
		PANTHER	PTHR10000		357	403			
		PANTHER	PTHR10000		1	267			
		PANTHER	PTHR10000:SF47		357	403			
		PANTHER	PTHR10000:SF47		1	267			
		Pfam	PF08282	haloacid dehalogenase-like hydrolase	7	258			
		Pfam	PF01121	Dephospho-CoA kinase	269	399	IPR001977	F	GO:0004140; GO:0005524; GO:0015937
		SUPERFAMILY	SSF52540		266	401	IPR027417	H	
		SUPERFAMILY	SSF56784		3	263	IPR036412	H	
		TIGRFAM	TIGR01484	HAD-SF-IIB: HAD hydrolase, family IIB	7	230	IPR006379	F	
M. felifaucium	190	Gene3D	G3DSA:3.40.50.300		1	187			
		Pfam	PF01121	Dephospho-CoA kinase	1	137	IPR001977	F	GO:0004140; GO:0005524; GO:0015937
		SUPERFAMILY	SSF52540		2	180	IPR027417	H	
M. felis	184	Gene3D	G3DSA:3.40.50.300		1	181			
		Pfam	PF01121	Dephospho-CoA kinase	4	118	IPR001977	F	GO:0004140; GO:0005524; GO:0015937
		SUPERFAMILY	SSF52540		1	133	IPR027417	H	
M. fermentans	190	CDD	cd02022	DPCK	1	146	IPR001977	F	GO:0004140; GO:0005524; GO:0015937

Mycoplasma species	Amino acid region	Database	Database ID	Database signature description	ID region start	ID region end	InterPro ID	ID type	Gene Ontology (GO) term
		Gene3D	G3DSA:3.40.50.300		1	181			
		PANTHER	PTHR10695		2	143			
		PANTHER	PTHR10695:SF26		2	143			
		Pfam	PF01121	Dephospho-CoA kinase	1	146	IPR001977	F	GO:0004140; GO:0005524; GO:0015937
		ProSiteProfiles	PS51219	Dephospho-CoA kinase (DPCK) domain profile.	1	190	IPR001977	F	GO:0004140; GO:0005524; GO:0015937
		SUPERFAMILY	SSF52540		2	142	IPR027417	H	
		TIGRFAM	TIGR00152	TIGR00152: dephospho-CoA kinase	1	146	IPR001977	F	GO:0004140; GO:0005524; GO:0015937
M. flocculare (HAD-DPCK)	447	Gene3D	G3DSA:3.40.50.300		266	439			
		Gene3D	G3DSA:3.40.50.1000		2	251	IPR023214	H	
		PANTHER	PTHR10000		1	264			
		PANTHER	PTHR10000		364	382			
		PANTHER	PTHR10000:SF47		1	264			
		PANTHER	PTHR10000:SF47		364	382			
		Pfam	PF08282	haloacid dehalogenase-like hydrolase	7	257			
		SUPERFAMILY	SSF56784		3	263	IPR036412	H	
		SUPERFAMILY	SSF52540		268	402	IPR027417	H	
		TIGRFAM	TIGR01484	HAD-SF-IIB: HAD hydrolase, family IIB	7	229	IPR006379	F	
M. gallinarum	189	Gene3D	G3DSA:3.40.50.300		1	175			
		Pfam	PF01121	Dephospho-CoA kinase	1	151	IPR001977	F	GO:0004140; GO:0005524; GO:0015937
		SUPERFAMILY	SSF52540		2	141	IPR027417	H	
M. gallisepticum	200	Gene3D	G3DSA:3.40.50.300		1	199			
		Hamap	MF_00376	Dephospho-CoA kinase [coaE].	7	197	IPR001977	F	GO:0004140; GO:0005524; GO:0015937
		PANTHER	PTHR10695:SF26		8	190			
		PANTHER	PTHR10695		8	190			
		Pfam	PF01121	Dephospho-CoA kinase	9	90	IPR001977	F	GO:0004140; GO:0005524; GO:0015937
		ProSiteProfiles	PS51219	Dephospho-CoA kinase (DPCK) domain profile.	9	200	IPR001977	F	GO:0004140; GO:0005524; GO:0015937
		SignalP_GRAM_POSITIVE	SignalP-TM		1	12			
		SUPERFAMILY	SSF52540		8	195	IPR027417	H	

Mycoplasma species	Amino acid region	Database	Database ID	Database signature description	ID region start	ID region end	InterPro ID	ID type	Gene Ontology (GO) term
		TIGRFAM	TIGR00152	TIGR00152: dephospho-CoA kinase	9	188	IPR001977	F	GO:0004140; GO:0005524; GO:0015937
M. genitalium	198	CDD	cd02022	DPCK	2	169	IPR001977	F	GO:0004140; GO:0005524; GO:0015937
		Gene3D	G3DSA:3.40.50.300		1	189			
		Hamap	MF_00376	Dephospho-CoA kinase [coaE].	1	188	IPR001977	F	GO:0004140; GO:0005524; GO:0015937
		PANTHER	PTHR10695:SF26		2	132			
		PANTHER	PTHR10695		2	132			
M. genitalium	198	Pfam	PF01121	Dephospho-CoA kinase	1	155	IPR001977	F	GO:0004140; GO:0005524; GO:0015937
		ProSiteProfiles	PS51219	Dephospho-CoA kinase (DPCK) domain profile.	2	90	IPR001977	F	GO:0004140; GO:0005524; GO:0015937
		SUPERFAMILY	SSF52540		1	186	IPR027417	H	
		TIGRFAM	TIGR00152	TIGR00152: dephospho-CoA kinase	2	180	IPR001977	F	GO:0004140; GO:0005524; GO:0015937
M. hyopneumoniae (HAD-DPCK)	446	Gene3D	G3DSA:3.40.50.300		269	429			
		Gene3D	G3DSA:3.40.50.1000		1	249	IPR023214	H	
		PANTHER	PTHR10000		1	266			
		PANTHER	PTHR10000		341	354			
		PANTHER	PTHR10000:SF47		1	266			
		PANTHER	PTHR10000:SF47		341	354			
		Pfam	PF08282	haloacid dehalogenase-like hydrolase	7	258			
		Pfam	PF01121	Dephospho-CoA kinase	270	428	IPR001977	F	GO:0004140; GO:0005524; GO:0015937
		SUPERFAMILY	SSF56784		2	263	IPR036412	H	
		SUPERFAMILY	SSF52540		269	402	IPR027417	H	
		TIGRFAM	TIGR01484	HAD-SF-IIB: HAD hydrolase, family IIB	7	227	IPR006379	F	
M. hyorhinis	195	Gene3D	G3DSA:3.40.50.300		1	184			
		Pfam	PF01121	Dephospho-CoA kinase	12	154	IPR001977	F	GO:0004140; GO:0005524; GO:0015937
		SUPERFAMILY	SSF52540		5	158	IPR027417	H	
M. imitans	196	CDD	cd02022	DPCK	9	182	IPR001977	F	GO:0004140; GO:0005524; GO:0015937
		Gene3D	G3DSA:3.40.50.300		1	196			

Mycoplasma species	Amino acid region	Database	Database ID	Database signature description	ID region start	ID region end	InterPro ID	ID type	Gene Ontology (GO) term
		PANTHER	PTHR10695:SF26		7	190			
		PANTHER	PTHR10695		7	190			
		Pfam	PF01121	Dephospho-CoA kinase	9	100	IPR001977	F	GO:0004140; GO:0005524; GO:0015937
		Phobius	SIGNAL_PEPTIDE_C_REGION	C-terminal region of a signal peptide.	14	18			
		Phobius	SIGNAL_PEPTIDE	Signal peptide region	1	18			
		Phobius	NON_CYTOPLASMIC_DOMAIN	Region of a membrane-bound protein predicted to be outside the membrane, in the extracellular region.	19	196			
		Phobius	SIGNAL_PEPTIDE_N_REGION	N-terminal region of a signal peptide.	1	7			
		Phobius	SIGNAL_PEPTIDE_H_REGION	Hydrophobic region of a signal peptide.	8	13			
M. imitans	196	ProSiteProfiles	PS51219	Dephospho-CoA kinase (DPCK) domain profile.	9	196	IPR001977	F	GO:0004140; GO:0005524; GO:0015937
		SUPERFAMILY	SSF52540		8	194	IPR027417	H	
		TIGRFAM	TIGR00152	TIGR00152: dephospho-CoA kinase	9	189	IPR001977	F	GO:0004140; GO:0005524; GO:0015937
M. iners	190	Gene3D	G3DSA:3.40.50.300		1	187			
		Pfam	PF01121	Dephospho-CoA kinase	2	140	IPR001977	F	GO:0004140; GO:0005524; GO:0015937
		SUPERFAMILY	SSF52540		2	137	IPR027417	H	
M. iowae	203	CDD	cd02022	DPCK	11	189	IPR001977	F	GO:0004140; GO:0005524; GO:0015937
		Gene3D	G3DSA:3.40.50.300		1	203			
		Hamap	MF_00376	Dephospho-CoA kinase [coaE].	9	200	IPR001977	F	GO:0004140; GO:0005524; GO:0015937
		PANTHER	PTHR10695		11	199			
		PANTHER	PTHR10695:SF35		11	199			
		Pfam	PF01121	Dephospho-CoA kinase	11	189	IPR001977	F	GO:0004140; GO:0005524; GO:0015937
		ProSiteProfiles	PS51219	Dephospho-CoA kinase (DPCK) domain profile.	11	203	IPR001977	F	GO:0004140; GO:0005524; GO:0015937
		SUPERFAMILY	SSF52540		8	200	IPR027417	H	
		TIGRFAM	TIGR00152	TIGR00152: dephospho-CoA kinase	11	195	IPR001977	F	GO:0004140; GO:0005524; GO:0015937

Mycoplasma species	Amino acid region	Database	Database ID	Database signature description	ID region start	ID region end	InterPro ID	ID type	Gene Ontology (GO) term
M. leachii	185	CDD	cd02022	DPCK	2	175	IPR001977	F	GO:0004140; GO:0005524; GO:0015937
		Gene3D	G3DSA:3.40.50.300		1	181			
		PANTHER	PTHR10695:SF26		2	159			
		PANTHER	PTHR10695		2	159			
		Pfam	PF01121	Dephospho-CoA kinase	1	163	IPR001977	F	GO:0004140; GO:0005524; GO:0015937
		ProSiteProfiles	PS51219	Dephospho-CoA kinase (DPCK) domain profile.	2	185	IPR001977	F	GO:0004140; GO:0005524; GO:0015937
		SUPERFAMILY	SSF52540		1	159	IPR027417	H	
M. leachii	185	TIGRFAM	TIGR00152	TIGR00152: dephospho-CoA kinase	2	163	IPR001977	F	GO:0004140; GO:0005524; GO:0015937
M. leonicaptivi	187	Gene3D	G3DSA:3.40.50.300		1	183			
		PANTHER	PTHR10695:SF26		2	120			
		PANTHER	PTHR10695		2	120			
		Pfam	PF01121	Dephospho-CoA kinase	1	120	IPR001977	F	GO:0004140; GO:0005524; GO:0015937
		SUPERFAMILY	SSF52540		2	116	IPR027417	H	
M. lipofaciens	190	Gene3D	G3DSA:3.40.50.300		1	190			
		Pfam	PF01121	Dephospho-CoA kinase	2	137	IPR001977	F	GO:0004140; GO:0005524; GO:0015937
		SUPERFAMILY	SSF52540		2	124	IPR027417	H	
M. mobile	187	Gene3D	G3DSA:3.40.50.300		1	186			
		Pfam	PF01121	Dephospho-CoA kinase	1	156	IPR001977	F	GO:0004140; GO:0005524; GO:0015937
		SUPERFAMILY	SSF52540		2	180	IPR027417	H	
M. molare	182	Gene3D	G3DSA:3.40.50.300		1	177			
		Pfam	PF01121	Dephospho-CoA kinase	1	135	IPR001977	F	GO:0004140; GO:0005524; GO:0015937
		ProSiteProfiles	PS51219	Dephospho-CoA kinase (DPCK) domain profile.	1	182	IPR001977	F	GO:0004140; GO:0005524; GO:0015937
		SUPERFAMILY	SSF52540		2	137	IPR027417	H	
		TIGRFAM	TIGR00152	TIGR00152: dephospho-CoA kinase	1	153	IPR001977	F	GO:0004140; GO:0005524; GO:0015937

Mycoplasma species	Amino acid region	Database	Database ID	Database signature description	ID region start	ID region end	InterPro ID	ID type	Gene Ontology (GO) term
M. mycoides subsp. *capri*	185	CDD	cd02022	DPCK	2	175	IPR001977	F	GO:0004140; GO:0005524; GO:0015937
		Gene3D	G3DSA:3.40.50.300		1	179			
		PANTHER	PTHR10695:SF26		2	170			
		PANTHER	PTHR10695		2	170			
		Pfam	PF01121	Dephospho-CoA kinase	1	170	IPR001977	F	GO:0004140; GO:0005524; GO:0015937
		ProSiteProfiles	PS51219	Dephospho-CoA kinase (DPCK) domain profile.	2	185	IPR001977	F	GO:0004140; GO:0005524; GO:0015937
		SUPERFAMILY	SSF52540		1	170	IPR027417		
M. mycoides subsp. *capri*	185	TIGRFAM	TIGR00152	TIGR00152: dephospho-CoA kinase	2	174	IPR001977	F	GO:0004140; GO:0005524; GO:0015937
M. mycoides subsp. *mycoides*	189	CDD	cd02022	DPCK	6	179	IPR001977	F	GO:0004140; GO:0005524; GO:0015937
		Gene3D	G3DSA:3.40.50.300		2	185			
		Hamap	MF_00376	Dephospho-CoA kinase [coaE].	4	188	IPR001977	F	GO:0004140; GO:0005524; GO:0015937
		PANTHER	PTHR10695		5	163			
		PANTHER	PTHR10695:SF26		5	163			
		Pfam	PF01121	Dephospho-CoA kinase	5	167	IPR001977	F	GO:0004140; GO:0005524; GO:0015937
		ProSiteProfiles	PS51219	Dephospho-CoA kinase (DPCK) domain profile.	6	189	IPR001977	F	GO:0004140; GO:0005524; GO:0015937
		SUPERFAMILY	SSF52540		4	163	IPR027417	H	
		TIGRFAM	TIGR00152	TIGR00152: dephospho-CoA kinase	6	167	IPR001977	F	GO:0004140; GO:0005524; GO:0015937
M. opalescens	187	Gene3D	G3DSA:3.40.50.300		1	180			
		Pfam	PF01121	Dephospho-CoA kinase	1	132	IPR001977	F	GO:0004140; GO:0005524; GO:0015937
		SUPERFAMILY	SSF52540		2	137	IPR027417	H	
M. ovipneumoniae (HAD-DPCK)	447	Gene3D	G3DSA:3.40.50.1000		2	267	IPR023214	H	
		Gene3D	G3DSA:3.40.50.300		268	431			
		PANTHER	PTHR10000		2	264			
		Pfam	PF08282	haloacid dehalogenase-like hydrolase	7	259			

Mycoplasma species	Amino acid region	Database	Database ID	Database signature description	ID region start	ID region end	InterPro ID	ID type	Gene Ontology (GO) term
		Pfam	PF01121	Dephospho-CoA kinase	269	369	IPR001977	F	GO:0004140; GO:0005524; GO:0015937
		SUPERFAMILY	SSF56784		2	263	IPR036412	H	
		SUPERFAMILY	SSF52540		266	410	IPR027417	H	
		TIGRFAM	TIGR01484	HAD-SF-IIB: HAD hydrolase, family IIB	6	229	IPR006379	F	
M. penetrans	206	CDD	cd02022	DPCK	14	143	IPR001977	F	GO:0004140; GO:0005524; GO:0015937
		Gene3D	G3DSA:3.40.50.300		3	153			
		Hamap	MF_00376	Dephospho-CoA kinase [coaE].	12	206	IPR001977	F	GO:0004140; GO:0005524; GO:0015937
M. penetrans	206	PANTHER	PTHR10695:SF35		14	200			
		PANTHER	PTHR10695		14	200			
		Pfam	PF01121	Dephospho-CoA kinase	14	144	IPR001977	F	GO:0004140; GO:0005524; GO:0015937
		ProSiteProfiles	PS51219	Dephospho-CoA kinase (DPCK) domain profile.	14	206	IPR001977	F	GO:0004140; GO:0005524; GO:0015937
		SUPERFAMILY	SSF52540		13	201	IPR027417	H	
		TIGRFAM	TIGR00152	TIGR00152: dephospho-CoA kinase	14	193	IPR001977	F	GO:0004140; GO:0005524; GO:0015937
M. pirum	194	CDD	cd02022	DPCK	7	179	IPR001977	F	GO:0004140; GO:0005524; GO:0015937
		Gene3D	G3DSA:3.40.50.300		1	194			
		PANTHER	PTHR10695:SF26		5	192			
		PANTHER	PTHR10695		5	192			
		Pfam	PF01121	Dephospho-CoA kinase	6	138	IPR001977	F	GO:0004140; GO:0005524; GO:0015937
		ProSiteProfiles	PS51219	Dephospho-CoA kinase (DPCK) domain profile.	7	194	IPR001977	F	GO:0004140; GO:0005524; GO:0015937
		SUPERFAMILY	SSF52540		6	192	IPR027417	H	
		TIGRFAM	TIGR00152	TIGR00152: dephospho-CoA kinase	7	185	IPR001977	F	GO:0004140; GO:0005524; GO:0015937
M. pneumoniae	200	CDD	cd02022	DPCK	2	156	IPR001977	F	GO:0004140; GO:0005524; GO:0015937

Mycoplasma species	Amino acid region	Database	Database ID	Database signature description	ID region start	ID region end	InterPro ID	ID type	Gene Ontology (GO) term
		Gene3D	G3DSA:3.40.50.300		1	199			
		Hamap	MF_00376	Dephospho-CoA kinase [coaE].	1	188	IPR001977	F	GO:0004140; GO:0005524; GO:0015937
		PANTHER	PTHR10695		2	156			
		PANTHER	PTHR10695:SF26		2	156			
		Pfam	PF01121	Dephospho-CoA kinase	1	156	IPR001977	F	GO:0004140; GO:0005524; GO:0015937
		ProSiteProfiles	PS51219	Dephospho-CoA kinase (DPCK) domain profile.	2	200	IPR001977	F	GO:0004140; GO:0005524; GO:0015937
		SUPERFAMILY	SSF52540		1	186	IPR027417	H	
M. pneumoniae	200	TIGRFAM	TIGR00152	TIGR00152: dephospho-CoA kinase	2	182	IPR001977	F	GO:0004140; GO:0005524; GO:0015937
M. primatum	190	Gene3D	G3DSA:3.40.50.300		1	188			
		Pfam	PF01121	Dephospho-CoA kinase	1	137	IPR001977	F	GO:0004140; GO:0005524; GO:0015937
		SUPERFAMILY	SSF52540		2	188	IPR027417	H	
M. pulmonis	172	CDD	cd02022	DPCK	1	170	IPR001977	F	GO:0004140; GO:0005524; GO:0015937
		Gene3D	G3DSA:3.40.50.300		1	171			
		Pfam	PF01121	Dephospho-CoA kinase	1	143	IPR001977	F	GO:0004140; GO:0005524; GO:0015937
		ProSiteProfiles	PS51219	Dephospho-CoA kinase (DPCK) domain profile.	1	172	IPR001977	F	GO:0004140; GO:0005524; GO:0015937
		SUPERFAMILY	SSF52540		2	137	IPR027417	H	
		TIGRFAM	TIGR00152	TIGR00152: dephospho-CoA kinase	1	143	IPR001977	F	GO:0004140; GO:0005524; GO:0015937
M. putrefaciens	186	CDD	cd02022	DPCK	2	168	IPR001977	F	GO:0004140; GO:0005524; GO:0015937
		Gene3D	G3DSA:3.40.50.300		1	185			
		PANTHER	PTHR10695		2	149			
		PANTHER	PTHR10695:SF26		2	149			
		Pfam	PF01121	Dephospho-CoA kinase	1	158	IPR001977	F	GO:0004140; GO:0005524; GO:0015937

Mycoplasma species	Amino acid region	Database	Database ID	Database signature description	ID region start	ID region end	InterPro ID	ID type	Gene Ontology (GO) term
		ProSiteProfiles	PS51219	Dephospho-CoA kinase (DPCK) domain profile.	2	186	IPR001977	F	GO:0004140; GO:0005524; GO:0015937
		SUPERFAMILY	SSF52540		1	161	IPR027417	H	
		TIGRFAM	TIGR00152	TIGR00152: dephospho-CoA kinase	2	162	IPR001977	F	GO:0004140; GO:0005524; GO:0015937
M. simbae	187	Gene3D	G3DSA:3.40.50.300		1	187			
		Pfam	PF01121	Dephospho-CoA kinase	1	144	IPR001977	F	GO:0004140; GO:0005524; GO:0015937
		SUPERFAMILY	SSF52540		2	157	IPR027417	H	
M. simbae	187	TIGRFAM	TIGR00152	TIGR00152: dephospho-CoA kinase	1	149	IPR001977	F	GO:0004140; GO:0005524; GO:0015937
M. sturni	187	Gene3D	G3DSA:3.40.50.300		1	187			
		SUPERFAMILY	SSF52540		2	117	IPR027417	H	
M. synoviae	168	Gene3D	G3DSA:3.40.50.300		1	161			
		SUPERFAMILY	SSF52540		2	124	IPR027417	H	
M. testudinis	205	CDD	cd02022	DPCK	2	175	IPR001977	F	GO:0004140; GO:0005524; GO:0015937
		Gene3D	G3DSA:3.40.50.300		1	198			
		PANTHER	PTHR10695:SF35		2	185			
		PANTHER	PTHR10695		2	185			
		Pfam	PF01121	Dephospho-CoA kinase	1	151	IPR001977	F	GO:0004140; GO:0005524; GO:0015937
		ProSiteProfiles	PS51219	Dephospho-CoA kinase (DPCK) domain profile.	2	201	IPR001977	F	GO:0004140; GO:0005524; GO:0015937
		SUPERFAMILY	SSF52540		1	188	IPR027417	H	
		TIGRFAM	TIGR00152	TIGR00152: dephospho-CoA kinase	2	173	IPR001977	F	GO:0004140; GO:0005524; GO:0015937
M. yeatsii	187	CDD	cd02022	DPCK	2	169	IPR001977	F	GO:0004140; GO:0005524; GO:0015937
		Gene3D	G3DSA:3.40.50.300		1	187			
		PANTHER	PTHR10695		2	115			
		PANTHER	PTHR10695:SF26		2	115			
		Pfam	PF01121	Dephospho-CoA kinase	1	163	IPR001977	F	GO:0004140; GO:0005524; GO:0015937

Mycoplasma species	Amino acid region	Database	Database ID	Database signature description	ID region start	ID region end	InterPro ID	ID type	Gene Ontology (GO) term
		ProSiteProfiles	PS51219	Dephospho-CoA kinase (DPCK) domain profile.	2	187	IPR001977	F	GO:0004140; GO:0005524; GO:0015937
		SUPERFAMILY	SSF52540		1	163	IPR027417	H	
		TIGRFAM	TIGR00152	TIGR00152: dephospho-CoA kinase	2	167	IPR001977	F	GO:0004140; GO:0005524; GO:0015937

[a]ID type abbreviations – H, Homologous superfamily; F, Family

[b]InterPro ID – IPR027417: P-loop containing nucleoside triphosphate hydrolase; IPR001977: Dephospho-CoA kinase; IPR036412: HAD-like superfamily; IPR023214: HAD superfamily; IPR006379: HAD-superfamily hydrolase, subfamily IIB

[c]GO term (Biological Process) – GO:0015937: Coenzyme A biosynthetic process

GO term (Molecular Function) – GO:0004140: Dephospho-CoA kinase activity; GO:0005524: ATP binding

Supplementary Table 15 DPCK MEME + motif locations

Mycoplasma species	*p*-value	Motif locations			
		**Motif 1**	**Motif 2**	**Motif 3**	**Motif 4**
M. sp. Ms02	5.40e-39	1-21	23-42	58-73	90-121
M. agalactiae	1.11e-57	1-21	23-43	58-73	90-121
M. alligatoris	1.89e-43	1-21	23-43	53-68	85-116
M. alvi	7.76e-20	2-22	23-43	58-73	-
M. anatis	6.14e-43	1-21	23-43	58-73	90-121
M. arginini	5.90e-52	1-21	23-43	57-72	89-120
M. bovigenitalium	6.45e-45	1-21	23-43	58-73	90-121
M. bovis	2.31e-56	1-21	23-43	58-73	90-121
*M. bovoculi**	5.64e-47	265-285	287-307	321-336	353-384
M. buteonis	2.98e-37	1-21	23-43	58-73	90-121
M. californicum	1.59e-43	1-21	23-43	58-73	90-121
M. canis	4.50e-39	1-21	23-43	57-72	89-120
M. capricolum subsp. *capricolum*	1.88e-24	6-26	30-50	63-78	-
M. capricolum subsp. *capripneumoniae*	5.67e-25	2-22	26-46	59-74	-
M. collis	1.28e-53	1-21	23-43	58-73	90-121
M. columbinum	4.89e-51	1-21	23-43	58-73	90-121

Mycoplasma species	*p*-value	Motif locations			
		Motif 1	Motif 2	Motif 3	Motif 4
M. columborale	2.88e-38	1-21	23-43	57-72	89-120
*M. conjunctivae**	4.10e-51	266-286	288-308	322-337	354-385
M. cricetuli	8.29e-41	1-21	23-43	57-72	89-120
M. crocodyli	3.68e-47	1-21	23-43	58-73	90-121
*M. dispar**	2.73e-58	268-288	290-310	324-339	356-387
M. felifaucium	5.30e-54	1-21	23-43	58-73	90-121
M. felis	4.01e-40	3-23	25-45	60-75	92-123
M. fermentans	1.28e-41	1-21	23-43	58-73	90-121
*M. flocculare**	3.42e-55	267-287	289-309	323-338	355-386
M. gallinarum	1.59e-45	1-21	22-42	57-72	89-120
M. gallisepticum	1.35e-26	9-29	31-51	66-81	-
M. genitalium	2.60e-29	2-22	25-45	60-75	-
*M. hyopneumoniae**	3.07e-57	267-287	289-309	323-338	355-386
M. hyorhinis	4.62e-51	7-27	36-56	70-85	102-133
M. imitans	9.80e-25	9-29	31-51	66-81	-
M. iners	2.08e-53	1-21	23-43	58-73	90-121
M. iowae	1.92e-23	11-31	33-53	68-83	-
M. leachii	3.03e-24	2-22	26-46	59-74	-
M. leonicaptivi	9.92e-43	1-21	23-43	58-73	90-121
M. lipofaciens	3.21e-47	1-21	23-43	58-73	90-121
M. mobile	7.43e-34	1-21	23-43	58-73	-
M. molare	1.02e-55	1-21	23-43	58-73	90-121
M. mycoides subsp. *capri*	8.30e-22	2-22	26-46	59-74	-
M. mycoides subsp. *mycoides*	1.38e-24	6-26	30-50	63-78	-
M. opalescens	7.78e-42	1-21	23-43	58-73	90-121
*M. ovipneumoniae**	4.21e-49	268-288	290-310	324-339	356-387
M. penetrans	2.96e-30	14-34	36-56	71-86	-
M. pirum	4.84e-25	7-27	28-48	63-78	-
M. pneumoniae	1.92e-27	2-22	25-45	133-148	-
M. primatum	2.97e-51	1-21	23-43	58-73	90-121
M. pulmonis	8.18e-55	1-21	23-43	58-73	90-121
M. putrefaciens	5.29e-22	2-22	26-46	60-75	-
M. simbae	2.38e-48	1-21	23-43	58-73	90-121
M. sturni	2.80e-43	1-21	23-43	58-73	90-121

***Mycoplasma* species**	***p*-value**	**Motif locations**			
		**Motif 1**	**Motif 2**	**Motif 3**	**Motif 4**
M. synoviae	5.90e-52	1-21	23-43	57-72	89-120
M. testudinis	5.80e-27	2-22	24-44	59-74	-
M. yeatsii	2.57e-21	2-22	26-46	60-75	-

*HAD-DPCK proteins
